# Comparative Mutational Analysis and the Glycosylation Patterns of a Peruvian Isolated Avian Influenza A Virus H5N1: Exploring Possible Viral Spillover Events Within One Health Approach

**DOI:** 10.3390/vetsci12040392

**Published:** 2025-04-21

**Authors:** Sandra Landazabal-Castillo, Lucero Alva-Alvarez, Dilan Suarez-Agϋero, Enrique Mamani-Zapana, Egma Mayta-Huatuco

**Affiliations:** Molecular and Clinical Virology Laboratory, National University of San Marcos, Lima 15081, Peru; lucero.alva@unmsm.edu.pe (L.A.-A.); dilan.suarez@unmsm.edu.pe (D.S.-A.); emamaniz@unmsm.edu.pe (E.M.-Z.); emaytah@unmsm.edu.pe (E.M.-H.)

**Keywords:** One Health, Influenza A viruses, H5N1, mutational analysis, N-linked glycosylations (NLGs) profile

## Abstract

(1) Background: The ongoing panzootic of highly pathogenic avian influenza virus (HPAIV) of subtype H5N1, clade 2.3.4.4b, has decimated wild/domestic birds and mammals’ populations worldwide with reports of sporadic cases in humans. (2) Methods: This study aimed to compare the mutational profile of H5N1 avian Influenza virus isolated from a Peruvian natural reserve, with recent data from other related international studies made in human and different species of domestic and wild birds and mammals. Briefly, the near complete protein sequences of the Influenza virus coming from a *Calidris alba* were analyzed at a multisegmented level, together with 55 samples collected between 2022 and 2024 in different countries. Moreover, the glycosylation patterns were also predicted in silico. (3) Results: A total of 603 amino acid changes were found among H5N1 viruses analyzed, underscoring the detection of critical mutations HA:11I, HA:211I, HA:336T, HA:492D, HA:527I, NA:10T, NA:269L, NA:405T, NP:377N, PA:57R, PA:68S, PA:322V/L, PA:432I, PB2:539V, PB1:207R, PB1:375N, PB1:264D, PB1:429R, PA-X:250Q, PB1-F2:65R, and PB1-F2:42Y, as well as PA:13V, PA-X:13V, PA20T, PA-X:20T, PA:36T PA-X:36T, PA:45S, PA-X:45S, PA:57Q, PA-X:57Q, PA:61I, PA-X:61I, PA:68S, PA-X:68S, PA:70V, PA-X:70V, PA:75Q, PA-X:75Q, PA:85T, PA-X:85T, PA:86I, PA-X:86I, PA:100I, PA-X:100I, PA:142E, PA-X:142E, PA:160E, PA-X:160E, PA:211I, PA-X:211Y, among others, considered of importance under the One Health perspective. Similarly, changes in the N-linked glycosylation sites (NLGs) predicted in both HA and NA proteins were found, highlighting the loss/acquisition or changes in some NLGs, such as 209NNTN, 100 NPTT, 302NSSM (HA) and 70NNTN, 68NISS, and 50NGSV (NA). (4) Conclusions: This study provides our understanding about the evolution of current Influenza A viruses H5N1 HPAIV circulating globally. These findings outline the importance of surveillance updating mutational profiles and glycosylation patterns of these highly evolved viruses.

## Simple Summary

Influenza A virus is a pathogen of significant global concern for public health and wildlife conservation. A deeper understanding of its mutational/glycosylation profile is essential, given its broad host range capabilities. This study provides the comparative variations found in all segmented proteins of the H5N1 viruses analyzed, some of the findings must be highlighted within the One Health framework.

## 1. Introduction

Influenza A virus (IAV) is an enveloped, negative-sense, single stranded, and eight segmented RNA virus, belonging to the genus *Alphainfluenza*, family *Orthomyxoviridae*, which is a major threat within a One Health framework of global concern for public health and wildlife conservation due to its highly contagious spread and the devastating impact it has caused in breeding colonies of different species of domestic and wild birds and mammals globally [1,2]. The ultimate record of these viruses across the Americas, spreading from Canada to the Antarctic and sub-Antarctic regions of South Georgia, the Sandwich Islands, and the Falkland Islands, has decimated animal populations, including places where high pathogenicity avian Influenza virus (HPAIV) had never previously been detected [3,4]; in regions of Peru, Chile, Argentina, Uruguay, and Brazil, the spreading of the virus has particularly affected marine mammal populations with increasing evidence of mammal–mammal transmission, and comprises mutations that were not reported in H5N1 viruses in other regions [5,6,7]. Likewise, the emerging infections of H5N1 avian origin virus reported in cattle [8,9], wild carnivores [10,11], domestic mammals [12,13], as well as reports of human infections [14] highlighted the characteristics of these exceptionally evolved viruses and the risk of possible spillover events.

Furthermore, the continuous outbreaks of Influenza H5N1 HPAIV clade 2.3.3.4b viruses never seen before worldwide represents a serious threat, given the ideal conditions to share key mutations between different species, including hosts with the potential to act as mixing vessels because they carry both avian and human-type receptors. The genetic variations acquired by antigenic drift (minor mutational changes over time) or shift (abrupt major change in the virus where genome segments may be rearranged to another subtypes), have increased the opportunities of viral evolution, and the emergence/reemergence of new virus strains. Nowadays, the risk of H5N1 host tropism broadening with the possibility of a panzootic/pandemic is looming; therefore, a primordial concern centers around the virus’s capabilities for genetic changes or reassortment events [15]. Hence, active surveillance of influenza mutational profiles is required, providing the opportunity to assess the risk posed by the different subtypes/clades/genotypes, that may enhance the mammalian adaptation, spillover events, and the likelihood of reassortment events, particularly H5N1 clade 2.3.4.4.b virus, quantifying their zoonotic potential.

A risk assessment framework was recently made to characterize the variations related to phenotypic traits for the adaptation to new hosts based in epidemiological and spatio-temporal data analysis of several Influenza viruses. A total of 592 mutations where identified, where 34 of them were associated with an increase in zoonotic potential, and five phenotypic traits (haemagglutinin stability, neuraminidase specificity, major receptor specificity, improved polymerase activity, and immune evasion). It was featured that H5 viruses of clade 2.3.4.4.b have acquired the highest number of zoonotic traits (up to four), in the European Union, during the last three years; emphasizing that there is still limited evidence, poor information available or not investigated for further mutations [16]. In addition, though the information related to certain mutations, and these biological properties, is scarce, it has been observed that some adaptational variations to new hosts can be provided through mutations [17,18]; such as NA:L204M, which enhanced viral replication, receptor binding, and virulence; PB2-E627K, which increased viral replication in mammals or HA:156A/V, which increased affinity for α2–6 the human type receptor. These are only examples of the high plasticity of these segmented viruses. Likewise, it is well known that the lofty variability of Influenza viruses presented over time during outbreaks, such in the case of two Influenza A/H1N1 pandemic viruses from 1918, that compared to the reference genome A/Brevig Mission/1/1918 (H1N1), a total of 76 SNPs from formalin-fixed, paraffin-embedded lung tissues were identified, of which 46 encode non-synonymous (NS) changes [19].

Additionally, key mutations that occurred in HA and NA proteins can vary the biophysical properties of Influenza viruses when N-linked glycosylation motifs are introduced/removed, which can facilitate immune evasion or increase the receptor binding affinity to maintain viral fitness [20]. It is well known that variable degrees of glycosylation confer capabilities to the globular head and stem of the HA ectodomain to bind host-derived glycans and hide or expose the functional region, a process required to initiate the cell viral entry [21]; moreover, glycosylation of NA protein has been related to changes in protein folding, stability, solubility, and viral budding, as well as being related to the neurovirulence of Influenza viruses [22,23]. In addition, the glycosylation dynamics in the current H5N1 Influenza viruses are not yet fully understood at all.

Given the critical role of mutations and glycosylation patterns in the dynamics of Influenza virus and its adaptive evolution, this study describes the comparative results obtained between several H5N1 Influenza virus genomes, mammal and non-mammal, within One Health approach.

## 2. Materials and Methods

### 2.1. Sample Collection

One duplicate sample oropharyngeal swab was collected from a *Calidris alba* with slowness fly and stagnation from Pantanos of Villa a Peruvian National Reserve during the period of time from March–April 2023. The material was transported and preserved in a triple packaging system to perform the molecular detection of Influenza A viruses following the standard diagnostic procedures by amplification [24].

### 2.2. Molecular Detection

Viral RNA was extracted using the Viral Nucleic acid extraction kit II (Geneaid, New Taipei, Taiwan) according to the manufacturer’s instructions in a class 2A biological safety cabinet (Biobase, Jinan, China). The extracted RNA was subjected to purity measurement using a spectrophotometer DS11 instrument (Denovix, Wilmington, USA) and Influenza virus detection was performed using a high-resolution melting analysis (HRM) procedure to target the M and HA gene following the standard procedures of World Organization of Health [25].

### 2.3. Whole-Genome Sequencing

Whole-genome sequencing (WGS) of amplicons per each genome segment of the isolate H5N1 virus were obtained using Mytaq^®^ Red DNA Polymerase kit (Meridian Bioscience, Cincinnati, OH, USA) and the sequencing was performed using Illumina next generation sequencing (NGS) technology (Miseq system with a 250-cycle paired-end). The reads were analyzed according to the tools described in our previous publication [26].

### 2.4. Data Sets

Full-length or near full-length protein sequences of 55 highly pathogenic avian Influenza A viruses (HPAIV) H5N1 were downloaded from the National Center for Biotechnology Information (NCBI), together with data from the Peruvian isolate H5N1.A/Calidris alba/Lima/Villa01/2023, within One Health perspective. The sample list included available data from outbreaks in 2022 and 2024, in the Americas region mainly and considered of importance to be included in the analysis. The criteria for data selection were geographic; host diversity (representative species of mammals/birds, some without a previous report of infection with H5N1 virus of avian origin); availability of complete/near complete protein sequences; data quality reports, and related recent publications. All sequences obtained from GenBank are listed in Supplementary S1, Table S1. All Influenza virus proteins were included in the perusal: PB2, PB1, PA, HA, NP, NA, M, and NEP (NS2), and the accessible information of Influenza virus accessory proteins were taken into special consideration.

### 2.5. Mutational Analysis and Genotype Identification

The data obtained at a multispecies level were analyzed in the search of amino acid changes, segment by segment manually and in silico, using Clustal Omega v12.4 [27], along with the command-line tool FluMut [28] and the web application Flusurver [29]. All available sequences of PB2, PB1, PA, HA, M, NP, NA, and NEP (NS2) were compared, including the accessory proteins PA-X, PB2-F1, and NS1. Genotype was determined using GenoFLU (https://github.com/USDA-VS/GenoFLU (accessed on 26 January 2025)) [30].

### 2.6. Prediction of Potential N-Glycosylation Sites in HA and NA Influenza A H5N1 Viruses

Potential N-linked glycosylation sites (NLG) of the sequences HA and NA proteins of the analyzed H5N1 viruses were predicted and compared with the reference genome (A/goose/Guandong/1996(H5N1) using the high prediction accuracy NetN-Glyc 1.0 server [31], detecting the Asn-X-Ser/Thr sequons (where X is any amino acid except proline) [32], and measures of prediction confidence >0.5 were taken into consideration as a threshold.

## 3. Results

### 3.1. Mutational Manual Analysis

**Figure 1:**
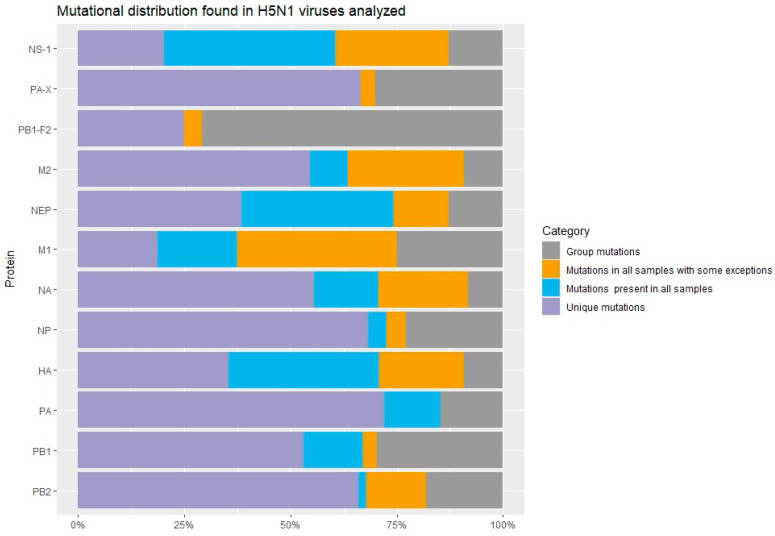
Mutational distribution found in the H5N1 viruses analyzed. The mutations were classified into 4 categories: unique mutations (mutations found only 1–3 samples), group mutations (mutations found in more of 4 samples), mutations present in all samples, and mutations found in almost all samples with some exceptions.

Through mutational manual analysis of the selected 55 samples of H5N1 Influenza viruses, coming from both birds and mammals, a total of 603 amino acid changes were identified, distributed throughout the 8 protein segments PB1, PB2, PA, HA, M, NA, NP, NEP, and the 4 accessory proteins M2, NS1, PB1-F2, and PA-X; where 76 mutations have been detected in the HA protein; 72 mutations in the NA protein; 22 mutations in M1; 11 mutations in M2; 22 mutations in NP protein; 94 mutations in NS1 protein; 39 mutations in NEP (NS2) protein; 56 mutations of PB2 protein; 64 mutations in PB1 protein; 48 mutations PB1-F2; 30 mutations in PA-X; and 75 mutations in PA protein (Figure 1). The complete mutational details can be observed in Supplementary S1 and S2.

It is noteworthy that the proportion of individual/group mutations detected per each protein segment, varied notoriously as follows: individual mutations (those found only in 1–3 of the samples evaluated) were observed more in PA, PB1, PB2, NP, NA, and PA-X proteins; while group mutations (those identified in more than 4 samples), were detected in larger quantities in PB1-F2 and PB1 proteins. In addition, more mutations in “all” samples analyzed, were found in M1 and NS1 proteins (S1 (Table S2)).

Furthermore, considering the size of each virus protein segment, the detection of more quantity of amino acid variations in the accessory proteins PB1-F2, NS1, and NEP, particularly in the “PB1-F2 protein” was conspicuous. Viruses such as A/LOU/WT, polar bear/ALK, and harbor-seal/ME, along with certain birds species shared 23 variations in PB1-F2 protein; likewise, they also had in common 7 mutations in PB1, 4 mutations in HA, 2 mutations in NS1, 1 mutation in PA-X, as well as group mutations PB1:375N, HA:492D, PB1-F2:22E, PB1-F2:90N, NS1:83P, and PA-X:215N. Similarly, a considerable number of individual mutations were found in proteins, NA (19 variations detected in A/LOU/WT, 3 in polar-bear/ALK), and PA (13 variations detected in A/LOU/WT, 6 in polar-bear/ALK). Additionally, the individual mutations NA:44N, NA:45H, NA:48T, NA:53V, NA:62I, NA:81D, NA:82P, NA:84A, NA:234I, NA:286S, NA:288V, NA:399L, and PA:201I/T, PA:211I, PA:322L, PA:399V, PA:626R were also found in these isolates. Keeping in mind that these specimens are the most recent isolates and considering the host species, it should be considered under the One Health perspective (Table 1 and Table 2).

Moreover, unique and/or group mutations stands out among other North/South America specimens, highlighting that many of them have also been found in both mammals and birds, such in case of individual variations shared found in North American isolates: A/CA, emu/CA (HA:104G, HA:336N, PA:68S, PA:486M, PA:655F, PA-X:68S, PB2:670R, NS1:67G, NP:119V); cattle/TX (M2:27A, PA:36T, PA:404S, PA-X:36T, PB1:384T, NEP:60S); South American Chilean-dolphin, elephant-seal/ARG, and terns/ARG (NS1:26K, NSI:226T, NP:119T, PA:57Q, PA-X:57Q, PA:86I, PA-X:86I, PA:336M, PA-X:20T, PB2:152V, PB1:40I, PB1:548F, PB1:515A, PB1:621K. Similarly, other specimens also had their own particular mutations, such as in South America: Panthera-leo/PER (HA:310V, PA:45S, PB2:190R, NEP:89V, NP:425V), Calidris-alba/LIM (HA:201R, NA:442I, M2:52S, PB2:679S, NS1:213L, NEP:56Y, NP:190A); black-necked-swan/UGY (PA:425F, PB2:199T, NP:323S, PB1-F2:90I); Andean-guayata/ARG (HA:9V); and in North America: peregrine-falcon/NY (NA:223T, NA:237F, M2:19Y, PA-X:118V, PA-X:207L, PA-X:250P, PB2:472D), vulture/FL (HA:87T, HA:102T, PA:59G, PA:272N, PA-X:59K, PB2:453S, PB1:431H, NEP:82E, PB1-F2:41L, PB1-F2:69L); harbor-seal/ME (HA10T, HA:152S, HA:226T, PA:465T, PA-X:70V, PB2:79G, PB2:715S, PB1:176T, PB1:372I, PB1:660I, NS1:67Q, PB1-F2:73E), goat/MI (HA:520R, PA:614S, PB2:274V, PB2:346A, PB2:353R, PB2:663R, PB2:667I, PB1:211K, NS1:36I, NS1:136M, NS1:201Y); raccoon/IA (M2:28T, PA:100I, PB2:539V); house-mouse/NM (NA:254R, PA:13V, PB1:384P, NS1:77R); alpaca (PA:142E, PA-X:142E), and red-fox/MI (PB2:596A). Additionally, the group mutations PB1:515A, NS1:26K, PA-X:86I and PA-X:57Q were detected in the South American mammals and birds analyzed.

Further, certain samples accumulated a greater quantity of individual/group amino acid changes in different protein segments such as in case of birds coming from Brazil (Numida/BR, Procellaria/BR mainly) and the specimens coming from Bangladesh, Japan, Egypt, and Germany included within the present study. (Table 2 and Table 3).

In addition, a number of mutations were found in “all” specimens, (concerning the reference genome) (Table 3), and in other cases some specimens had mutations shared in almost all samples with little exceptions detected (Table 4). Be aware that several of these mutational changes could have been transmitted over time at a multispecies level, bringing them with/without a specific trait, effect, or significance; nonetheless, these were common findings even in specimens far away from our geographical region.

In general, during the mutational scanning, certain mutations were acquired in all samples tested, whilst others were only detected in certain groups of them. But, evidently, there was an existing “different” mutations distribution between the North American/South American isolates. Also, among the North American samples, two large groups with similar amino acid changes were distinguishable: a first large group with certain homogeneity in the mutations found, and the second smaller that included the most recent specimens that shared new mutations, of which there are no still studies on the possible biological function, such as PB1-F2:4G, PB1-F2:7I/T/M, PB1-F2:8Q, and PB1:12S among others (complete mutational scanning can be seen in Supplementary S2.

### 3.2. FluMut Mutational Analysis

Our mutational analysis, were compared with the results obtained with FluMut program, and a match in the detection of the next mutations/molecular markers was found as follows: HA:154N, HA:156A, M2:27I; NA:155H, NA:223T, NA:364N; NS1:53D, NS1:55E, NS1:66E, NS1:74N, NS1:205S, NS1:210R; NS2:48A; NP:41V, NP:105V; PA:63I, PA:142E, PA:190S, PA:497R; PB1:207R, PB1:375S, PB1:598L; PB1-F2:56A, PB1-F2:66S; PB2:9N, PB2:292V, PB2:339K, PB2:495I, PB2:590S, PB2:631L, PB2:676T, PB2:699R, PB2:715N; emphasizing that some of the mutations presented amino acid changes in the mutation designated, such as in the case of HA:154N (HA:154Q), or NA:53D (NA:53G), and taking into account, as previously mentioned, that certain mutations were found in all samples whilst others were only detected in individual samples. (Complete FluMut mutational analysis can be seen in Supplementary S3.

### 3.3. Genotype Identification

Four genotypes were found in the H5N1 viruses analyzed: B3.13(30.5%), B3.2(37.3%), B1.3(3.4%), A3(3.4%), A2(1.7%), B1.1(1.7%); and in (22%) of specimens the genotype was not assigned because not all the segments matched the total of segments in input file. (Complete Genoflu genotype determination can be seen in Supplementary S4.

### 3.4. Glycosylation Patterns

#### 3.4.1. N-Linked Glycosylations (NLG) in the HA of Influenza H5N1 Viruses

A total of 7 N-linked glycosylations were found in HA protein of the reference genome (A/goose/Guandong/1996(H5N1) at positions 27 NSTE, 39 NVTV, 181 NNTN, 209 NPTT, 302 NSSM, 500 NGTY, and 559 NGSL, respectively, taking into consideration that 209 NPTT position had included a warning (Pro-X1), due the presence of a proline in the sequon. The complete list of glycosylation predictions is Supplementary S5. Particularly, the specimens that had an absent 209 NPTT NLG, had a change in the amino acid position 211 from T in the HA protein.

**Figure 2:**
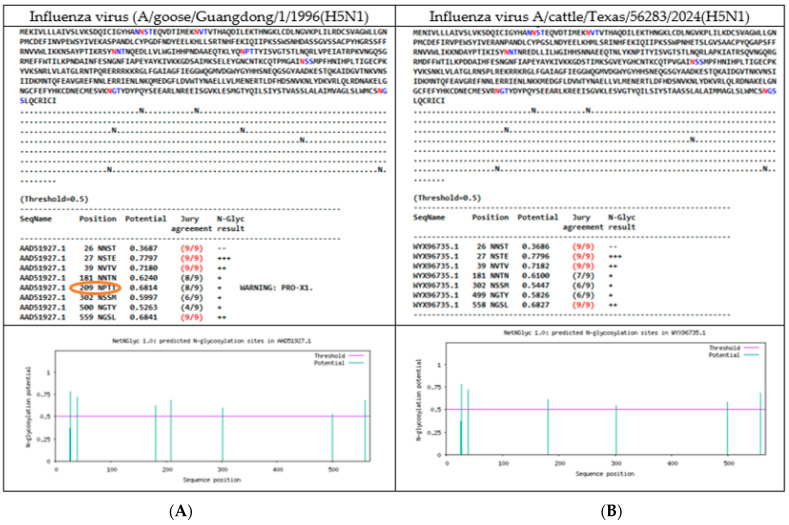
Comparison of glycosylation sites for HA protein of H5N1 viruses: (**A**). Reference genome Influenza A virus (A/goose/Guangdong/1/1996(H5N1) had 7 NLGs predicted; (**B**). Influenza A virus/cattle/Texas/56283/2024, had 6 predicted NLGs, with substitution of 2 NLGs (499 NGTV and 558 NGSL) and is highlighthed the loss of the 209 NPTT site (bold in orange).

For instance, the isolates coming from North America had two characteristics (presence or absence of 209 NPTT NLG): first, 4 mammal specimens (2 A/LOU/WA, 1 harbor-seal/ME/ME, 1 polar bear/AK), and 6 birds ones (peregrine-falcon/NY, goose/AK, Northern-pintail/USA, chicken/JPN, duck/BD, white-tailed-eagle/JPN, turkey/GER and pintail/EG) had the predicted 209 NPTT glycosylation site, and showed 7 NLGs; second, 16 mammal samples (4 A/CA/MT/CO, 1 alpaca/ID, 3 bovine/TX/ID, 2 feline/TX/ID, 1 red-fox/MI, 2 goat/MI, 1 raccoon/USA, 1 house-mouse/NM, 1 domestic cat/TX), and 3 bird samples (Emu, chicken/Idaho and grackle/Texas) only had 6 NLGs predicted and had lost 209 NPTT. A comparison between the glycosylation sites predicted (HA protein) to the reference genome and the specimen cattle/TX can be observed in Figure 2.

In the case of mammal isolates coming from South America (elephant-seal/ARG, Panthera-leo/PE and dolphin/CHIL), as well as the 19 bird isolates (Andean-guayata/ARG, Sterna-hirundo/BR, Humboldt-penguin/CHIL, Procellaria-aequinoctialis/BR, Numida-meleagris/BR, cormorant/CHIL, belcher-gull/PE, wild-duck/CO, 2 pelecanus/PE, backyard-duck/UY, Fregata-magnificens/BR, chimango-caracara/CHIL, black-necked-swam/UY, royal-tern/AR, Calidris-alba/PE, gallus-gallus/PE, Thalasseus-acuflavidus/BR and South America-tern/ARG) all of these had 7 NLG, including 209 NPTT.

Likewise, the glycosylation predictor showed a substitution in the NLG site to 499 NGTY and 558 NGSL (from 500 NGTY and 559 NGSL of the reference genome) in all specimens analyzed. This ultimate characteristic coincided with the presence of a mutation in the amino acid sequence prior to the NGTY sequon, from K to R in position 499. In addition, a slight increase in the glycosylation potential was seen as 0.5263 (reference genome) versus 0.58 found in all samples analyzed.

Furthermore, 2 North American avian isolates (chicken/JPN and vulture/FL) had 8 predicted NLGs, with an extra NLG site as 100 NPTN, which also concurred with a mutation in the HA protein, 102 site from A to T amino acid; the feature was only shown in these two isolates. It should be noted that a 100 NPTN glycosylation prediction was found between samples very far away geographically.

**Figure 3:**
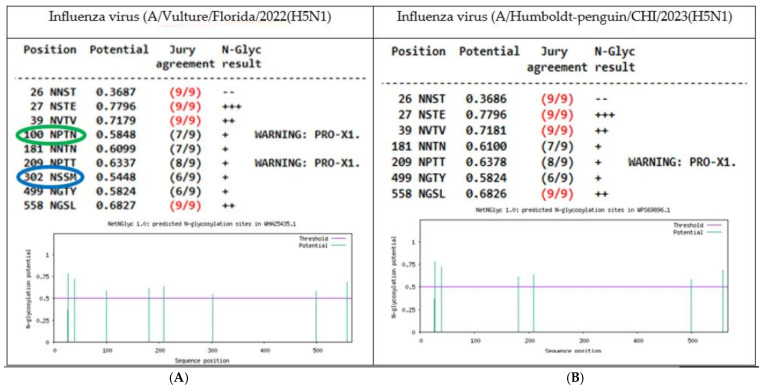
Comparison of glycosylation sites for HA protein of H5N1 viruses: (**A**). Left. North American Influenza A virus (A/Vulture/Florida/2022(H5N1) had 8 predicted NLGs, with a gain of 100 NPTN site (green bold); (**B**). Right. South American Influenza A virus/Humboldt-penguin/CHI/56283/2023(H5N1) had 6 NGLs predicted with loss of 302 NSSM site (blue bold).

In addition, two South American avian samples (Sterna-hirundo/BR and Humboldt-penguin/CHL) showed 6 NLGs with a loss of glycosylation site 302 NSSM. In both cases, a substitution was observed in the amino acid 304, where S was replaced by N. A comparison between the North American specimen (A/Vulture/Florida/2022(H5N1) with 8 NLGs glycosylation sites predicted (HA protein) versus the South American (A/Humboldt-penguin/CHI/2023(H5N1) with 6 NLGs is shown in Figure 3.

#### 3.4.2. N-Linked Glycosylations in the NA of Influenza H5N1 Viruses

The predicted NLGs in NA protein of the reference genome (A/goose/Guandong/1996(H5N1) found 7 sites of glycosylation at 50 NQSI, 58 NNTW, 63 NQYT, 68 NISN, 88 NSSL, 146 NGTV, and 253 NGSC positions, and differences in the predicted glycosylation positions were found in both mammal and bird specimens.

First, the 3 South American mammals’ isolates (elephant-seal/ARG, Panthera-leo/PE and dolphin/CHIL), as well as 17 South America avian isolates (Andean-guayata/ARG, Sterna-hirundo/BR, Humboldt-penguin/CHIL (Figure 3), Procellaria-aequinoctialis/BR, Numida-meleagris/BR, cormorant/CHIL, belcher-gull/PE, pelecanus/PE, backyard-duck/UY, Fregata-magnificens/BR, chimango-caracara/CHIL, black-necked-swam/UY, royal-tern/AR, Calidris-alba/PE, gallus-gallus/PE, thalasseus-acuflavidus/BR and South America-tern/ARG) presented the same 7 NLGs positions described for the reference genome. Nevertheless, the wild-duck/CO specimen had a substitution from 68 NISN to 70 NNTN. This same feature was also seen in the 2 avian isolates (duck/BD, white-tailed-eagle/JPN), and (polar-bear/ALK) a sample of mammal origin.

In the case of North American isolates, 2 avian samples (vulture/FL and Goose/AK, shared the same patron of glycosylation with NA reference genome, while 3 specimens (Emu/CA, grackle/TX, chicken/ID) had the amino acid change from N to S in position 68 (68NISN to 68NISS) of NA protein. It is worth highlighting that this characteristic was also observed in 11 North American mammal isolates (2 A/CA, and 1 A/CO, 1 domestic-cat/TX, 1 alpaca/ID, 3 bovine/TX/ID, 2 feline/TX and 1 racoon/USA), along with a substitution in the amino acid of the 67 position from V to I, just prior to the sequon location. Additionally, the potential of glycosylation predicted in this particular site was quite variable: from 0.73 in the reference genome, 0.67–0.68 in samples 68 NISN, such as (A/LOU/WT/MO, red-fox/MI and cat/TX isolates) or 0.71 in samples that presented the variation 68 NISS.

**Figure 4:**
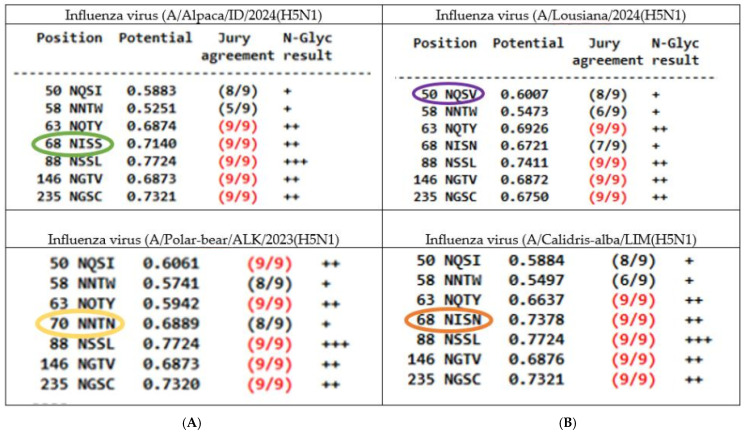
Comparison of glycosylation sites predicted for NA protein of H5N1 viruses: (**A**). Left/up: Influenza virus (A/Alpaca/ID/2024(H5N1) had 7 NLGs, with a 68 NISS site (green bold); left/down: Influenza virus (A/polar-bear/ALK/2023) had 7 NLGs, with a 70 NNTN site (yellow bold) (**B**). Right/up: Influenza virus (A/Louisiana/2024(H5N1) had 7 NGLs with the substitution 50 NQSV (purple bold); right/down: Influenza virus (A/Calidris-alba/LIM(H5N1) had 7 predicted NGLs with 68 NISN (orange bold).

Furthermore, two recent North American mammal isolates (2 A/LOU/WT) had the unique characteristic 50 NQSV predicted, with a slight increase in glycosylation potential from 0.50 to 0.60 compared with other samples (50 NQSI), for instance the reference genome had a potential of 0.55 in this NLG predicted site. A comparative scheme of the predicted NLGs glycosylation sites (NA protein) of 4 representative isolates can be seen in Figure 4.

Conversely, 1 North American avian sample (peregrine-falcon/NY) had a change from 235 NGSC to 221 NNTL, with a notable reduction in the glycosylation potential from 0.73 to 0.47. In contrast, a decrease in the glycosylation potential was also observed in the avian sample (Northern-pintail/USA) and mammal samples (A/LOU/WT) to 0.67. Likewise, these 3 samples (Northern-pintail/USA and 2 A/LOU/WT) had a mutation detected in position 234 where V was replaced by I.

On the other hand, the sample (turkey/GER) had 6 NGLs, due to the loss of 88 NSSL glycosylation site predicted, and it matches with a substitution in the amino acid of 90 position from S to P. In addition, the isolate (chicken/JPN) showed a slight increase in the glycosylation potential at the 146 NGTV site of 0.68 to 0.7377, accompanied by a substitution in the amino acid position 150 from K to E.

## 4. Discussion

Noteworthy, the threat of Influenza H5N1 viruses globally within the One Health framework is undeniable, and one of the significant challenges of our era is to focus on limiting exposure and preventing the virus spread. Considering animal welfare, and the protection of threatened species is a breaking point, in order to maintain a sustainable connection between nature and people. Current facts such as the overexploitation of wild and domestic animals, unsustainable production systems [33], as well as poor water quality, with high loads of ubiquitous environmental pollutants, such as heavy metals (with immunosuppressive effects) [34,35], strongly impact aquatic habitats, altering water/sediment quality, and it directly affects microorganisms and birds/marine mammals, the principal reservoirs, and victims of Influenza viruses. Therefore, thinking about ecology facts and holistic perspectives to mitigate the transmission risk (including zoonosis reverse) between mammals and humans is so crucial. Likewise, the understanding of Influenza evolution plays a vital role in the mutations analysis and enhances viral fitness over time, and what better example than the amino acid change HA:190D in the pandemic Influenza virus of the 1918 H1N1 that switched its binding preference from SAα2,3Gal to SAα2,6Gal [36].

In the extended listing of amino acid variations in the present study, it was observed that some of them have already been described or studied, but the majority of recent mutations found still have unknown biological functions. Certain variations were detected in both bird and mammal species highlighted in spite of these having been classified previously as a warning level 1 [8] or having been detected with a different amino acid combination have been considered of importance: HA:11L, HA:52A, HA:104G, HA:152S, HA:211I, HA:336N/R, HA:492D, HA:527I, NA:6R, NA:10T, NA:70N, NA:405T, NA:234I, NA:329S, NA:436V, NP:377N, PA:354F, PA:545V, PA:614D/S, PA-X:250Q, PB2:9N, NS1:67G/Q, NP:119V/T, PA:322V/L, PB2:532L, PB2:539V, PB2:666I, PB2:670R, PB1:211K, PB1:384P/T/A, PB1:621K, PB1:738G, PB1:16D, PB1:154S, PB1:172D, PB1:207R, PB1:215K, PB1:264D, PB1:375N, PB1:548F, PB1:694S, PB1-F2:4G, PB1-F2:7I/T/M, PB1-F2:8Q, PB1-F2:12S PB1-F2:17S, PB1-F2:18T, PB1-F2:20R, PB1-F2:21R, PB1-F2:22E, PB1:36T, PB1-F2:40G, PB1-F2:42Y, PB1-F2:49A, PB1-F2:56A, PB1-F2:57C/F, PB1-F2:58W, PB1-F2:65R, PB1-F2:75L, PB1-F2:82S, PB1-F2:84S, PB1-F2:90N, PA-X:193S, PA-X:245N. Moreover, mutations found in only 1 animal (mammal): HA:226T [37], HA:310V; HA:520R, HA:432R, PA:336M, PA:351G, PA:399V, PA:404S, PA:459V, PA:486M, PA:489S, PA:538G, PA:655F, PA:655F, PB2:79G, PB2:152V, PB2:190R, PB1:251K, PB2:190R, PB2:251K, PB2:255A, PB2:274V, PB2:346A, PB2:353R, PB2:596A, PB2:663R, PB2:667I, PB2:670R, PB2:677K, PB2:715S, PB1:40I, PB1:171A, PB1:291A, PB1:390G, PB1:533S, PB1:660I, may also be considered for future analysis based on the limited information associated. In general, a greater number of mutations were detected in North America isolates, particularly in the most recent ones coming from mammals.

Likewise, a striking fact was observed in certain mammal isolates: the presentation of the same amino acid variation in both PA and PA-X proteins simultaneously, such as case of mutations PA:13V, PA-X:13V, PA:36T PA-X:36T, PA:45S, PA-X:45S, PA:70V, PA-X:70V, PA:75Q, PA-X:75Q, PA:142E, PA-X:142E, PA:160E, PA-X:160E, PA:211I, PA-X:211Y. In addition, the following group mutations are noteworthy and warrant further research considering the key role of the PA protein in the emergence of pandemic viruses: PA20T, PA-X:20T, PA:57Q, PA-X:57Q, PA:85T, PA-X:85T, PA:86I, PA-X:86I, (South America mammals/birds) and PA:61I, PA-X:61I, PA:68S, PA-X:68S, PA:100I, PA-X:100I (North America mammals/birds). The 2009 H1N1 (H1N1pdm09) virus gained multiple mutations in the PA gene through activation polymerase activity in mammalian cells, even in the absence of previously identified host adaptive mutations in others polymerase genes. In the same way, the activity of the accessory protein PA-X varies between different hosts and could play a role in host adaptation [38]. Now, studies of H5N1 viruses have found that mutations PA:343T and PA:383D could activate polymerase activity in human cells in the “absence” of key mutations in PB2, such as E627K or D701N [39,40]. Additionally, considering the most recent mammal isolates of H5N1 viruses have accumulated too many new mutations mainly in NA and PB1-F2 proteins (NA:23V, NA:44N, NA:45H, NA:48T, NA:53V, NA:62I, NA:67I, NA:74L, NA:75I, NA:81D, NA:82P, NA:84A, NA:221S, NA:241I, NA:257R, NA:286S, NA:288V, NA:329S, PB1-F2:4G, PB1-F2:7I/T/M, PB1-F2:8Q, PB1-F2:12S, PB1-F2:18T, PB1-F2:20R, PB1-F2:21R, PB1-F2:22E, PB1:36T, PB1-F2:40G, PB1-F2:42Y, PB1-F2:49A, PB1-F2:58W, PB1-F2:65R, PB1-F2:75L, PB1-F2:82S, PB1-F2:84S), as well as PA:201V, PA:322V, PA:348L, PA:626R, PA-X:195K), it is recommended to analyze these variations carefully, in particular PB1-F2 due to the high number of changes found in a protein of only 87–90 amino acids, for which data from the reference genome (A/goose/Guandong/1996(H5N1) is not available. A study carried out in highly pathogenic H5N1 avian influenza viruses and the H1N1 pandemic strain of 1918 found that a single point mutation from N to S at position 66 of the PB1-F2 protein dramatically increased the virulence, and identified the antagonism to interferon [41,42]. Also, it is well-known that PB1-F2 had proapoptotic functions in immune cells and a pathogenicity potential increased through of dysregulation of the inflammatory response [43].

In addition, some key mutations associated with changes in glycosylation patterns have previously been observed such as HA:304N, NA:70N, NA:90P, NA:237F, NA:364N [44], and NA:329S [45]. Our specimens analyzed had included to the list: HA:211A, HA:499R, HA:102T, NA:67I, NA:234I, NA:150E and NA:50V. Its variations were found between mammals and non-mammals’ species, hence future analysis is required. Glycosylation process is an indispensable factor for the infectivity, survival and transmissibility of the Influenza virions and NLG are an essential component in the adaptation of Influenza viruses to new hosts [23]. In the case of HA protein, the addition of oligosaccharides has been associated with changes in the ability to bind to cellular receptors, interaction with neutralizing antibodies (immune evasion), properly protein fold, fusion process, efficient transport, stability, fit budding, and virulence [22,46]; nonetheless, the exact function acquired through of a glycosylation is related with the “specific” area of HA is being strengthened: HA stem region is paramount for the membrane fusion, and correct protein folding; while the head region is related to mislead the immune system (masking antigenic sites of the receptor binding domain) [32], as well as the receptor-binding site (RBS), that influence viral receptor binding preferences [47]. Studies of the H1N1 virus have shown first that different antibody responses when HA protein was glycosylated [48] and second the appearance of a new glycosylation site (179) in HA protein, observed exclusively during the specific evolutionary phases of the seasonal virus strain, and it was suggested that the association of the emergence of this new glycosylation site with the increased incidence of Influenza A cases in 2023 [49]. On the other hand, the functions of glycosylation’s in NA protein remains still unclear, there is relatively little research on this protein [22,50], but due to the opposing roles of HA and NA, it is recommended that both proteins should be studied jointly: the stability between HA binding and NA cleavage action is “essential” for overcoming host barriers and adaptation to new host species; so any important change observed in glycosylation patterns of any of these proteins, should be considered in detail since it could be a predicting feature of future pandemic/panzootia of Influenza viruses [47]. Our understanding of how glycosylation affects viral fitness is still limited; thus, it is recommended to carry out complementary analysis such as protein modeling and mass spectrophotometry based on comparative proteomics [51] to discern more about all the NLGs differences found in H5N1 viruses, especially when new glycosylation forms might appear and occur suddenly in a near future [32].

Otherwise, an important point to stand out under One Health approach is the finding of mutations associated with possible mild/strong antiviral resistance such as case of amantadine in mutation M2:27A/I (2 mammals, 1 bird) [52], as well as, NA:436V (1 mammal, 1 bird) [53] and NA:432R (1 mammal) [54]. Although, they have only been detected in a few animals, this fact is particularly important because there are mutations related to antiviral resistance to amantadine or oseltamivir. Furthermore, other variations detected, NA:103D, NA:106I, NA:107R, NA:141N/D, NA:142D, NA:144H, NA:153S, NA:188I/F, NA:248N, NA:354G, NA:400S, NA429G, NA:438T, have shown different sensitive grades to oseltamivir, zanamivir, peramivir, and laninamivir [29]. The antiviral resistance has been more widely studied in H1N1 and H3N2 viruses, reporting the sporadic emergence of drug-resistant strains through compensatory mutations, underscoring the need to strengthen global surveillance and the urgency of new antiviral strategies to different strains of Influenza viruses [55,56]. A recent study of H5N1 viruses found a reassortant virus derived from a lineage of low pathogenicity avian Influenza virus, circulating in poultry with NA:275H, a marker of resistance to the oseltamivir. It is well known that antiviral resistance mutations favor high fatality cases and may necessitate a reevaluation of Influenza treatment strategies [57].

Finally, in general terms, we found some interesting facts related to Influenza virus mutational studies: (1) limited global database to keep up to date mutational and glycosylation profiles, in both the ancient Influenza viruses as the emerging/reemerging subtypes (not only H5N1). The bioinformatics servers upload all the information available, but are still required to have more analytical tools and data, for example, about the highly variable influenza accessory proteins in different numbering; (2) Influenza mutations have different nomenclature (numbering H1, H3, H5…), which makes it difficult to follow-up: for instance the mutation HA:S149A (H5 numbering) or S137A in (H3 numbering) [58,59], assigned warning level 3, (the most significant) [8], has been detected only by one of the tools used (in any numbering). So, it is a classic example of how mutational information of Influenza viruses does not have a consensus at all; (3) not all bioinformatics tools offer the option of selecting the “specific” virus variant with which you want to compare a mutation, and it is essential. The results differ greatly, for example, if you compare with a different variant, a vaccine candidate, a reference genome or one of the most recent H5N1 isolates now circulating. For instance, the mutation NA:329S has been recently related with changes in glycosylation patterns in viruses H3N2 [45], and there may be uncertainty, if it is similar to viruses of different numbering such as H5N1, H7N7, H1N1, H9N2; (4) a lack of data to reference genome (A/goose/Guandong/1996(H5N1) of accessory proteins PA-X and PB1-F2. As has been shown in the findings, there are many key mutations in these two proteins, which warrant further research.

## 5. Conclusions

Our study identified several key mutations between the 55 highly pathogenic avian Influenza A viruses (HPAIV) H5N1 isolates in outbreaks that occurred in 2022 and 2024, in the Americas region at a multi segment level such as HA:211I, NA:10T, NP:377N, PA:322V/L, PB2:539V, PB1:207R, PA-X:250Q, and PB1-F2:42Y, among others for which there is still little evidence of their possible biological function; it emphasizes the importance of carrying out meticulous surveillance in humans and animals to track crucial mutations. Additionally, the most relevant finding was the identification of a patron of cognate mutations: PA:13V, PA-X:13V, PA20T, PA-X:20T, PA:36T, PA-X:36T, PA:45S, PA-X:45S, PA:57Q, PA-X:57Q, PA:68S, PA-X:68S, PA:70V, PA-X:70V, PA:75Q, PA-X:75Q, PA:85T, PA-X:85T, PA:86I, PA-X:86I, PA:100I, PA-X:100I, PA:61I, PA-X:61I, PA:142E, PA-X:142E, PA:160E, PA-X:160E, PA:211I, PA-X:211Y, which highlight the role of the PA protein in the evolution of Influenza viruses through the activation of polymerase activity in the mammalian host, even in the absence of recognized host adaptive mutations in other polymerases such as PB2 [39]. In addition, the scanning of glycosylation profiles of H5N1 viruses highlights the loss/acquisition of NLG active sites 209NNTN, 100 NPTT, 302NSSM in HA protein and 70NNTN, 68NISS, 50NGSV in NA, providing valuable insights into viral evolution, notwithstanding, the limitations of the own study, and that the knowledge in this subject is still limited. The emergence of extra mutations and new glycosylation forms detected in H5N1 viruses underscore the necessity for further analysis and fosters science-based consensus to enhance our understanding of the evolution of these changeable viruses. Thus, reinforcing public databases is recommended, as is updating the H5N1 virus mutational profiles and including NLGs relevant sites.

## Figures and Tables

**Table 1:** Unique mutations (only detected in 1–3 samples) are considered of interest in H5N1 viruses identified per each protein segment.

Mutation	Mutation to Highlighting
HA:9V, HA:87T, HA:99S/D, HA:225M, HA:248L, HA:259C, HA:277Y, HA:285E, HA:316E, HA:324T, HA:473K, HA:493K, HA:531L	HA:10T, HA:170D, HA:104G, HA:147M, HA:152S, HA:226T, HA:304N, HA:310V, HA:336N/R, HA:520R/N
NA:20A/I, NA:216V, NA:217R, NA:223T, NA:284N, NA:308R/K, NA:340Y/F, NA:364N, NA:442I	NA:23V, NA:44N, NA:45H, NA:48T, NA:53V, NA:62I NA:67I, NA:74L, NA:75I, NA:81D; NA:82P, NA:84A, NA:90P, NA:155H, NA:221S, NA:234I, NA:237F, NA:241I, NA:254R, NA:257R, NA:286S, NA:288V, NA:329S, NA:374V, NA:399L, NA:432R, NA:436V
M1:55M, M1:125T, M1:191H, M1:218A, M1:236K	
M2:12R, M2:21G, M2:28T, M2:52S	M2:27A
PA:42V, PA:59K/G, PA:118U, PA:184S, PA:190F, PA:207V, PA:213K, PA:272N, PA:269K, PA:323I, PA:330V, PA:382G, PA:423T, PA:425F, PA:523L, PA:561V, PA:581I, PA:621V, PA:664R, PA:688G	PA:13V, PA:36T, PA:45S, PA:68S, PA:75Q, PA:86I, PA:100I, PA:142E, PA:201I/T, PA:211I, PA:322L/V, PA:336M, PA:348L, PA:351G, PA:354F, PA:388G, PA:399V, PA:404S, PA:459V, PA:465M/T, PA:486M/L, PA:489S, PA:538G, PA:545V, PA:614D/S, PA:626R, PA:655F
PA-X:42V, PA-X:52D, PA-X:62T, PA-X:118V, PA-X:122I, PA-X:184N, PA-X:190F, PA-X:207L,	PA-X:20T, PA-X:36D/T, PA-X:68S, PA-X:70V, PA-X:75Q, PA-X:86I, PA-X:142E, PA-X:160E, PA-X:195K, PA-X:211Y, PA-X:250P,
PB2:191G, PB2:199T, PB2:292V, PB2:339R, PB2:444G, PB2:451V, PB2:452V, PB2:453S, PB2:472D, PB2:560M, PB2:575V, PB2:639S, PB2:660R, PB2:679S, PB2:683A, PB2:684S, PB2:697M, PB2:711S	PB2:9N, PB2:79G, PB2:152V, PB2:190R, PB2:251K, PB2:255A, PB2:274V, PB2:346A, PB2:353R, PB2:532L, PB2:539V, PB2:596A, PB2:663R, PB2:666I, PB2:667I, PB2:670R, PB2:677K, PB2:680G, PB2:715S
PB1:11R, PB1:14V, PB1:51E, PB1:53E, PB1:121N, PB1:147V, PB1:176T, PB1:321I, PB1:339V, PB1:348V, PB1:371D, PB1:383G, PB1:455D, PB1:394S, PB1:431H, PB1:512L, PB1:576M, PB1:584H, PB1:657H, PB1:719I, PB1:739D	PB1:40I, PB1:171A, PB1:211K, PB1:291A, PB1:372I, PB1:384P/T/A, PB1:390G, PB1:533S, PB1:621K, PB1:660I, PB1:738G
NS1:66D, NS1:81V, NS1:88H, NS1:129T, NSI:210R, NSI:202T, NS1:217T, NSI:213L, NSI:219E	NSI:36I, NS1:67G/Q, NS1:75G, NS1:76A, NS1:77R, NS1:136M, NS1:193Q, NSI:201Y
NEP:27G, NEP:52V, NEP:56Y, NEP:61K, NEP:64T, NSI:76M, NEP:77K, NEP:85Q NEP:81G, NEP:82E	NEP:36V, NEP:60N, NEP:63E, NEP:89T/V
PB1-F2:11R/L, PB1-F2:29R, PB1-F2:35L, PB1-F2:41L, PB1-F2:69L, PB1-F2:78R, PB1-F2:79Q, PB1-F2:90I	PB1-F2:39T, PB1-F2:57Y, PB1-F2:73E
NP:41V, NP:190A, NP:221K, NP:234S, NP:253V, NP:323S, NP:363I	NP:48R, NP:63T, NP:119T/V, NP:230L, NP:318L, NP:411A, NP:425V

The complete list of mutations per each segment with details of the host can be seen in Supplementary S1. Mutations highlighted in blue were observed in both bird and mammal species or by the host which was found.

**Table 2:** Mutations identified in H5N1 viruses per samples group (4 or more isolates).

Mutation	Mutation to Highlighting
HA:242I, HA:504Y	HA:11I, HA:52A, HA:211I, HA:492D, HA:527I
NA:71S, NA:321I	NA:6R, NA:10T, NA:70N, NA:405T
M1:82S, M1:227T	
M2:88N/D	
NP:52H, NP:105M, NP:293K, NP:482N	NP:377N
PB2:154F, PB2:362G, PB2:441N, PB2:495I, PB2:631L, PB2:649I, PB2:676A	PB2:58A, PB2:109I, PB2:139I,
PB1:179I, PB1:587P, PB1:646I	PB1:16D, PB1:154S, PB1:172D, PB1:207R, PB1:215K, PB1:264D, PB1:375N, PB1:378M, PB1:399D, PB1:429R, PB1:430K/E, PB1:515A, PB1:548F, PB1:614D, PB1:694S
PB1-F2:30L, PB1-F2:31E, PB1-F2:44R, PB1-F2:46T, PB1-F2:47S, PB1-F2:48R, PB1-F2:50G, PB1-F2:54K, PB1-F2:55I, PB1-F2:66S, PB1-F2:68I, PB1-F2:70G	PB1-F2:4G, PB1-F2:7I/T/M, PB1-F2:8Q, PB1-F2:12S PB1-F2:17S, PB1-F2:18T, PB1-F2:20R, PB1-F2:21R, PB1-F2:22E, PB1:36T, PB1-F2:40G, PB1-F2:42Y, PB1-F2:49A, PB1-F2:56A, PB1-F2:57C/F, PB1-F2:58W, PB1-F2:65R, PB1-F2:75L, PB1-F2:82S, PB1-F2:84S, PB1-F2:90N
NS1:7S/L, NS1:21Q, NS1:88C, NS1:189N	NSI:21R, NS1:26K, NSI:53G, NS1:83P, NS1:87S, NS1:116C/S/N, NS1:147I, NS1:226T
NEP:7V/S, NEP:31V, NEP:67G/E	
PA:113R, PA:237K/A, PA:272E, PA:277P, PA:479E, PA:558L	PA:57R, PA:219I, PA:432I, PA:497R,
PA-X:113R, PA-X:215L,	PA-X:57Q, PA-X:61I, PA-X:85T, PA-X:193S, PA-X:245N

Mutations highlighted in blue were observed in both bird and mammal species or by the host which was found.

**Table 3:** Mutations detected in all H5N1 viruses analyzed.

Protein	Mutation
HA	110S, 139P, 142E, 143T, 154Q, 157P, 171D, 208K 234Q, 239R
NA	46P, 76A, 78Q, 99I, 100Y, 258I, 289M, 366S, 382E, 418M, 434N
M1	140A, 144L, 165I,
M2	18N
NP	136L
PB2	699K, 741S
PB1	177E, 478S, 490F, 535I, 536N, 558T, 598P, 609Y, 610C
PB1-F2	No PB1-F2 sequence in the reference genome
NS1	6I, 18V, 22F, 23S, 24D, 25Q, 27L, 28C, 54I, 60A, 73S, 84V, 94T, 95L, 112A, 114G, 117I, 127R, 137L, 140Q, 146L, 153E, 158G, 161S, 163L, 170T, 180V, 191T, 194V, 197T, 198L, 205S, 206S, 211R, 221K, 224R, 225T
NEP (NS2)	6V, 14M, 22G, 26E, 37S, 40L, 48A, 49V, 68Q, 83V, 86R, 88K, 100M, 111Q
PA	63V, 129I, 212C, 228N, 361K, 536K, 544E, 585L, 586L, 716R
PA-X	no PA-X sequence in the reference genome
PB2	355R, 699K

The comparisons were performed with the reference genome A/goose/Guandong/1996(H5N1), which no have sequences available to the accessory proteins PB1-F2 and PA-X. Mutations highlighted in blue were observed in both bird and mammal species or by the host which was found.

**Table 4:** Mutations found in almost all H5N1 viruses with some exceptions.

Protein	Mutation
PB1	59S, 75D
PB2	334S, 340R, 463V, 464M, 471T, 478I, 590G, 616V
NP	450N
M2	28I, 51V, 61G
M1	85S, 87T, 101R, 200V, 230R, 232D
HA	120M, 131L, 199N, 201E, 205N, 226A/T, 336S/N, 527V/I, 549M
NA	8T, 20V/A, 44Y/N, 81T/D, 155Y, 188I, 269M, 287D, 340S, 336S, 338M, 339P, 340S, 395E, 460G
NEP	63A, 64K, 81E, 85H, 89I
PA-x	252R/K
PB1-F2	11Q, 12L/S
NS1	44R, 55E, 56T, 59R, 63Q, 70E/G, 71E, 74D, 90L, 111V/A, 118R, 139D, 145I, 166L, 171D, 192V, 204R, 207N, 209D, 213P, 226T

Mutations highlighted in blue were observed in both bird and mammal species or by the host which was found.

## Data Availability

Data are contained within the article and Supplementary Material.

## Supplementary Materials

The following supporting information can be downloaded at: https://www.mdpi.com/article/10.3390/vetsci12040392/s1, S1: Listing of data sets/mutations found: S1.Table S1. Listing of H5N1 Influenza A viruses data set (NCBI) used in the study; S1.Table S2. Listing of mutations found in H5N1 Influenza viruses and its characteristics; S1.Table S3. Unique mutations (only detected in 1–3 samples) in H5N1 viruses identified per each protein segment; S1. Table S4: Mutations identified in H5N1 viruses per samples group (4 or more isolates); S1.Table S5. Mutations detected in all H5N1 viruses analyzed; S1.Table S6. Mutations found in almost all H5N1 viruses with some exceptions. S2: Complete mutational data analysis; S3: FluMut data analysis; S4: Genoflu data analysis S5: Listing of N-Linked Glycosylations (NLGs) profiles.
