# Supplementary material for: Comparative Mutational Analysis and the Glycosylation Patterns of a Peruvian Isolated Avian Influenza A Virus H5N1: Exploring Possible Viral Spillover Events Within One Health Approach
Source: Vet Sci. 2025 Apr 21;12(4):392. doi: 10.3390/vetsci12040392 (PMC12031540; doi:10.3390/vetsci12040392)

DATOS GLICOSILACION

Asn-Xaa-Ser/Thr sequons in the sequence output below are highlighted in blue.
Asparagines predicted to be N-glycosylated are highlighted in red.

1. >XLD56333.1 hemagglutinin [Influenza A virus/California/181/2024]

```
Name:  XLD56333.1      Length:  567
MENIVLLLAIVSLVKSDQICIGYHANNSTEQVDTIMEKNVTVTHAQDILEKTHNGKLCDLNGVKPLILKDCSVAGWLLGN      80
PMCDEFIRVPEWSYIVERANPANGLCYPGSLNDYEELKHMLSRINHFEKIQIIPKSSWPNHETSLGMSAACPYQGAPSFF     160
RNVVWLIKKNDAYPTIKISYNNTNREDLLILWGIHHSNNAEEQTNLYKNPITYISVGTSTLNQRLAPKIATRSQVNGQRG     240
RMDFFWTILKPDDAIHFESNGNFIAPEYAYKIVKKGDSTIMKSGVEYGHCNTKCQTPVGAINSSMPFHNIHPLTIGECPK     320
YVKSNKLVLATGLRNNPLREKRRKRGLFGAIAGFIEGGWQGMVDGWYGYHHSNEQGSGYAADKESTQKAIDGVTNKVNSI     400
IDKMNTQFEAVGREFNNLERRIENLNKKMEDGFLDVWTYNAELLVLMENERTLDFHDSNVKNLYDKVRLQLRDNAKELGN     480
GCFEFYHKCDNECMESVRNGTYDYPQYSEEARLKREEISGVKLESVGTYQILSIYSTAASSLALAIMMAGLSLWMCSNGS     560
LQCRICI                                                                              640
..........................N...........N.........................................      80
................................................................................     160
....................N...........................................................     240
.............................................................N..................     320
................................................................................     400
................................................................................     480
..................N..........................................................N..     560
.......                                                                              640
```

(Threshold=0.5)

SeqName	Position		Potential	Jury agreement	N-Glyc result
XLD56333.1	26	NNST	0.3687	(9/9)	--
XLD56333.1	27	NSTE	0.7796	(9/9)	+++
XLD56333.1	39	NVTV	0.7183	(9/9)	++
XLD56333.1	181	NNTN	0.6100	(7/9)	+
XLD56333.1	302	NSSM	0.5447	(6/9)	+
XLD56333.1	499	NGTY	0.5826	(6/9)	+
XLD56333.1	558	NGSL	0.6830	(9/9)	++

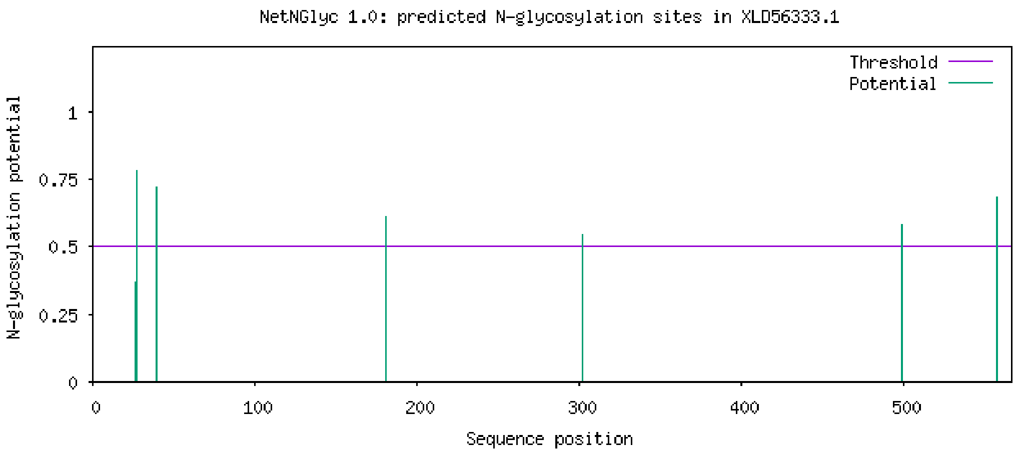

>XLD56341.1 neuraminidase [Influenza A virus/California/181/2024]

```
Name:  XLD56341.1        Length:  469
MNPNQKITTIGSICMVIGIVSLMLQIGNIISIWVSHSIQTGNQYQPEPCNQSIITYENNTWVNQTYINISSTNFLAEQAV      80
TSVTLAGNSSLCPISGWAIYSKDNGIRIGSKGDVFVIREPFISCSHLECRTFFLTQGALLNDKHSNGTVKDRSPYRTLMS     160
CPVGEAPSPYNSRFESVAWSASACHDGISWLTIGISGPDNGAVAVLKYNGIITDTIKSWRNNILRTQESECACVNGSCFT     240
VMTDGPSNGQASYKIFKIEKGKVVKSVEMNAPNYHYEECSCYPDAGDIMCVCRDNWHGSNRPWVSFNQNLEYQIGYICSG     320
IFGDNPRPNDGTGSCSPMPSNGAYGVKGFSFKYGNGVWIGRTKSTSSRSGFEMIWDPNGWTETDSSFSVKQDIVEITDWS     400
GYSGSFVQHPELTGLDCMRPCFWVELIRGRPKENTIWTSGSSISFCGVNSDTVGWSWPDGAELPFTIDK                480
.................................................N.......N....N....N............      80
.......N.........................................................N..............     160
..........................................................................N.....     240
................................................................................     320
................................................................................     400
.....................................................................                480
```

(Threshold=0.5)

SeqName	Position	Potential	Jury agreement	N-Glyc result
XLD56341.1	50 NQSI	0.5883	(8/9)	+
XLD56341.1	58 NNTW	0.5251	(5/9)	+
XLD56341.1	63 NQTY	0.6874	(9/9)	++
XLD56341.1	68 NISS	0.7140	(9/9)	++
XLD56341.1	88 NSSL	0.7724	(9/9)	+++
XLD56341.1	146 NGTV	0.6873	(9/9)	++
XLD56341.1	235 NGSC	0.7321	(9/9)	++

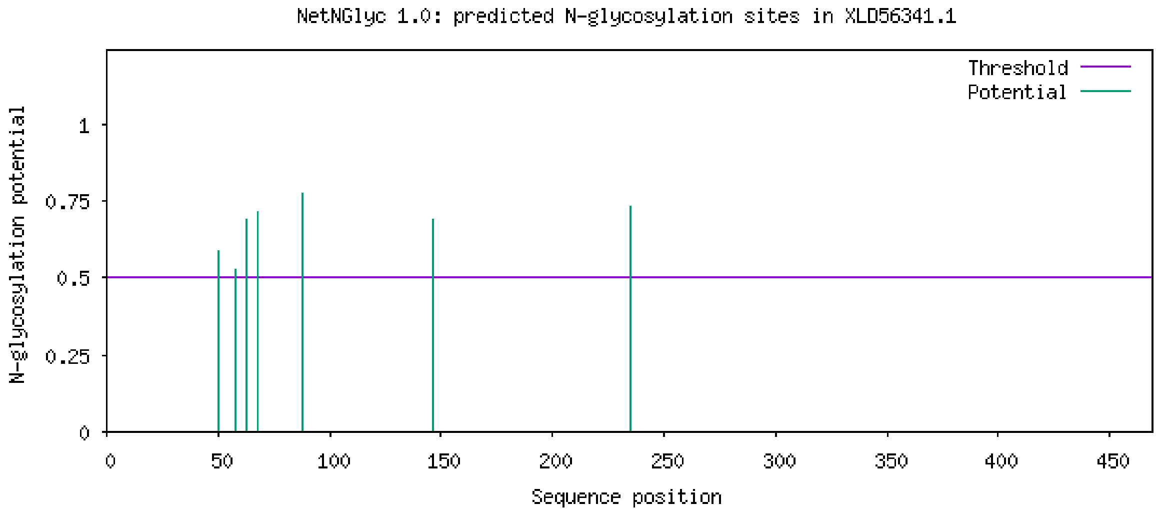

2. >XCO67366.1 hemagglutinin [Influenza A virus/alpaca/Idaho/2024]

```
Name:  XCO67366.1       Length:  567
MENIVLLLAIVSLVKSDQICIGYHANNSTEQVDTIMEKNVTVTHAQDILEKTHNGKLCDLNGVKPLILKDCSVAGWLLGN      80
PMCDEFIRVPEWSYIVERANPANDLCYPGSLNDYEELKHMLSRINHFEKIQIIPKSSWPNHETSLGVSAACPYQGAPSFF     160
RNVVWLIKKNDAYPTIKISYNNTNREDLLILWGIHHSNNAEEQTNLYKNPITYISVGTSTLNQRLAPKIATRSQVNGQRG     240
RMDFFWTILKPDDAIHFESNGNFIAPEYAYKIVKKGDSTIMKSGVEYGHCNTKCQTPVGAINSSMPFHNIHPLTIGECPK     320
YVKSNKLVLATGLRNSPLREKRRKRGLFGAIAGFIEGGWQGMVDGWYGYHHSNEQGSGYAADKESTQKAIDGVTNKVNSI     400
IDKMNTQFEAVGREFNNLERRIENLNKKMEDGFLDVWTYNAELLVLMENERTLDFHDSNVKNLYDKVRLQLRDNAKELGN     480
GCFEFYHKCDNECMESVRNGTYDYPQYSEEARLKREEISGVKLESVGTYQILSIYSTAASSLALAIMMAGLSLWMCSNGS     560
LQCRICI                                                                              640
..........................N...........N.........................................      80
................................................................................     160
....................N...........................................................     240
.............................................................N..................     320
................................................................................     400
................................................................................     480
..................N..........................................................N..     560
.......                                                                              640

(Threshold=0.5)
```

SeqName	Position	Potential	Jury agreement	N-Glyc result
XCO67366.1	26 NNST	0.3686	(9/9)	--
XCO67366.1	27 NSTE	0.7796	(9/9)	+++
XCO67366.1	39 NVTV	0.7182	(9/9)	++
XCO67366.1	181 NNTN	0.6100	(7/9)	+
XCO67366.1	302 NSSM	0.5447	(6/9)	+
XCO67366.1	499 NGTY	0.5826	(6/9)	+
XCO67366.1	558 NGSL	0.6827	(9/9)	++

NetNGlyc 1.0: predicted N-glycosylation sites in XCO67366.1

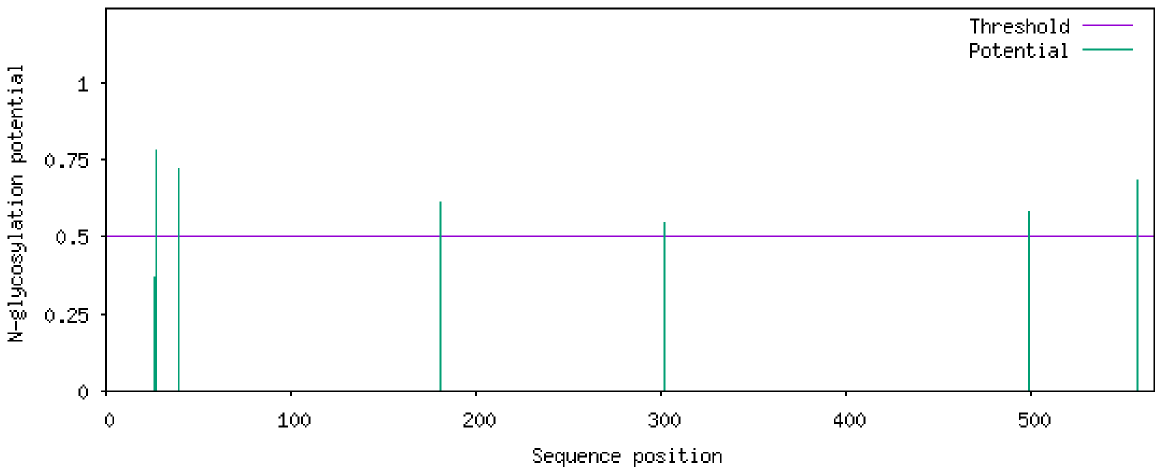

>XCO67368.1 neuraminidase [Influenza A virus/alpaca/Idaho/2024]

```
Name:  XCO67368.1       Length:  469
MNPNQKITTIGSICMVIGIVSLMLQIGNIISIWVSHSIQTGNQYQPEPCNQSIITYENNTWVNQTYINISSTNFLAEQAV      80
TSVTLAGNSSLCPISGWAIYSKDNGIRIGSKGDVFVIREPFISCSHLECRTFFLTQGALLNDKHSNGTVKDRSPYRTLMS     160
CPVGEAPSPYNSRFESVAWSASACHDGISWLTIGISGPDNGAVAVLKYNGIITDTIKSWRNNILRTQESECACVNGSCFT     240
VMTDGPSNGQASYKIFKIEKGKVVKSVEMNAPNYHYEECSCYPDAGDIMCVCRDNWHGSNRPWVSFNQNLEYQIGYICSG     320
IFGDNPRPNDGTGSCSPMPSNGAYGVKGFSFKYGNGVWIGRTKSTSSRSGFEMIWDPNGWTETDSSFSVKQDIVEITDWS     400
GYSGSFVQHPELTGLDCMRPCFWVELIRGRPKENTIWTSGSSISFCGVNSDTVGWSWPDGAELPFTIDK                480
.................................................N.......N....N....N............      80
.......N.........................................................N..............     160
..........................................................................N.....     240
................................................................................     320
................................................................................     400
.....................................................................                480
```

(Threshold=0.5)

```
----------------------------------------------------------------------
SeqName      Position  Potential   Jury     N-Glyc
                                 agreement  result
----------------------------------------------------------------------
XCO67368.1    50 NQSI   0.5883     (8/9)    +
XCO67368.1    58 NNTW   0.5251     (5/9)    +
XCO67368.1    63 NQTY   0.6874     (9/9)    ++
XCO67368.1    68 NISS   0.7140     (9/9)    ++
XCO67368.1    88 NSSL   0.7724     (9/9)    +++
XCO67368.1   146 NGTV   0.6873     (9/9)    ++
XCO67368.1   235 NGSC   0.7321     (9/9)    ++
----------------------------------------------------------------------
```

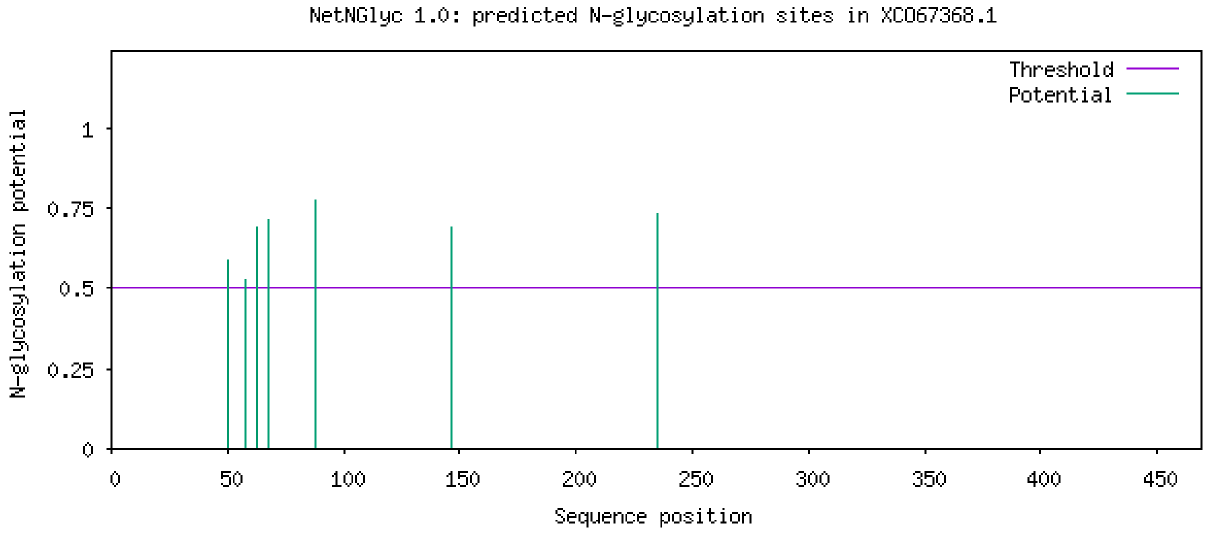

3. >XCO01697.1 hemagglutinin [Influenza A virus/Southern elephant seal/Peninsula Valdes/HA_CH-PD032]

```
Name:  XCO01697.1       Length:  567
MENIVLLLAIVSLVKSDQICIGYHANNSTEQVDTIMEKNVTVTHAQDILEKTHNGKLCDLNGVKPLILKDCSVAGWLLGN      80
PMCDEFIRVPEWSYIVERANPANDLCYPGSLNDYEELKHMLSRINHFEKIQIIPKSSWPNHETSLGVSAACPYQGAPSFF     160
RNVVWLIKKNDAYPTIKISYNNTNREDLLILWGIHHSNNAEEQTNLYKNPTTYISVGTSTLNQRLAPKIATRSQVNGQRG     240
RMDFFWTILKPDDAIHFESNGNFIAPEYAYKIVKKGDSTIMKSGVEYGHCNTKCQTPVGAINSSMPFHNIHPLTIGECPK     320
YVKSNKLVLATGLRNSPLREKRRKRGLFGAIAGFIEGGWQGMVDGWYGYHHSNEQGSGYAADKESTQKAIDGVTNKVNSI     400
IDKMNTQFEAVGREFNNLERRIENLNKKMEDGFLDVWTYNAELLVLMENERTLDFHDSNVKNLYDKVRLQLRDNAKELGN     480
GCFEFYHKCDNECMESVRNGTYDYPQYSEEARLKREEISGVKLESVGTYQILSIYSTAASSLALAIMMAGLSLWMCSNGS     560
LQCRICI                                                                              640
..........................N...........N.........................................      80
................................................................................     160
....................N...........................N...............................     240
.............................................................N..................     320
................................................................................     400
................................................................................     480
..................N..........................................................N..     560
.......                                                                              640
```

(Threshold=0.5)

SeqName	Position	Potential	Jury agreement	N-Glyc result	
XCO01697.1	26 NNST	0.3686	(9/9)	--	
XCO01697.1	27 NSTE	0.7797	(9/9)	+++	
XCO01697.1	39 NVTV	0.7182	(9/9)	++	
XCO01697.1	181 NNTN	0.6100	(7/9)	+	
XCO01697.1	209 NPTT	0.6336	(8/9)	+	WARNING: PRO-X1.
XCO01697.1	302 NSSM	0.5447	(6/9)	+	
XCO01697.1	499 NGTY	0.5824	(6/9)	+	
XCO01697.1	558 NGSL	0.6827	(9/9)	++	

NetNGlyc 1.0: predicted N-glycosylation sites in XCO01697.1

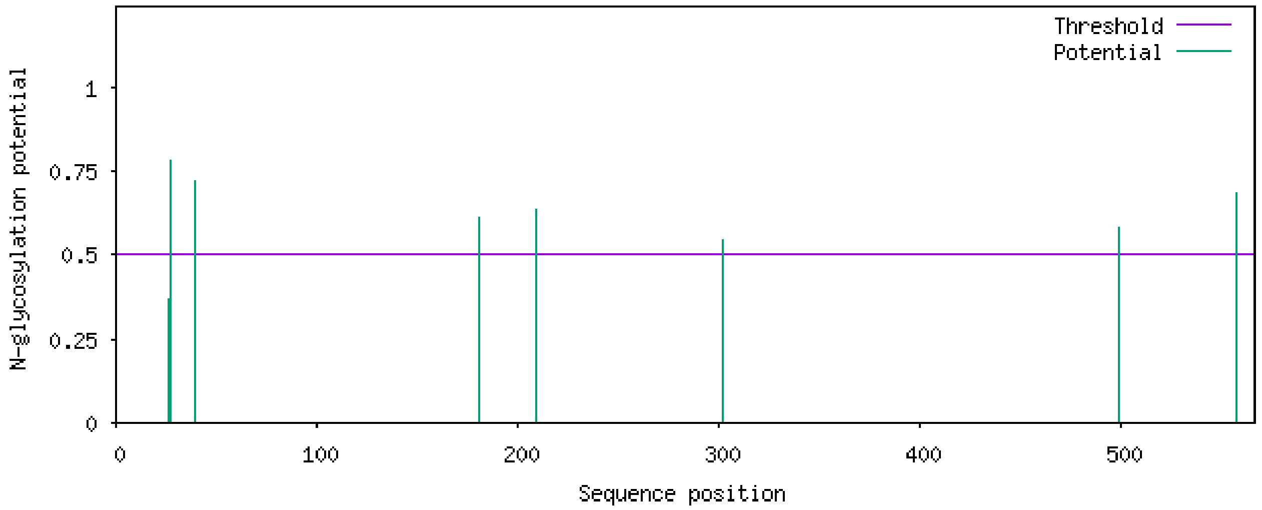

>XCO01699.1 neuraminidase [Influenza A virus/Southern-elephant-seal/Peninsula Valdes/NA_CH-PD032/2023]

```
Name:  XCO01699.1        Length:  469
MNPNQKITTIGSICMVIGIVSLMLQIGNIISIWVSHSIQTGNQYQPEPCNQSIITYENNTWVNQTYVNISNTNFLAEQAV      80
TSVTLAGNSSLCPISGWAIYSKDNGIRIGSKGDVFVIREPFISCSHLECRTFFLTQGALLDDKHSNGTVKDRSPYRTLMS     160
CPVGEAPSPYNSRFESVAWSASACHDGISWLTIGISGPDNGAVAVLKYNGIITDTIKSWRNNILRTQESECACVNGSCFT     240
VMTDGPSNGQASYKIFKIEKGKVVKSVEMNAPNYHYEECSCYPDAGDIMCVCRDNWHGSNRPWVSFNQNLEYQIGYICSG     320
VFGDNPRPNDGTGSCSPMPSNGAYGVKGFSFKYGNGVWIGRTKSTSSRSGFEMIWDPNGWTETDSSFSVKQDIVEITDWS     400
GYSGSFVQHPELTGLDCMRPCFWVELIRGRPKENTIWTSGSSISFCGVNSDTVGWSWPDGAELPFTIDK                480
.................................................N.......N....N....N............      80
.......N.........................................................N..............     160
..........................................................................N.....     240
................................................................................     320
................................................................................     400
.....................................................................                480

(Threshold=0.5)
----------------------------------------------------------------------
SeqName      Position  Potential   Jury    N-Glyc
                                 agreement result
----------------------------------------------------------------------
XCO01699.1    50 NQSI   0.5886     (8/9)   +
XCO01699.1    58 NNTW   0.5496     (6/9)   +
XCO01699.1    63 NQTY   0.6634     (9/9)   ++
XCO01699.1    68 NISN   0.7378     (9/9)   ++
XCO01699.1    88 NSSL   0.7724     (9/9)   +++
XCO01699.1   146 NGTV   0.7042     (9/9)   ++
XCO01699.1   235 NGSC   0.7321     (9/9)   ++
----------------------------------------------------------------------
```

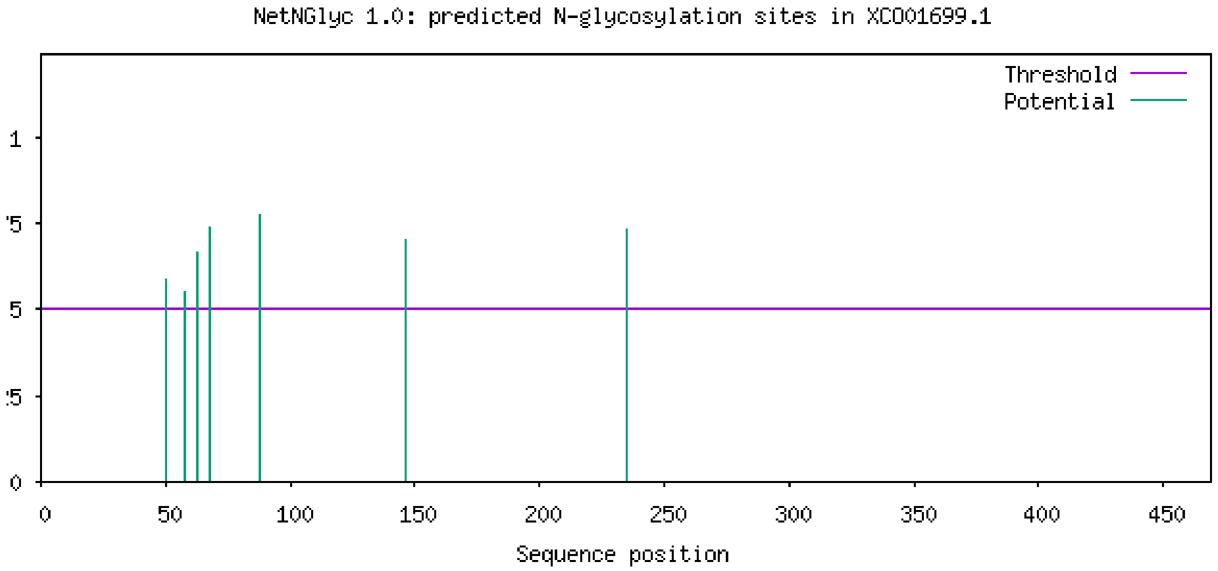

4. >WXB47604.1 hemagglutinin [Influenza A virus/South-American-tern/Argentina/CH-PD030 /2023]

```
Name:  WXB47604.1       Length:  567
MENIVLLLAIVSLVKSDQICIGYHANNSTEQVDTIMEKNVTVTHAQDILEKTHNGKLCDLNGVKPLILKDCSVAGWLLGN      80
PMCDEFIRVPEWSYIVERANPANDLCYPGSLNDYEELKHMLSRINHFEKIQIIPKSSWPNHETSLGVSAACPYQGAPSFF     160
RNVVWLIKKNDAYPTIKISYNNTNREDLLILWGIHHSNNAEEQTNLYKNPTTYISVGTSTLNQRLAPKIATRSQVNGQRG     240
RMDFFWTILKPDDAIHFESNGNFIAPEYAYKIVKKGDSTIMKSGVEYGHCNTKCQTPVGAINSSMPFHNIHPLTIGECPK     320
YVKSNKLVLATGLRNSPLREKRRKRGLFGAIAGFIEGGWQGMVDGWYGYHHSNEQGSGYAADKESTQKAIDGVTNKVNSI     400
IDKMNTQFEAVGREFNNLERRIENLNKKMEDGFLDVWTYNAELLVLMENERTLDFHDSNVKNLYDKVRLQLRDNAKELGN     480
GCFEFYHKCDNECMESVRNGTYDYPQYSEEARLKREEISGVKLESVGTYQILSIYSTAASSLALAIMMAGLSLWMCSNGS     560
LQCRICI                                                                              640
..........................N...........N.........................................      80
................................................................................     160
....................N...........................N...............................     240
.............................................................N..................     320
................................................................................     400
................................................................................     480
..................N..........................................................N..     560
.......                                                                              640

(Threshold=0.5)
----------------------------------------------------------------------
SeqName      Position  Potential   Jury    N-Glyc
                                 agreement result
----------------------------------------------------------------------
WXB47604.1     26 NNST   0.3686    (9/9)   --
WXB47604.1     27 NSTE   0.7797    (9/9)   +++
WXB47604.1     39 NVTV   0.7182    (9/9)   ++
WXB47604.1    181 NNTN   0.6100    (7/9)   +
WXB47604.1    209 NPTT   0.6336    (8/9)   +     WARNING: PRO-X1.
WXB47604.1    302 NSSM   0.5447    (6/9)   +
WXB47604.1    499 NGTY   0.5824    (6/9)   +
WXB47604.1    558 NGSL   0.6827    (9/9)   ++
----------------------------------------------------------------------
```

NetNGlyc 1.0: predicted N-glycosylation sites in WXB47604.1

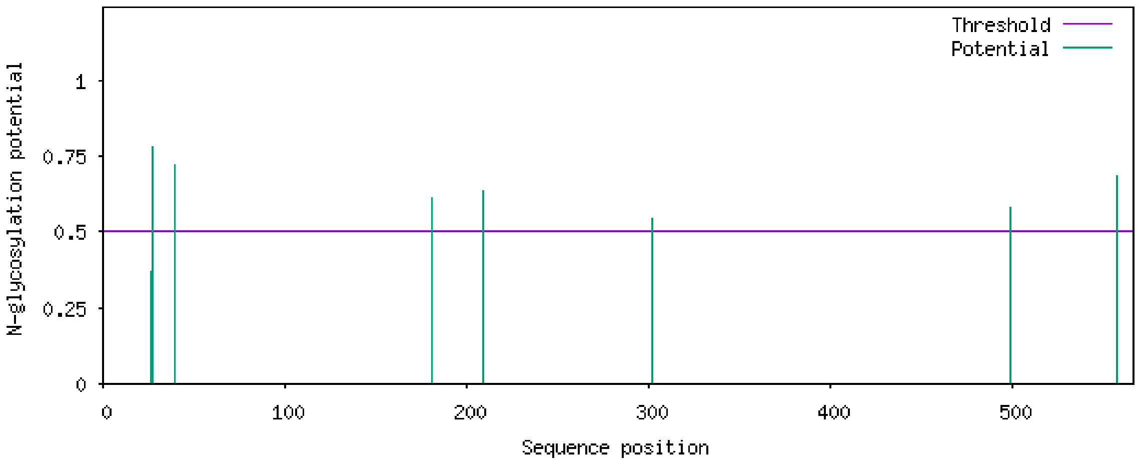

>WXB47606.1 neuraminidase [Influenza A virus/South-American-tern/Argentina/CH-PD030/2023]

```
Name:  WXB47606.1       Length:  469
MNPNQKITTIGSICMVIGIVSLMLQIGNIISIWVSHSIQTGNQYQPEPCNQSIITYENNTWVNQTYVNISNTNFLAEQAV      80
TSVTLAGNSSLCPISGWAIYSKDNGIRIGSKGDVFVIREPFISCSHLECRTFFLTQGALLNDKHSNGTVKDRSPYRTLMS     160
CPVGEAPSPYNSRFESVAWSASACHDGISWLTIGISGPDNGAVAVLKYNGIITDTIKSWRNNILRTQESECACVNGSCFT     240
VMTDGPSNGQASYKIFKIEKGKVVKSVEMNAPNYHYEECSCYPDAGDIMCVCRDNWHGSNRPWVSFNQNLEYQIGYICSG     320
VFGDNPRPNDGTGSCSPMPSNGAYGVKGFSFKYGNGVWIGRTKSTSSRSGFEMIWDPNGWTETDSSFSVKQDIVEITDWS     400
GYSGSFVQHPELTGLDCMRPCFWVELIRGRPKENTIWTSGSSISFCGVNSDTVGWSWPDGAELPFTIDK                480
.................................................N.......N....N....N............      80
.......N.........................................................N..............     160
..........................................................................N.....     240
................................................................................     320
................................................................................     400
.....................................................................                480

(Threshold=0.5)
----------------------------------------------------------------------
SeqName      Position  Potential   Jury    N-Glyc
                                agreement  result
----------------------------------------------------------------------
WXB47606.1     50 NQSI   0.5884     (8/9)   +
WXB47606.1     58 NNTW   0.5496     (6/9)   +
WXB47606.1     63 NQTY   0.6636     (9/9)   ++
WXB47606.1     68 NISN   0.7378     (9/9)   ++
WXB47606.1     88 NSSL   0.7724     (9/9)   +++
WXB47606.1    146 NGTV   0.6876     (9/9)   ++
WXB47606.1    235 NGSC   0.7321     (9/9)   ++
----------------------------------------------------------------------
```

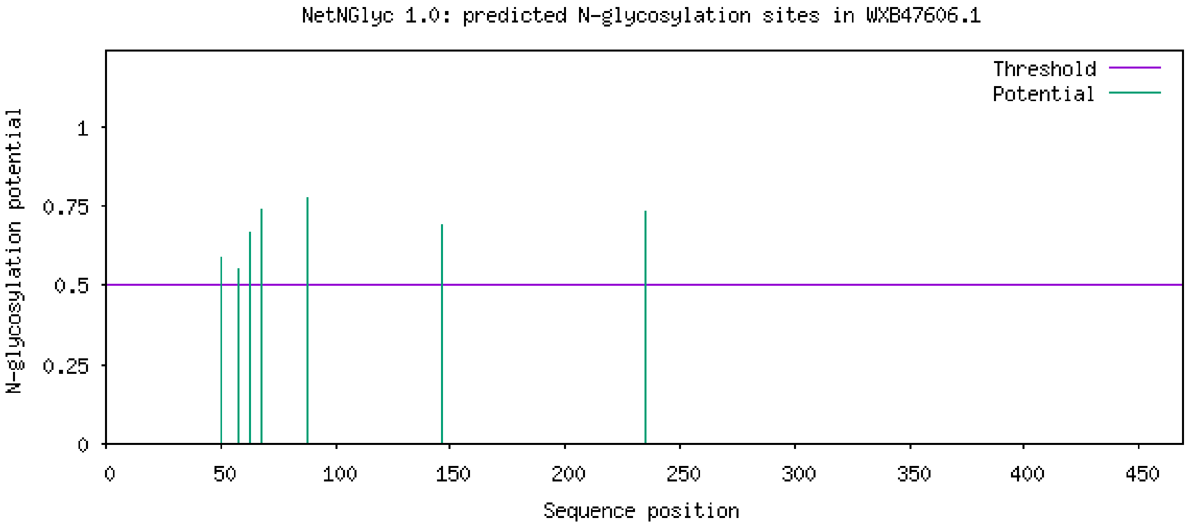

5. >WYX94321.1 hemagglutinin [Influenza A virus/bovine/Texas/2024]

```
Name:  WYX94321.1       Length:  567
MENIVLLLAIVSLVKSDQICIGYHANNSTEQVDTIMEKNVTVTHAQDILEKTHNGKLCDLNGVKPLILKDCSVAGWLLGN      80
PMCDEFIRVPEWSYIVERANPANDLCYPGSLNDYEELKHMLSRINHFEKIQIIPKSSWPNHETSLGVSAACPYQGAPSFF     160
RNVVWLIKKNDAYPTIKISYNNTNREDLLILWGIHHSNNAEEQTNLYKNPITYISVGTSTLNQRLAPKIATRSQVNGQRG     240
RMDFFWTILKPDDAIHFESNGNFIAPEYAYKIVKKGDSTIMKSGVEYGHCNTKCQTPVGAINSSMPFHNIHPLTIGECPK     320
YVKSNKLVLATGLRNSPLREKRRKRGLFGAIAGFIEGGWQGMVDGWYGYHHSNEQGSGYAADKESTQKAIDGVTNKVNSI     400
IDKMNTQFEAVGREFNNLERRIENLNKKMEDGFLDVWTYNAELLVLMENERTLDFHDSNVKNLYDKVRLQLRDNAKELGN     480
GCFEFYHKCDNECMESVRNGTYDYPQYSEEARLKREEISGVKLESVGTYQILSIYSTAASSLALAIMMAGLSLWMCSNGS     560
LQCRICI                                                                              640
..........................N...........N.........................................      80
................................................................................     160
....................N...........................................................     240
.............................................................N..................     320
................................................................................     400
................................................................................     480
..................N..........................................................N..     560
.......                                                                              640

(Threshold=0.5)
----------------------------------------------------------------------
SeqName      Position  Potential   Jury    N-Glyc
                                 agreement result
----------------------------------------------------------------------
WYX94321.1     26 NNST   0.3686     (9/9)   --
WYX94321.1     27 NSTE   0.7796     (9/9)   +++
WYX94321.1     39 NVTV   0.7182     (9/9)   ++
WYX94321.1    181 NNTN   0.6100     (7/9)   +
WYX94321.1    302 NSSM   0.5447     (6/9)   +
WYX94321.1    499 NGTY   0.5826     (6/9)   +
WYX94321.1    558 NGSL   0.6827     (9/9)   ++
----------------------------------------------------------------------
```

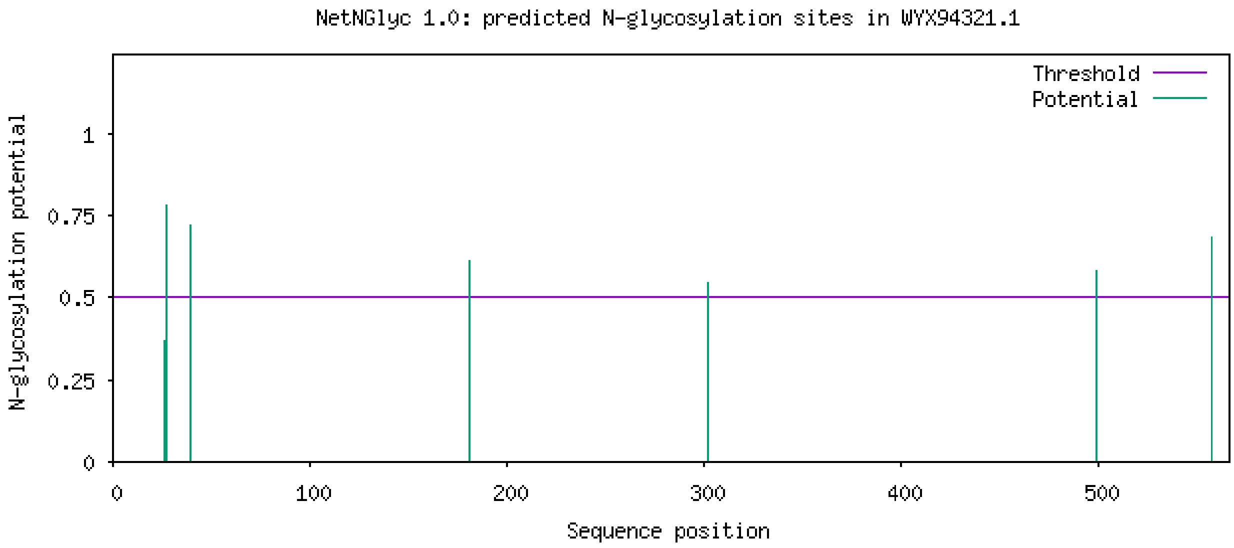

>WYX94323.1 neuraminidase [Influenza A virus/Bovine/Texas/2024]

```
Name:  WYX94323.1       Length:  469
MNPNQKITTIGSICMVIGIVSLMLQIGNIISIWVSHSIQTGNQYQPEPCNQSIITYENNTWVNQTYINISSTNFLAEQAV      80
TSVTLAGNSSLCPISGWAIYSKDNGIRIGSKGDVFVIREPFISCSHLECRTFFLTQGALLNDKHSNGTVKDRSPYRTLMS     160
CPVGEAPSPYNSRFESVAWSASACHDGISWLTIGISGPDNGAVAVLKYNGIITDTIKSWRNNILRTQESECACVNGSCFT     240
VMTDGPSNGQASYKIFKIEKGKVVKSVEMNAPNYHYEECSCYPDAGDIMCVCRDNWHGSNRPWVSFNQNLEYQIGYICSG     320
IFGDNPRPNDGTGSCSPMPSNGAYGVKGFSFKYGNGVWIGRTKSTSSRSGFEMIWDPNGWTETDSSFSVKQDIVEITDWS     400
GYSGSFVQHPELTGLDCMRPCFWVELIRGRPKENTIWTSGSSISFCGVNSDTVGWSWPDGAELPFTIDK                480
.................................................N.......N....N....N............      80
.......N.........................................................N..............     160
..........................................................................N.....     240
................................................................................     320
................................................................................     400
.....................................................................                480

(Threshold=0.5)
----------------------------------------------------------------------
SeqName      Position  Potential   Jury    N-Glyc
                                 agreement result
----------------------------------------------------------------------
WYX94323.1     50 NQSI   0.5883     (8/9)   +
WYX94323.1     58 NNTW   0.5251     (5/9)   +
WYX94323.1     63 NQTY   0.6874     (9/9)   ++
WYX94323.1     68 NISS   0.7140     (9/9)   ++
WYX94323.1     88 NSSL   0.7724     (9/9)   +++
WYX94323.1    146 NGTV   0.6873     (9/9)   ++
WYX94323.1    235 NGSC   0.7321     (9/9)   ++
----------------------------------------------------------------------
```

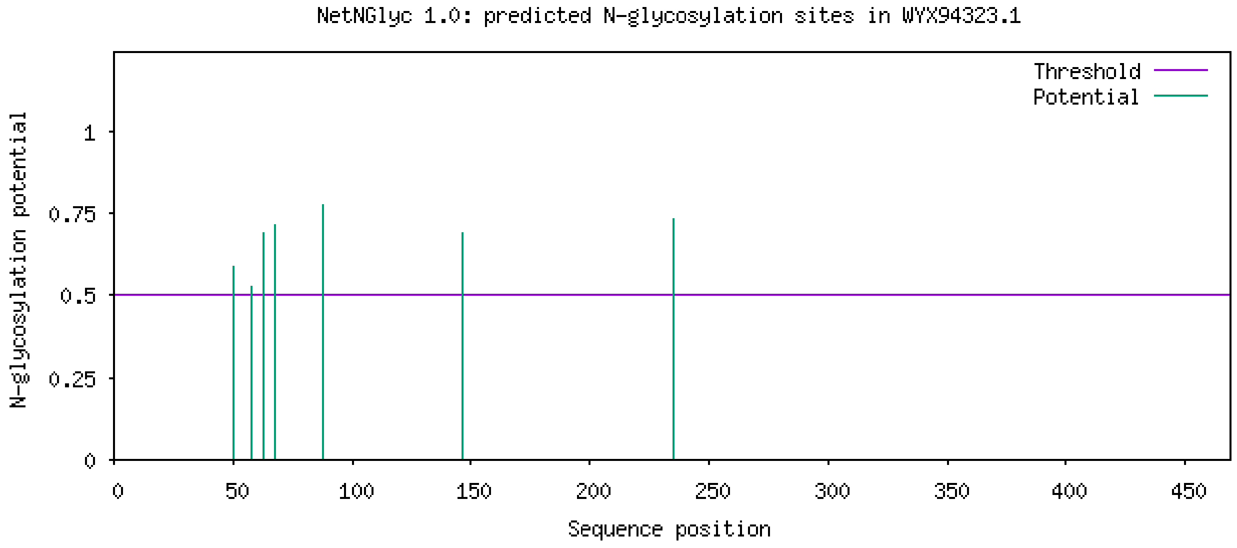

6. >WKF49009.1 hemagglutinin [Influenza A virus/Thalasseus-acuflavidus/EspiritoSanto/1339_N2/2023]

```
Name:  WKF49009.1       Length:  567
MENIVLLLAIVSLVKSDQICIGYHANNSTEQVDTIMEKNVTVTHAQDILEKTHNGKLCDLNGVKPLILKDCSVAGWLLGN      80
PMCDEFIRVPEWSYIVERANPANDLCYPGSLNDYEELKHMLSRINHFEKIQIIPKSSWPNHETSLGVSAACPYQGAPSFF     160
RNVVWLIKKNDAYPTIKISYNNTNREDLLILWGIHHSNNAEEQTNLYKNPTTYISVGTSTLNQRLAPKIATRSQVNGQRG     240
RIDFFWTILKPDDAIHFESNGNFIAPEYAYKIVKKGDSTIMKSGVEYGHCNTKCQTPVGAINSSMPFHNIHPLTIGECPK     320
YVKSNKLVLATGLRNSPLREKRKKRGLFGAIAGFIEGGWQGMVDGWYGYHHSNEQGSGYAADKESTQKAIDGVTNKVNSI     400
IDKMNTQFEAVGREFNNLERRIENLNKKMEDGFLDVWTYNAELLVLMENERTLDFHDSNVKNLYDKVRLQLRDNAKELGN     480
GCFEFYHKCDNECMESVRNGTYYYPQYSEEARLKREEISGVKLESVGTYQILSIYSTAASSLALAIMMAGLSLWMCSNGS     560
LQCRICI                                                                              640
.........................N...........N..........................................      80
................................................................................     160
....................N..........................N................................     240
.............................................................N..................     320
................................................................................     400
................................................................................     480
..................N...........................................................N..     560
.......                                                                              640

(Threshold=0.5)
----------------------------------------------------------------------
SeqName      Position  Potential   Jury    N-Glyc
                                agreement  result
----------------------------------------------------------------------
WKF49009.1    26 NNST   0.3686     (9/9)    --
WKF49009.1    27 NSTE   0.7797     (9/9)    +++
WKF49009.1    39 NVTV   0.7181     (9/9)    ++
WKF49009.1   181 NNTN   0.6100     (7/9)    +
WKF49009.1   209 NPTT   0.6336     (8/9)    +     WARNING: PRO-X1.
WKF49009.1   302 NSSM   0.5447     (6/9)    +
WKF49009.1   499 NGTY   0.5821     (5/9)    +
WKF49009.1   558 NGSL   0.6826     (9/9)    ++
----------------------------------------------------------------------
```

NetNGlyc 1.0: predicted N-glycosylation sites in WKF49009.1

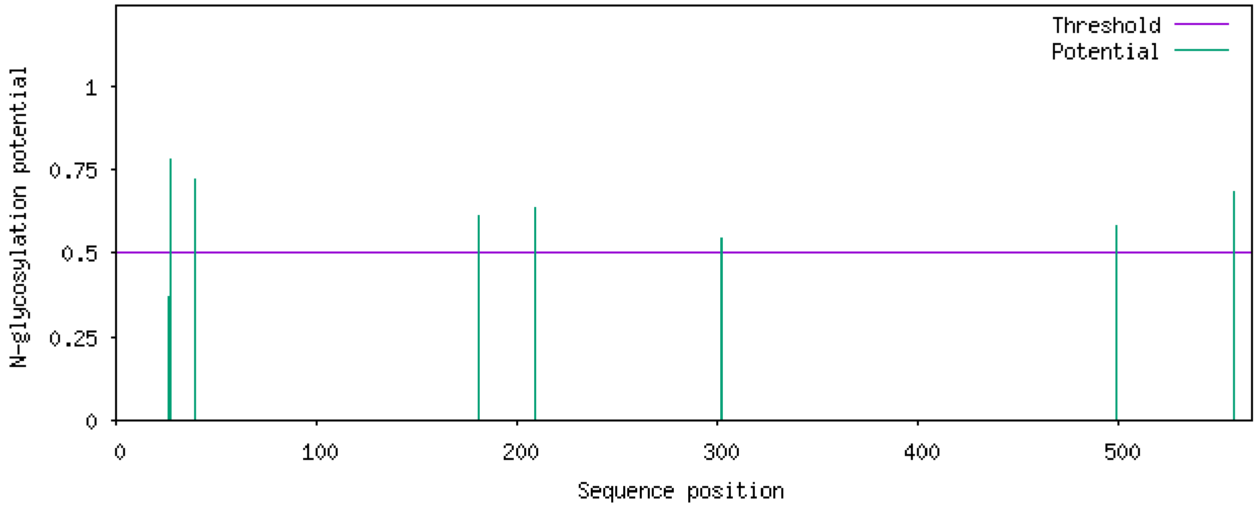

>WKF49011.1 neuraminidase [Influenza A virus/Thalaseus/Brazil]

```
Name:  WKF49011.1        Length:  469
MNPNQKITTIGSICMVIGIVSLMLQIGNIISIWVSHSIQTGNQYQPEPCNQSIITYENNTWVNQTYVNISNTNFLAEQAV      80
TSVTLAGNSSLCPISGWAIYSKDNGIRIGSKGDVFVIREPFISCSHLECRTFFLTQGALLNDKHSNGTVKDRSPYRTLMS     160
CPVGEAPSPYNSRFESVAWSASACHDGISWLTIGISGPDNGAVAVLKYNGIITDTIKSWRNNILRTQESECACVNGSCFT     240
VMTDGPSNGQASYKIFKIEKGKVVKSVEMNAPNYHYEECSCYPDAGDIMCVCRDNWHGSNRPWVSFNQNLEYQIGYICSG     320
VFGDNPRPNDGTGSCSPMPSNGAYGVKGFSFKYGNGVWIGRTKSTSSRSGFEMIWDPNGWTETDSSFSVKQDIVEITDWS     400
GYSGSFVQHPELTGLDCMRPCFWVELIRGRPKENTIWTSGSSISFCGVNSDTVGWSWPDGAELPFTIDK                480
.................................................N.......N....N....N............      80
.......N.........................................................N..............     160
..........................................................................N.....     240
................................................................................     320
................................................................................     400
.....................................................................                480

(Threshold=0.5)
```

SeqName	Position	Potential	Jury agreement	N-Glyc result
WKF49011.1	50 NQSI	0.5884	(8/9)	+
WKF49011.1	58 NNTW	0.5496	(6/9)	+
WKF49011.1	63 NQTY	0.6636	(9/9)	++
WKF49011.1	68 NISN	0.7378	(9/9)	++
WKF49011.1	88 NSSL	0.7724	(9/9)	+++
WKF49011.1	146 NGTV	0.6876	(9/9)	++
WKF49011.1	235 NGSC	0.7321	(9/9)	++

NetNGlyc 1.0: predicted N-glycosylation sites in WKF49011.1

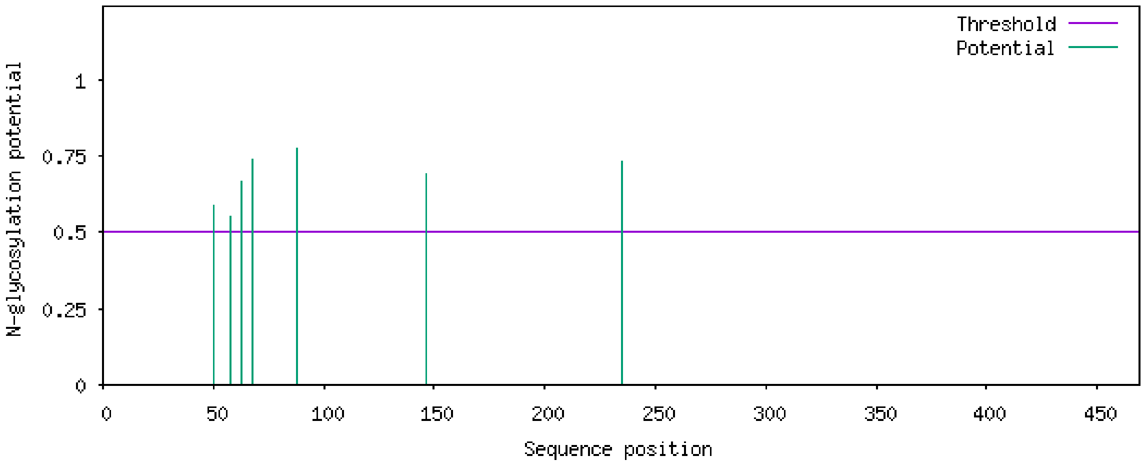

7. >WZL56507.1 hemagglutinin [Influenza A virus/feline/Texas/2024]

```
Name:  WZL56507.1       Length:  567
MENIVLLLAIVSLVKSDQICIGYHANNSTEQVDTIMEKNVTVTHAQDILEKTHNGKLCDLNGVKPLILKDCSVAGWLLGN      80
PMCDEFIRVPEWSYIVERANPANDLCYPGSLNDYEELKHMLSRINHFEKIQIIPKSSWPNHETSLGVSAACPYQGAPSFF     160
RNVVWLIKKNDAYPTIKISYNNTNREDLLILWGIHHSNNAEEQTNLYKNPITYISVGTSTLNQRLAPKIATRSQVNGQRG     240
RMDFFWTILKPDDAIHFESNGNFIAPEYAYKIVKKGDSTIMKSGVEYGHCNTKCQTPVGAINSSMPFHNIHPLTIGECPK     320
YVKSNKLVLATGLRNSPLREKRRKRGLFGAIAGFIEGGWQGMVDGWYGYHHSNEQGSGYAADKESTQKAIDGVTNKVNSI     400
IDKMNTQFEAVGREFNNLERRIENLNKKMEDGFLDVWTYNAELLVLMENERTLDFHDSNVKNLYDKVRLQLRDNAKELGN     480
GCFEFYHKCDNECMESVRNGTYDYPQYSEEARLKREEISGVKLESVGTYQILSIYSTAASSLALAIMMAGLSLWMCSNGS     560
LQCRICI                                                                              640
..........................N...........N.........................................      80
................................................................................     160
....................N...........................................................     240
.............................................................N..................     320
................................................................................     400
................................................................................     480
..................N...........................................................N..     560
.......                                                                              640

(Threshold=0.5)
----------------------------------------------------------------------
SeqName      Position  Potential   Jury    N-Glyc
                                 agreement result
----------------------------------------------------------------------
WZL56507.1     26 NNST   0.3686    (9/9)    --
WZL56507.1     27 NSTE   0.7796    (9/9)    +++
WZL56507.1     39 NVTV   0.7182    (9/9)    ++
WZL56507.1    181 NNTN   0.6100    (7/9)    +
WZL56507.1    302 NSSM   0.5447    (6/9)    +
WZL56507.1    499 NGTY   0.5826    (6/9)    +
WZL56507.1    558 NGSL   0.6827    (9/9)    ++
----------------------------------------------------------------------
```

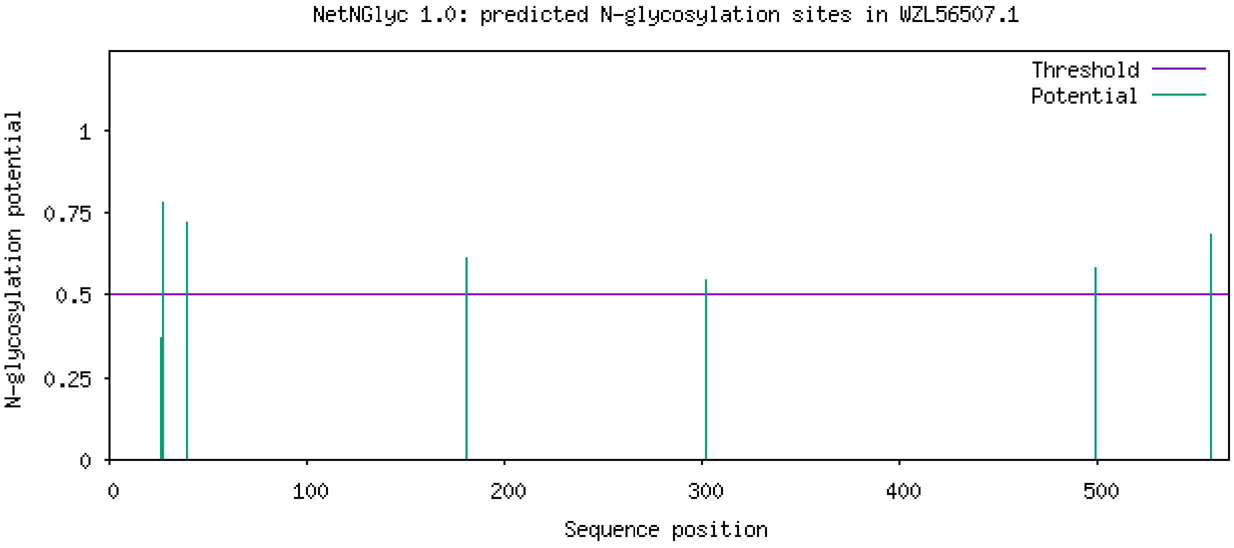

>WZL56509.1 neuraminidase [Influenza A virus/Feline/Texas]

```
Name:  WZL56509.1       Length:  469
MNPNQKITTIGSICMVIGIVSLMLQIGNIISIWVSHSIQTGNQYQPEPCNQSIITYENNTWVNQTYINISSTNFLAEQAV      80
TSVTLAGNSSLCPISGWAIYSKDNGIRIGSKGDVFVIREPFISCSHLECRTFFLTQGALLNDKHSNGTVKDRSPYRTLMS     160
CPVGEAPSPYNSRFESVAWSASACHDGISWLTIGISGPDNGAVAVLKYNGIITDTIKSWRNNILRTQESECACVNGSCFT     240
VMTDGPSNGQASYKIFKIEKGKVVKSVEMNAPNYHYEECSCYPDAGDIMCVCRDNWHGSNRPWVSFNQNLEYQIGYICSG     320
IFGDNPRPNDGTGSCSPMPSNGAYGVKGFSFKYGNGVWIGRTKSTSSRSGFEMIWDPNGWTETDSSFSVKQDIVEITDWS     400
GYSGSFVQHPELTGLDCMRPCFWVELIRGRPKENTIWTSGSSISFCGVNSDTVGWSWPDGAELPFTIDK                480
.................................................N.......N....N....N............      80
.......N.........................................................N..............     160
..........................................................................N.....     240
................................................................................     320
................................................................................     400
.....................................................................                480

(Threshold=0.5)
----------------------------------------------------------------------
SeqName      Position  Potential    Jury    N-Glyc
                                 agreement  result
----------------------------------------------------------------------
WZL56509.1     50 NQSI    0.5883     (8/9)   +
WZL56509.1     58 NNTW    0.5251     (5/9)   +
WZL56509.1     63 NQTY    0.6874     (9/9)   ++
WZL56509.1     68 NISS    0.7140     (9/9)   ++
WZL56509.1     88 NSSL    0.7724     (9/9)   +++
WZL56509.1    146 NGTV    0.6873     (9/9)   ++
WZL56509.1    235 NGSC    0.7321     (9/9)   ++
----------------------------------------------------------------------
```

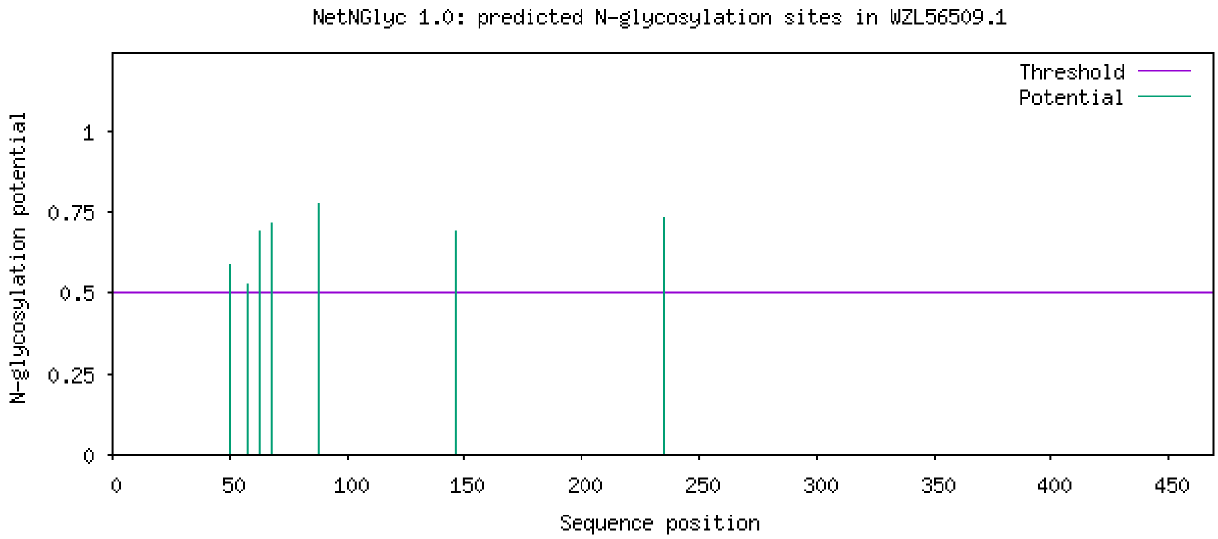

8. >XLE44792.1 hemagglutinin [Influenza A virus/Emu/CA/24-031663-001-original/2024]

```
Name:  XLE44792.1      Length:  567
MKNIVLLLAIVSLVKSDQICIGYHANNSTEQVDTIMEKNVTVTHAQDILEKTHNGKLCDLNGVKPLILKDCSVAGWLLGN      80
PMCDEFIRVPEWSYIVERANPANGLCYPGSLNDYEELKHMLSRINHFEKIQIIPKSSWPNHETSLGVSAACPYQGAPSFF     160
RNVVWLIKKNDAYPTIKISYNNTNREDLLILWGIHHSNNAEEQTNLYKNPITYISVGTSTLNQRLAPKIATRSQVNGQRG     240
RMDFFWTILKPDDAIHFESNGNFIAPEYAYKIVKKGDSTIMKSGVEYGHCNTKCQTPVGAINSSMPFHNIHPLTIGECPK     320
YVKSNKLVLATGLRNNPLREKRRKRGLFGAIAGFIEGGWQGMVDGWYGYHHSNEQGSGYAADKESTQKAIDGVTNKVNSI     400
IDKMNTQFEAVGREFNNLERRIENLNKKMEDGFLDVWTYNAELLVLMENERTLDFHDSNVKNLYDKVRLQLRDNAKELGN     480
GCFEFYHKCDNECMESVRNGTYDYPQYSEEARLKREEISGVKLESVGTYQILSIYSTAASSLALAIMMAGLSLWMCSNGS     560
LQCRICI                                                                              640
..........................N...........N.........................................      80
................................................................................     160
....................N...........................................................     240
.............................................................N..................     320
................................................................................     400
................................................................................     480
..................N..........................................................N..     560
.......                                                                              640
```

(Threshold=0.5)

SeqName	Position	Potential	Jury agreement	N-Glyc result
XLE44792.1	26 NNST	0.3687	(9/9)	--
XLE44792.1	27 NSTE	0.7796	(9/9)	+++
XLE44792.1	39 NVTV	0.7180	(9/9)	++
XLE44792.1	181 NNTN	0.6100	(7/9)	+
XLE44792.1	302 NSSM	0.5447	(6/9)	+
XLE44792.1	499 NGTY	0.5824	(6/9)	+
XLE44792.1	558 NGSL	0.6829	(9/9)	++

NetNGlyc 1.0: predicted N-glycosylation sites in XLE44792.1

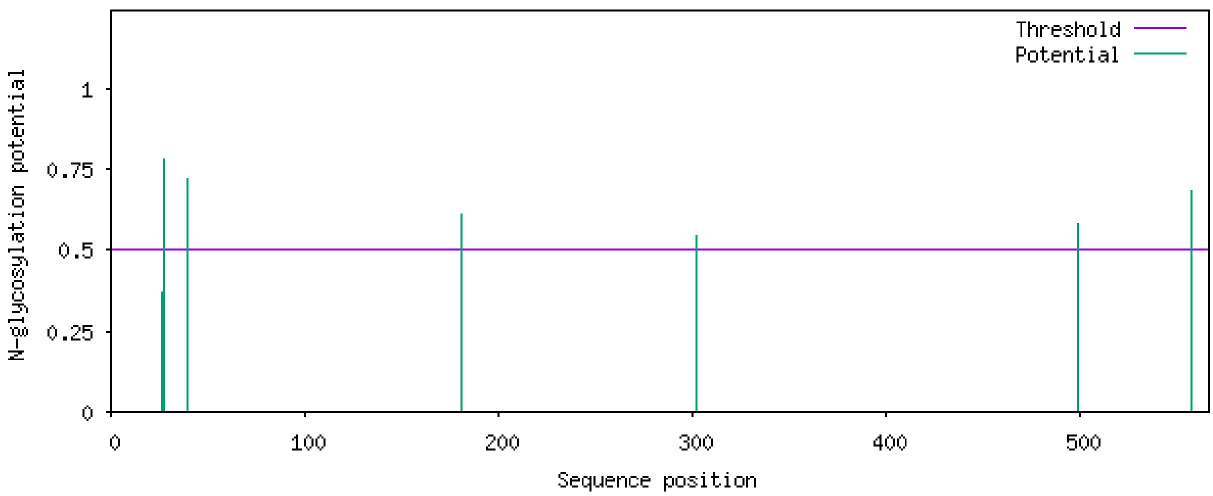

>XLE44794.1 neuraminidase [Influenza A virus/Emu/2024]

```
Name:  XLE44794.1       Length:  469
MNPNQKITTIGSICMVIGIVSLMLQIGNIISIWVSHSIQTGNQYQPEPCNQSIITYENNTWVNQTYINISSTNFLAEQAV      80
TSVTLAGNSSLCPISGWAIYSKDNGIRIGSKGDVFVIREPFISCSHLECRTFFLTQGALLNDKHSNGTVKDRSPYRTLMS     160
CPVGEAPSPYNSRFESVAWSASACHDGISWLTIGISGPDNGAVAVLKYNGIITDTIKSWRNNILRTQESECACVNGSCFT     240
VMTDGPSNGQASYKIFKIEKGKVVKSVEMNAPNYHYEECSCYPDAGDIMCVCRDNWHGSNRPWVSFNQNLEYQIGYICSG     320
IFGDNPRPNDGTGSCSPMPSNGAYGVKGFSFKYGNGVWIGRTKSTSSRSGFEMIWDPNGWTETDSSFSVKQDIVEITDWS     400
GYSGSFVQHPELTGLDCMRPCFWVELIRGRPKENTIWTSGSSISFCGVNSDTVGWSWPDGAELPFTIDK                480
.................................................N.......N....N....N............      80
.......N.........................................................N..............     160
..........................................................................N.....     240
................................................................................     320
................................................................................     400
.....................................................................                480

(Threshold=0.5)
----------------------------------------------------------------------
SeqName      Position  Potential   Jury    N-Glyc
                                 agreement result
----------------------------------------------------------------------
XLE44794.1     50 NQSI   0.5883     (8/9)    +
XLE44794.1     58 NNTW   0.5251     (5/9)    +
XLE44794.1     63 NQTY   0.6874     (9/9)    ++
XLE44794.1     68 NISS   0.7140     (9/9)    ++
XLE44794.1     88 NSSL   0.7724     (9/9)    +++
XLE44794.1    146 NGTV   0.6873     (9/9)    ++
XLE44794.1    235 NGSC   0.7321     (9/9)    ++
----------------------------------------------------------------------
```

NetNGlyc 1.0: predicted N-glycosylation sites in XLE44794.1

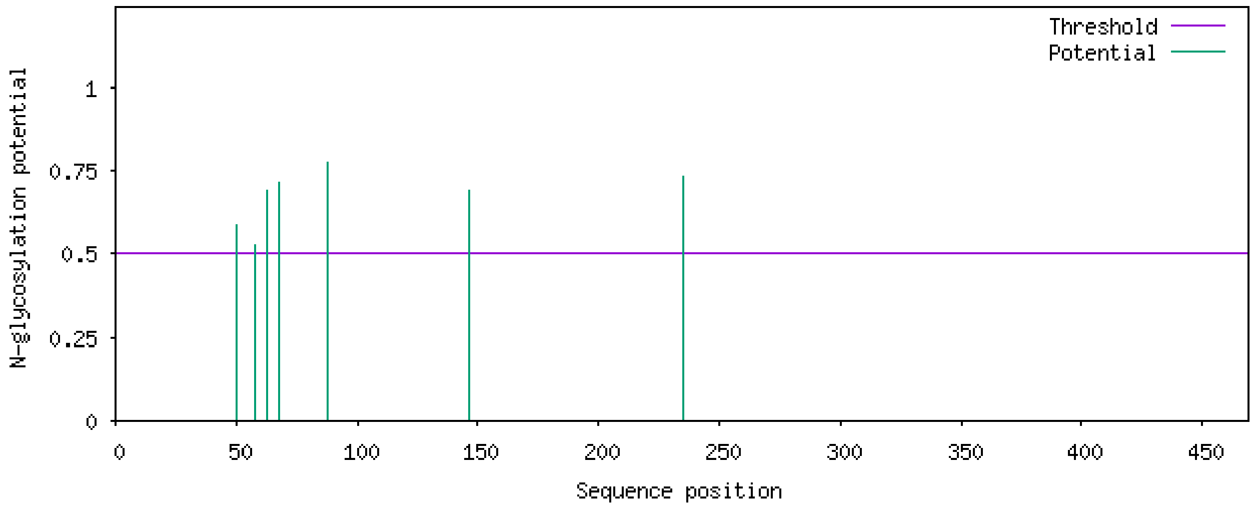

9. >XCZ64304.1 hemagglutinin [Influenza A virus/Red-Fox/MI/24-013016-004-original/2024]

```
Name:  XCZ64304.1       Length:  567
MENIVLLLAIVSLVKSDQICIGYHANNSTEQVDTIMEKNVTVTHAQDILEKTHNGKLCDLNGVKPLILKDCSVAGWLLGN      80
PMCDEFIRVPEWSYIVERANPANDLCYPGSLNDYEELKHMLSRINHFEKIQIIPKSSWPNHETSLGVSAACPYQGAPSFF     160
RNVVWLIKKNDAYPTIKISYNNTNREDLLILWGIHHSNNAEEQTNLYKNPITYISVGTSTLNQRLAPKIATRSQVNGQRG     240
RMDFFWTILKPDDAIHFESNGNFIAPEYAYKIVKKGDSTIMKSGVEYGHCNTKCQTPVGAINSSMPFHNIHPLTIGECPK     320
YVKSNKLVLATGLRNSPLREKRRKRGLFGAIAGFIEGGWQGMVDGWYGYHHSNEQGSGYAADKESTQKAIDGVTNKVNSI     400
IDKMNTQFEAVGREFNNLERRIENLNKKMEDGFLDVWTYNAELLVLMENERTLDFHDSNVKNLYDKVRLQLRDNAKELGN     480
GCFEFYHKCDNECMESVRNGTYDYPQYSEEARLKREEISGVKLESVGTYQILSIYSTAASSLALAIMMAGLSLWMCSNGS     560
LQCRICI                                                                              640
..........................N...........N.........................................      80
................................................................................     160
....................N...........................................................     240
.............................................................N..................     320
................................................................................     400
................................................................................     480
..................N..........................................................N..     560
.......                                                                              640

(Threshold=0.5)
----------------------------------------------------------------------
SeqName      Position  Potential   Jury    N-Glyc
                                  agreement result
----------------------------------------------------------------------
XCZ64304.1     26 NNST   0.3686     (9/9)   --
XCZ64304.1     27 NSTE   0.7796     (9/9)   +++
XCZ64304.1     39 NVTV   0.7182     (9/9)   ++
XCZ64304.1    181 NNTN   0.6100     (7/9)   +
XCZ64304.1    302 NSSM   0.5447     (6/9)   +
XCZ64304.1    499 NGTY   0.5826     (6/9)   +
XCZ64304.1    558 NGSL   0.6827     (9/9)   ++
----------------------------------------------------------------------
```

NetNGlyc 1.0: predicted N-glycosylation sites in XCZ64304.1

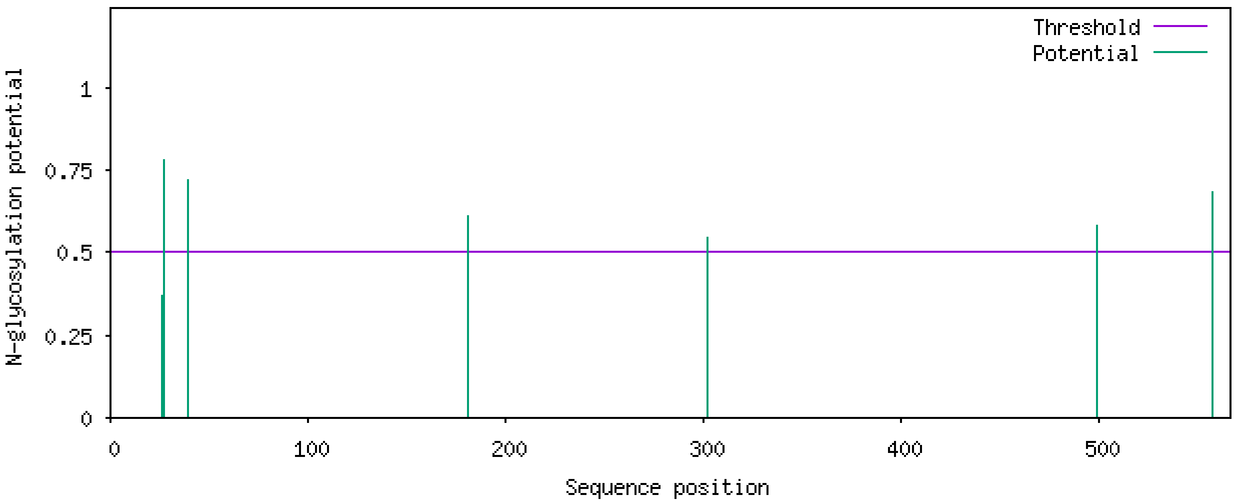

>XCZ64306.1 neuraminidase [Influenza A virus/Red-Fox/2024]

```
Name:  XCZ64306.1       Length:  469
MNPNQKITTIGSICMVIGIVSLMLQIGNIISIWVSHSIQTGNQYQPEPCNQSIITYENNTWVNQTYINISNTNFLAEQAV      80
TSVTLAGNSSLCPISGWAIYSKDNGIRIGSKGDVFVIREPFISCSHLECRTFFLTQGALLNDKHSNGTVKDRSPYRTLMS     160
CPVGEAPSPYNSRFESVAWSASACHDGISWLTIGISGPDNGAVAVLKYNGIITDTIKSWRNNILRTQESECACVNGSCFT     240
VMTDGPSNGQASYKIFKIEKGKVVKSVEMNAPNYHYEECSCYPDAGDIMCVCRDNWHGSNRPWVSFNQNLEYQIGYICSG     320
IFGDNPRPNDGTGSCSPMPSNGAYGVKGFSFKYGNGVWIGRTKSTSSRSGFEMIWDPNGWTETDSSFSVKQDIVEITDWS     400
GYSGSFVQHPELTGLDCMRPCFWVELIRGRPKENTIWTSGSSISFCGVNSDTVGWSWPDGAELPFTIDK                480
.................................................N.......N....N....N............      80
.......N.........................................................N..............     160
..........................................................................N.....     240
................................................................................     320
................................................................................     400
.....................................................................                480

(Threshold=0.5)
----------------------------------------------------------------------
SeqName      Position  Potential   Jury    N-Glyc
                                 agreement result
----------------------------------------------------------------------
XCZ64306.1     50 NQSI   0.5881     (8/9)   +
XCZ64306.1     58 NNTW   0.5252     (5/9)   +
XCZ64306.1     63 NQTY   0.6762     (9/9)   ++
XCZ64306.1     68 NISN   0.6866     (7/9)   +
XCZ64306.1     88 NSSL   0.7724     (9/9)   +++
XCZ64306.1    146 NGTV   0.6874     (9/9)   ++
XCZ64306.1    235 NGSC   0.7321     (9/9)   ++
----------------------------------------------------------------------
```

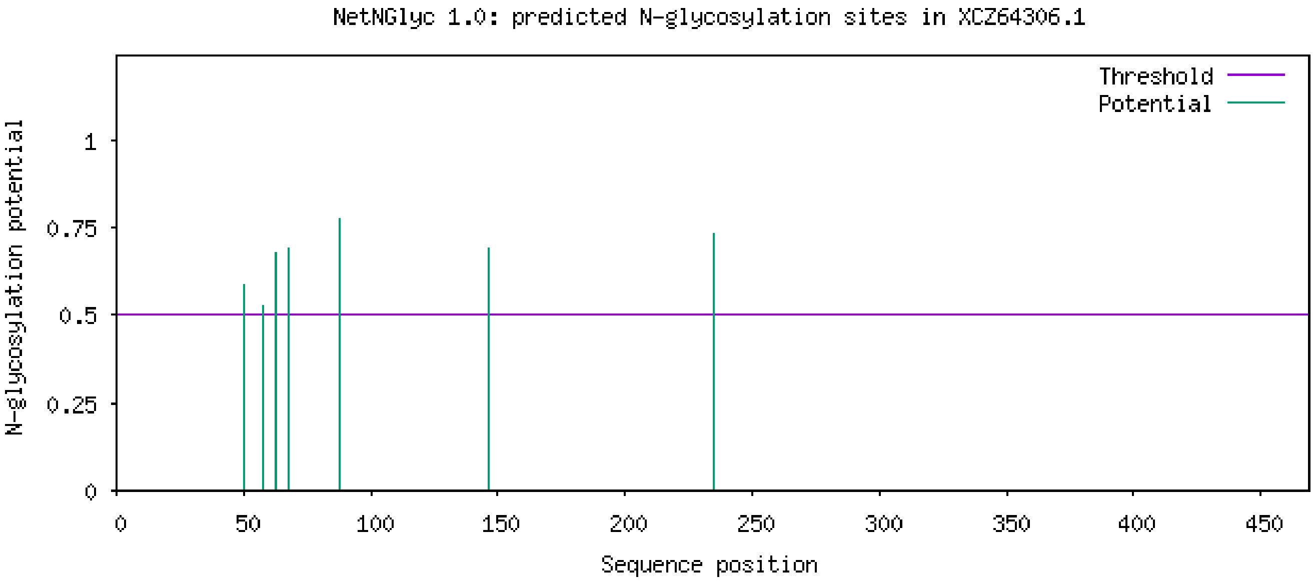

10. >WDV81467.1 hemagglutinin [Influenza A virus/Gallus-gallus/Peru/AIS0545/2022]

```
Name:  WDV81467.1       Length:  567
MENIVLLLAIVSLVKSDQICIGYHANNSTEQVDTIMEKNVTVTHAQDILEKTHNGKLCDLNGVKPLILKDCSVAGWLLGN      80
PMCDEFIRVPEWSYIVERANPANDLCYPGSLNDYEELKHMLSRINHFEKIQIIPKSSWPNHETSLGVSAACPYQGAPSFF     160
RNVVWLIKKNDAYPTIKISYNNTNREDLLILWGIHHSNNAEEQTNLYKNPTTYISVGTSTLNQRLAPKIATRSQVNGQRG     240
RMDFFWTILKPDDAIHFESNGNFIAPEYAYKIVKKGDSTIMKSGVEYGHCNTKCQTPVGAINSSMPFHNIHPLTIGECPK     320
YVKSNKLVLATGLRNSPLREKRRKRGLFGAIAGFIEGGWQGMVDGWYGYHHSNEQGSGYAADKESTQKAIDGVTNKVNSI     400
IDKMNTQFEAVGREFNNLERRIENLNKKMEDGFLDVWTYNAELLVLMENERTLDFHDSNVKNLYDKVRLQLRDNAKELGN     480
GCFEFYHKCDNECMESVRNGTYDYPQYSEEARLKREEISGVKLESVGTYQILSIYSTAASSLALAIMMAGLSLWMCSNGS     560
LQCRICI                                                                              640
..........................N...........N.........................................      80
................................................................................     160
....................N...........................N...............................     240
.............................................................N..................     320
................................................................................     400
................................................................................     480
..................N..........................................................N..     560
.......                                                                              640

(Threshold=0.5)
----------------------------------------------------------------------
SeqName      Position  Potential   Jury    N-Glyc
                                 agreement result
----------------------------------------------------------------------
WDV81467.1    26 NNST   0.3686     (9/9)   --
WDV81467.1    27 NSTE   0.7797     (9/9)   +++
WDV81467.1    39 NVTV   0.7182     (9/9)   ++
WDV81467.1   181 NNTN   0.6100     (7/9)   +
WDV81467.1   209 NPTT   0.6336     (8/9)   +    WARNING: PRO-X1.
WDV81467.1   302 NSSM   0.5447     (6/9)   +
WDV81467.1   499 NGTY   0.5824     (6/9)   +
WDV81467.1   558 NGSL   0.6827     (9/9)   ++
----------------------------------------------------------------------
```

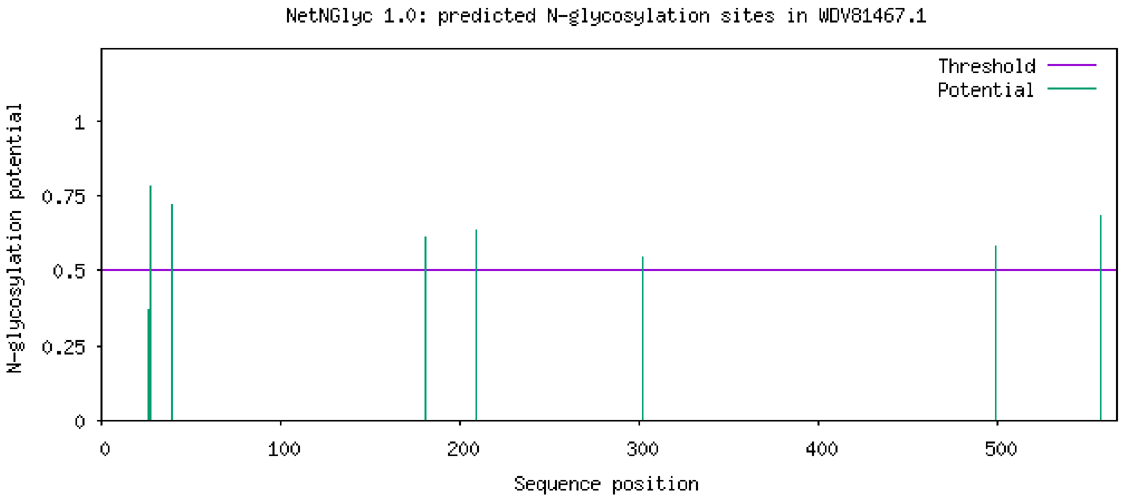

>WDV81469.1 neuraminidase [Influenza A virus/Gallus-gallus/Peru/2022]

```
Name:  WDV81469.1      Length:  469
MNPNQKITTIGSICMVIGIVSLMLQIGNIISIWVSHSIQTGNQYQPEPCNQSIITYENNTWVNQTYVNISNTNFLAEQAV      80
TSVTLAGNSSLCPISGWAIYSKDNGIRIGSKGDVFVIREPFISCSHLECRTFFLTQGALLNDKHSNGTVKDRSPYRTLMS     160
CPVGEAPSPYNSRFESVAWSASACHDGISWLTIGISGPDNGAVAVLKYNGIITDTIKSWRNNILRTQESECACVNGSCFT     240
VMTDGPSNGQASYKIFKIEKGKVVKSVEMNAPNYHYEECSCYPDAGDIMCVCRDNWHGSNRPWVSFNQNLEYQIGYICSG     320
VFGDNPRPNDGTGSCSPMPSNGAYGVKGFSFKYGNGVWIGRTKSTSSRSGFEMIWDPNGWTETDSSFSVKQDIVEITDWS     400
GYSGSFVQHPELTGLDCMRPCFWVELIRGRPKENTIWTSGSSISFCGVNSDTVGWSWPDGAELPFTIDK                480
.................................................N.......N....N....N............      80
.......N.........................................................N..............     160
..........................................................................N.....     240
................................................................................     320
................................................................................     400
.....................................................................                480

(Threshold=0.5)
----------------------------------------------------------------------
SeqName      Position  Potential   Jury    N-Glyc
                                agreement  result
----------------------------------------------------------------------
WDV81469.1    50 NQSI   0.5884     (8/9)   +
WDV81469.1    58 NNTW   0.5496     (6/9)   +
WDV81469.1    63 NQTY   0.6636     (9/9)   ++
WDV81469.1    68 NISN   0.7378     (9/9)   ++
WDV81469.1    88 NSSL   0.7724     (9/9)   +++
WDV81469.1   146 NGTV   0.6876     (9/9)   ++
WDV81469.1   235 NGSC   0.7321     (9/9)   ++
----------------------------------------------------------------------
```

NetNGlyc 1.0: predicted N-glycosylation sites in WDV81469.1

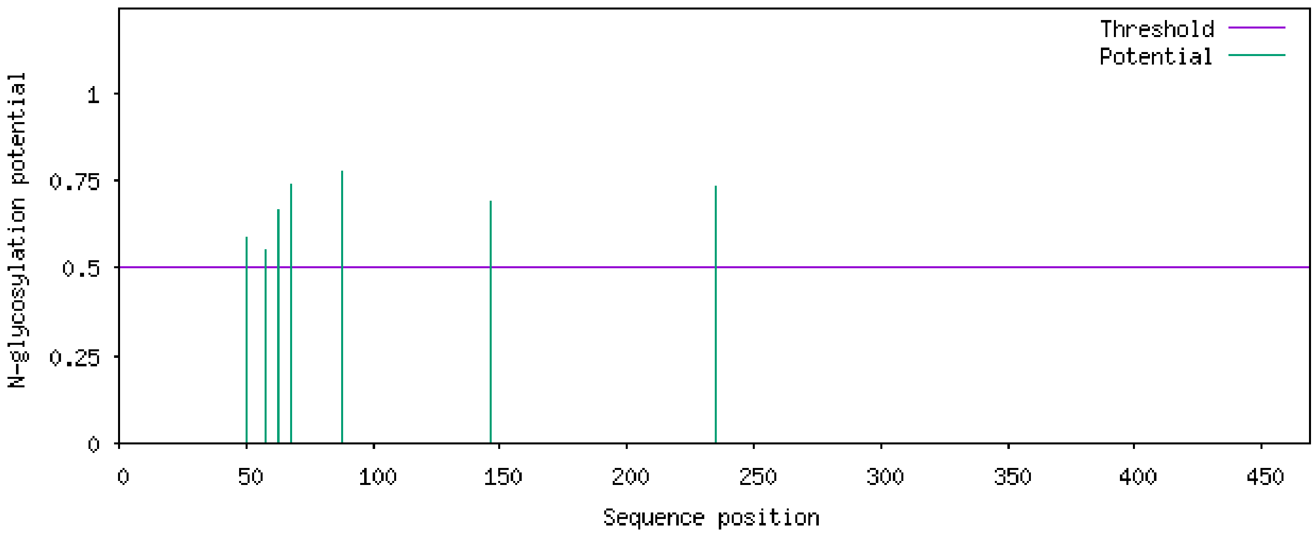

11. >XAJ25247.1 hemagglutinin [Influenza A virus/A/goat/Minnesota/24-007234-059/2024]

```
Name:  XAJ25247.1       Length:  567
MENIVLLLAIVSLVKSDQICIGYHANNSTEQVDTIMEKNVTVTHAQDILEKTHNGKLCDLNGVKPLILKDCSVAGWLLGN      80
PMCDEFIRVPEWSYIVERANPANDLCYPGSLNDYEELKHMLSRINHFEKIQIIPKSSWPNHETSLGVSAACPYQGAPSFF     160
RNVVWLIKKNDAYPTIKISYNNTNREDLLILWGIHHSNNAEEQTNLYKNPITYISVGTSTLNQRLAPKIATRSQVNGQRG     240
RMDFFWTILKPDDAIHFESNGNFIAPEYAYKIVKKGDSTIMKSGVEYGHCNTKCQTPVGAINSSMPFHNIHPLTIGECPK     320
YVKSNKLVLATGLRNSPLREKRRKRGLFGAIAGFIEGGWQGMVDGWYGYHHSNEQGSGYAADKESTQKAIDGVTNKVNSI     400
IDKMNTQFEAVGREFNNLERRIENLNKKMEDGFLDVWTYNAELLVLMENERTLDFHDSNVKNLYDKVRLQLRDNAKELGN     480
GCFEFYHKCDNECMESVRNGTYDYPQYSEEARLKREEIRGVKLESVGTYQILSIYSTAASSLALAIMMAGLSLWMCSNGS     560
LQCRICI                                                                              640
..........................N...........N.........................................      80
................................................................................     160
....................N...........................................................     240
.............................................................N..................     320
................................................................................     400
................................................................................     480
..................N..........................................................N..     560
.......                                                                              640

----------------------------------------------------------------------
Sequence      26 NNST   0.3686     (9/9)   --
Sequence      27 NSTE   0.7796     (9/9)   +++
Sequence      39 NVTV   0.7182     (9/9)   ++
Sequence     181 NNTN   0.6100     (7/9)   +
Sequence     302 NSSM   0.5447     (6/9)   +
Sequence     499 NGTY   0.5824     (6/9)   +
Sequence     558 NGSL   0.6828     (9/9)   ++
----------------------------------------------------------------------
```

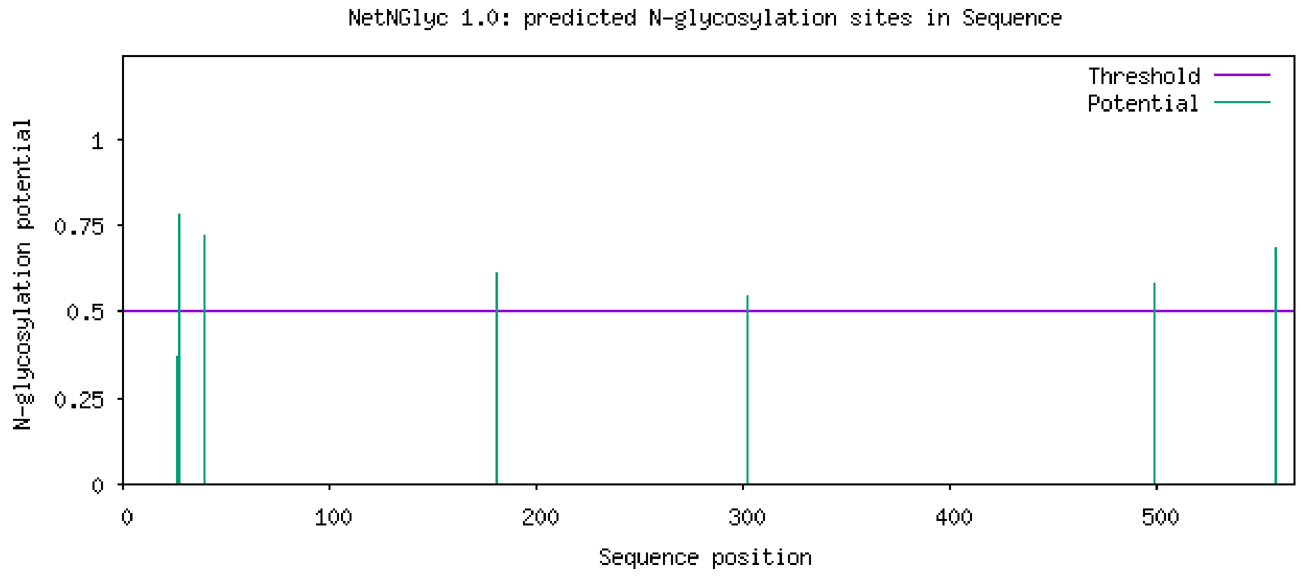

>XAJ25250.1 neuraminidase [Influenza A virus/goat/Minnesota/24-007234-059/2024]

```
Name:  XAJ25250.1      Length:  469
MNPNQKITTIGSICMVIGIASLMLQIGNIISIWVSHSIQTGNQYQPEPCNQSIITYENNTWVNQTYVNISNTNFLAEQAV      80
TSVTLAGNSSLCPISGWAIYSKDNGIRIGSKGDVFVIREPFISCSHLECRTFFLTQGALLNDKHSNGTVKDRSPHRTLMS     160
CPVGEAPSPYNSRFESVAWSASACHDGISWLTIGISGPDNGAVAVLKYNGIITDTIKSWRNNILRTQESECACVNGSCFT     240
VMTDGPSNGQASYKIFKIEKGKVVKSVEMNAPNYHYEECSCYPDAGDIMCVCRDNWHGSNRPWVSFNQNLEYQIGYICSG     320
IFGDNPRPNDGTGSCSPMPSNGAYGVKGFSFKYGNGVWIGRTKSTSSRNGFEMIWDPNGWTETDSSFSVKQDIVEITDWS     400
GYSGSFVQHPELTGLDCMRPCFWVELIRGRPKENTIWTSGSSISFCGVNSDTVGWSWPDGAELPFTIDK                480
.................................................N.......N....N....N............      80
.......N.........................................................N..............     160
..........................................................................N.....     240
................................................................................     320
................................................................................     400
.....................................................................                480
```

(Threshold=0.5)

SeqName	Position	Potential	Jury agreement	N-Glyc result
XAJ25250.1	50 NQSI	0.5882	(8/9)	+
XAJ25250.1	58 NNTW	0.5494	(6/9)	+
XAJ25250.1	63 NQTY	0.6636	(9/9)	++
XAJ25250.1	68 NISN	0.7377	(9/9)	++
XAJ25250.1	88 NSSL	0.7724	(9/9)	+++
XAJ25250.1	146 NGTV	0.6880	(9/9)	++
XAJ25250.1	235 NGSC	0.7320	(9/9)	++

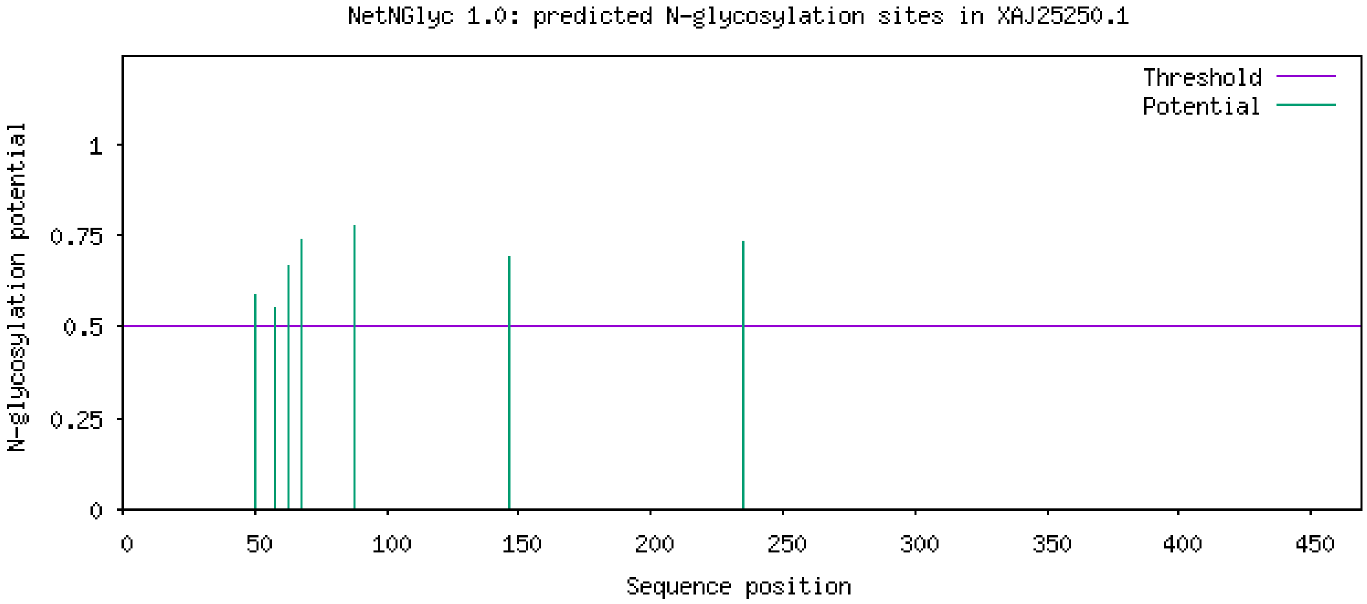

12. >WZL56530.1 hemagglutinin [Influenza A virus/Calidris-alba/Lima]

```
Name:  WZL56530.1       Length:  567
MENIVLLLAIVSLVKSDQICIGYHANNSTEQVDTIMEKNVTVTHAQDILEKTHNGKLCDLNGVKPLILKDCSVAGWLLGN      80
PMCDEFIRVPEWSYIVERANPANDLCYPGSLNDYEELKHMLSRINHFEKIQIIPKSSWPNHETSLGVSAACPYQGAPSFF     160
RNVVWLIKKNDAYPTIKISYNNTNREDLLILWGIHHSNNAREQTNLYKNPTTYISVGTSTLNQRLAPKIATRSQVNGQRG     240
RMDFFWTILKPDDAIHFESNGNFIAPEYAYKIVKKGDSTIMKSGVEYGHCNTKCQTPVGAINSSMPFHNIHPLTIGECPK     320
YVKSNKLVLATGLRNSPLREKRRKRGLFGAIAGFIEGGWQGMVDGWYGYHHSNEQGSGYAADKESTQKAIDGVTNKVNSI     400
IDKMNTQFEAVGREFNNLERRIENLNKKMEDGFLDVWTYNAELLVLMENERTLDFHDSNVKNLYDKVRLQLRDNAKELGN     480
GCFEFYHKCDNECMESVRNGTYDYPQYSEEARLKREEISGVKLESVGTYQILSIYSTAASSLALAIMMAGLSLWMCSNGS     560
LQCRICI                                                                              640
..........................N...........N.........................................      80
................................................................................     160
....................N...........................N...............................     240
.............................................................N..................     320
................................................................................     400
................................................................................     480
..................N..........................................................N..     560
.......                                                                              640
```

(Threshold=0.5)

SeqName	Position	Potential	Jury agreement	N-Glyc result	
WZL56530.1	26 NNST	0.3687	(9/9)	--	
WZL56530.1	27 NSTE	0.7797	(9/9)	+++	
WZL56530.1	39 NVTV	0.7181	(9/9)	++	
WZL56530.1	181 NNTN	0.6100	(7/9)	+	
WZL56530.1	209 NPTT	0.6324	(8/9)	+	WARNING: PRO-X1.
WZL56530.1	302 NSSM	0.5447	(6/9)	+	
WZL56530.1	499 NGTY	0.5824	(6/9)	+	
WZL56530.1	558 NGSL	0.6827	(9/9)	++	

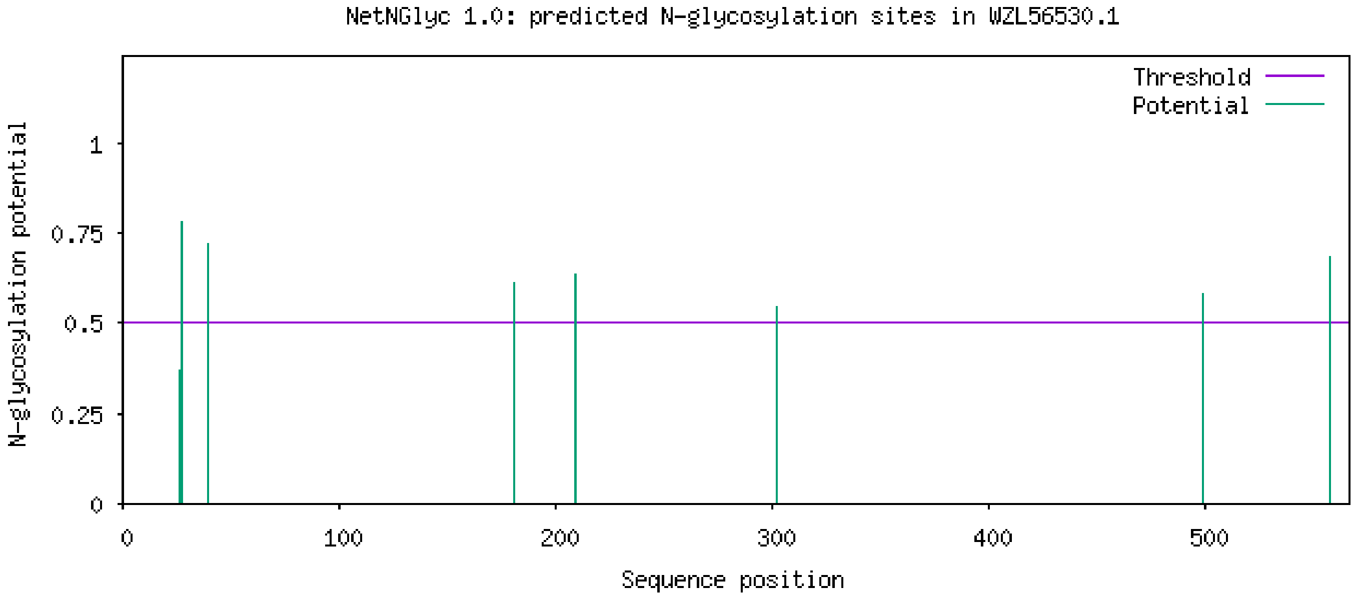

>WZL56532.1 neuraminidase [Influenza A virus/Calidris-alba/Lima]

```
Name:  WZL56532.1        Length:  469
MNPNQKITTIGSICMVIGIVSLMLQIGNIISIWVSHSIQTGNQYQPEPCNQSIITYENNTWVNQTYVNISNTNFLAEQAV      80
TSVTLAGNSSLCPISGWAIYSKDNGIRIGSKGDVFVIREPFISCSHLECRTFFLTQGALLNDKHSNGTVKDRSPYRTLMS     160
CPVGEAPSPYNSRFESVAWSASACHDGISWLTIGISGPDNGAVAVLKYNGIITDTIKSWRNNILRTQESECACVNGSCFT     240
VMTDGPSNGQASYKIFKIEKGKVVKSVEMNAPNYHYEECSCYPDAGDIMCVCRDNWHGSNRPWVSFNQNLEYQIGYICSG     320
VFGDNPRPNDGTGSCSPMPSNGAYGVKGFSFKYGNGVWIGRTKSTSSRSGFEMIWDPNGWTETDSSFSVKQDIVEITDWS     400
GYSGSFVQHPELTGLDCMRPCFWVELIRGRPKENTIWTSGSIISFCGVNSDTVGWSWPDGAELPFTIDK                480
.................................................N.......N....N....N............      80
.......N.........................................................N..............     160
..........................................................................N.....     240
................................................................................     320
................................................................................     400
.....................................................................                480

(Threshold=0.5)
----------------------------------------------------------------------
SeqName       Position  Potential    Jury    N-Glyc
                                   agreement result
----------------------------------------------------------------------
WZL56532.1     50 NQSI   0.5884     (8/9)    +
WZL56532.1     58 NNTW   0.5497     (6/9)    +
WZL56532.1     63 NQTY   0.6637     (9/9)    ++
WZL56532.1     68 NISN   0.7378     (9/9)    ++
WZL56532.1     88 NSSL   0.7724     (9/9)    +++
WZL56532.1    146 NGTV   0.6876     (9/9)    ++
WZL56532.1    235 NGSC   0.7321     (9/9)    ++
----------------------------------------------------------------------
```

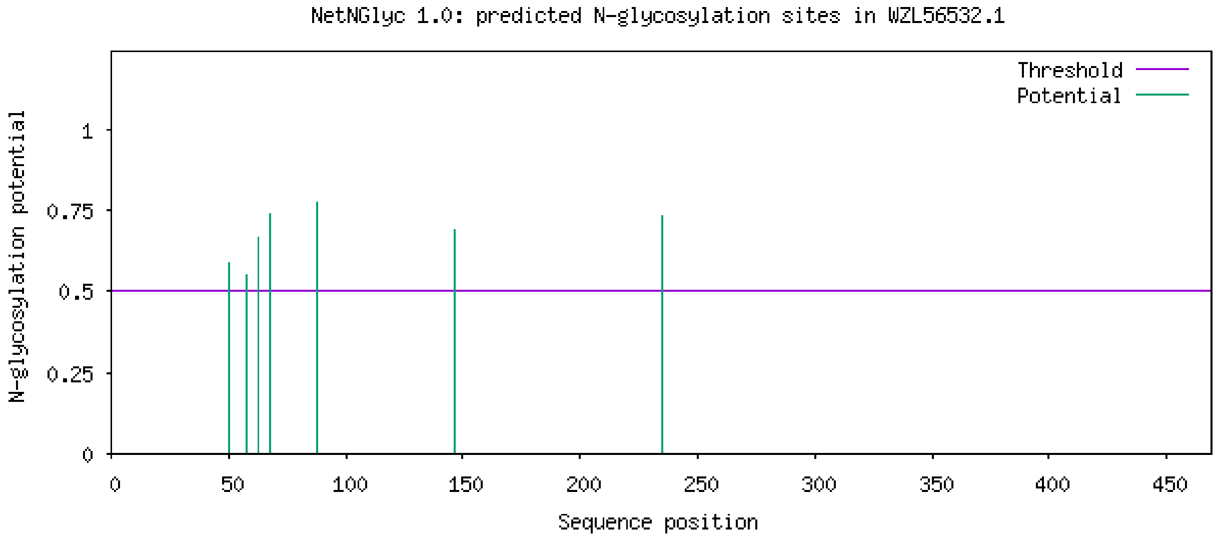

13. >AAD51927.1 hemagglutinin [Influenza A virus (A/goose/Guangdong/1/1996(H5N1))]

```
Name:  AAD51927.1      Length:  568
MEKIVLLLAIVSLVKSDQICIGYHANNSTEQVDTIMEKNVTVTHAQDILEKTHNGKLCDLNGVKPLILRDCSVAGWLLGN      80
PMCDEFINVPEWSYIVEKASPANDLCYPGDFNDYEELKHLLSRTNHFEKIQIIPKSSWSNHDASSGVSSACPYHGRSSFF     160
RNVVWLIKKNSAYPTIKRSYNNTNQEDLLVLWGIHHPNDAAEQTKLYQNPTTYISVGTSTLNQRLVPEIATRPKVNGQSG     240
RMEFFWTILKPNDAINFESNGNFIAPEYAYKIVKKGDSAIMKSELEYGNCNTKCQTPMGAINSSMPFHNIHPLTIGECPK     320
YVKSNRLVLATGLRNTPQRERRRKKRGLFGAIAGFIEGGWQGMVDGWYGYHHSNEQGSGYAADKESTQKAIDGVTNKVNS     400
IIDKMNTQFEAVGREFNNLERRIENLNKQMEDGFLDVWTYNAELLVLMENERTLDFHDSNVKNLYDKVRLQLRDNAKELG     480
NGCFEFYHKCDNECMESVKNGTYDYPQYSEEARLNREEISGVKLESMGTYQILSIYSTVASSLALAIMVAGLSLWMCSNG     560
SLQCRICI                                                                             640
..........................N...........N.........................................      80
................................................................................     160
....................N...........................N...............................     240
..............................................................N.................     320
................................................................................     400
................................................................................     480
...................N............................................................N.     560
........                                                                             640
```

(Threshold=0.5)

SeqName	Position	Potential	Jury agreement	N-Glyc result	
AAD51927.1	26 NNST	0.3687	(9/9)	--	
AAD51927.1	27 NSTE	0.7797	(9/9)	+++	
AAD51927.1	39 NVTV	0.7180	(9/9)	++	
AAD51927.1	181 NNTN	0.6240	(8/9)	+	
AAD51927.1	209 NPTT	0.6814	(8/9)	+	WARNING: PRO-X1.
AAD51927.1	302 NSSM	0.5997	(6/9)	+	
AAD51927.1	500 NGTY	0.5263	(4/9)	+	
AAD51927.1	559 NGSL	0.6841	(9/9)	++	

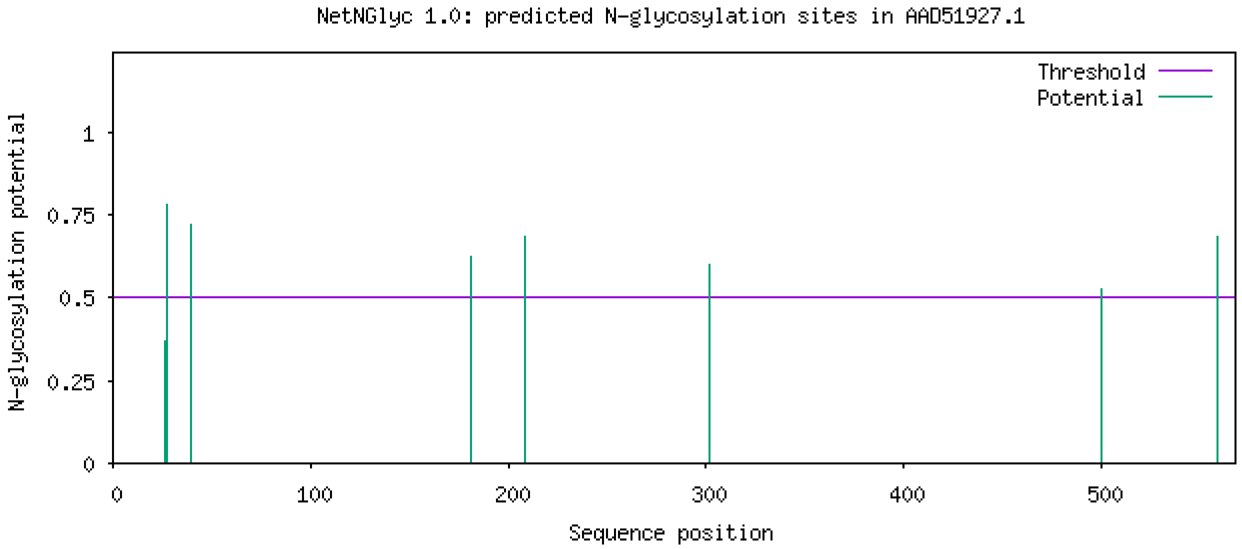

>AAD51926.1 neuraminidase [Influenza A virus (A/goose/Guangdong/1/1996(H5N1))]

```
Name:  AAD51926.1       Length:  469
MNPNQKIITIGSICMVVGIISLMLQIGNIISIWVSHSIQTGNQHQAEPCNQSIITYENNTWVNQTYVNISNTNFLTEKAV      80
ASVTLAGNSSLCPISGWAVHSKDNGIRIGSKGDVFVIREPFISCSHLECRTFFLTQGALLNDKHSNGTVKDRSPHRTLMS     160
CPVGEAPSPYNSRFESVAWSASACHDGTSWLTIGISGPDNGAVAVLKYNGIITDTIKSWRNNILRTQESECACVNGSCFT     240
VMTDGPSNGQASYKIFKMEKGKVVKSVELNAPNYHYEECSCYPDAGEITCVCRDNWHGSNRPWVSFNQNLEYQIGYICSG     320
VFGDNPRPNDGTGSCGPVSPNGAYGVKGFSFKYGNGVWIGRTKSTNSRSGFEMIWDPNGWTGTDSSFSVKQDIVAITDWS     400
GYSGSFVQHPELTGLDCIRPCFWVELIRGRPKESTIWTSGSSISFCGVNSDTVGWSWPDDAELPFTIDK                480
.................................................N.......N....N....N............      80
.......N.........................................................N..............     160
..........................................................................N.....     240
................................................................................     320
................................................................................     400
.....................................................................                480

(Threshold=0.5)
----------------------------------------------------------------------
SeqName      Position  Potential   Jury    N-Glyc
                                 agreement result
----------------------------------------------------------------------
AAD51926.1     50 NQSI   0.5516    (7/9)    +
AAD51926.1     58 NNTW   0.5496    (6/9)    +
AAD51926.1     63 NQTY   0.6633    (9/9)    ++
AAD51926.1     68 NISN   0.7391    (9/9)    ++
AAD51926.1     88 NSSL   0.7739    (9/9)    +++
AAD51926.1    146 NGTV   0.6880    (9/9)    ++
AAD51926.1    235 NGSC   0.7321    (9/9)    ++
----------------------------------------------------------------------
```

NetNGlyc 1.0: predicted N-glycosylation sites in AAD51926.1

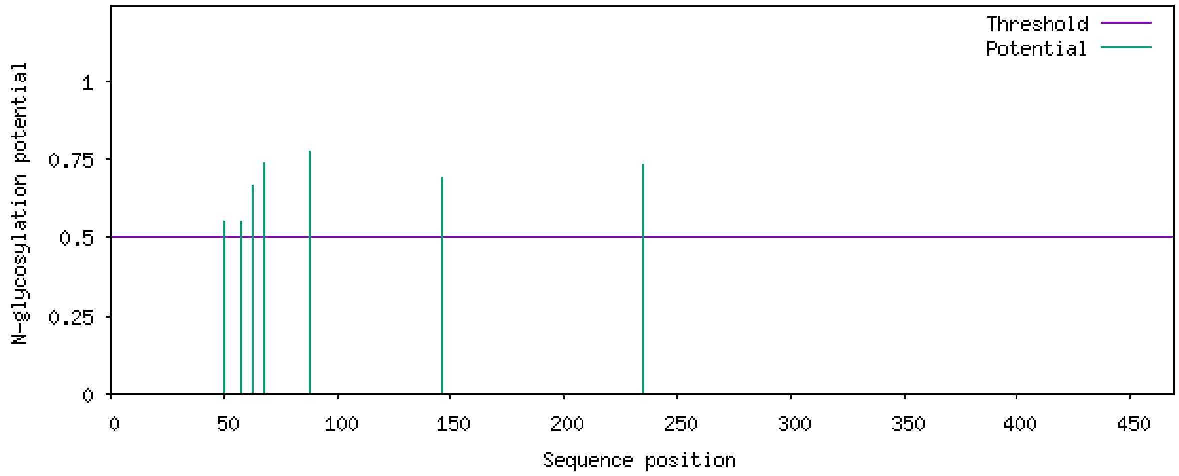

14. >WDV81644.1 hemagglutinin [Influenza A virus/Panthera leo/Peru]

```
Name:  WDV81644.1       Length:  567
MENIVLLLAIVSLVKSDQICIGYHANNSTEQVDTIMEKNVTVTHAQDILEKTHNGKLCDLNGVKPLILKDCSVAGWLLGN      80
PMCDEFIRVPEWSYIVERANPANDLCYPGSLNDYEELKHMLSRINHFEKIQIIPKSSWPNHETSLGVSAACPYQGAPSFF     160
RNVVWLIKKNDAYPTIKISYNNTNREDLLILWGIHHSNNAEEQTNLYKNPTTYISVGTSTLNQRLAPKIATRSQVNGQRG     240
RMDFFWTILKPDDAIHFESNGNFIAPEYAYKIVKKGDSTIMKSGVEYGHCNTKCQTPVGAINSSMPFHNVHPLTIGECPK     320
YVKSNKLVLATGLRNSPLRGKRRKRGLFGAIAGFIEGGWQGMVDGWYGYHHSNEQGSGYAADKESTQKAIDGVTNKVNSI     400
IDKMNTQFEAVGREFNNLERRIENLNKKMEDGFLDVWTYNAELLVLMENERTLDFHDSNVKNLYDKVRLQLRDNAKELGN     480
GCFEFYHKCDNECMESVRNGTYDYPQYSEEARLKREEISGVKLESVGTYQILSIYSTAASSLALAIMMAGLSLWMCSNGS     560
LQCRICI                                                                              640
..........................N...........N.........................................      80
................................................................................     160
....................N...........................N...............................     240
.............................................................N..................     320
................................................................................     400
................................................................................     480
..................N..........................................................N..     560
.......                                                                              640
```

(Threshold=0.5)

SeqName	Position	Potential	Jury agreement	N-Glyc result	
WDV81644.1	26 NNST	0.3686	(9/9)	--	
WDV81644.1	27 NSTE	0.7796	(9/9)	+++	
WDV81644.1	39 NVTV	0.7180	(9/9)	++	
WDV81644.1	181 NNTN	0.6101	(7/9)	+	
WDV81644.1	209 NPTT	0.6337	(8/9)	+	WARNING: PRO-X1.
WDV81644.1	302 NSSM	0.5443	(6/9)	+	
WDV81644.1	499 NGTY	0.5824	(6/9)	+	
WDV81644.1	558 NGSL	0.6827	(9/9)	++	

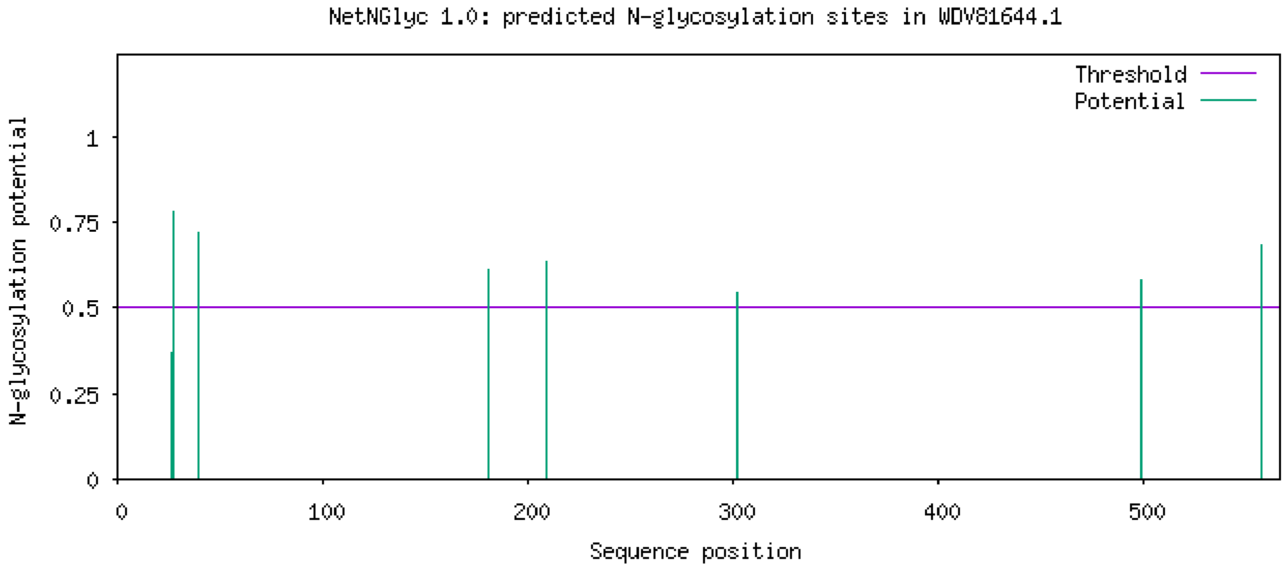

>WDV81646.1 neuraminidase [Influenza A virus/Panthera-leo-/Peru/2023]

```
Name:  WDV81646.1      Length:  469
MNPNQKITTIGSICMVIGIVSLMLQIGNIISIWVSHSIQTGNQYQPEPCNQSIITYENNTWVNQTYVNISNTNFLAEQAV      80
TSVTLAGNSSLCPISGWAIYSKDNGIRIGSKGDVFVIREPFISCSHLECRTFFLTQGALLNDKHSNGTVKDRSPYRTLMS     160
CPVGEAPSPYNSRFESVAWSASACHDGISWLTIGISGPDNGAVAVLKYNGIITDTIKSWRNNILRTQESECACVNGSCFT     240
VMTDGPSNGQASYKIFKIEKGKVVKSVEMNAPNYHYEECSCYPDAGDIMCVCRDNWHGSNRPWVSFNQNLEYQIGYICSG     320
VFGDNPRPNDGPGSCSPMPSNGAYGVKGFSFKYGNGVWIGRTKSTSSRSGFEMIWDPNGWTETDSSFSVKQDIVEITDWS     400
GYSGSFVQHPELTGLDCMRPCFWVELIRGRPKENTIWTSGSSISFCGVNSDTVGWSWPDGAELPFTIDK                480
.................................................N.......N....N....N............      80
.......N.........................................................N..............     160
..........................................................................N.....     240
................................................................................     320
................................................................................     400
.....................................................................                480

(Threshold=0.5)
----------------------------------------------------------------------
SeqName      Position  Potential   Jury    N-Glyc
                                agreement  result
----------------------------------------------------------------------
WDV81646.1    50 NQSI   0.5884     (8/9)   +
WDV81646.1    58 NNTW   0.5496     (6/9)   +
WDV81646.1    63 NQTY   0.6637     (9/9)   ++
WDV81646.1    68 NISN   0.7378     (9/9)   ++
WDV81646.1    88 NSSL   0.7724     (9/9)   +++
WDV81646.1   146 NGTV   0.6876     (9/9)   ++
WDV81646.1   235 NGSC   0.7321     (9/9)   ++
----------------------------------------------------------------------
```

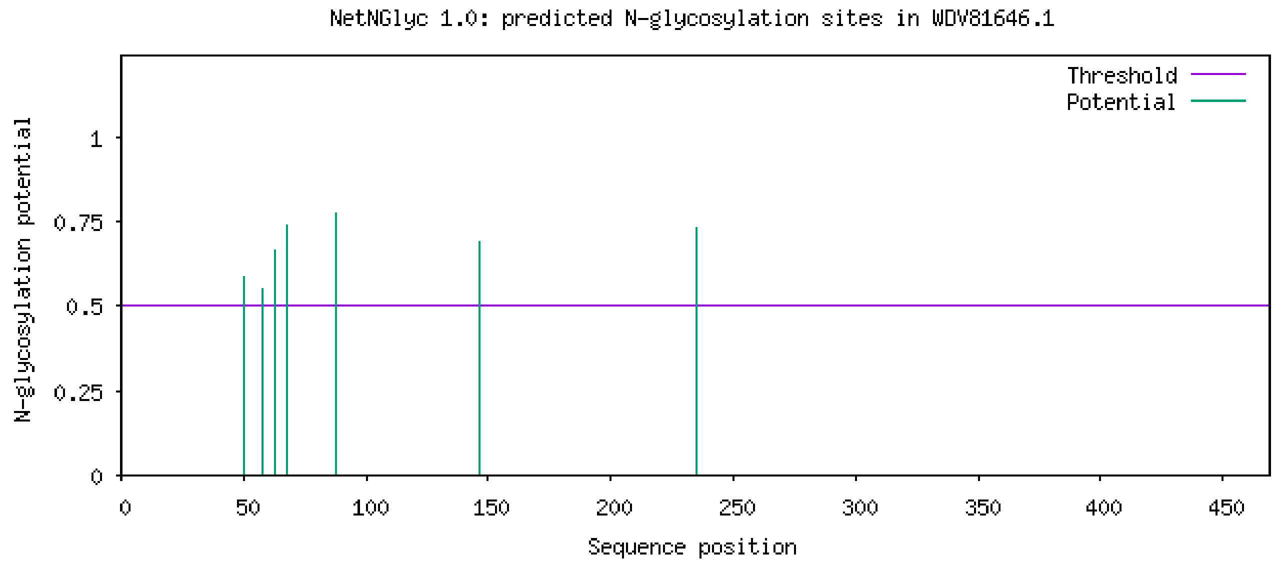

15. >XHO52173.1 hemagglutinin [Influenza A virus/Racoon/2024]

```
Name:  XHO52173.1      Length:  567
MENIVLLLAIVSLVKSDQICIGYHANNSTEQVDTIMEKNVTVTHAQDILEKTHNGKLCDLNGVKPLILKDCSVAGWLLGN      80
PMCDEFIRVPEWSYIVERANPANDLCYPGSLNDYEELKHMLSRINHFEKIQIIPKSSWPNHETSLGVSAACPYQGAPSFF     160
RNVVWLIKKNDAYPTIKISYNNTNREDLLILWGIHHSNNAEEQTNLYKNPITYISVGTSTLNQRLAPKIATRSQVNGQRG     240
RMDFFWTILKPDDAIHFESNGNFIAPEYAYKIVKKGDSTIMKSGVEYGHCNTKCQTPVGAINSSMPFHNIHPLTIGECPK     320
YVKSNKLVLATGLRNSPLREKRRKRGLFGAIAGFIEGGWQGMVDGWYGYHHSNEQGSGYAADKESTQKAIDGVTNKVNSI     400
IDKMNTQFEAVGREFNNLERRIENLNKKMEDGFLDVWTYNAELLVLMENERTLDFHDSNVKNLYDKVRLQLRDNAKELGN     480
GCFEFYHKCDNECMESVRNGTYDYPQYSEEARLKREEISGVKLESVGTYQILSIYSTAASSLALAIMMAGLSLWMCSNGS     560
LQCRICI                                                                              640
.........................N............N.........................................      80
................................................................................     160
....................N...........................................................     240
.............................................................N..................     320
................................................................................     400
................................................................................     480
..................N..........................................................N..     560
.......                                                                              640

(Threshold=0.5)
----------------------------------------------------------------------
SeqName      Position  Potential   Jury    N-Glyc
                                 agreement result
----------------------------------------------------------------------
XHO52173.1     26 NNST   0.3686    (9/9)   --
XHO52173.1     27 NSTE   0.7796    (9/9)   +++
XHO52173.1     39 NVTV   0.7182    (9/9)   ++
XHO52173.1    181 NNTN   0.6100    (7/9)   +
XHO52173.1    302 NSSM   0.5447    (6/9)   +
XHO52173.1    499 NGTY   0.5826    (6/9)   +
XHO52173.1    558 NGSL   0.6827    (9/9)   ++
----------------------------------------------------------------------
```

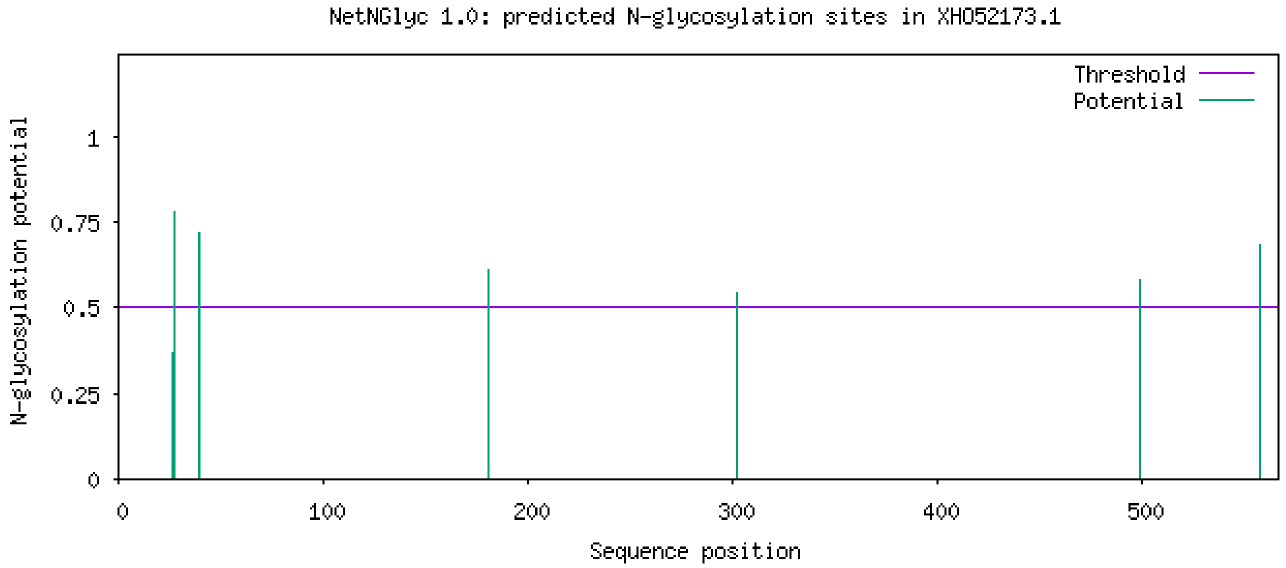

>XHO52175.1 neuraminidase [Influenza A virus/Raccoon/2024]

```
Name:  XHO52175.1        Length:  469
MNPNQKITTIGSICMVIGIVSLMLQIGNIISIWVSHSIQTGNQYQPEPCNQSIITYENNTWVNQTYINISSTNFLAEQAV      80
TSVTLAGNSSLCPISGWAIYSKDNGIRIGSKGDVFVIREPFISCSHLECRTFFLTQGALLNDKHSNGTVKDRSPYRTLMS     160
CPVGEAPSPYNSRFESVAWSASACHDGISWLTIGISGPDNGAVAVLKYNGIITDTIKSWRNNILRTQESECACVNGSCFT     240
VMTDGPSNGQASYKIFKIEKGKVVKSVEMNAPNYHYEECSCYPDAGDIMCVCRDNWHGSNRPWVSFNQNLEYQIGYICSG     320
IFGDNPRPNDGTGSCSPMPSNGAYGVKGFSFKYGNGVWIGRTKSTSSRSGFEMIWDPNGWTETDSSFSVKQDIVEITDWS     400
GYSGSFVQHPELTGLDCMRPCFWVELIRGRPKENTIWTSGSSISFCGVNSDTVGWSWPDGAELPFTIDK                480
.................................................N.......N....N....N............      80
.......N.........................................................N..............     160
..........................................................................N.....     240
................................................................................     320
................................................................................     400
.....................................................................                480

(Threshold=0.5)
----------------------------------------------------------------------
SeqName      Position  Potential   Jury    N-Glyc
                                 agreement result
----------------------------------------------------------------------
XHO52175.1     50 NQSI   0.5883     (8/9)   +
XHO52175.1     58 NNTW   0.5251     (5/9)   +
XHO52175.1     63 NQTY   0.6874     (9/9)   ++
XHO52175.1     68 NISS   0.7140     (9/9)   ++
XHO52175.1     88 NSSL   0.7724     (9/9)   +++
XHO52175.1    146 NGTV   0.6873     (9/9)   ++
XHO52175.1    235 NGSC   0.7321     (9/9)   ++
----------------------------------------------------------------------
```

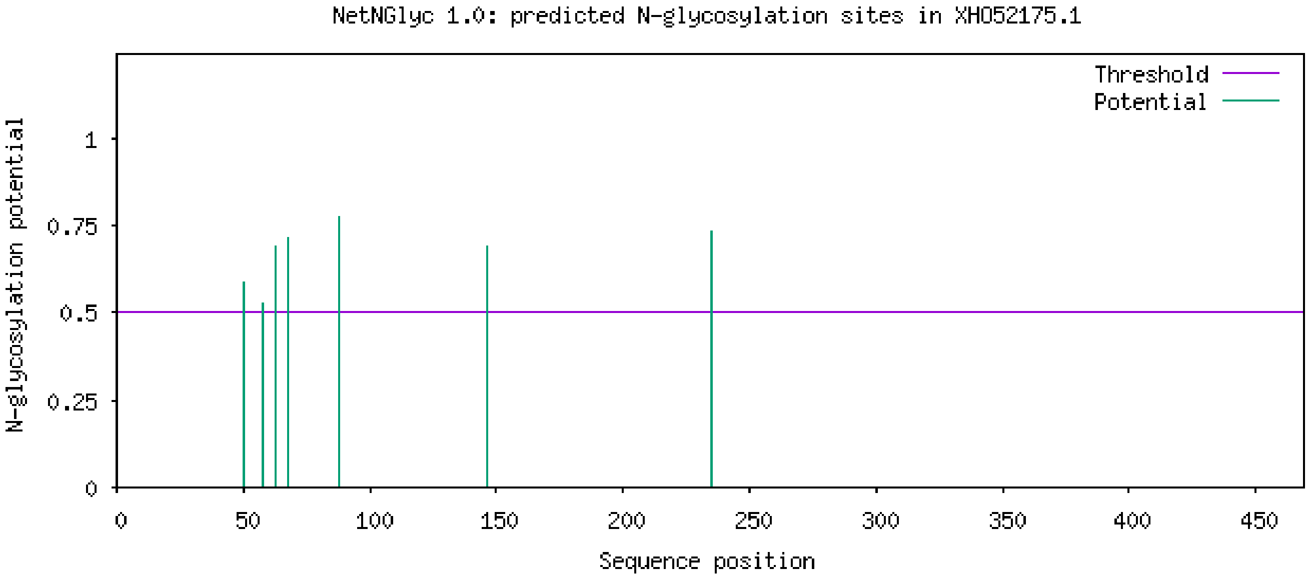

16. >WXB47616.1 hemagglutinin [Influenza A virus/royal-tern/Argentina/CH-PD036/2023]

```
Name:  WXB47616.1       Length:  567
MENIVLLLAIVSLVKSDQICIGYHANNSTEQVDTIMEKNVTVTHAQDILEKTHNGKLCDLNGVKPLILKDCSVAGWLLGN      80
PMCDEFIRVPEWSYIVERANPANDLCYPGSLNDYEELKHMLSRINHFEKIQIIPKSSWPNHETSLGVSAACPYQGAPSFF     160
RNVVWLIKKNDAYPTIKISYNNTNREDLLILWGIHHSNNAEEQTNLYKNPTTYISVGTSTLNQRLAPKIATRSQVNGQRG     240
RMDFFWTILKPDDAIHFESNGNFIAPEYAYKIVKKGDSTIMKSGVEYGHCNTKCQTPVGAINSSMPFHNIHPLTIGECPK     320
YVKSNKLVLATGLRNSPLREKRRKRGLFGAIAGFIEGGWQGMVDGWYGYHHSNEQGSGYAADKESTQKAIDGVTNKVNSI     400
IDKMNTQFEAVGREFNNLERRIENLNKKMEDGFLDVWTYNAELLVLMENERTLDFHDSNVKNLYDKVRLQLRDNAKELGN     480
GCFEFYHKCDNECMESVRNGTYDYPQYSEEARLKREEISGVKLESVGTYQILSIYSTAASSLALAIMMAGLSLWMCSNGS     560
LQCRICI                                                                              640
..........................N...........N.........................................      80
................................................................................     160
....................N...........................N...............................     240
.............................................................N..................     320
................................................................................     400
................................................................................     480
..................N..........................................................N..     560
.......                                                                              640

(Threshold=0.5)
----------------------------------------------------------------------
SeqName      Position  Potential   Jury    N-Glyc
                                agreement  result
----------------------------------------------------------------------
WXB47616.1    26 NNST   0.3686     (9/9)   --
WXB47616.1    27 NSTE   0.7797     (9/9)   +++
WXB47616.1    39 NVTV   0.7182     (9/9)   ++
WXB47616.1   181 NNTN   0.6100     (7/9)   +
WXB47616.1   209 NPTT   0.6336     (8/9)   +     WARNING: PRO-X1.
WXB47616.1   302 NSSM   0.5447     (6/9)   +
WXB47616.1   499 NGTY   0.5824     (6/9)   +
WXB47616.1   558 NGSL   0.6827     (9/9)   ++
----------------------------------------------------------------------
```

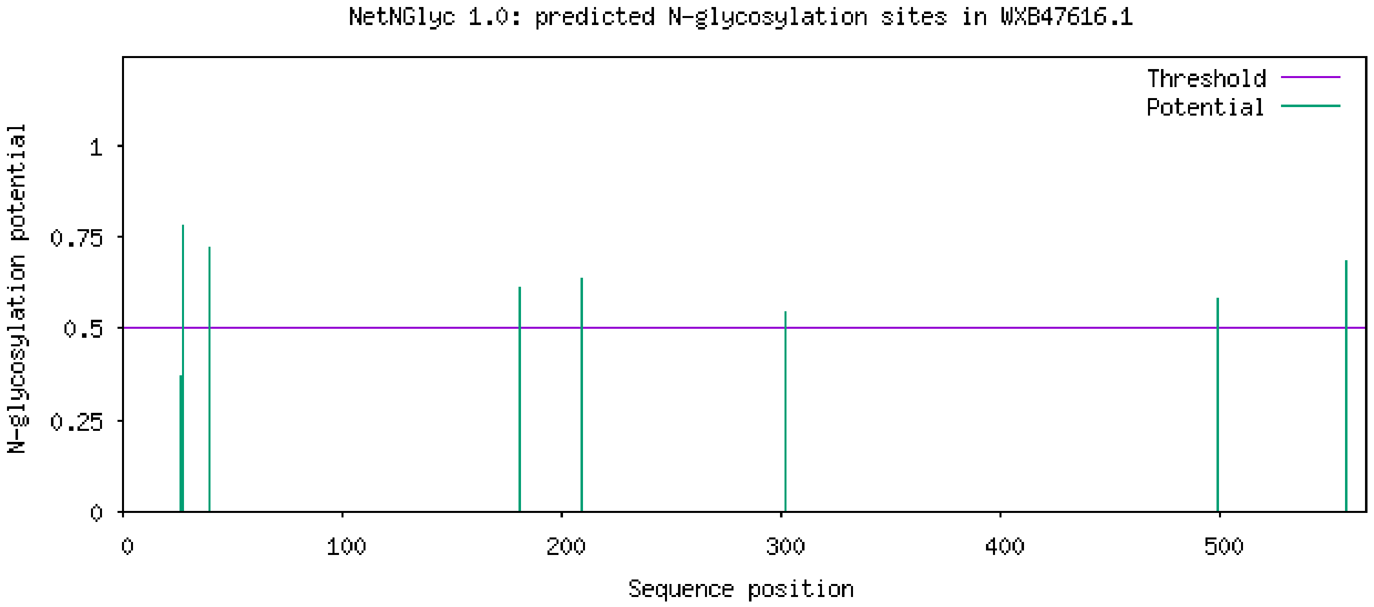

>WXB47618.1 neuraminidase [Influenza A virus/Royal-tern/Argentina/2023]

```
Name:  WXB47618.1       Length:  469
MNPNQKITTIGSICMVIGIVSLMLQIGNIISIWVSHSIQTGNQYQPEPCNQSIITYENNTWVNQTYVNISNTNFLAEQAV      80
TSVTLAGNSSLCPISGWAIYSKDNGIRIGSKGDVFVIREPFISCSHLECRTFFLTQGALLNDKHSNGTVKDRSPYRTLMS     160
CPVGEAPSPYNSRFESVAWSASACHDGISWLTIGISGPDNGAVAVLKYNGIITDTIKSWRNNILRTQESECACVNGSCFT     240
VMTDGPSNGQASYKIFKIEKGKVVKSVEMNAPNYHYEECSCYPDAGDIMCVCRDNWHGSNRPWVSFNQNLEYQIGYICSG     320
VFGDNPRPNDGTGSCSPMPSNGAYGVKGFSFKYGNGVWIGRTKSTSSRSGFEMIWDPNGWTETDSSFSVKQDIVEITDWS     400
GYSGSFVQHPELTGLDCMRPCFWVELIRGRPKENTIWTSGSSISFCGVNSDTVGWSWPDGAELPFTIDK                480
.................................................N.......N....N....N............      80
.......N.........................................................N..............     160
..........................................................................N.....     240
................................................................................     320
................................................................................     400
.....................................................................                480

(Threshold=0.5)
----------------------------------------------------------------------
SeqName      Position  Potential   Jury    N-Glyc
                                 agreement result
----------------------------------------------------------------------
WXB47618.1    50 NQSI   0.5884     (8/9)   +
WXB47618.1    58 NNTW   0.5496     (6/9)   +
WXB47618.1    63 NQTY   0.6636     (9/9)   ++
WXB47618.1    68 NISN   0.7378     (9/9)   ++
WXB47618.1    88 NSSL   0.7724     (9/9)   +++
WXB47618.1   146 NGTV   0.6876     (9/9)   ++
WXB47618.1   235 NGSC   0.7321     (9/9)   ++
----------------------------------------------------------------------
```

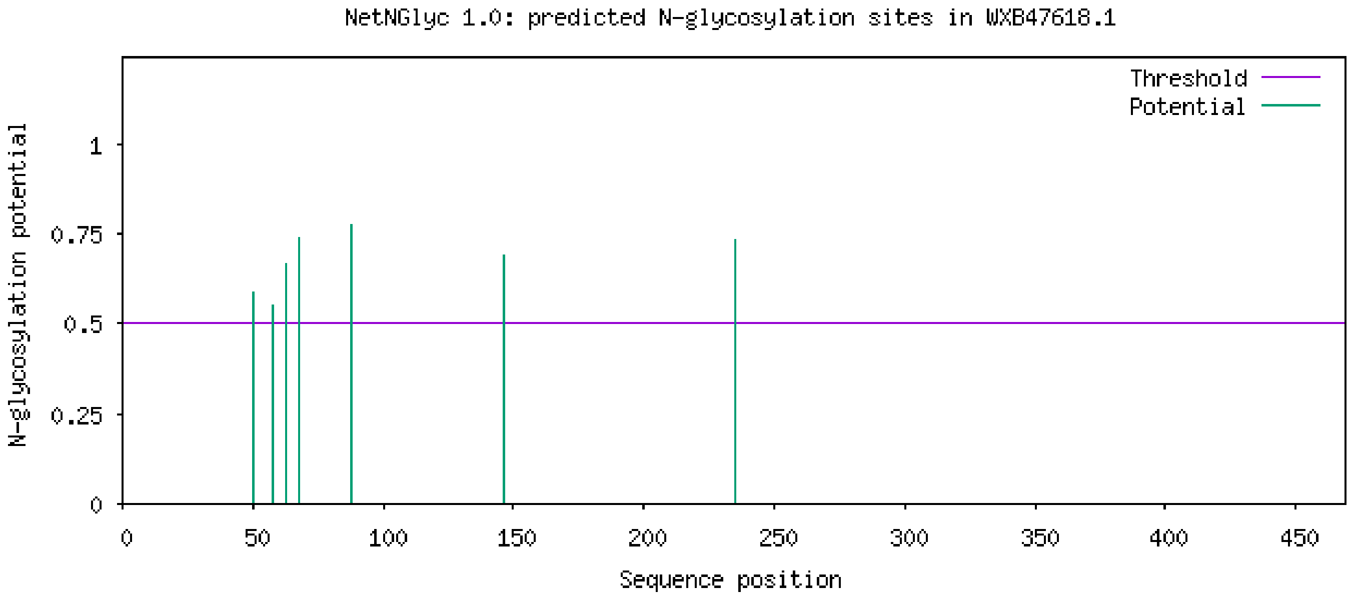

17. >WEU54184.1 hemagglutinin [Influenza A virus/wild-duck/Colombia/Choco/3501/2022]

```
Name:  WEU54184.1      Length:  567
MENIVLLLAIVSLVKSDQICIGYHANNSTEQVDTIMEKNVTVTHAQDILEKTHNGKLCDLNGVKPLILKDCSVAGWLLGN      80
PMCDEFIRVPEWSYIVERANPANDLCYPGSLNDYEELKHMLSRINHFEKILIIPKSSWPNHETSLGVSAACPYQGAPSFF     160
RNVVWLIKKNDAYPTIRISYNNTNREDLLILWGIHHSNSAEEQTNLYKNPTTYISVGTSTLNQRLAPKIATRSQVNGQRG     240
RMDFFWTILKPDDAIHFESNGNFIAPEYAYKIVKKGDSTIMKSGVEYGHCNTKCQTPVGAINSSMPFHNIHPLTIGECPK     320
YVKSNKLVLATGLRNSPLREKRRKRGLFGAIAGFIEGGWQGMVDGWYGYHHSNEQGSGYAADKESTQKAIDGVTNKVNSI     400
IDKMNTQFEAVGREFNNLERRIENLNKKMEDGFLDVWTYNAELLVLMENERTLDFHDSNVKNLYDKVRLQLRDNAKELGN     480
GCFEFYHKCDNECMESVRNGTYDYPQYSEEARLKREEISGVKLESVGTYQILSIYSTAASSLALAIMMAGLSLWMCSNGS     560
LQCRICI                                                                              640
.........................N............N.........................................      80
................................................................................     160
....................N...........................N...............................     240
.............................................................N..................     320
................................................................................     400
................................................................................     480
..................N..........................................................N..     560
.......                                                                              640

(Threshold=0.5)
```

SeqName	Position	Potential	Jury agreement	N-Glyc result	
WEU54184.1	26 NNST	0.3687	(9/9)	--	
WEU54184.1	27 NSTE	0.7796	(9/9)	+++	
WEU54184.1	39 NVTV	0.7181	(9/9)	++	
WEU54184.1	181 NNTN	0.6126	(7/9)	+	
WEU54184.1	209 NPTT	0.6292	(8/9)	+	WARNING: PRO-X1.
WEU54184.1	302 NSSM	0.5447	(6/9)	+	
WEU54184.1	499 NGTY	0.5824	(6/9)	+	
WEU54184.1	558 NGSL	0.6828	(9/9)	++	

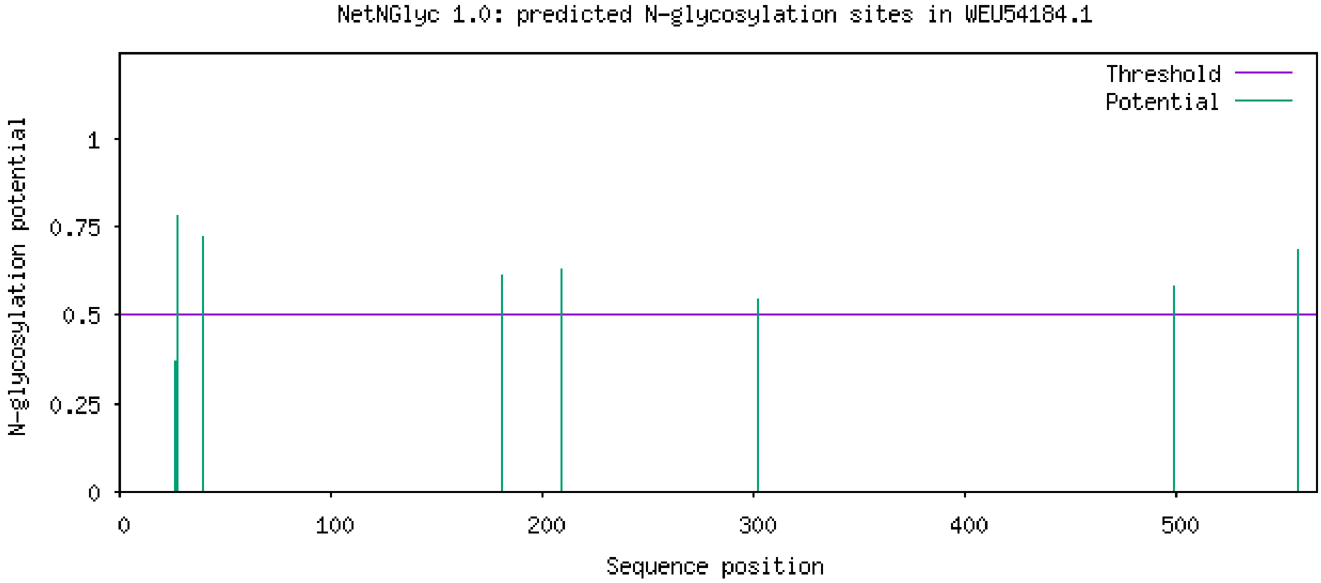

>WEU54186.1 neuraminidase [Influenza A virus/wild-duck/Colombia/Choco/3501/2022]

```
Name:  WEU54186.1       Length:  469
MNPNQKITTIGSICMVIGIVSLMLQIGNIISIWVSHSIRTGNQYQPEPCNQSIITYENNTWVNQTYVNINNTNFLAEQAV      80
TSVTLAGNSSLCPISGWAIYSKDNGIRIGSKGDVFVIREPFISCSHLECRTFFLTQGALLNDKHSNGTVKDRSPYRTLMS     160
CPVGEAPSPYNSRFESVAWSASACHDGISWLTIGISGPDNGAVAVLKYNGIITDTIKSWRNNILRTQESECACVNGSCFT     240
VMTDGPSNGQASSKIFKIEKGKVVKSVELNAPNYHYEECSCYPDAGDIMCVCRDNWHGSNRPWVSFNQNLEYQIGYICSG     320
VFGDNPRPNDGTGSCSPMSSNGAYGIKGFSFKYGNGVWIGRTKSTSSRSGFEMIWDPNGWTETDSSFSVKQDIVEITDWS     400
GYSGSFVQHPELTGLDCMRPCFWVELIRGRPKENTIWTSGSSISFCGVNSDTVGWSWPDGGRFARWSAE                480
.................................................N.......N....N......N..........      80
.......N.........................................................N..............     160
..........................................................................N.....     240
................................................................................     320
................................................................................     400
.....................................................................                480

(Threshold=0.5)
----------------------------------------------------------------------
SeqName      Position  Potential   Jury    N-Glyc
                                 agreement result
----------------------------------------------------------------------
WEU54186.1    50 NQSI   0.5881     (8/9)   +
WEU54186.1    58 NNTW   0.5496     (6/9)   +
WEU54186.1    63 NQTY   0.6564     (9/9)   ++
WEU54186.1    70 NNTN   0.6887     (8/9)   +
WEU54186.1    88 NSSL   0.7724     (9/9)   +++
WEU54186.1   146 NGTV   0.6872     (9/9)   ++
WEU54186.1   235 NGSC   0.7322     (9/9)   ++
----------------------------------------------------------------------
```

NetNGlyc 1.0: predicted N-glycosylation sites in WEU54186.1

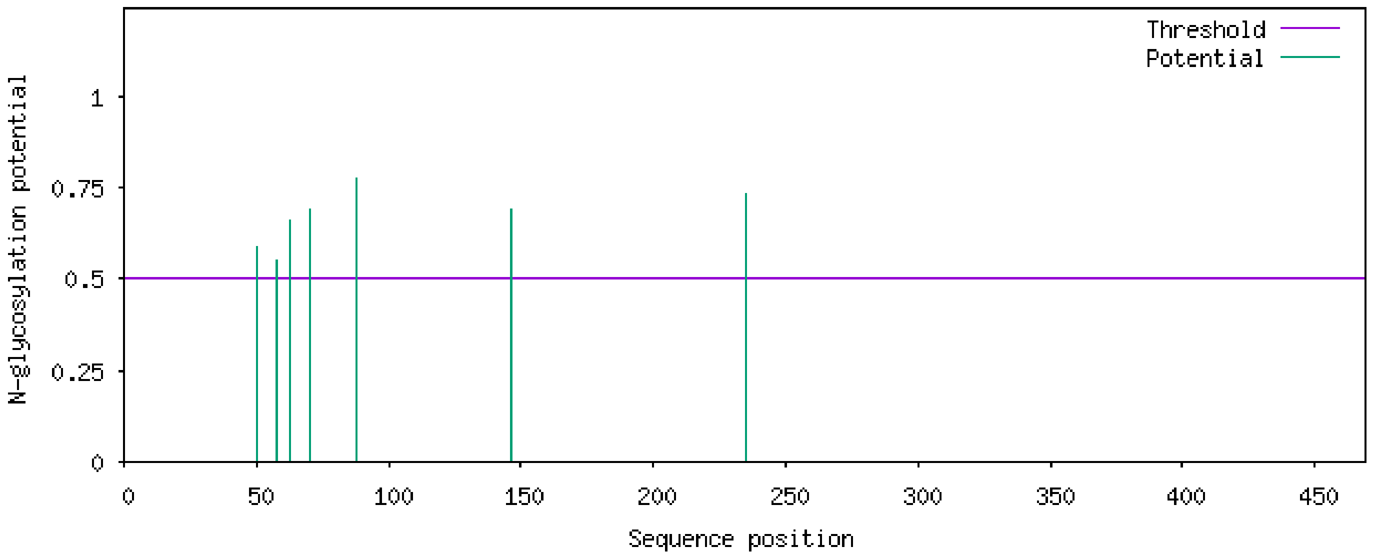

18. >WLC96969.1 hemagglutinin [Influenza A virus/Black-necked-swan/Uruguay/078-M2/2023]

```
Name:  WLC96969.1       Length:  567
MENIVLLLAIVSLVKSDQICIGYHANNSTEQVDTIMEKNVTVTHAQDILEKTHNGKLCDLNGVKPLILKDCSVAGWLLGN      80
PMCDEFIRVPEWSYIVERANPANDLCYPGSLNDYEELKHMLSRINHFEKIQIIPKSSWPNHETSLGVSAACPYQGAPSFF     160
RNVVWLIKKNDAYPTIKISYNNTNREDLLILWGIHHSNNAEEQTNLYKNPTTYISVGTSTLNQRLAPKIATRSQVNGQRG     240
RMDFFWTILKPDDAIHFESNGNFIAPEYAYKIVKKGDSTIMKSGVEYGHCNTKCQTPVGAINSSMPFHNIHPLTIGECPK     320
YVKSNKLVLATGLRNSPLREKRRKRGLFGAIAGFIEGGWQGMVDGWYGYHHSNEQGSGYAADKESTQKAIDGVTNKVNSI     400
IDKMNTQFEAVGREFNNLERRIENLNKKMEDGFLDVWTYNAELLVLMENERTLDFHDSNVKNLYDKVRLQLRDNAKELGN     480
GCFEFYHKCDNECMESVRNGTYDYPQYSEEARLKREEISGVKLESVGTYQILSIYSTAASSLALAIMMAGLSLWMCSNGS     560
LQCRICI                                                                              640
..........................N...........N.........................................      80
................................................................................     160
....................N...........................N...............................     240
.............................................................N..................     320
................................................................................     400
................................................................................     480
..................N..........................................................N..     560
.......                                                                              640

(Threshold=0.5)
----------------------------------------------------------------------
SeqName      Position  Potential   Jury    N-Glyc
                                 agreement result
----------------------------------------------------------------------
WLC96969.1    26 NNST  0.3686     (9/9)    --
WLC96969.1    27 NSTE  0.7797     (9/9)    +++
WLC96969.1    39 NVTV  0.7182     (9/9)    ++
WLC96969.1   181 NNTN  0.6100     (7/9)    +
WLC96969.1   209 NPTT  0.6336     (8/9)    +     WARNING: PRO-X1.
WLC96969.1   302 NSSM  0.5447     (6/9)    +
WLC96969.1   499 NGTY  0.5824     (6/9)    +
WLC96969.1   558 NGSL  0.6827     (9/9)    ++
----------------------------------------------------------------------
```

NetNGlyc 1.0: predicted N-glycosylation sites in WLC96969.1

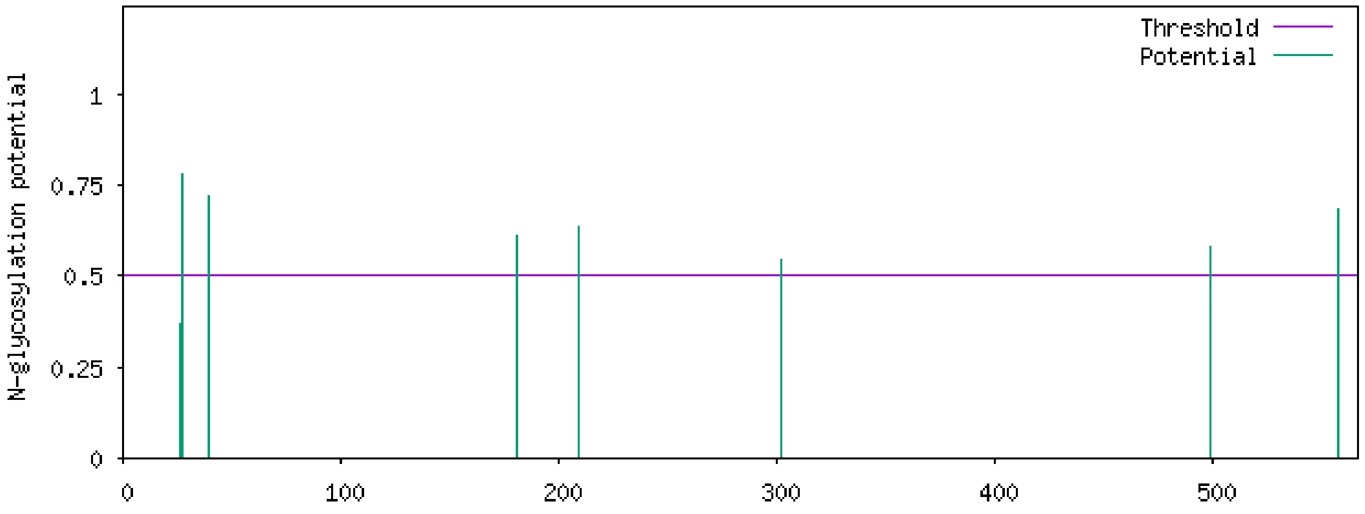

>WLC96967.1 neuraminidase [Influenza A virus/Black-necked-swan/Uruguay/078-M2/2023]

```
Name:  WLC96967.1      Length:  469
MNPNQKITTIGSICMVIGIVSLMLQIGNIISIWVSHSIQTGNQYQPEPCNQSIITYENNTWVNQTYVNISNTNFLAEQAV      80
TSVTLAGNSSLCPISGWAIYSKDNGIRIGSKGDVFVIREPFISCSHLECRTFFLTQGALLNDKHSNGTVKDRSPYRTLMS     160
CPVGEAPSPYNSRFESVAWSASACHDGISWLTIGISGPDNGAVAVLKYNGIITDTIKSWRNNILRTQESECACVNGSCFT     240
VMTDGPSNGQASYKIFKIEKGKVVKSVEMNAPNYHYEECSCYPNAGDIMCVCRDNWHGSNRPWVSFNQNLEYQIGYICSG     320
VFGDNPRPNDGTGSCSPMPSNGAYGVKGFSFKYGNGVWIGRTKSTSSRSGFEMIWDPNGWTETDSSFSVKQDIVEITDWS     400
GYSGSFVQHPELTGLDCMRPCFWVELIRGRPKENTIWTSGSSISFCGVNSDTVGWSWPDGAELPFTIDK                480
.................................................N.......N....N....N............      80
.......N.........................................................N..............     160
..........................................................................N.....     240
................................................................................     320
................................................................................     400
.....................................................................                480
```

(Threshold=0.5)

SeqName	Position		Potential	Jury agreement	N-Glyc result
WLC96967.1	50	NQSI	0.5884	(8/9)	+
WLC96967.1	58	NNTW	0.5496	(6/9)	+
WLC96967.1	63	NQTY	0.6636	(9/9)	++
WLC96967.1	68	NISN	0.7378	(9/9)	++
WLC96967.1	88	NSSL	0.7724	(9/9)	+++
WLC96967.1	146	NGTV	0.6873	(9/9)	++
WLC96967.1	235	NGSC	0.7320	(9/9)	++

NetNGlyc 1.0: predicted N-glycosylation sites in WLC96967.1

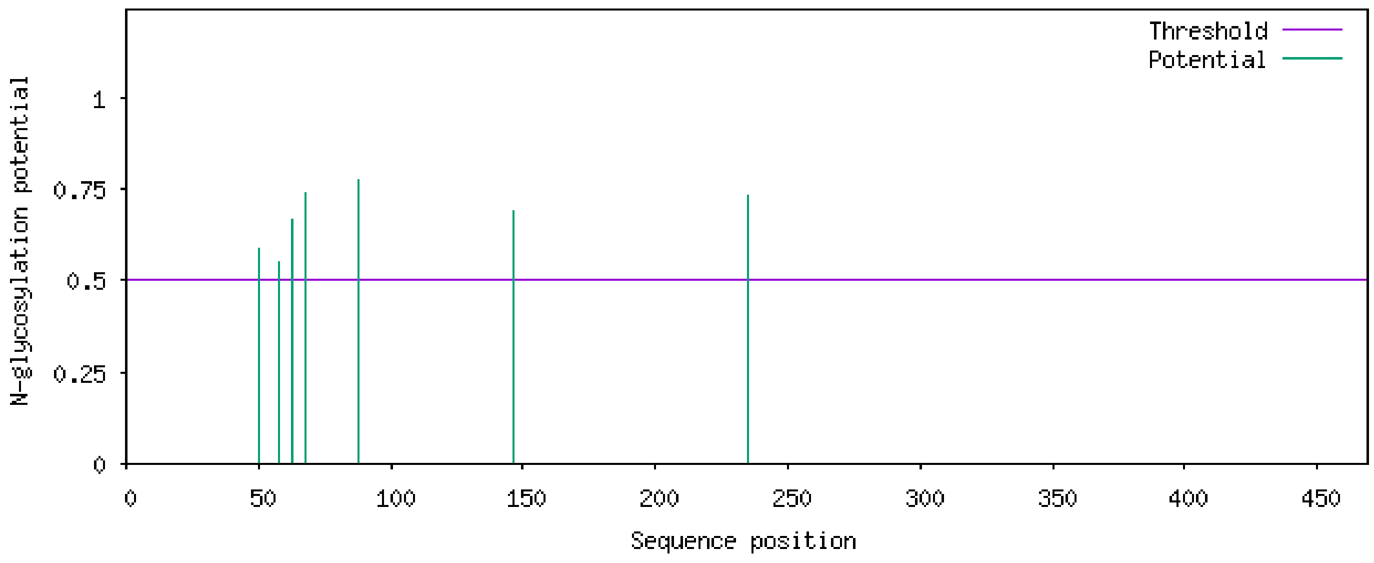

19. >WPS70011.1 hemagglutinin [Influenza A virus/Chimango-caracara/Coquimbo/242326-1/2023]

```
Name:  WPS70011.1       Length:  567
MENIVLLLAIVSLVKSDQICIGYHANNSTEQVDTIMEKNVTVTHAQDILEKTHNGKLCDLNGVKPLILKDCSVAGWLLGN      80
PMCDEFIRVPEWSYIVERANPANDLCYPGSLNDYEELKHMLSRINHFEKIQIIPKSSWPNHETSLGVSAACPYQGAPSFF     160
RNVVWLIKKNDAYPTIKISYNNTNREDLLILWGIHHSNNAKEQTNLYKNPTTYISVGTSTLNQRLAPKIATRSQVNGQRG     240
RMDFFWTILKPDDAIHFESNGNFIAPEYAYKIVKKGDSTIMKSGVEYGHCNTKCQTPVGAINSSMPFHNIHPLTIGECPK     320
YVKSNKLVLATGLRNSPLREKRRKRGLFGAIAGFIEGGWQGMVDGWYGYHHSNEQGSGYAADKESTQKAIDGVTNKVNSI     400
IDKMNTQFEAVGREFNNLERRIENLNKKMEDGFLDVWTYNAELLVLMENERTLDFHDSNVKNLYDKVRLQLRDNAKELGN     480
GCFEFYHKCDNECMESVRNGTYDYPQYSEEARLKREEISGVKLESVGTYQILSIYSTAASSLALAIMMAGLSLWMCSNGS     560
LQCRICI                                                                              640
.........................N............N.........................................      80
................................................................................     160
....................N...........................N...............................     240
.............................................................N..................     320
................................................................................     400
................................................................................     480
..................N..........................................................N..     560
.......                                                                              640

(Threshold=0.5)
----------------------------------------------------------------------
SeqName      Position  Potential   Jury    N-Glyc
                                agreement  result
----------------------------------------------------------------------
WPS70011.1     26 NNST   0.3687     (9/9)   --
WPS70011.1     27 NSTE   0.7796     (9/9)   +++
WPS70011.1     39 NVTV   0.7180     (9/9)   ++
WPS70011.1    181 NNTN   0.6100     (7/9)   +
WPS70011.1    209 NPTT   0.6378     (8/9)   +    WARNING: PRO-X1.
WPS70011.1    302 NSSM   0.5446     (6/9)   +
WPS70011.1    499 NGTY   0.5824     (6/9)   +
WPS70011.1    558 NGSL   0.6826     (9/9)   ++
----------------------------------------------------------------------
```

NetNGlyc 1.0: predicted N-glycosylation sites in WPS70011.1

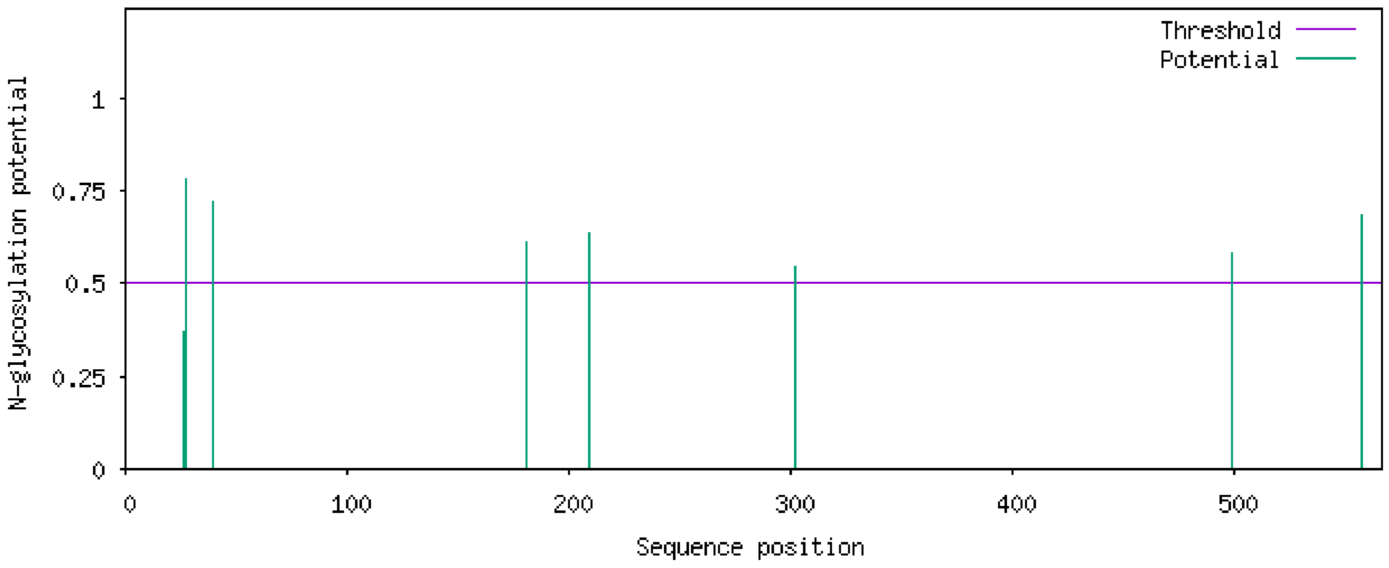

>WPS70013.1 neuraminidase [Influenza A virusA/Chimango-caracara/Coquimbo/242326-1/2023]

```
Name:  WPS70013.1      Length:  469
MNPNQKITTIGSICMVIGIVSLMLQIGNIISIWVSHSIQTGNQYQPEPCNQSIITYENNTWVNQTYVNISNTNFLAEQAV      80
TSVTLAGNSSLCPISGWAIYSKDNGIRIGSKGDVFVIREPFISCSHLECRTFFLTQGALLNDKHSNGTVKDRSPYRTLMS     160
CPVGEAPSPYNSRFESVAWSASACHDGISWLTIGISGPDNGAVAVLKYNGIITDTIKSWRNNILRTQESECACVNGSCFT     240
VMTDGPSNGQASYKIFKIEKGKVVKSVEMNAPNYHYEECSCYPDAGDIMCVCRDNWHGSNRPWVSFNQNLEYQIGYICSG     320
VFGDNPRPNDGTGSCSPMPSNGAYGVKGFSFKYGNGVWIGRTKSTSSRSGFEMIWDPNGWTETDSSFSVKQDIVEITDWS     400
GYSGSFVQHPELTGLDCMRPCFWVELIRGRPKENTIWTSGSSISFCGVNSDTVGWSWPDGAELPFTIDK                480
.................................................N.......N....N....N............      80
.......N.........................................................N..............     160
..........................................................................N.....     240
................................................................................     320
................................................................................     400
.....................................................................                480

(Threshold=0.5)
----------------------------------------------------------------------
SeqName      Position  Potential   Jury    N-Glyc
                                 agreement result
----------------------------------------------------------------------
WPS70013.1    50 NQSI   0.5884     (8/9)   +
WPS70013.1    58 NNTW   0.5496     (6/9)   +
WPS70013.1    63 NQTY   0.6636     (9/9)   ++
WPS70013.1    68 NISN   0.7378     (9/9)   ++
WPS70013.1    88 NSSL   0.7724     (9/9)   +++
WPS70013.1   146 NGTV   0.6876     (9/9)   ++
WPS70013.1   235 NGSC   0.7321     (9/9)   ++
----------------------------------------------------------------------
```

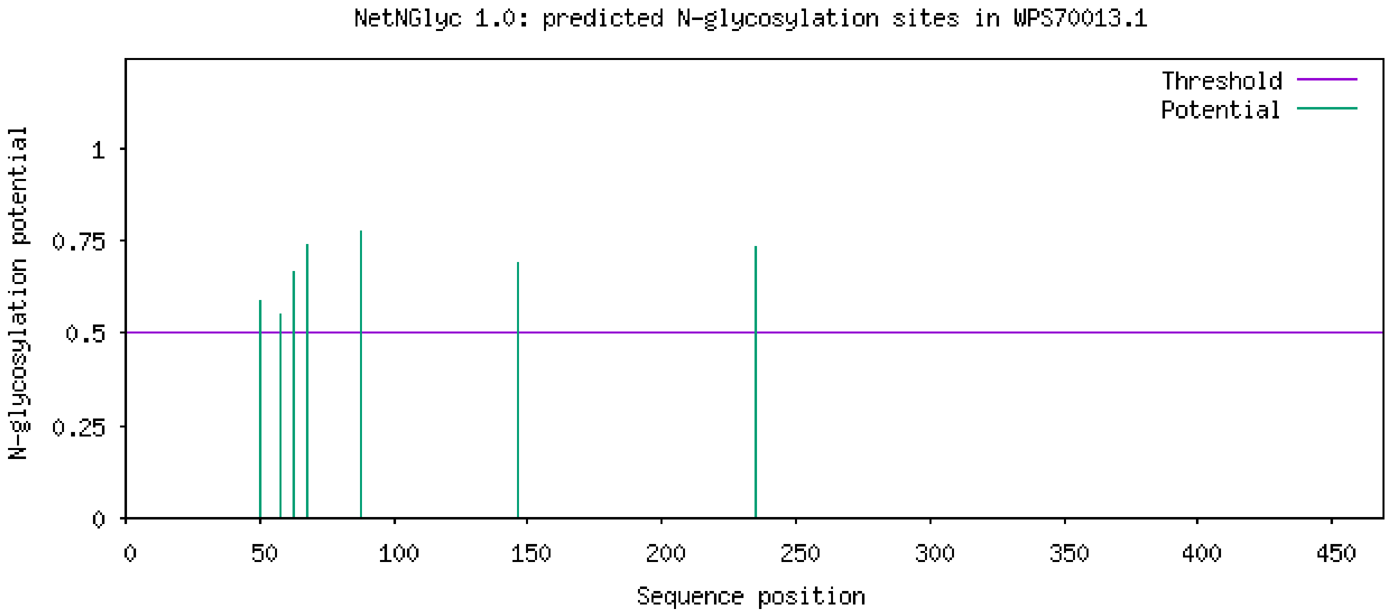

20. >WPF49852.1 hemagglutinin [Influenza A virus/peregrine-falcon/NewYork/NYCVH-160820/2022]

```
Name:  WPF49852.1      Length:  567
MENIVLLLAIVSLVKSDQICIGYHANNSTEQVDTIMEKNVTVTHAQDILEKTHNGKLCDLNGVKPLILKDCSVAGWLLGN      80
PMCDEFIRVPEWSYIVERANPANDLCYPGSLNDYEELKHMLSRINHFEKILIIPKSSWPNHETSLGVSAACPYQGAPSFF     160
RNVVWLIKKNDAYPTIKISYNNTNREDLLILWGIHHSNNAEEQTNLYKNPTTYISVGTSTLNQRLAPKIATRSQVNGQRG     240
RMDFFWTILKPDDAIHFESNGNFIAPEYAYKIVKKGDSTIMKSGVEYGHCNTKCQTPVGAINSSMPFHNIHPLTIGECPK     320
YVKSNKLVLATGLRNSPLREKRRKRGLFGAIAGFIEGGWQGMVDGWYGYHHSNEQGSGYAADKESTQKAIDGVTNKVNSI     400
IDKMNTQFEAVGREFNNLERRIENLNKKMEDGFLDVWTYNAELLVLMENERTLDFHDSNVKNLYDKVRLQLRDNAKELGN     480
GCFEFYHKCDNECMESVRNGTYDYPQYSEEARLKREEISGVKLESVGTYQILSIYSTAASSLALAIMMAGLSLWMCSNGS     560
LQCRICI                                                                              640
..........................N...........N.........................................      80
................................................................................     160
....................N...........................N...............................     240
.............................................................N..................     320
................................................................................     400
................................................................................     480
..................N..........................................................N..     560
.......                                                                              640

(Threshold=0.5)
----------------------------------------------------------------------
SeqName      Position  Potential   Jury     N-Glyc
                                agreement  result
----------------------------------------------------------------------
WPF49852.1    26 NNST   0.3687    (9/9)     --
WPF49852.1    27 NSTE   0.7797    (9/9)     +++
WPF49852.1    39 NVTV   0.7182    (9/9)     ++
WPF49852.1   181 NNTN   0.6100    (7/9)     +
WPF49852.1   209 NPTT   0.6336    (8/9)     +     WARNING: PRO-X1.
WPF49852.1   302 NSSM   0.5447    (6/9)     +
WPF49852.1   499 NGTY   0.5824    (6/9)     +
WPF49852.1   558 NGSL   0.6828    (9/9)     ++
----------------------------------------------------------------------
```

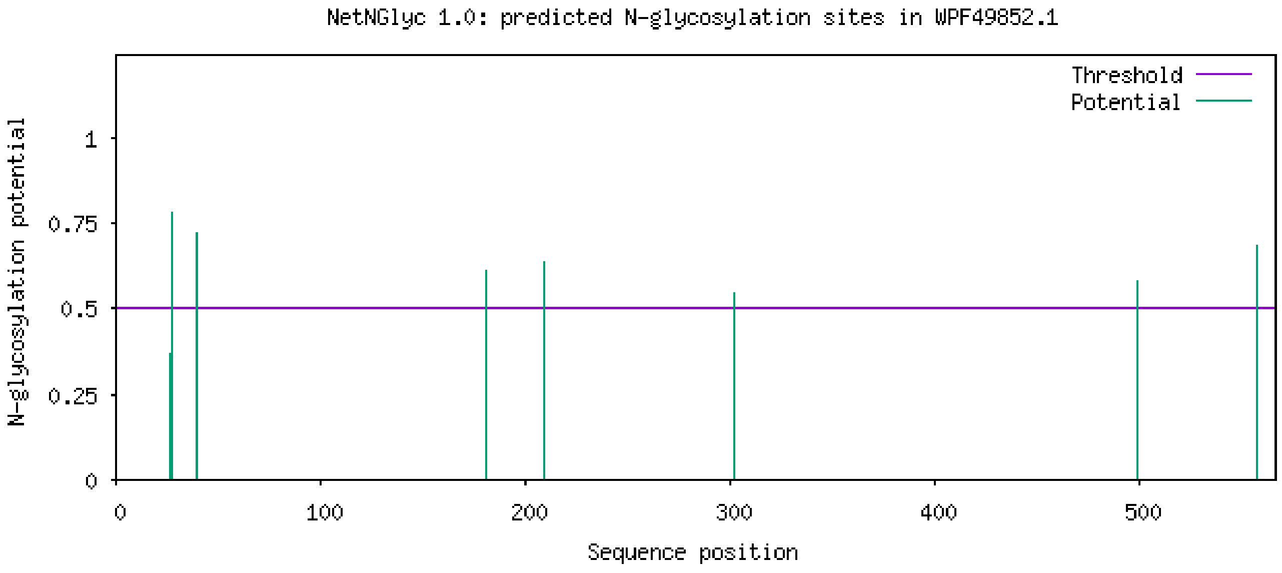

>WPF49853.1 neuraminidase,[Influenza A virus/A/peregrine-falcon/NewYork/NYCVH-160820/2022]

```
Name:  WPF49853.1       Length:  469
MNPNQKITTIGSICMVIGIVSLMLQIGNIISIWVSHSIRTGNQYQPEPCNQSIITYENNTWVNQTYVNISNTNFLAEQAV      80
TSVTLAGNSSLCPISGWAIYSKDNGIRIGSKGDVFVIREPFISCSHLECRTFFLTQGALLNDKHSNGTVKDRSPYRTLMS     160
CPVGEAPSPYNSRFESVAWSASACHDGISWLTIGISGPDNGAVAVLKYNGIITDTIKSWRNNTLRTQESECACVNGFCFT     240
VMTDGPSNGQASYKIFKIEKGKVVKSVELNAPNYHYEECSCYPDAGDIMCVCRDNWHGSNRPWVSFNQNLEYQIGYICSG     320
VFGDNPRPNDGTGSCSPMSSNGAYGVKGFSFKYGNGVWIGRTKSTSSRSGFEMIWDPNGWTETDSSFSVKQDIVEITDWS     400
GYSGSFVQHPELTGLDCMRPCFWVELIRGRPKENTIWTSGSSISFCGVNSDTVGWSWPDGAELPFTIDK                480
.................................................N.......N....N....N............      80
.......N.........................................................N..............     160
................................................................................     240
................................................................................     320
................................................................................     400
.....................................................................                480
```

(Threshold=0.5)

SeqName	Position	Potential	Jury agreement	N-Glyc result
WPF49853.1	50 NQSI	0.5886	(8/9)	+
WPF49853.1	58 NNTW	0.5497	(6/9)	+
WPF49853.1	63 NQTY	0.6634	(9/9)	++
WPF49853.1	68 NISN	0.7378	(9/9)	++
WPF49853.1	88 NSSL	0.7724	(9/9)	+++
WPF49853.1	146 NGTV	0.6872	(9/9)	++
WPF49853.1	221 NNTL	0.4778	(3/9)	-

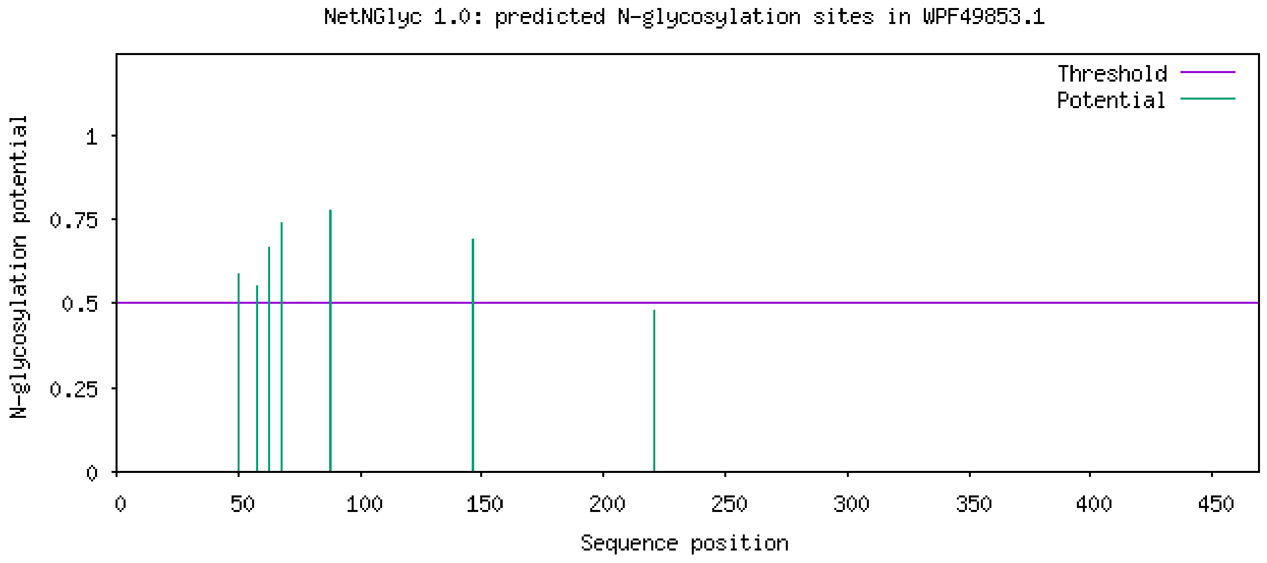

21. >WHA25435.1 hemagglutinin [Influenza A virus/Vulture/Florida/22-012327-003/2022]

```
Name:  WHA25435.1       Length:  567
MENIVLLLAIVSLVKSDQICIGYHANNSTEQVDTIMEKNVTVTHAQDILEKTHNGKLCDLNGVKPLILKDCSVAGWLLGN      80
PMCDEFTRVPEWSYIVERANPTNDLCYPGSLNDYEELKHMLSRINHFEKILIIPKSSWPNHETSLGVSAACPYQGAPSFF     160
RNVVWLIKKNDAYPTIKISYNNTNREDLLILWGIHHSNNAEEQTNLYKNPTTYISVGTSTLNQRLAPKIATRSQVNGQRG     240
RMDFFWTILKPDDAIHFESNGNFIAPEYAYKIVKKGDSTIMKSGVEYGHCNTKCQTPVGAINSSMPFHNIHPLTIGECPK     320
YVKSNKLVLATGLRNSPLREKRRKRGLFGAIAGFIEGGWQGMVDGWYGYHHSNEQGSGYAADKESTQKAIDGVTNKVNSI     400
IDKMNTQFEAVGREFNNLERRIENLNKKMEDGFLDVWTYNAELLVLMENERTLDFHDSNVKNLYDKVRLQLRDNAKELGN     480
GCFEFYHKCDNECMESVRNGTYDYPQYSEEARLKREEISGVKLESVGTYQILSIYSTAASSLALAIMMAGLSLWMCSNGS     560
LQCRICI                                                                              640
..........................N...........N.........................................      80
...................N............................................................     160
....................N...........................N...............................     240
.............................................................N..................     320
................................................................................     400
................................................................................     480
..................N..........................................................N..     560
.......                                                                              640

(Threshold=0.5)
----------------------------------------------------------------------
SeqName      Position  Potential   Jury    N-Glyc
                                 agreement result
----------------------------------------------------------------------
WHA25435.1     26 NNST   0.3687     (9/9)   --
WHA25435.1     27 NSTE   0.7796     (9/9)   +++
WHA25435.1     39 NVTV   0.7179     (9/9)   ++
WHA25435.1    100 NPTN   0.5848     (7/9)   +     WARNING: PRO-X1.
WHA25435.1    181 NNTN   0.6099     (7/9)   +
WHA25435.1    209 NPTT   0.6337     (8/9)   +     WARNING: PRO-X1.
WHA25435.1    302 NSSM   0.5448     (6/9)   +
WHA25435.1    499 NGTY   0.5824     (6/9)   +
WHA25435.1    558 NGSL   0.6827     (9/9)   ++
----------------------------------------------------------------------
```

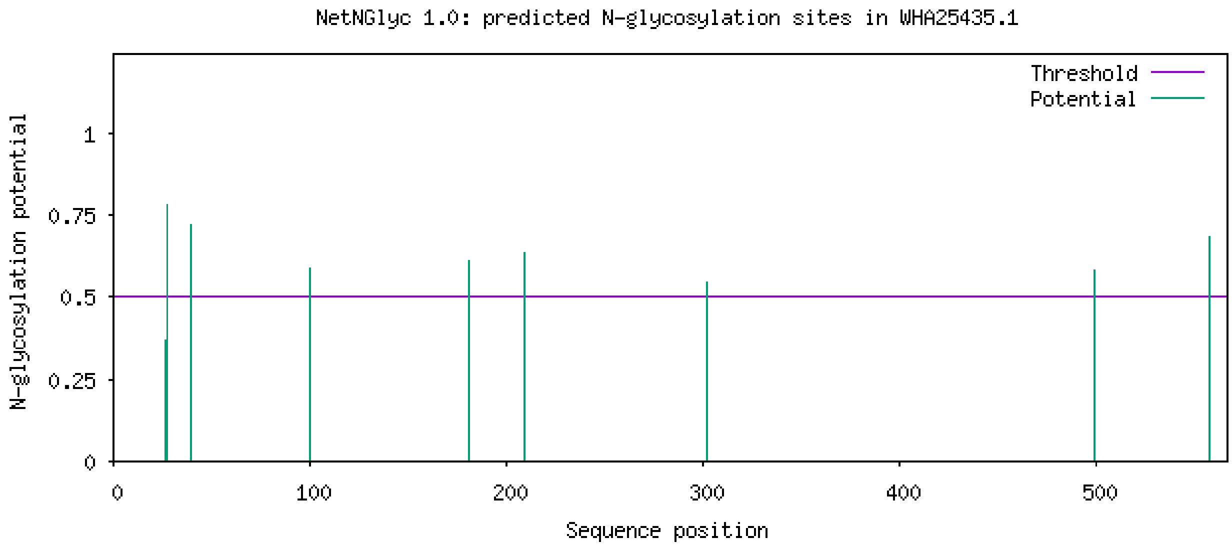

>WHA25437.1 neuraminidase [Influenza A virusA/Vulture/Florida/22-012327-003/2022]

```
Name:  WHA25437.1       Length:  469
MNPNQKITTIGSICMVIGIVSLMLQIGNIISIWVSHSIQTGNQYQPEPCNQSIITYENNTWVNQTYVNISNTNFLAEQAV      80
TSVTLAGNSSLCPISGWAIYSKDNGIRIGSKGDVFVIREPFISCSHLECRTFFLTQGALLNDKHSNGTVKDRSPYRTLMS     160
CPVGEAPSPYNSRFESVAWSASACHDGISWLTIGISGPDNGAVAVLKYNGIITDTIKSWRNNILRTQESECACVNGSCFT     240
VMTDGPSNGQASYKIFKIEKGKVVKSVELNAPNYHYEECSCYPDAGDIMCVCRDDWHGSNRPWVSFNQNLEYQIGYICSG     320
VFGDNPRPNDGTGSCSPMSSNGAYGVKGFSFKYGNGVWIGRTKSTSSRSGFEMIWDPNGWTETDSSFSVKQDIVEITDWS     400
GYSGSFVQHPELTGLDCMRPCFWVELIRGRPKENTIWTSGSSISFCGVNSDTVGWSWPDGAELPFTIDK                480
.................................................N.......N....N....N............      80
.......N.........................................................N..............     160
..........................................................................N.....     240
................................................................................     320
................................................................................     400
.....................................................................                480
```

(Threshold=0.5)

SeqName	Position	Potential	Jury agreement	N-Glyc result
WHA25437.1	50 NQSI	0.5884	(8/9)	+
WHA25437.1	58 NNTW	0.5494	(6/9)	+
WHA25437.1	63 NQTY	0.6636	(9/9)	++
WHA25437.1	68 NISN	0.7377	(9/9)	++
WHA25437.1	88 NSSL	0.7724	(9/9)	+++
WHA25437.1	146 NGTV	0.6874	(9/9)	++
WHA25437.1	235 NGSC	0.7321	(9/9)	++

NetNGlyc 1.0: predicted N-glycosylation sites in WHA25437.1

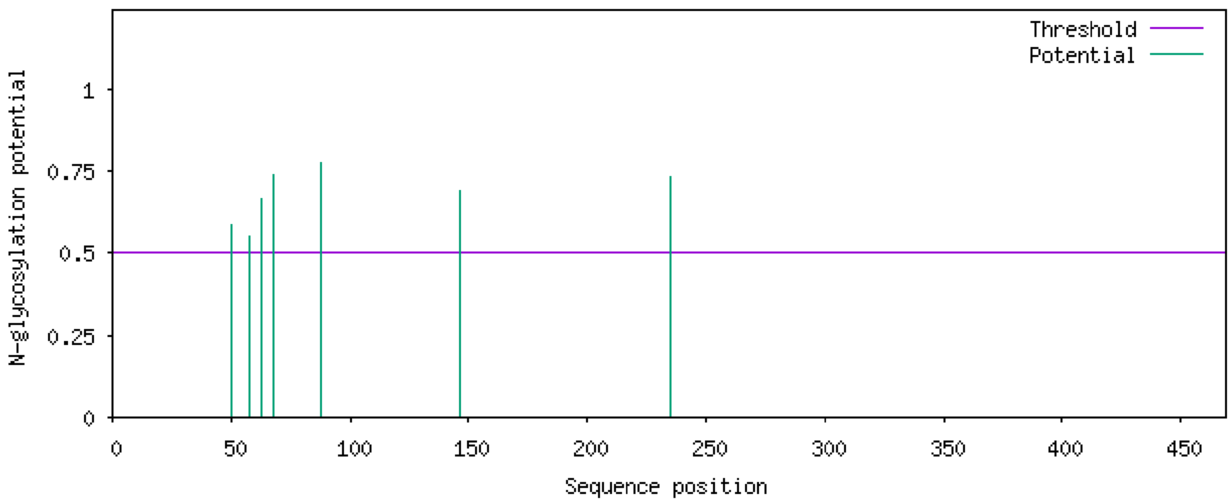

22. >WLK83164.1 hemagglutinin [Influenza A virus/Fregata-magnificens/RJ/1532-N/2023]

```
Name:  WLK83164.1      Length:  567
MENIVLLLAIVSLVKSDQICIGYHANNSTEQVDTIMEKNVTVTHAQDILEKTHNGKLCDLNGVKPLILKDCSVAGWLLGN      80
PMCDEFIRVPEWSYIVERANPANDLCYPGSLNDYEELKHMLSRINHFEKIQIIPKSSWPNHETSLGVSAACPYQGAPSFF     160
RNVVWLIKKNDAYPTIKISYNNTNREDLLILWGIHHSNNAEEQTNLYKNPTTYISVGTSTLNQRLAPKIATRSQVNGQRG     240
RIDFFWTILKPDDAIHFESNGNFIAPEYAYKIVKKGDSTIMKSGVEYGHCNTKCQTPVGAINSSMPFHNIHPLTIGECPK     320
YVKSNKLVLATGLRNSPLREKRKKRGLFGAIAGFIEGGWQGMVDGWYGYHHSNEQGSGYAADKESTQKAIDGVTNKVNSI     400
IDKMNTQFEAVGREFNNLERRIENLNKKMEDGFLDVWTYNAELLVLMENERTLDFHDSNVKNLYDKVRLQLRDNAKELGN     480
GCFEFYHKCDNECMESVRNGTYYYPQYSEEARLKREEISGVKLESVGTYLILSIYSTAASSLALAIMMAGLSLWMCSNGS     560
LQCRICI                                                                              640
..........................N...........N.........................................      80
................................................................................     160
....................N...........................N...............................     240
.............................................................N..................     320
................................................................................     400
................................................................................     480
..................N..........................................................N..     560
.......                                                                              640

(Threshold=0.5)
----------------------------------------------------------------------
SeqName      Position  Potential   Jury     N-Glyc
                                 agreement  result
----------------------------------------------------------------------
WLK83164.1     26 NNST   0.3687     (9/9)    --
WLK83164.1     27 NSTE   0.7796     (9/9)    +++
WLK83164.1     39 NVTV   0.7181     (9/9)    ++
WLK83164.1    181 NNTN   0.6100     (7/9)    +
WLK83164.1    209 NPTT   0.6336     (8/9)    +     WARNING: PRO-X1.
WLK83164.1    302 NSSM   0.5447     (6/9)    +
WLK83164.1    499 NGTY   0.5821     (5/9)    +
WLK83164.1    558 NGSL   0.6828     (9/9)    ++
----------------------------------------------------------------------
```

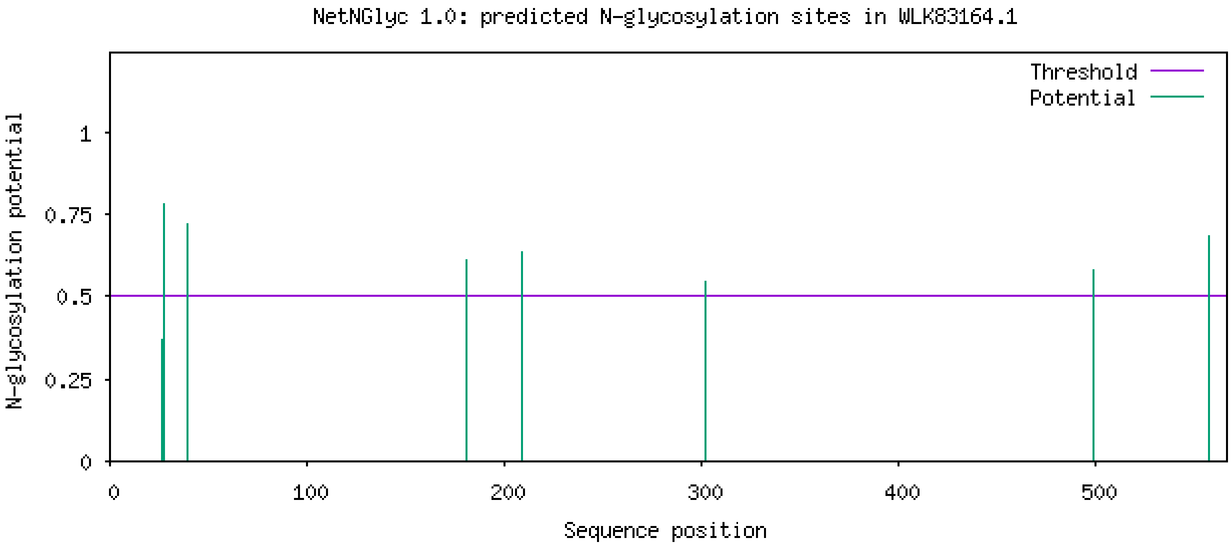

>WLK83166.1 neuraminidase, partial [Influenza A virus/Fregata-magnificens/RJ/1532-N/2023]

```
Name:  WLK83166.1       Length:  459
MNPNQKITTIGSICMVIGIVSLMLQIGNIISIWVSHSIQTGNQYQPEPCNQSIITYENNTWVNQTYVNISNTNFLAEQAV      80
TSVTLAGNSSLCPISGWAIYSKDNGIRIGSKGDVFVIREPFISCSHLECRTFFLTQGALLNDKHSNGTVKDRSPYRTLMS     160
CPVGEAPSPYNSRFESVAWSASACHDGISWLTIGISGPDNGAVAVLKYNGIITDTIKSWRNNILRTQESECACVNGSCFT     240
VMTDGPSNGQASYKIFKIEKGKVVKSVEMNAPNYHYEECSCYPDAGDIMCVCRDNWHGSNRPWVSFNQNLEYQIGYICSG     320
VFGDNPRPNDGTGSCSPMPSNGAYGVKGFSFKYGNGVWIGRTKSTSSRSGFEMIWDPNGWTETDSSFSVKQDIVEITDWS     400
GYSGSFVQHPELTGLDCMRPCFWVELIRGRPKENTIWTSGSSISFCGVNSDTVGWSWPD                          480
.................................................N.......N....N....N............      80
.......N.........................................................N..............     160
..........................................................................N.....     240
................................................................................     320
................................................................................     400
...........................................................                          480

(Threshold=0.5)
----------------------------------------------------------------------
SeqName      Position  Potential   Jury    N-Glyc
                                 agreement result
----------------------------------------------------------------------
WLK83166.1    50 NQSI   0.5881     (8/9)    +
WLK83166.1    58 NNTW   0.5493     (6/9)    +
WLK83166.1    63 NQTY   0.6631     (9/9)    ++
WLK83166.1    68 NISN   0.7373     (9/9)    ++
WLK83166.1    88 NSSL   0.7721     (9/9)    +++
WLK83166.1   146 NGTV   0.6863     (9/9)    ++
WLK83166.1   235 NGSC   0.7307     (9/9)    ++
----------------------------------------------------------------------
```

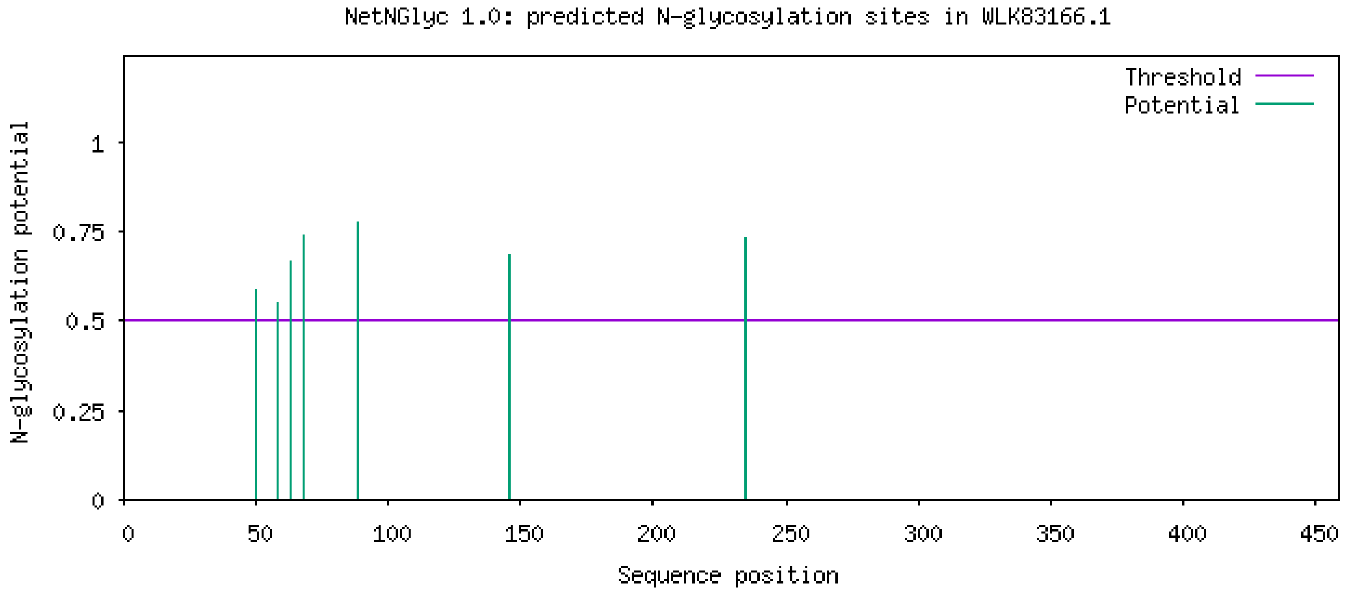

23. >WLC96945.1 hemagglutinin [Influenza A virus/Backyard-duck/Uruguay/124-M3/2023]

```
Name:  WLC96945.1       Length:  567
MENIVLLLAIVSLVKSDQICIGYHANNSTEQVDTIMEKNVTVTHAQDILEKTHNGKLCDLNGVKPLILKDCSVAGWLLGN      80
PMCDEFIRVPEWSYIVERSNPANDLCYPGSLNDYEELKHMLSRINHFEKIQIIPKSSWPNHETSLGVSAACPYQGAPSFF     160
RNVVWLIKKNDAYPTIKISYNNTNREDLLILWGIHHSNNAEEQTNLYKNPTTYISVGTSTLNQRLAPKIATRSQVNGQRG     240
RMDFFWTILKPDDAIHFESNGNFIAPEYAYKIVKKGDSTIMKSGVEYGHCNTKCQTPVGAINSSMPFHNIHPLTIGECPK     320
YVKSNKLVLATGLRNSPLREKRRKRGLFGAIAGFIEGGWQGMVDGWYGYHHSNEQGSGYAADKESTQKAIDGVTNKVNSI     400
IDKMNTQFEAVGREFNNLERRIENLNKKMEDGFLDVWTYNAELLVLMENERTLDFHDSNVKNLYDKVRLQLRDNAKELGN     480
GCFEFYHKCDNECMESVRNGTYDYPQYSEEARLKREEISGVKLESVGTYQILSIYSTAASSLALAIMMAGLSLWMCSNGS     560
LQCRICI                                                                              640
..........................N...........N.........................................      80
................................................................................     160
....................N...........................N...............................     240
.............................................................N..................     320
................................................................................     400
................................................................................     480
..................N...........................................................N.     560
.......                                                                              640

(Threshold=0.5)
----------------------------------------------------------------------
SeqName      Position  Potential   Jury    N-Glyc
                                 agreement result
----------------------------------------------------------------------
WLC96945.1    26 NNST   0.3686     (9/9)   --
WLC96945.1    27 NSTE   0.7797     (9/9)   +++
WLC96945.1    39 NVTV   0.7181     (9/9)   ++
WLC96945.1   181 NNTN   0.6101     (7/9)   +
WLC96945.1   209 NPTT   0.6336     (8/9)   +     WARNING: PRO-X1.
WLC96945.1   302 NSSM   0.5447     (6/9)   +
WLC96945.1   499 NGTY   0.5824     (6/9)   +
WLC96945.1   558 NGSL   0.6828     (9/9)   ++
----------------------------------------------------------------------
```

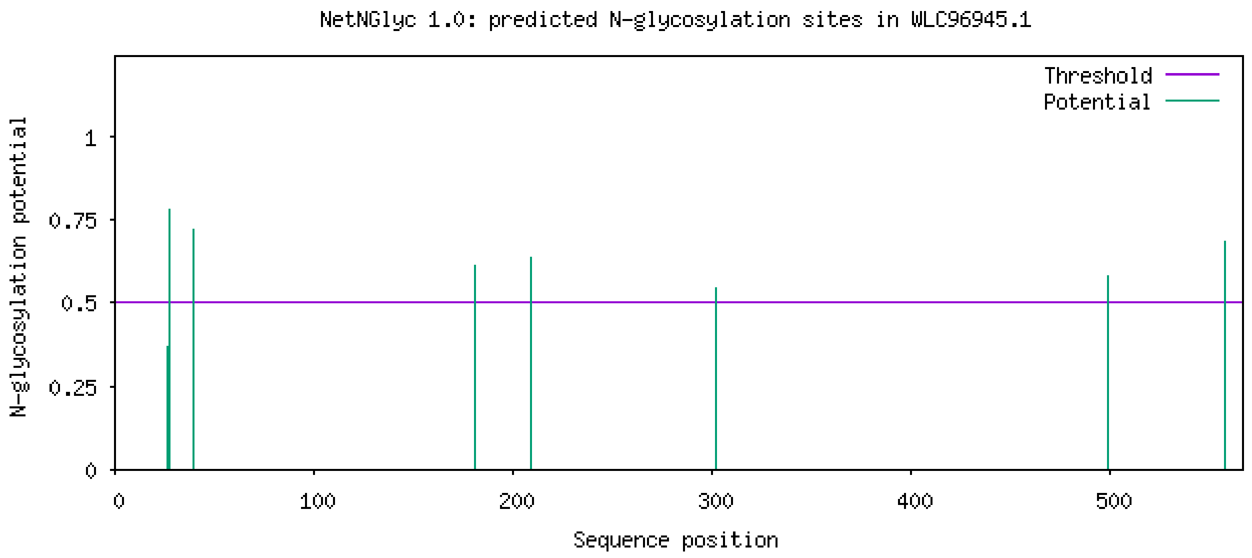

>WLC96943.1 neuraminidase [Influenza A virus/Backyard-duck/Uruguay/124-M3/2023]

```
Name:  WLC96943.1       Length:  469
MNPNQKITTIGSICMVIGIVSLMLQIGNIISIWVSHSIQTGNQYQPEPCNQSIITYENNTWVNQTYVNISNTNFLAEQAV      80
TSVTLAGNSSLCPISGWAIYSKDNGIRIGSKGDVFVIREPFISCSHLECRTFFLTQGALLNDKHSNGTVKDRSPYRTLMS     160
CPVGEAPSPYNSRFESVAWSASACHDGISWLTIGISGPDNGAVAVLKYNGIITDTIKSWRNNILRTQESECACVNGSCFT     240
VMTDGPSNGQASYKIFKIEKGKVVKSVEMNAPNYHYEECSCYPDAGDIMCVCRDNWHGSNRPWVSFNQNLEYQIGYICSG     320
VFGDNPRPNDGTGSCSPMPYNGAYGVKGFSFKYGNGVWIGRTKSTSSRSGFEMIWDPNGWTETDSSFSVKQDIVEITDWS     400
GYSGSFVQHPELTGLDCMRPCFWVELIRGRPKENTIWTSGSSISFCGVNSDTVGWSWPDGAELPFTIDK                480
.................................................N.......N....N....N............      80
.......N.........................................................N..............     160
..........................................................................N.....     240
................................................................................     320
................................................................................     400
.....................................................................                480

(Threshold=0.5)
----------------------------------------------------------------------
SeqName      Position  Potential   Jury    N-Glyc
                                agreement  result
----------------------------------------------------------------------
WLC96943.1    50 NQSI   0.5886     (8/9)   +
WLC96943.1    58 NNTW   0.5496     (6/9)   +
WLC96943.1    63 NQTY   0.6637     (9/9)   ++
WLC96943.1    68 NISN   0.7378     (9/9)   ++
WLC96943.1    88 NSSL   0.7724     (9/9)   +++
WLC96943.1   146 NGTV   0.6874     (9/9)   ++
WLC96943.1   235 NGSC   0.7320     (9/9)   ++
----------------------------------------------------------------------
```

NetNGlyc 1.0: predicted N-glycosylation sites in WLC96943.1

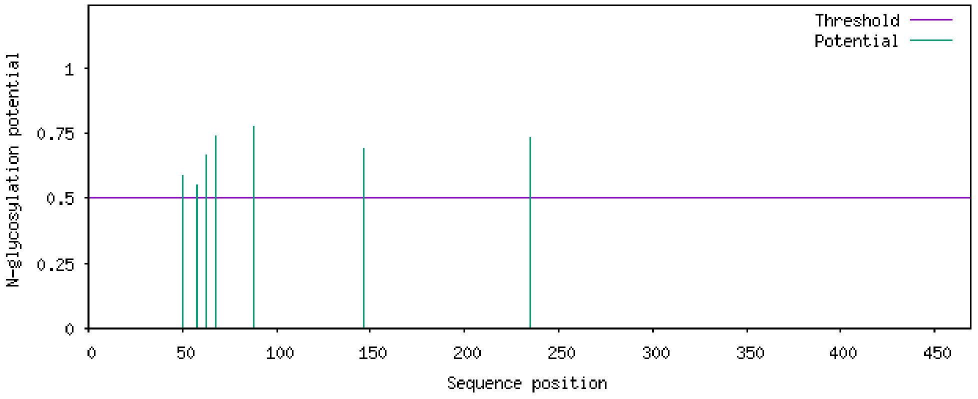

24. >WNI41922.1 hemagglutinin [Influenza A virus/Belcher-gull/Peru/A267/2022]

```
Name:  WNI41922.1_HA_Influenza_A_virus_Belcher-gull_Peru_A267_2022_     Length:  567
MENIVLLLAIVSLVKSDQICIGYHANNSTEQVDTIMEKNVTVTHAQDILEKTHNGKLCDLNGVKPLILKDCSVAGWLLGN      80
PMCDEFIRVPEWSYIVERANPANDLCYPGSLNDYEELKHMLSRINHFEKIQIIPKSSWPNHETSLGVSAACPYQGAPSFF     160
RNVVWLIKKNDAYPTIKISYNNTNREDLLILWGIHHSNNAEEQTNLYKNPTTYISVGTSTLNQRLAPKIATRSQVNGQRG     240
RMDFFWTILKPDDAIHFESNGNFIAPEYAYKIVKKGDSTIMKSGVEYGHCNTKCQTPVGAINSSMPFHNIHPLTIGECPK     320
YVKSNKLVLATGLRNSPLREKRRKRGLFGAIAGFIEGGWQGMVDGWYGYHHSNEQGSGYAADKESTQKAIDGVTNKVNSI     400
IDKMNTQFEAVGREFNNLERRIENLNKKMEDGFLDVWTYNAELLVLMENERTLDFHDSNVKNLYDKVRLQLRDNAKELGN     480
GCFEFYHKCDNECMESVRNGTYDYPQYSEEARLKREEISGVKLESVGTYQILSIYSTAASSLALAIMMAGLSLWMCSNGS     560
LQCRICI                                                                              640
..........................N...........N.........................................      80
................................................................................     160
....................N...........................N...............................     240
.............................................................N..................     320
................................................................................     400
................................................................................     480
..................N..........................................................N..     560
.......                                                                              640

(Threshold=0.5)
```

SeqName	Position		Potential	Jury agreement	N-Glyc result	
WNI41922.1_HA_Influenza_A_virus_Belcher-gull_Peru_A267_2022_	26	NNST	0.3686	(9/9)	--	
WNI41922.1_HA_Influenza_A_virus_Belcher-gull_Peru_A267_2022_	27	NSTE	0.7797	(9/9)	+++	
WNI41922.1_HA_Influenza_A_virus_Belcher-gull_Peru_A267_2022_	39	NVTV	0.7182	(9/9)	++	
WNI41922.1_HA_Influenza_A_virus_Belcher-gull_Peru_A267_2022_	181	NNTN	0.6100	(7/9)	+	
WNI41922.1_HA_Influenza_A_virus_Belcher-gull_Peru_A267_2022_	209	NPTT	0.6336	(8/9)	+	WARNING: PRO-X1.
WNI41922.1_HA_Influenza_A_virus_Belcher-gull_Peru_A267_2022_	302	NSSM	0.5447	(6/9)	+	
WNI41922.1_HA_Influenza_A_virus_Belcher-gull_Peru_A267_2022_	499	NGTY	0.5824	(6/9)	+	
WNI41922.1_HA_Influenza_A_virus_Belcher-gull_Peru_A267_2022_	558	NGSL	0.6827	(9/9)	++	

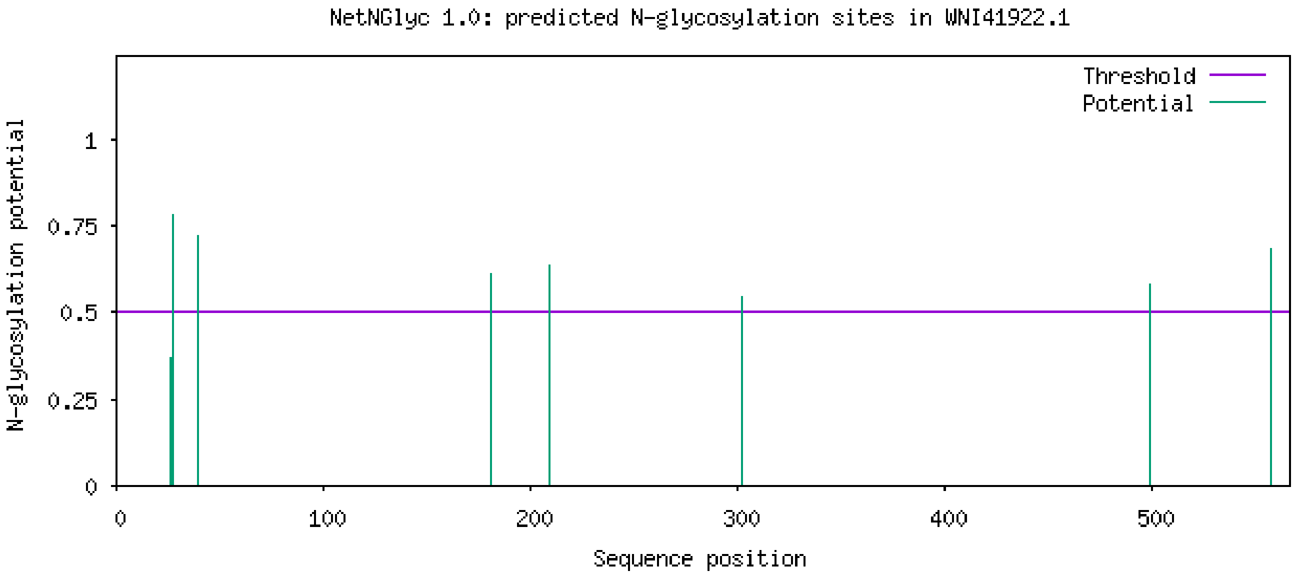

>WNI41940.1 neuraminidase [Influenza A virus/Belcher-gull/Peru/A267/2022]

```
Name:  WNI41940.1       Length:  469
MNPNQKITTIGSICMVIGIVSLMLQIGNIISIWVSHSIQTGNQYQPEPCNQSIITYENNTWVNQTYVNISNTNFLAEQAV      80
TSVTLAGNSSLCPISGWAIYSKDNGIRIGSKGDVFVIREPFISCSHLECRTFFLTQGALLNDKHSNGTVKDRSPYRTLMS     160
CPVGEAPSPYNSRFESVAWSASACHDGISWLTIGISGPDNGAVAVLKYNGIITDTVKSWRNNILRTQESECACVNGSCFT     240
VMTDGPSNGQASYKIFKIEKGKVVKSVEMNAPNYHYEECSCYPDAGDIMCVCRDNWHGSNRPWVSFNQNLEYQIGYICSG     320
VFGDNPRPNDGTGSCSPMPSNGAYGVKGFSFKYGNGVWIGRTKNTSSRSGFEMIWDPNGWTETDSSFSVKQDIVEITDWS     400
GYSGSFVQHPELTGLDCMRPCFWVELIRGRPKENTIWTSGSSISFCGVNSDTVGWSWPDGAELPFTIDK                480
.................................................N.......N....N....N............      80
.......N.........................................................N..............     160
..........................................................................N.....     240
................................................................................     320
................................................................................     400
.....................................................................                480

(Threshold=0.5)
----------------------------------------------------------------------
SeqName      Position  Potential   Jury    N-Glyc
                                agreement  result
----------------------------------------------------------------------
WNI41940.1     50 NQSI   0.5884     (8/9)    +
WNI41940.1     58 NNTW   0.5494     (6/9)    +
WNI41940.1     63 NQTY   0.6636     (9/9)    ++
WNI41940.1     68 NISN   0.7378     (9/9)    ++
WNI41940.1     88 NSSL   0.7724     (9/9)    +++
WNI41940.1    146 NGTV   0.6874     (9/9)    ++
WNI41940.1    235 NGSC   0.7320     (9/9)    ++
WNI41940.1    364 NTSS   0.3551     (9/9)    --
----------------------------------------------------------------------
```

NetNGlyc 1.0: predicted N-glycosylation sites in WNI41940.1

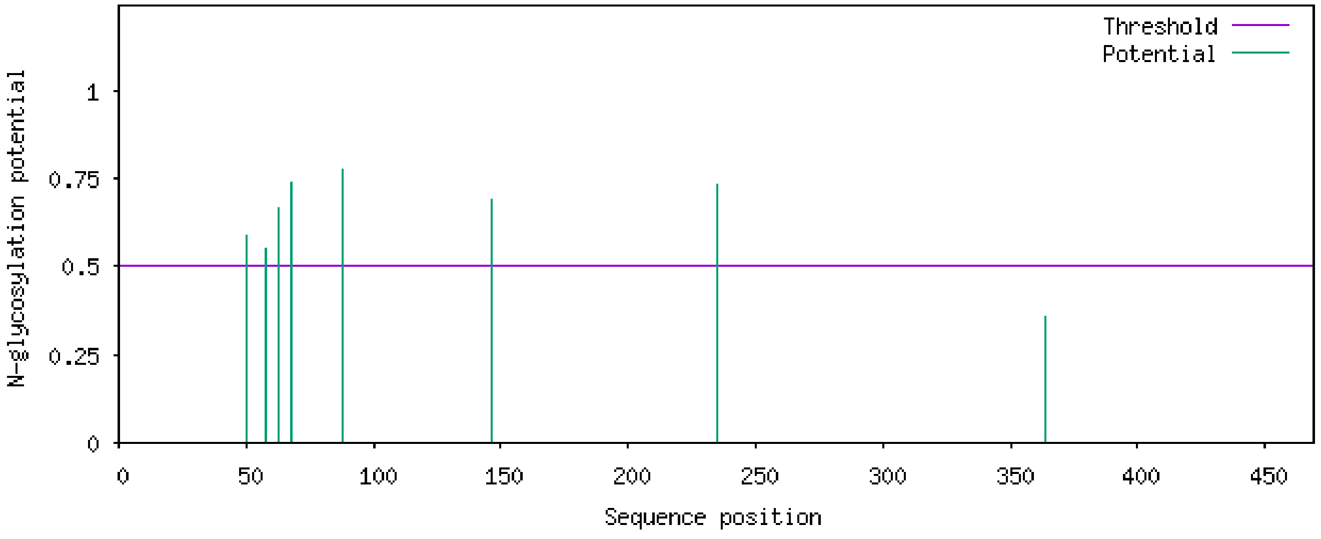

25. >WDG26823.1 hemagglutinin [Influenza A virus/Pelican/CHL/227023-1/2022]

```
Name:  WDG26823.1       Length:  567
MENIVLLLAIVSLVKSDQICIGYHANNSTEQVDTIMEKNVTVTHAQDILEKTHNGKLCDLNGVKPLILKDCSVAGWLLGN      80
PMCDEFIRVPEWSYIVERANPANDLCYPGSLNDYEELKHMLSRINHFEKIQIIPKSSWPNHETSLGVSAACPYQGAPSFF     160
RNVVWLIKKNDAYPTIKISYNNTNREDLLILWGIHHSNNAEEQTNLYKNPTTYISVGTSTLNQRLAPKIATRSQVNGQRG     240
RMDFFWTILKPDDAIHFESNGNFIAPEYAYKIVKKXDSTIMKSGVEYGHCNTKCQTPVGAINSSMPFHNIHPLTIGECPK     320
YVKSNKLVLATGLRNSPLREKRRKRGLFGAIAGFIEGGWQGMVDGWYGYHHSNEXGSGYAADKESTQKAIDGVTNKVNSI     400
IDKMNTQFEAVGREFNNLERRIENLNKKMEDGFLDVWTYNAELLVLMENERTLDFHDSNVKNLYDKVRLQLRDNAKELGN     480
GCFEFYHKCDNECMESVRNGTYDYPQYSEEARLKREEISGVKLESVGTYQILSIYSTAASSLALAIMMAGLSLWMCSNGS     560
LQCRICI                                                                              640
..........................N...........N.........................................      80
................................................................................     160
....................N...........................N...............................     240
.............................................................N..................     320
................................................................................     400
................................................................................     480
..................N..........................................................N..     560
.......                                                                              640
```

```
(Threshold=0.5)
----------------------------------------------------------------------
SeqName      Position  Potential   Jury    N-Glyc
                                 agreement result
----------------------------------------------------------------------
WDG26823.1     26 NNST   0.3687     (9/9)   --
WDG26823.1     27 NSTE   0.7796     (9/9)   +++
WDG26823.1     39 NVTV   0.7182     (9/9)   ++
WDG26823.1    181 NNTN   0.6100     (7/9)   +
WDG26823.1    209 NPTT   0.6336     (8/9)   +     WARNING: PRO-X1.
WDG26823.1    302 NSSM   0.5447     (6/9)   +
WDG26823.1    499 NGTY   0.5824     (6/9)   +
WDG26823.1    558 NGSL   0.6828     (9/9)   ++
----------------------------------------------------------------------
```

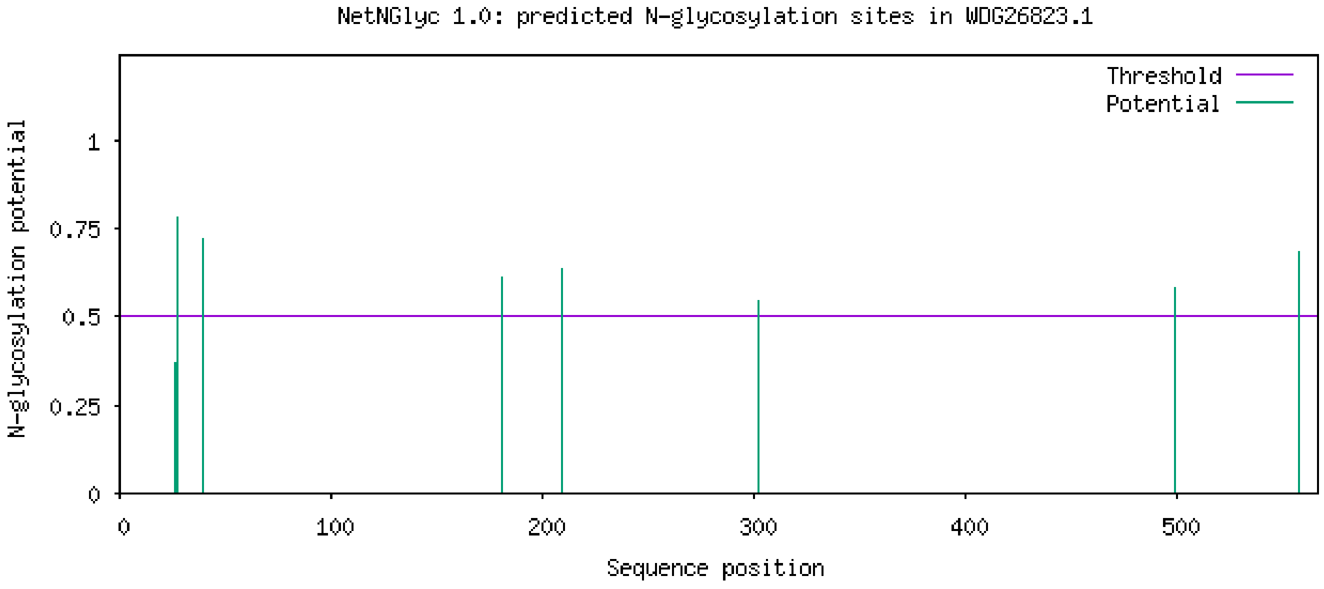

No tiene esta NA muestra

26. >WPS69896.1 hemagglutinin [Influenza A virusA/Humboldt-penguin/Antofagasta/236063-2/2023]

```
Name:  WPS69896.1       Length:  567
MENIVLLLAIVSLVKSDQICIGYHANNSTEQVDTIMEKNVTVTHAQDILEKTHNGKLCDLNGVKPLILKDCSVAGWLLGN      80
PMCDEFIRVPEWSYIVERANPANDLCYPGSLNDYEELKHMLSRINHFEKIQIIPKSSWPNHETSLGVSAACPYQGAPSFF     160
RNVVWLIKKNDAYPTIKISYNNTNREDLLILWGIHHSNNAKEQTNLYKNPTTYISVGTSTLNQRLAPKIATRSQVNGQRG     240
RMDFFWTILKPDDAIHFESNGNFIAPEYAYKIVKKGDSTIMKSGVEYGHCNTKCQTPVGAINSNMPFHNIHPLTIGECPK     320
YVKSNKLVLATGLRNSPLREKRRKRGLFGAIAGFIEGGWQGMVDGWYGYHHSNEQGSGYAADKESTQKAIDGVTNKVNSI     400
IDKMNTQFEAVGREFNNLERRIENLNKKMEDGFLDVWTYNAELLVLMENERTLDFHDSNVKNLYDKVRLQLRDNAKELGN     480
GCFEFYHKCDNECMESVRNGTYDYPQYSEEARLKREEISGVKLESVGTYQILSIYSTAASSLALAIMMAGLSLWMCSNGS     560
LQCRICI                                                                              640
..........................N...........N.........................................      80
................................................................................     160
....................N...........................N...............................     240
................................................................................     320
................................................................................     400
................................................................................     480
..................N..........................................................N..     560
.......                                                                              640

(Threshold=0.5)
----------------------------------------------------------------------
SeqName      Position  Potential   Jury     N-Glyc
                                 agreement  result
----------------------------------------------------------------------
WPS69896.1    26 NNST   0.3686     (9/9)    --
WPS69896.1    27 NSTE   0.7796     (9/9)    +++
WPS69896.1    39 NVTV   0.7181     (9/9)    ++
WPS69896.1   181 NNTN   0.6100     (7/9)    +
WPS69896.1   209 NPTT   0.6378     (8/9)    +     WARNING: PRO-X1.
WPS69896.1   499 NGTY   0.5824     (6/9)    +
WPS69896.1   558 NGSL   0.6826     (9/9)    ++
----------------------------------------------------------------------
```

NetNGlyc 1.0: predicted N-glycosylation sites in WPS69896.1

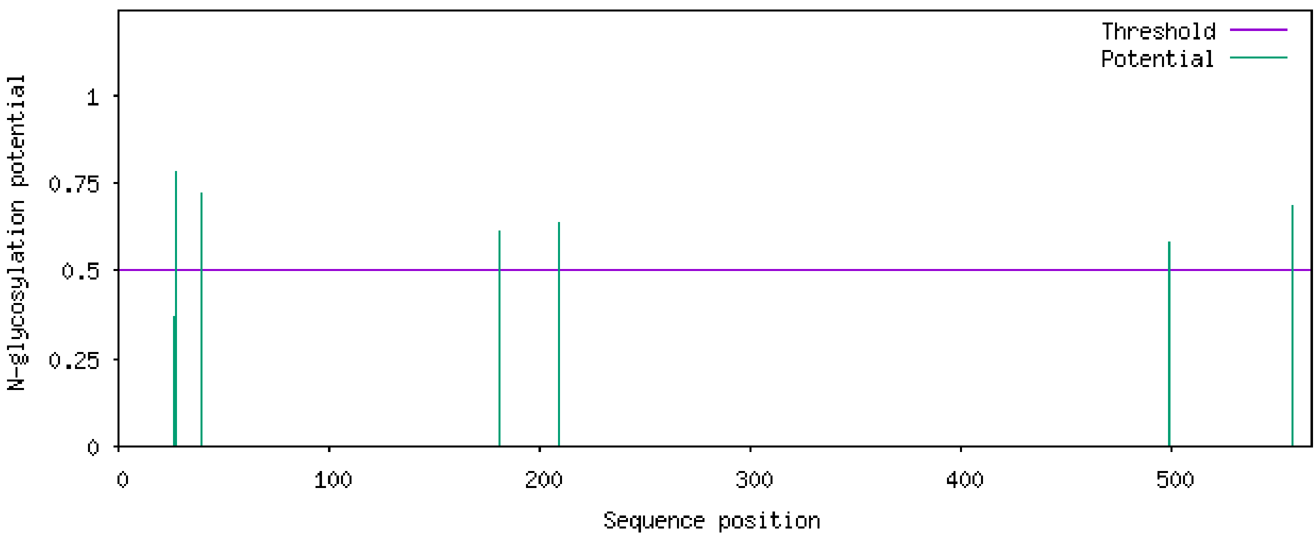

>WPS69898.1 neuraminidase [Influenza A virusHumboldt-penguin/Antofagasta/236063-2/2023]

```
Name:  WPS69898.1       Length:  469
MNPNQKITTIGSICMVIGIVSLMLQIGNIISIWVSHSIQTGNQYQPEPCNQSIITYENNTWVNQTYVNISNTNFLAEQAV      80
TSVTLAGNSSLCPISGWAIYSKDNGIRIGSKGDVFVIREPFISCSHLECRTFFLTQGALLNDKHSNGTVKDRSPYRTLMS     160
CPVGEAPSPYNSRFESVAWSASACHDGISWLTIGISGPDNGAVAVLKYNGIITDTIKSWRNNILRTQESECACVNGSCFT     240
VMTDGPSNGQASYKIFKIEKGKVVKSVEMNAPNYHYEECSCYPDAGDIMCVCRDNWHGSNRPWVSFNQNLEYQIGYICSG     320
VFGDNPRPNDGTGSCSPMPSNGAYGVKGFSFKYGNGVWIGRTKSTSSRSGFEMIWDPNGWTETDSSFSVKQDIVEITDWS     400
GYSGSFVQHPELTGLDCMRPCFWVELIRGRPKENTIWTSGSSISFCGVNSDTVGWSWPDGAELPFTIDK                480
.................................................N.......N....N....N............      80
.......N.........................................................N..............     160
..........................................................................N.....     240
................................................................................     320
................................................................................     400
.....................................................................                480

(Threshold=0.5)
----------------------------------------------------------------------
SeqName      Position  Potential   Jury    N-Glyc
                                agreement  result
----------------------------------------------------------------------
WPS69898.1     50 NQSI   0.5884     (8/9)   +
WPS69898.1     58 NNTW   0.5496     (6/9)   +
WPS69898.1     63 NQTY   0.6636     (9/9)   ++
WPS69898.1     68 NISN   0.7378     (9/9)   ++
WPS69898.1     88 NSSL   0.7724     (9/9)   +++
WPS69898.1    146 NGTV   0.6876     (9/9)   ++
WPS69898.1    235 NGSC   0.7321     (9/9)   ++
----------------------------------------------------------------------
```

NetNGlyc 1.0: predicted N-glycosylation sites in WPS69898.1

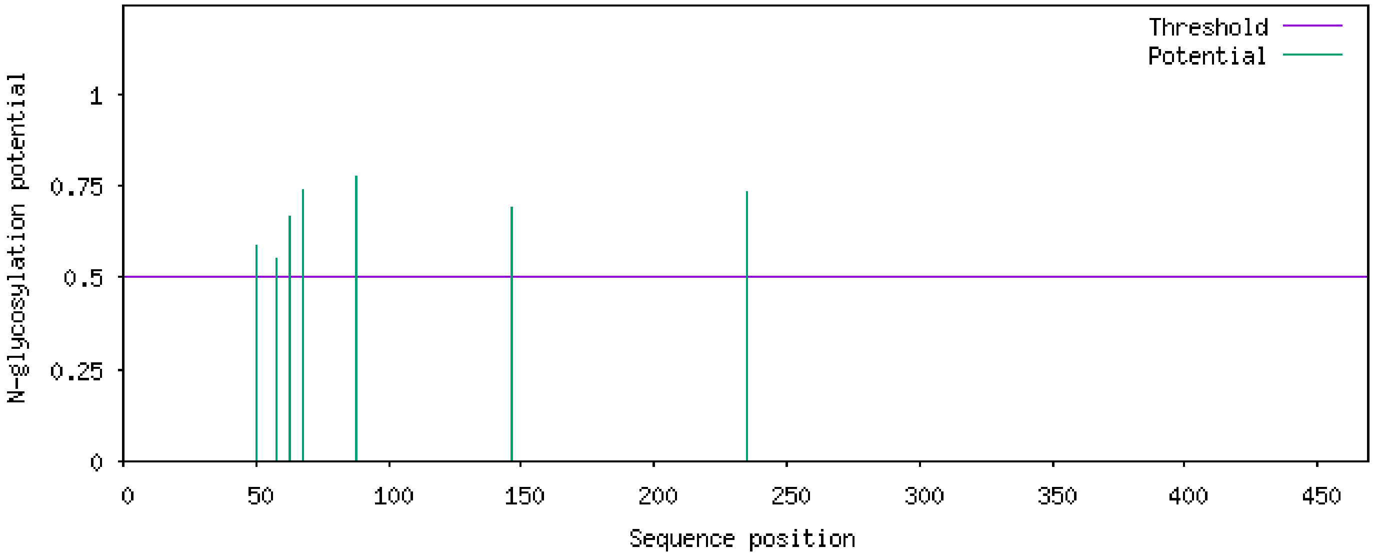

27. >WDZ16834.1 hemagglutinin [Influenza A virus/Pelecanus/Peru/VFAR-140/2022]

```
Name:  WDZ16834.1      Length:  567
MENIVLLLAIVSLVKSDQICIGYHANNSTEQVDXIMEKNVTVTHAQDILEKTHNGKLCDLNGVKPLILKDCSVAGWLLGN      80
PMCDEFIRVPEWSYIVEXANPANDLCYPGSLNDYEELKHMLSRINHFEKIQIIPKSSWPNHETSLGVSAACPYQGAPSFF     160
RNVVWLIKKNDAYPTIKISYNNTNXEDLLILWGIHHSNNAEEQTNLYKNPTTYISVGTSTLNQRLAPKIATRSQVNXQRG     240
RMDFFWTILKPDDAIHFESNGNFIAPEYAYKIVKKGDSTIMKSGVEYGHCNTKCQTPVGAINSSMPFHNIHPLTIGECPK     320
YVKSNKLVLATGLRNSPLREKRRKRGLFGAIAGFIEGGWQGMVDGWYGYHHSNEQGSGYAADKESTQKAIDGVTNKVNSI     400
IDKMNTQFEAVGREFNNLERRIENLNKKMEDGFLDVWTYNAELLVLMENERTLDFHDSNVKNLYDKVRLQLRDNAKELGN     480
GCFEFYHKCDNECMESVRNGTYDYPQYSEEARLKREEISGVKLESVGTYQILSIYSTAASSLALAIMMAGLSLWMCSNGS     560
LQCRICI                                                                              640
.........................N...........N..........................................      80
................................................................................     160
....................N...........................N...............................     240
..............................................................N.................     320
................................................................................     400
................................................................................     480
..................N...........................................................N..     560
.......                                                                              640
```

(Threshold=0.5)

SeqName	Position	Potential	Jury agreement	N-Glyc result	
WDZ16834.1	26 NNST	0.3638	(9/9)	--	
WDZ16834.1	27 NSTE	0.7810	(9/9)	+++	
WDZ16834.1	39 NVTV	0.7296	(9/9)	++	
WDZ16834.1	181 NNTN	0.6100	(6/9)	+	
WDZ16834.1	209 NPTT	0.6336	(8/9)	+	WARNING: PRO-X1.
WDZ16834.1	302 NSSM	0.5447	(6/9)	+	
WDZ16834.1	499 NGTY	0.5826	(6/9)	+	
WDZ16834.1	558 NGSL	0.6826	(9/9)	++	

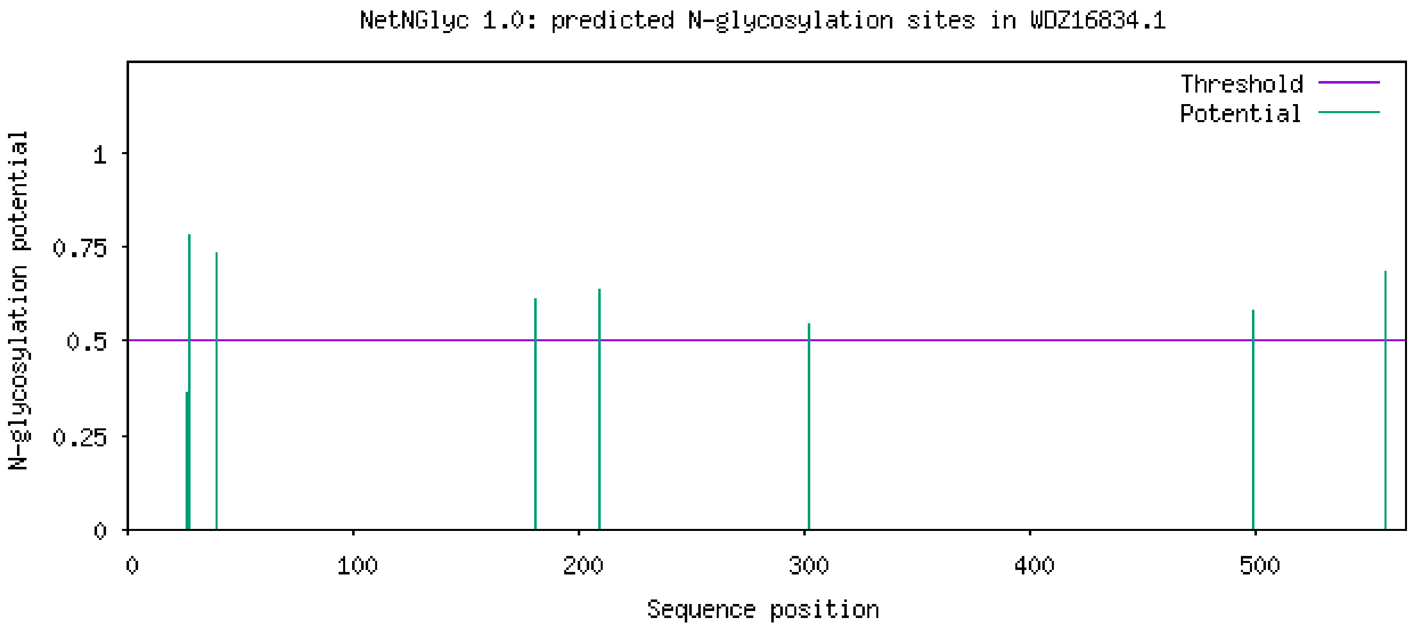

>WDZ16836.1 neuraminidase, partial [Influenza A virus/Pelecanus/Peru/VFAR-140/2022]

```
Name:  WDZ16836.1      Length:  460
IGSXCMVIGIVSLMLQIGNIISIWVSHSIQTGNQYQPEPCNQSIITYENNTWVNQTYVNISNTNFLAEQAVTSVTLAGNS      80
SLCPISGWAIYSKDNGIRIGSKGDVFVIREPFISCSHLECRTFFLTQGALLNDKHSNGTVKDRSPYRTLMSCPVGEAPSP     160
YNSRFESVAWSASACHDGISWLTIGISGPDNGAVAVLKYNGIITDTIKSWRNNILRTQESECACVNGSCFTVMTDGPSNG     240
QASYKIFKIEKGKVVKSVEMNAPNYHYEECSCYPDAGDIMCVCRDNWHGSNRPWVSFNQNLEYQIGYICSGVFXDNPRPN     320
DGTGSCSPMPSNGAYGVKGFSFKYGNGVWIGRTKSTSSRSGFEMIWDPNGWTETDSSFSVKQDIVEITDWSGYSGSFVQH     400
PELTGLDCMRPCFWVELIRGRPKENTIWTSGSSISFCGVNSDTVGWSWPDXAELPFTIDK                         480
........................................N.......N....N....N...................N.      80
........................................................N.......................     160
.................................................................N..............     240
................................................................................     320
................................................................................     400
............................................................                         480

(Threshold=0.5)
----------------------------------------------------------------------
SeqName      Position  Potential   Jury    N-Glyc
                                agreement  result
----------------------------------------------------------------------
WDZ16836.1    41 NQSI   0.5917     (8/9)    +
WDZ16836.1    49 NNTW   0.5529     (6/9)    +
WDZ16836.1    54 NQTY   0.6662     (9/9)    ++
WDZ16836.1    59 NISN   0.7394     (9/9)    ++
WDZ16836.1    79 NSSL   0.7742     (9/9)    +++
WDZ16836.1   137 NGTV   0.6897     (9/9)    ++
WDZ16836.1   226 NGSC   0.7333     (9/9)    ++
----------------------------------------------------------------------
```

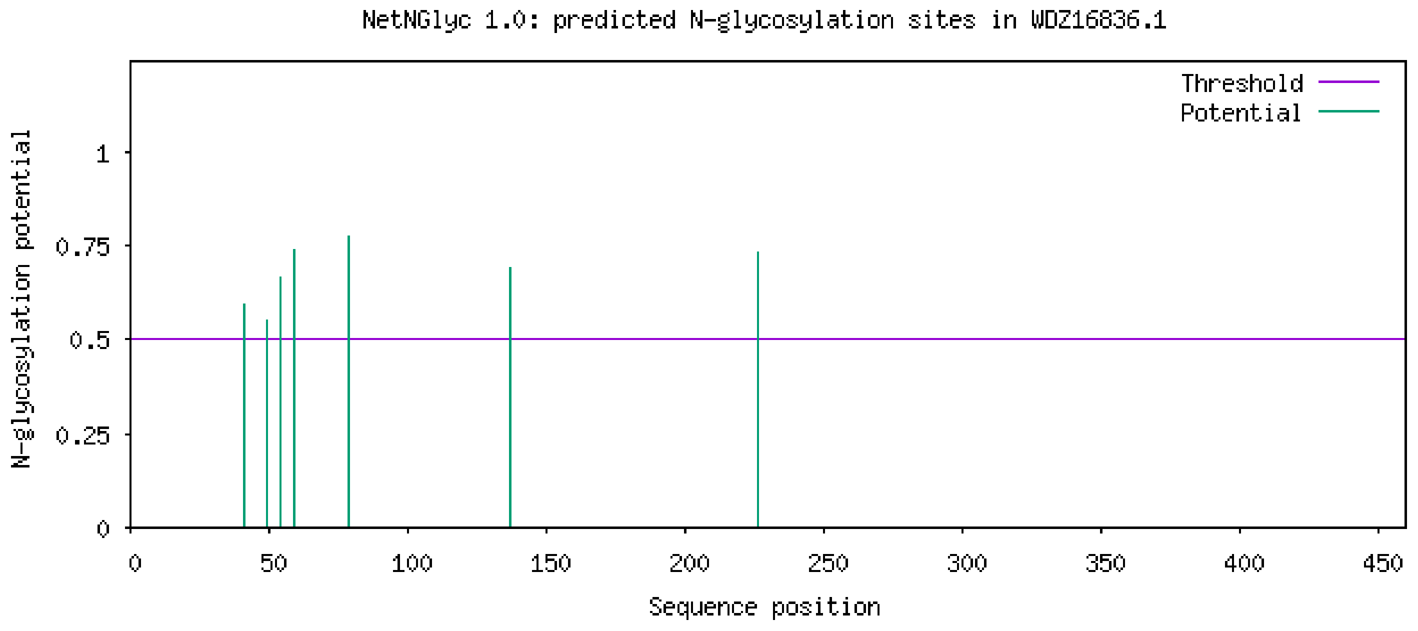

28. >WPS69889.1 hemagglutinin [Influenza A virus/cormorant/Antofagasta/236025-1/2023]

```
Name:  WPS69889.1       Length:  567
MENIVLLLAIVSLVKSDQICIGYHANNSTEQVDTIMEKNVTVTHAQDILEKTHNGKLCDLNGVKPLILKDCSVAGWLLGN      80
PMCDEFIRVPEWSYIVERANPANDLCYPGSLNDYEELKHMLSRINHFEKIQIIPKSSWPNHETSLGVSAACPYQGAPSFF     160
RNVVWLIKKNDAYPTIKISYNNTNREDLLILWGIHHSNNAKEQTNLYKNPTTYISVGTSTLNQRLAPKIATRSQVNGQRG     240
RMDFFWTILKPDDAIHFESNGNFIAPEYAYKIVKKGDSTIMKSGVEYGHCNTKCQTPVGAINSSMPFHNIHPLTIGECPK     320
YVKSNKLVLATGLRNSPLREKRRKRGLFGAIAGFIEGGWQGMVDGWYGYHHSNEQGSGYAADKESTQKAIDGVTNKVNSI     400
IDKMNTQFEAVGREFNNLERRIENLNKKMEDGFLDVWTYNAELLVLMENERTLDFHDSNVKNLYDKVRLQLRDNAKELGN     480
GCFEFYHKCDNECMESVRNGTYDYPQYSEEARLKREEISGVKLESVGTYQILSIYSTAASSLALAIMLAGLSLWMCSNGS     560
LQCRICI                                                                              640
..........................N...........N.........................................      80
................................................................................     160
....................N...........................N...............................     240
.............................................................N..................     320
................................................................................     400
................................................................................     480
..................N..........................................................N..     560
.......                                                                              640
```

(Threshold=0.5)

SeqName	Position	Potential	Jury agreement	N-Glyc result	
WPS69889.1	26 NNST	0.3687	(9/9)	--	
WPS69889.1	27 NSTE	0.7794	(9/9)	+++	
WPS69889.1	39 NVTV	0.7181	(9/9)	++	
WPS69889.1	181 NNTN	0.6100	(7/9)	+	
WPS69889.1	209 NPTT	0.6379	(8/9)	+	WARNING: PRO-X1.
WPS69889.1	302 NSSM	0.5446	(6/9)	+	
WPS69889.1	499 NGTY	0.5824	(6/9)	+	
WPS69889.1	558 NGSL	0.6808	(9/9)	++	

NetNGlyc 1.0: predicted N-glycosylation sites in WPS69889.1

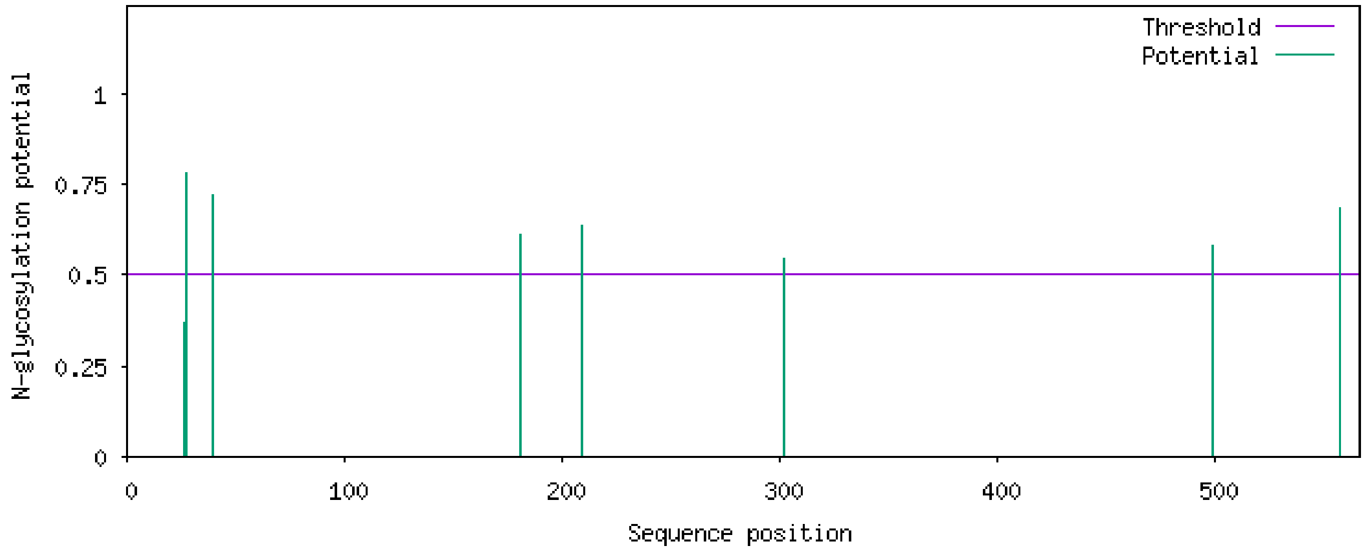

>WQF62332.1 neuraminidase [Influenza A virus/cormorant/Antofagasta/236025-1/2023]

```
Name:  WQF62332.1      Length:  469
MNPNQKITTIGSICMVIGIVSLMLQIGNIISIWVSHSIQTGNQYQPEPCNQSIITYENNTWVNQTYVNISNTNFLAEQAV      80
TSVTLAGNSSLCPISGWAIYSKDNGIRIGSKGDVFVIREPFISCSHLECRTFFLTQGALLNDKHSNGTVKDRSPYRTLMS     160
CPVGEAPSPYNSRFESVAWSASACHDGISWLTIGISGPDNGAVAVLKYNGIITDTIKSWRNNILRTQESECACVNGSCFT     240
VMTDGPSNGQASYKIFKIEKGKVVKSVEMNAPNYHYEECSCYPDAGDIMCVCRDNWHGSNRPWVSFNQNLEYQIGYICSG     320
VFGDNPRPNDGTGSCSPMPSNGAYGVKGFSFKYGNGVWIGRTKSTSSRSGFEMIWDPNGWTETDSSFSVKQDIVEITDWS     400
GYSGSFVQHPELTGLDCMRPCFWVELIRGRPKENTIWTSGSSISFCGVNSDTVGWSWPDGAELPFTIDK                480
.................................................N.......N....N....N............      80
.......N.........................................................N..............     160
..........................................................................N.....     240
................................................................................     320
................................................................................     400
.....................................................................                480

(Threshold=0.5)
----------------------------------------------------------------------
SeqName       Position  Potential   Jury      N-Glyc
                                  agreement  result
----------------------------------------------------------------------
WQF62332.1     50 NQSI   0.5884     (8/9)     +
WQF62332.1     58 NNTW   0.5496     (6/9)     +
WQF62332.1     63 NQTY   0.6636     (9/9)     ++
WQF62332.1     68 NISN   0.7378     (9/9)     ++
WQF62332.1     88 NSSL   0.7724     (9/9)     +++
WQF62332.1    146 NGTV   0.6876     (9/9)     ++
WQF62332.1    235 NGSC   0.7321     (9/9)     ++
----------------------------------------------------------------------
```

NetNGlyc 1.0: predicted N-glycosylation sites in WQF62332.1

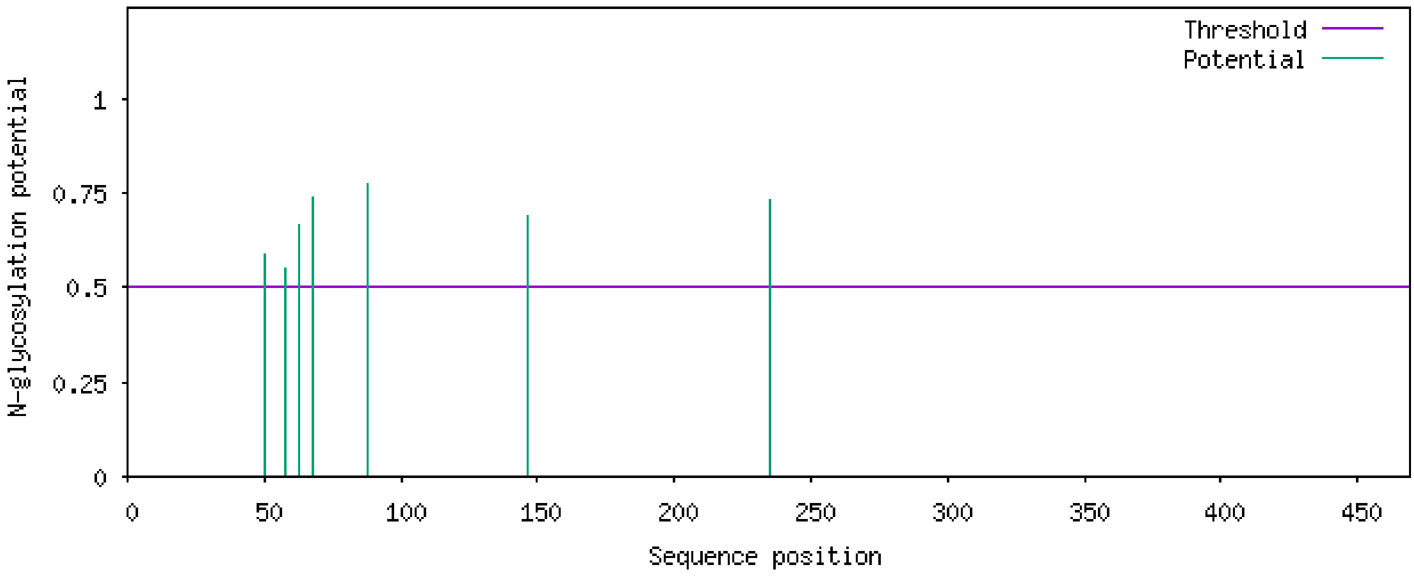

29. >WGF23330.2 hemagglutinin, partial [Influenza A virus/Andean-Guayata/Argentina/140223/2023]

```
Name:  Sequence          Length:  563
VLLLVIVSLVKSDQICIGYHANNSTEQVDTIMEKNVTVTHAQDILEKTHNGKLCDLNGVKPLILKDCSVAGWLLGNPMCD      80
EFIRVPEWSYIVERANPANDLCYPGSLNDYEELKHMLSRINHFEKIQIIPKSSWPNHETSLGVSAACPYQGAPSFFRNVV     160
WLIKKNDAYPTIKISYNNTNREDLLILWGIHHSNNAEEQTNLYKNPTTYISVGTSTLNQRLAPKIATRSQVNGQRGRMDF     240
FWTILKPDDAIHFESNGNFIAPEYAYKIVKKGDSTIMKSGVEYGHCNTKCQTPVGAINSSMPFHNIHPLTIGECPKYVKS     320
NKLVLATGLRNSPLREKRRKRGLFGAIAGFIEGGWQGMVDGWYGYHHSNEQGSGYAADKESTQKAIDGVTNKVNSIIDKM     400
NTQFEAVGREFNNLERRIENLNKKMEDGFLDVWTYNAELLVLMENERTLDFHDSNVKNLYDKVRLQLRDNAKELGNGCFE     480
FYHKCDNECMESVRNGTYDYPQYSEEARLKREEISGVKLESVGTYQILSIYSTAASSLALAIMMAGLSLWMCSNGSLQCR     560
ICI                                                                                  640
......................N...........N.............................................      80
................................................................................     160
................N...........................N...................................     240
.........................................................N......................     320
................................................................................     400
................................................................................     480
..............N..........................................................N......     560
...                                                                                  640

(Threshold=0.5)
----------------------------------------------------------------------
SeqName      Position  Potential   Jury    N-Glyc
                                agreement  result
----------------------------------------------------------------------
Sequence       22 NNST   0.3699    (9/9)    --
Sequence       23 NSTE   0.7802    (9/9)    +++
Sequence       35 NVTV   0.7190    (9/9)    ++
Sequence      177 NNTN   0.6108    (7/9)    +
Sequence      205 NPTT   0.6346    (8/9)    +     WARNING: PRO-X1.
Sequence      298 NSSM   0.5456    (6/9)    +
Sequence      495 NGTY   0.5824    (6/9)    +
Sequence      554 NGSL   0.6829    (9/9)    ++
----------------------------------------------------------------------
```

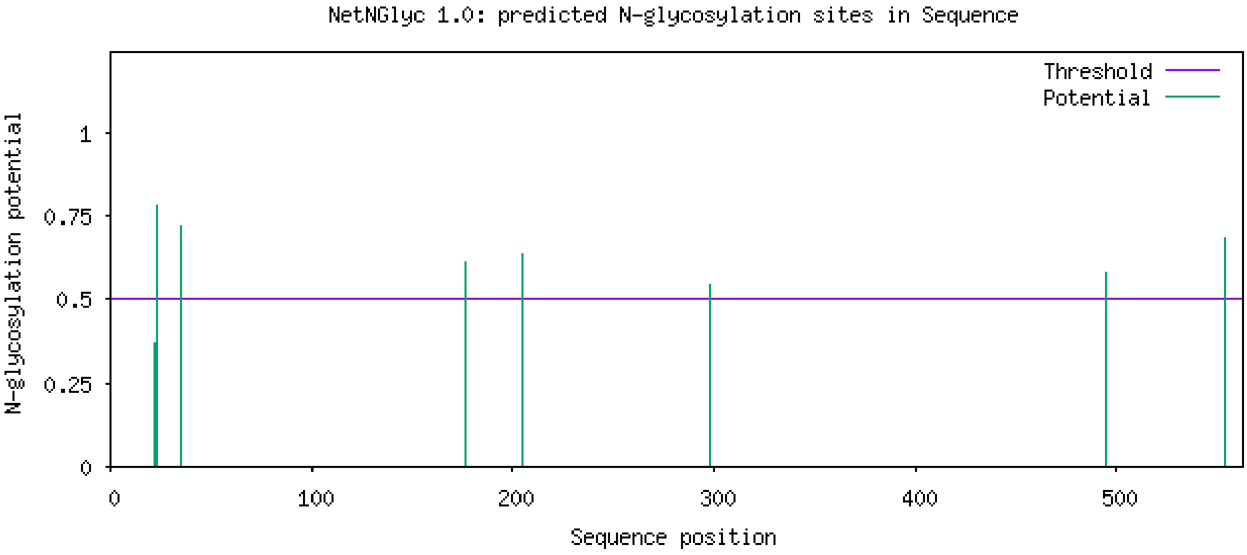

>WGF23959.1 neuraminidase, partial [Influenza A virus/Andean-Guayata/Argentina/140223/2023]

```
Name:  WGF23959.1       Length:  451
CMVIGIVSLMLQTGNIISIWVSHSIQTGNQYQPEPCNQSIITYENNTWVNQTYVNISNTNFLAEQAVTSVTLAGNSSLCP      80
ISGWAIYSKDNGIRIGSKGDVFVIREPFISCSHLECRTFFLTQGALLNDKHSNGTVKDRSPYRTLMSCPVGEAPSPYNSR     160
FESVAWSASACHDGISWLTIGISGPDNGAVAVLKYNGIITDTIKSWRNNILRTQESECACVNGSCFTVMTDGPSNGQASY     240
KIFKIEKGKVVKSVEMNAPNYHYEECSCYPDAGDIMCVCRDNWHGSNRPWVSFNQNLEYQIGYICSGVFGDNPRPNDGTG     320
SCSPMPSNGAYGVKGFSFKYGNGVWIGRTKSTSSRSGFEMIWDPNGWTETDSSFSVKQDIVEITDWSGYSGSFVQHPELT     400
GLDCMRPCFWVELIRGRPKENTIWTSGSSISFCGVNSDTVGWSWPDGAELP                                  480
....................................N.......N....N....N...................N.....      80
....................................................N...........................     160
.............................................................N..................     240
................................................................................     320
................................................................................     400
...................................................                                  480
```

(Threshold=0.5)

SeqName	Position		Potential	Jury agreement	N-Glyc result
WGF23959.1	37	NQSI	0.5931	(8/9)	+
WGF23959.1	45	NNTW	0.5543	(6/9)	+
WGF23959.1	50	NQTY	0.6677	(9/9)	++
WGF23959.1	55	NISN	0.7403	(9/9)	++
WGF23959.1	75	NSSL	0.7749	(9/9)	+++
WGF23959.1	133	NGTV	0.6901	(9/9)	++
WGF23959.1	222	NGSC	0.7334	(9/9)	++

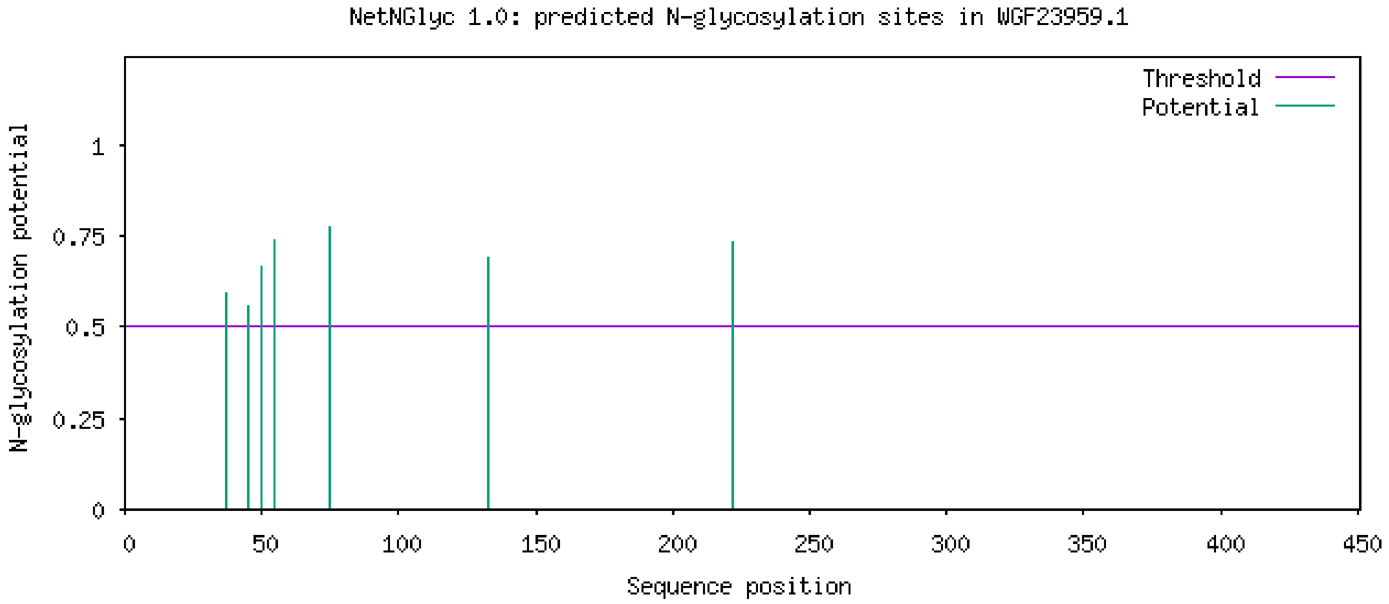

30. >WKF21371.1 hemagglutinin [Influenza A virus/Northern_Pintail/USA/IZ22_0760_swab/2022]

```
Name:  WKF21371.1       Length:  567
MENIVLLLAIVSLVKSDQICIGYHANNSTEQVDTIMEKNVTVTHAQDILEKTHNGKLCDLNGVKPLILKDCSVAGWLLGN      80
PMCDEFIRVPEWSYIVERANPANDLCYPGSLNDYEELKHMLSRINHFEKILIIPKSSWPNHETSLGVSAACPYQGAPSFF     160
RNVVWLIKKNDAYPTIKISYNNTNREDLLILWGIHHSNNAEEQTNLYKNPTTYISVGTSTLNQRLAPKIATRSQVNGQRG     240
RMDFFWTILKPDDAIHFESNGNFIAPEYAYKIVKKGDSTIMKSGVEYGHCNTKCQTPVGAINSSMPFHNIHPLTIGECPK     320
YVKSNKLVLATGLRNSPLREKRRKRGLFGAIAGFIEGGWQGMVDGWYGYHHSNEQGSGYAADKESTQKAIDGVTNKVNSI     400
IDKMNTQFEAVGREFNNLERRIENLNKKMEDGFLDVWTYNAELLVLMENERTLDFHDSNVKNLYDKVRLQLRDNAKELGN     480
GCFEFYHKCDNECMESVRNGTYDYPQYSEEARLKREEISGVKLESVGTYQILSIYSTAASSLALAIMMAGLSLWMCSNGS     560
LQCRICI                                                                              640
..........................N...........N.........................................      80
................................................................................     160
....................N...........................N...............................     240
.............................................................N..................     320
................................................................................     400
................................................................................     480
..................N..........................................................N..     560
.......                                                                              640

(Threshold=0.5)
----------------------------------------------------------------------
SeqName      Position  Potential   Jury    N-Glyc
                                 agreement result
----------------------------------------------------------------------
WKF21371.1    26 NNST   0.3687     (9/9)   --
WKF21371.1    27 NSTE   0.7797     (9/9)   +++
WKF21371.1    39 NVTV   0.7182     (9/9)   ++
WKF21371.1   181 NNTN   0.6100     (7/9)   +
WKF21371.1   209 NPTT   0.6336     (8/9)   +     WARNING: PRO-X1.
WKF21371.1   302 NSSM   0.5447     (6/9)   +
WKF21371.1   499 NGTY   0.5824     (6/9)   +
WKF21371.1   558 NGSL   0.6828     (9/9)   ++
----------------------------------------------------------------------
```

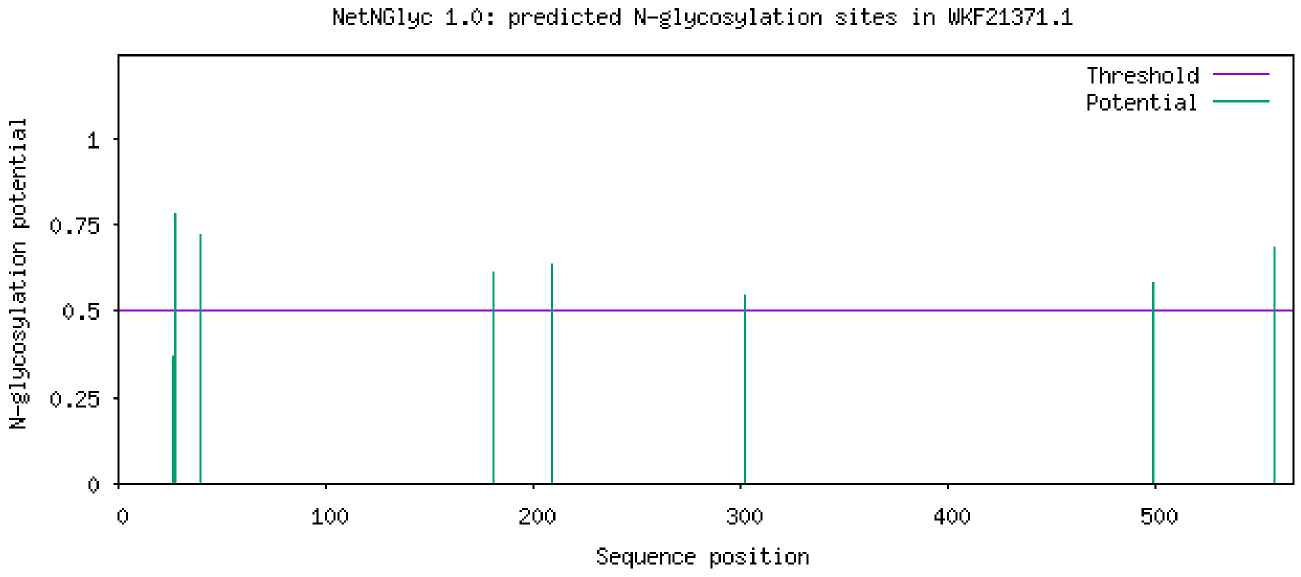

>WKF21373.1 neuraminidase [Influenza A virus/Northern_Pintail/USA/IZ22_0760_swab/2022]

```
Name:  WKF21373.1       Length:  469
MNPNQKITTIGSICMVIGIVSLMLQIGNIISIWVSHSIQTGNQYQPEPCNQSIITYENNTWVNQTYVNISNTNFLAEQAV      80
TSVTLAGNSSLCPISGWAIYSKDNGIRIGSKGDVFVIREPFISCSHLECRTFFLTQGALLNDKHSNGTVKDRSPYRTLMS     160
CPVGEAPSPYNSRFESVAWSASACHDGFSWLTIGISGPDNGAVAVLKYNGIITDTIKSWRNNILRTQESECACINGSCFT     240
VMTDGPSNGQASYKIFKIEKGKVVKSVELNAPNYHYEECSCYPDAGDIMCVCRDNWHGSNRPWVSFNQNLEYQIGYICSG     320
VFGDNPRPNDGTGSCSPMSSNGAYGVKGFSFKYGNGVWIGRTKSTSSRSGFEMIWDPNGWTETDSSFSVKQDIVEITEWS     400
GYSGSFVQHPELTGLDCMRPCFWVELIRGRPKENTIWTSGSSISFCGVNSDTVGWSWPDGAELPFTIDK                480
.................................................N.......N....N....N............      80
.......N.........................................................N..............     160
..........................................................................N.....     240
................................................................................     320
................................................................................     400
.....................................................................                480

(Threshold=0.5)
```

SeqName	Position	Potential	Jury agreement	N-Glyc result
WKF21373.1	50 NQSI	0.5882	(8/9)	+
WKF21373.1	58 NNTW	0.5496	(6/9)	+
WKF21373.1	63 NQTY	0.6634	(9/9)	++
WKF21373.1	68 NISN	0.7376	(9/9)	++
WKF21373.1	88 NSSL	0.7723	(9/9)	+++
WKF21373.1	146 NGTV	0.6873	(9/9)	++
WKF21373.1	235 NGSC	0.6786	(9/9)	++

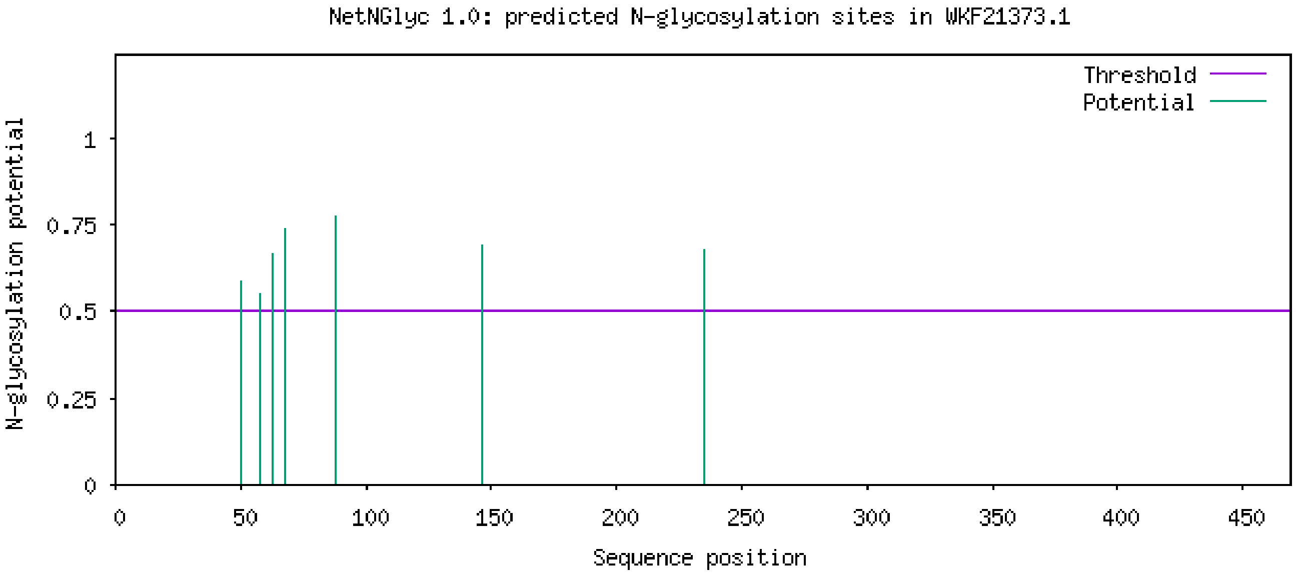

31. >WHA27283.1 hemagglutinin [Influenza A virus/goose/Alaska/22-013831-003/2022]

```
Name:  WHA27283.1       Length:  567
MENIVLLLAIVSLVKSDQICIGYHANNSTEQVDTIMEKNVTVTHAQDILEKTHNGKLCDLNGVKPLILKDCSVAGWLLGN      80
PMCDEFIRVPEWSYIVERANPANDLCYPGSLNDYEELKHMLSRINHFEKILIIPKSSWPNHETSLGVSAACPYQGAPSFF     160
RNVVWLIKKNDAYPTIKISYNNTNQEDLLILWGIHHSNNAEEQTNLYKNPTTYISVGTSTLNQRLAPKIATRSQVNGQRG     240
RMDFFWTILKPDDAIHFESNGNFIAPEYAYKIVKKGDSTIMKSGVEYGHCNTKCQTPVGAINSSMPFHNIHPLTIGECPK     320
YVKSNKLVLATGLRNSPLREKRRKRGLFGAIAGFIEGGWQGMVDGWYGYHHSNEQGSGYAADKESTQKAIDGVTNKVNSI     400
IDKMNTQFEAVGREFNNLERRIENLNKKMEDGFLDVWTYNAELLVLMENERTLDFHDSNVKNLYDKVRLQLRDNAKELGN     480
GCFEFYHKCDNECMESVRNGTYDYPQYSEEARLKREEISGVKLESVGTYQILSIYSTAASSLALAIMMAGLSLWMCSNGS     560
LQCRICI                                                                              640
..........................N...........N.........................................      80
................................................................................     160
....................N...........................N...............................     240
.............................................................N..................     320
................................................................................     400
................................................................................     480
..................N..........................................................N..     560
.......                                                                              640
```

(Threshold=0.5)

SeqName	Position	Potential	Jury agreement	N-Glyc result	
WHA27283.1	26 NNST	0.3686	(9/9)	--	
WHA27283.1	27 NSTE	0.7796	(9/9)	+++	
WHA27283.1	39 NVTV	0.7181	(9/9)	++	
WHA27283.1	181 NNTN	0.6410	(7/9)	+	
WHA27283.1	209 NPTT	0.6336	(8/9)	+	WARNING: PRO-X1.
WHA27283.1	302 NSSM	0.5447	(6/9)	+	
WHA27283.1	499 NGTY	0.5824	(6/9)	+	
WHA27283.1	558 NGSL	0.6826	(9/9)	++	

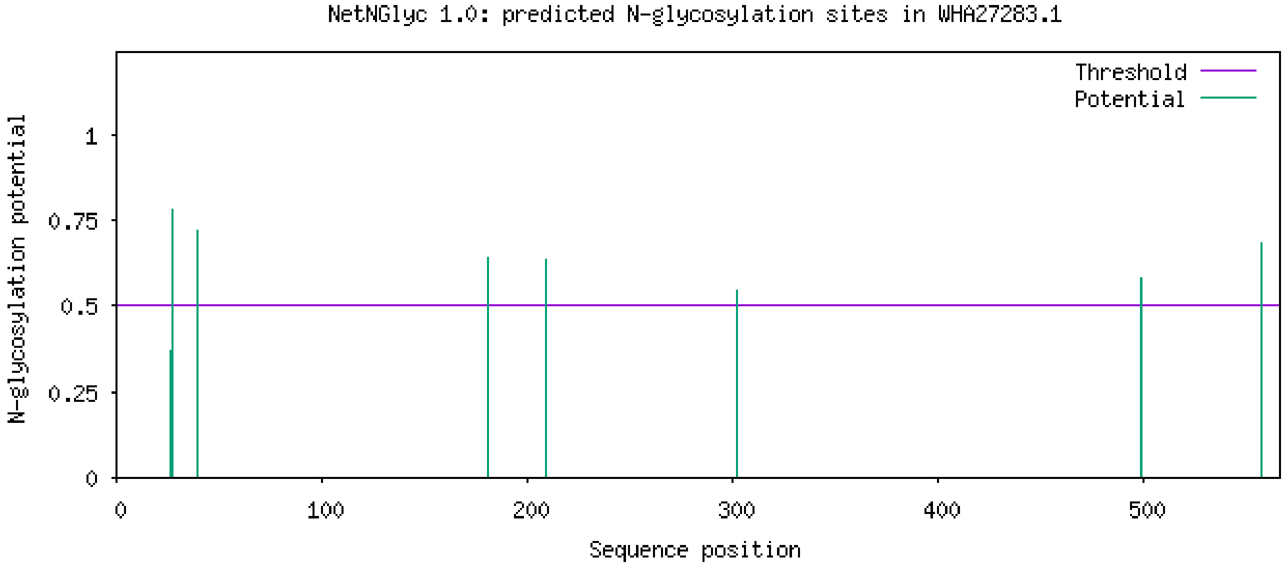

>WHA27285.1 neuraminidase [Influenza A virus/goose/Alaska/22-013831-003/2022]

```
Name:   WHA27285.1        Length:   469
MNPNQKITTIGSICMVIGIVSLMLQIGNIISIWVSHSIQTGNQYQPEPCNQSIITYENNTWVNQTYVNISNTNFLAEQAV      80
TSVTLAGNSSLCPISGWAIYSKDNGIRIGSKGDVFVIREPFISCSHLECRTFFLTQGALLNDKHSNGTVKDRSPYRTLMS     160
CPVGEAPSPYNSRFESVAWSASACHDGISWLTIGISGPDNGAVAVLKYNGIITDTIKSWRNNILRTQESECACVNGSCFT     240
VMTDGPSNGQASYKIFKIEKGKVVKSVELNAPNYHYEECSCYPDAGDIMCVCRDNWHGSNRPWVSFNQNLEYQIGYICSG     320
VFGDNPRPNDGTGSCSPMSSNGAYGVKGFSFKYGNGVWIGRTKSTSSRSGFEMIWDPNGWTETDSSFSVKQDIVEITDWS     400
GYSGSFVQHPELTGLDCMRPCFWVELIRGRPKENTIWTSGSSISFCGVNSDTVGWSWPDGAELPFTIDK                480
.................................................N.......N....N....N............      80
.......N.........................................................N..............     160
..........................................................................N.....     240
................................................................................     320
................................................................................     400
.....................................................................                480

(Threshold=0.5)
----------------------------------------------------------------------
SeqName      Position  Potential    Jury    N-Glyc
                                 agreement  result
----------------------------------------------------------------------
WHA27285.1     50 NQSI   0.5883     (8/9)    +
WHA27285.1     58 NNTW   0.5494     (6/9)    +
WHA27285.1     63 NQTY   0.6636     (9/9)    ++
WHA27285.1     68 NISN   0.7377     (9/9)    ++
WHA27285.1     88 NSSL   0.7724     (9/9)    +++
WHA27285.1    146 NGTV   0.6873     (9/9)    ++
WHA27285.1    235 NGSC   0.7321     (9/9)    ++
----------------------------------------------------------------------
```

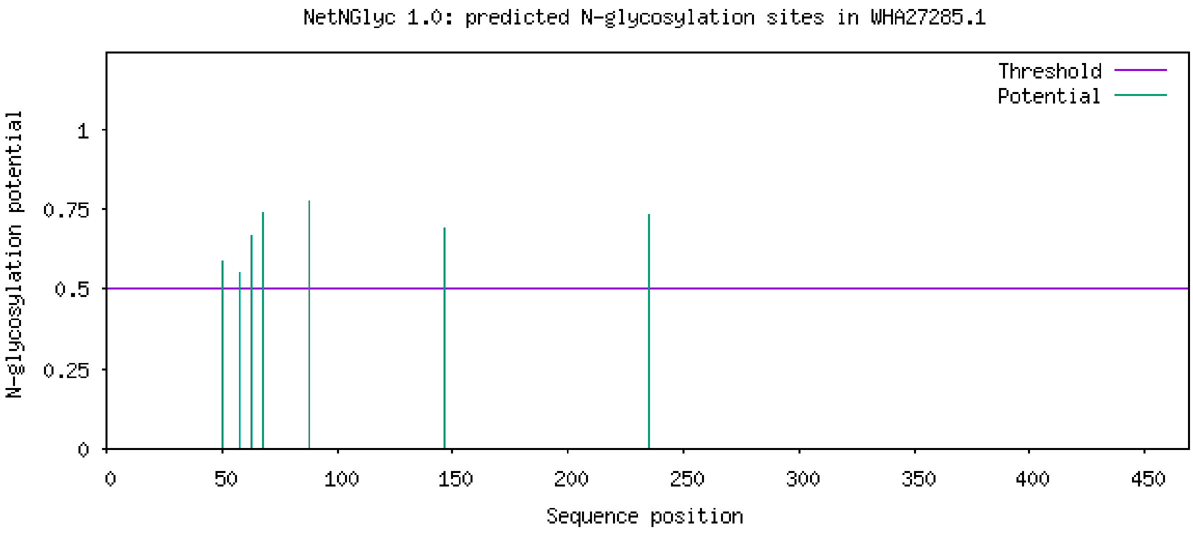

32. >UXW95954.1 hemagglutinin [Influenza A virus/pintail/Egypt/RA19853OP/2021]

```
Name:  UXW95954.1      Length:  567
MENIVLLLAIVSLVKSDQICIGYHANNSTEQVDTIMEKNVTVTHAQDILEKTHNGKLCDLNGVKPLILKDCSVAGWLLGN      80
PMCDEFIRVPEWSYIVERANPANDLCYPGSLNDYEELKHLLSRINHFEKILIIPKSSWPNHETSLGVSAACPYQGAPSFF     160
RNVVWLIKKNDAYPTIKISYNNTNREDLLILWGIHHSNNAEEQTNLYKNPTTYISVGTSTLNQRLVPKIATRSQVNGQRG     240
RMDFFWTILKPDDAIHFESNGNFIAPEYAYKIVKKGDSTIMKSGVEYGHCNTKCQTPVGAINSSMPFHNIHPLTIGECPK     320
YVKSNKLVLATGLRNSPLREKRRKRGLFGAIAGFIEGGWQGMVDGWYGYHHSNEQGSGYAADKESTQRAIDGVTNKVNSI     400
IDKMNTQFEAVGREFNNLERRIENLNKKMEDGFLDVWTYNAELLVLMENERTLDFHDSNVKNLYDKVRLQLRDNAKELGN     480
GCFEFYHKCDNECMESVRNGTYDYPQYSEEARLKREEISGVKLESIGTYQILSIYSTAASSLALAIMIAGLSLWMCSNGS     560
LQCRICI                                                                              640
.........................N............N.........................................      80
................................................................................     160
....................N...........................N...............................     240
.............................................................N..................     320
................................................................................     400
................................................................................     480
..................N..........................................................N..     560
.......                                                                              640

(Threshold=0.5)
----------------------------------------------------------------------
SeqName      Position  Potential   Jury    N-Glyc
                                 agreement result
----------------------------------------------------------------------
UXW95954.1    26 NNST   0.3686     (9/9)   --
UXW95954.1    27 NSTE   0.7796     (9/9)   +++
UXW95954.1    39 NVTV   0.7182     (9/9)   ++
UXW95954.1   181 NNTN   0.6100     (7/9)   +
UXW95954.1   209 NPTT   0.6336     (8/9)   +     WARNING: PRO-X1.
UXW95954.1   302 NSSM   0.5447     (6/9)   +
UXW95954.1   499 NGTY   0.5824     (6/9)   +
UXW95954.1   558 NGSL   0.6736     (9/9)   ++
----------------------------------------------------------------------
```

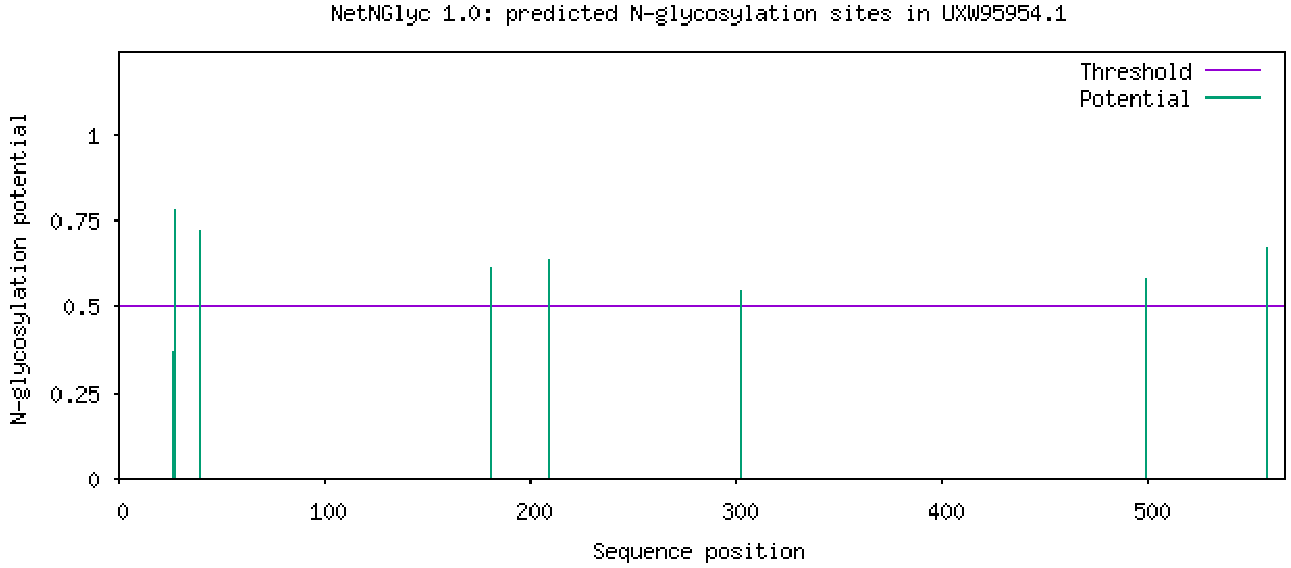

>UXW95956.1 neuraminidase [Influenza A virus/pintail/Egypt/RA19853OP/2021]

```
Name:  UXW95956.1       Length:  469
MNPNQRIITTGSICMVIGIVSLMLQIGNIISIWVSHSIQTGNQYQPEPCNQSIITYENNTWVNQTYVNISNTNFLAEQAV      80
TSVTLAGNSSLCPISGWAIYSKDNGIRIGSKGDVFVIREPFISCSHLECRTFFLTQGALLNDKHSNGTVKDRSPYRTLMS     160
CPVGEAPSPYNSRFESVAWSASACHDGISWLTIGISGPDNGAVAVLKYNGIITDTIKSWRNNILRTQESECACVNGSCFT     240
VMTDGPSNGQASYKIFKIEKGKVVKSVELNAPNYHYEECSCYPDAGEIMCVCRDNWHGSNRPWVSFNQNLEYQIGYICSG     320
VFGDNPRPNDGTGSCSPMSSNGAYGVKGFSFKYGNGVWIGRTKSTSSRSGFEMIWDPNGWTETDSSFSVKQDIVAITDWS     400
GYSGTFVQHPELTGLDCMRPCFWVELIRGRPKENTIWTSGSSISFCGVNSDTVGWSWPDGAELPFTIDK                480
.................................................N.......N....N....N............      80
.......N.........................................................N..............     160
..........................................................................N.....     240
................................................................................     320
................................................................................     400
.....................................................................                480

(Threshold=0.5)
----------------------------------------------------------------------
SeqName      Position  Potential   Jury    N-Glyc
                                agreement  result
----------------------------------------------------------------------
UXW95956.1     50 NQSI   0.5882     (8/9)    +
UXW95956.1     58 NNTW   0.5497     (6/9)    +
UXW95956.1     63 NQTY   0.6634     (9/9)    ++
UXW95956.1     68 NISN   0.7377     (9/9)    ++
UXW95956.1     88 NSSL   0.7724     (9/9)    +++
UXW95956.1    146 NGTV   0.6874     (9/9)    ++
UXW95956.1    235 NGSC   0.7320     (9/9)    ++
----------------------------------------------------------------------
```

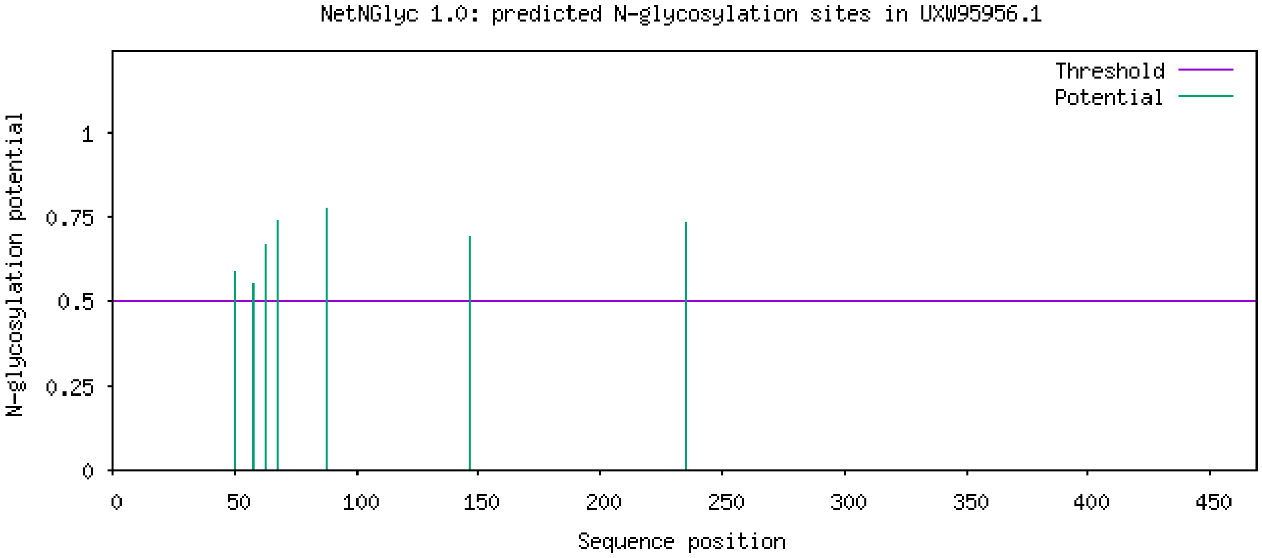

33. >WZL56519.1 hemagglutinin [Influenza A virus/feline/Texas/24-029329-02/2024]

```
Name:  WZL56519.1      Length:  567
MENIVLLLAIVSLVKSDQICIGYHANNSTEQVDTIMEKNVTVTHAQDILEKTHNGKLCDLNGVKPLILKDCSVAGWLLGN      80
PMCDEFIRVPEWSYIVERANPANDLCYPGSLNDYEELKHMLSRINHFEKIQIIPKSSWPNHETSLGVSAACPYQGAPSFF     160
RNVVWLIKKNDAYPTIKISYNNTNREDLLILWGIHHSNNAEEQTNLYKNPITYISVGTSTLNQRLAPKIATRSQVNGQRG     240
RMDFFWTILKPDDAIHFESNGNFIAPEYAYKIVKKGDSTIMKSGVEYGHCNTKCQTPVGAINSSMPFHNIHPLTIGECPK     320
YVKSNKLVLATGLRNSPLREKRRKRGLFGAIAGFIEGGWQGMVDGWYGYHHSNEQGSGYAADKESTQKAIDGVTNKVNSI     400
IDKMNTQFEAVGREFNNLERRIENLNKKMEDGFLDVWTYNAELLVLMENERTLDFHDSNVKNLYDKVRLQLRDNAKELGN     480
GCFEFYHKCDNECMESVRNGTYDYPQYSEEARLKREEISGVKLESVGTYQILSIYSTAASSLALAIMMAGLSLWMCSNGS     560
LQCRICI                                                                              640
.........................N............N.........................................      80
................................................................................     160
....................N...........................................................     240
.............................................................N..................     320
................................................................................     400
................................................................................     480
..................N..........................................................N..     560
.......                                                                              640

(Threshold=0.5)
----------------------------------------------------------------------
SeqName      Position  Potential   Jury    N-Glyc
                                 agreement result
----------------------------------------------------------------------
WZL56519.1    26 NNST   0.3686     (9/9)   --
WZL56519.1    27 NSTE   0.7796     (9/9)   +++
WZL56519.1    39 NVTV   0.7182     (9/9)   ++
WZL56519.1   181 NNTN   0.6100     (7/9)   +
WZL56519.1   302 NSSM   0.5447     (6/9)   +
WZL56519.1   499 NGTY   0.5826     (6/9)   +
WZL56519.1   558 NGSL   0.6827     (9/9)   ++
----------------------------------------------------------------------
```

NetNGlyc 1.0: predicted N-glycosylation sites in WZL56519.1

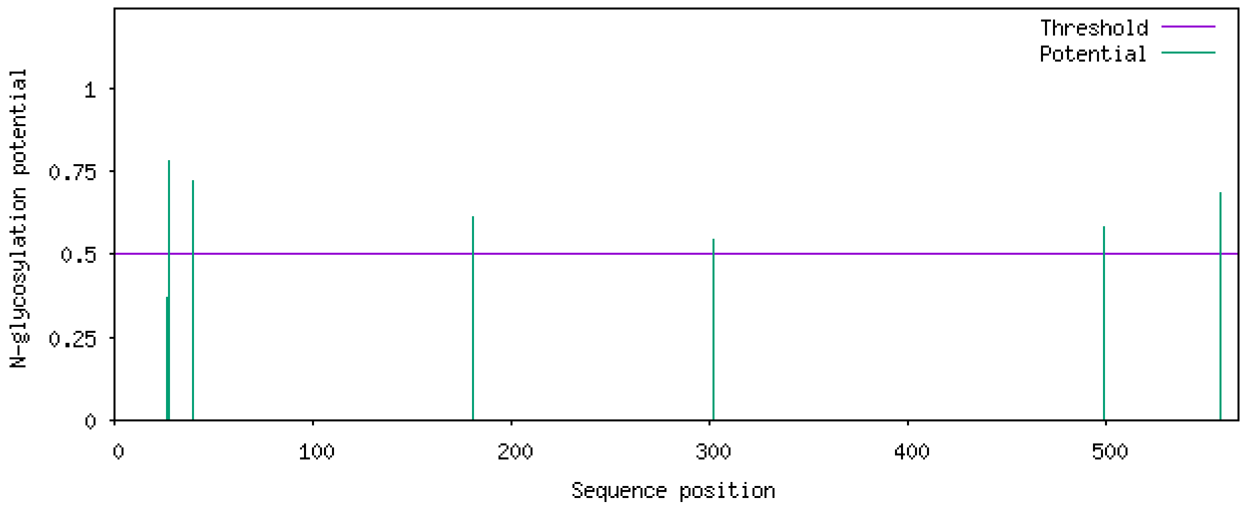

>WZL56521.1 neuraminidase [Influenza A virus/feline/Texas/24-029329-02/2024]

```
Name:  WZL56521.1       Length:  469
MNPNQKITTIGSICMVIGIVSLMLQIGNIISIWVSHSIQTGNQYQPEPCNQSIITYENNTWVNQTYINISSTNFLAEQAV      80
TSVTLAGNSSLCPISGWAIYSKDNGIRIGSKGDVFVIREPFISCSHLECRTFFLTQGALLNDKHSNGTVKDRSPYRTLMS     160
CPVGEAPSPYNSRFESVAWSASACHDGISWLTIGISGPDNGAVAVLKYNGIITDTIKSWRNNILRTQESECACVNGSCFT     240
VMTDGPSNGQASYKIFKIEKGKVVKSVEMNAPNYHYEECSCYPDAGDIMCVCRDNWHGSNRPWVSFNQNLEYQIGYICSG     320
IFGDNPRPNDGTGSCSPMPSNGAYGVKGFSFKYGNGVWIGRTKSTSSRSGFEMIWDPNGWTETDSSFSVKQDIVEITDWS     400
GYSGSFVQHPELTGLDCMRPCFWVELIRGRPKENTIWTSGSSISFCGVNSDTVGWSWPDGAELPFTIDK                480
.................................................N.......N....N....N............      80
.......N.........................................................N..............     160
..........................................................................N.....     240
................................................................................     320
................................................................................     400
.....................................................................                480

(Threshold=0.5)
----------------------------------------------------------------------
SeqName      Position  Potential   Jury    N-Glyc
                                 agreement result
----------------------------------------------------------------------
WZL56521.1     50 NQSI   0.5883     (8/9)   +
WZL56521.1     58 NNTW   0.5251     (5/9)   +
WZL56521.1     63 NQTY   0.6874     (9/9)   ++
WZL56521.1     68 NISS   0.7140     (9/9)   ++
WZL56521.1     88 NSSL   0.7724     (9/9)   +++
WZL56521.1    146 NGTV   0.6873     (9/9)   ++
WZL56521.1    235 NGSC   0.7321     (9/9)   ++
----------------------------------------------------------------------
```

NetNGlyc 1.0: predicted N-glycosylation sites in WZL56521.1

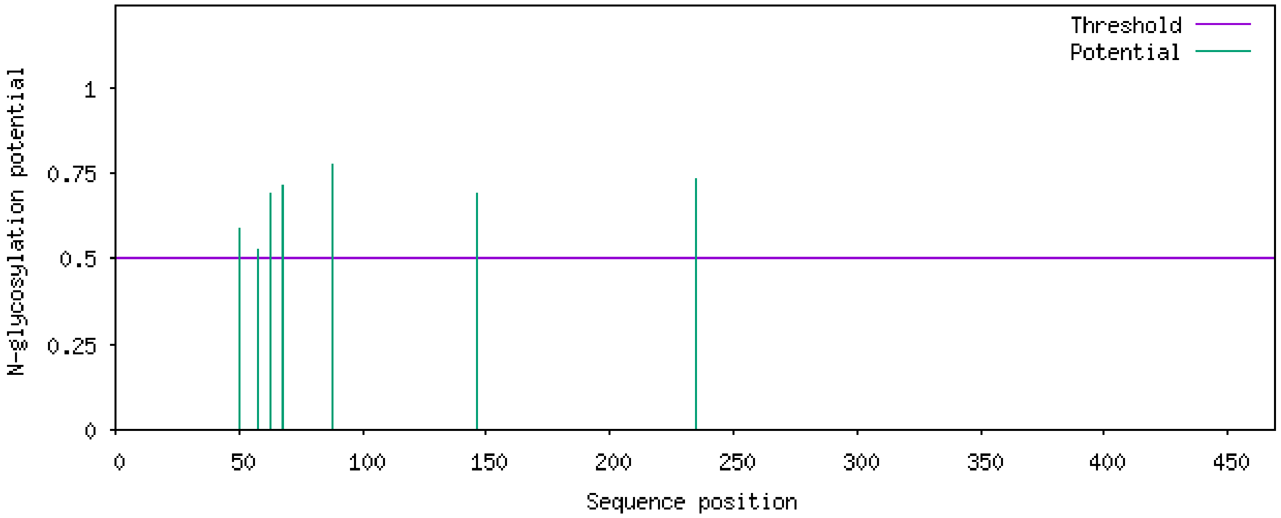

34. >WFD50829.1 hemagglutinin [Influenza A virus/harbor-seal/Maine/MME-22-185Pv-ns/2022]

```
Name:  WFD50829.1      Length:  567
MENIVLLLATVSLVKSDQICIGYHANNSTEQVDTIMEKNVTVTHAQDILEKTHNGKLCDLNGVKPLILKDCSVAGWLLGN      80
PMCDEFIRVPEWSYIVERANPANDLCYPGSLNDYEELKHMLSRINHFEKILIIPKSSWPNHETSLGVSAACSYQGAPSFF     160
RNVVWLIKKNDAYPTIKLSYNNTNREDLLILWGIHHSNNAEEQTNLYKNPTTYISVGTSTLNQRLTPKIATRSQVNGQRG     240
RMDFFWTILKPDDAIHFESNGNFIAPEYAYKIVKKGDSTIMKSGVEYGHCNTKCQTPVGAINSSMPFHNIHPLTIGECPK     320
YVKSNKLVLATGLRNSPLREKRRKRGLFGAIAGFIEGGWQGMVDGWYGYHHSNEQGSGYAADKESTQKAIDGVTNKVNSI     400
IDKMNTQFEAVGREFNNLERRIENLNKKMEDGFLDVWTYNAELLVLMENERTLDFHDSNVKNLYDKVRLQLRDNAKELGN     480
GCFEFYHKCDNECMESVRNGTYDYPQYSEEARLKREEISGVKLESVGTYQILSIYSTAASSLALAIMMAGLSLWMCSNGS     560
LQCRICI                                                                              640
..........................N...........N.........................................      80
................................................................................     160
....................N...........................N...............................     240
.............................................................N..................     320
................................................................................     400
................................................................................     480
..................N.............................................................N..     560
.......                                                                              640
```

(Threshold=0.5)

SeqName	Position		Potential	Jury agreement	N-Glyc result	
WFD50829.1	26	NNST	0.3687	(9/9)	--	
WFD50829.1	27	NSTE	0.7796	(9/9)	+++	
WFD50829.1	39	NVTV	0.7180	(9/9)	++	
WFD50829.1	181	NNTN	0.6146	(8/9)	+	
WFD50829.1	209	NPTT	0.6336	(8/9)	+	WARNING: PRO-X1.
WFD50829.1	302	NSSM	0.5448	(6/9)	+	
WFD50829.1	499	NGTY	0.5824	(6/9)	+	
WFD50829.1	558	NGSL	0.6827	(9/9)	++	

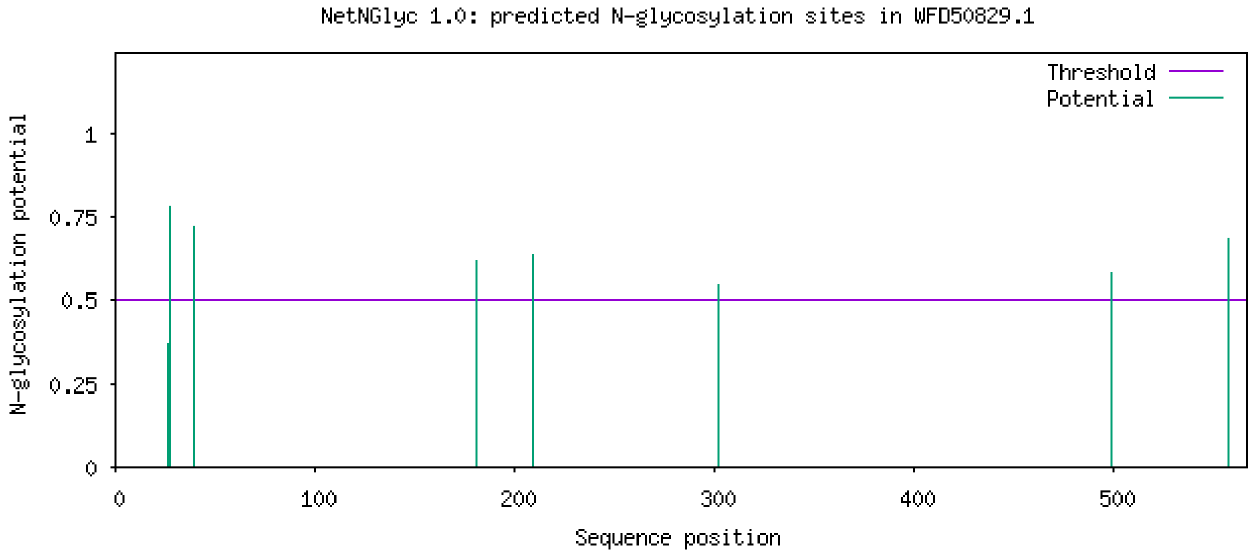

>WFD50830.1 neuraminidase [Influenza A virus/harbor-seal/Maine/MME-22-185Pv-ns/2022]

```
Name:  WFD50830.1      Length:  469
MNPNQKITTIGSICMVIGIVSLMLQIGNIISIWVSHSIQTGNQYQPEPCNQSIITYENNTWVNQTYVNISNTNFLAEQAV      80
TSVTLAGNSSLCPISGWAIYSKDNGIRIGSKGDVFVIREPFISCSHLECRTFFLTQGALLNDKHSNGTVKDRSPYRTLMS     160
CPVGEAPSPYNSRFESVAWSASACHDGISWLTIGISGPDNGAVAVLKYNGIITDTIKSWRNNILRTQESECACVNGSCFT     240
VMTDGPSNGQASYKIFKIEKGKVVKSVELNAPNYHYEECSCYPDAGEIMCVCRDNWHGSNRPWVSFNQNLEYQIGYICSG     320
VFGDNPRPNDGTGSCSPMSSNGAYGVKGFSFKYGNGVWIGRIKSTSSRSGFEMIWDPNGWTETDSSFSVKQDIVEITDWS     400
GYSGSFVQHPELTGLDCMRPCFWVELIRGRPKENTIWTSGSSISFCGVNSDTVGWSWPDGAELPFTIDK                480
.................................................N.......N....N....N............      80
.......N.........................................................N..............     160
..........................................................................N.....     240
................................................................................     320
................................................................................     400
.....................................................................                480
```

(Threshold=0.5)

SeqName	Position		Potential	Jury agreement	N-Glyc result
WFD50830.1	50	NQSI	0.5882	(8/9)	+
WFD50830.1	58	NNTW	0.5497	(6/9)	+
WFD50830.1	63	NQTY	0.6634	(9/9)	++
WFD50830.1	68	NISN	0.7376	(9/9)	++
WFD50830.1	88	NSSL	0.7723	(9/9)	+++
WFD50830.1	146	NGTV	0.6873	(9/9)	++
WFD50830.1	235	NGSC	0.7320	(9/9)	++

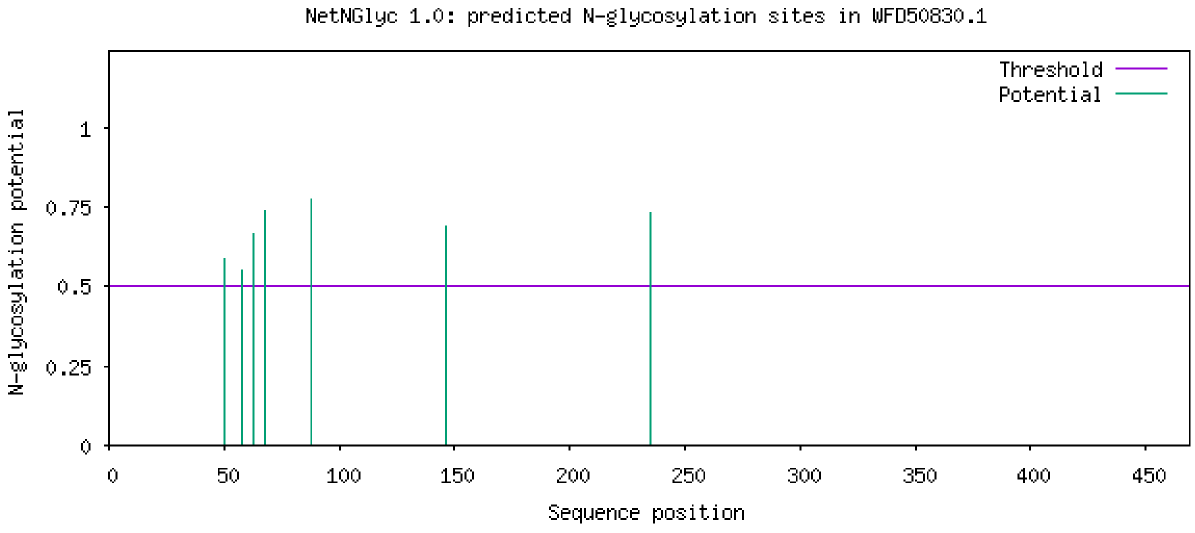

35. >WYX96735.1 hemagglutinin [Influenza A virus/cattle/Texas/56283/2024]

```
Name:  WYX96735.1       Length:  567
MENIVLLLAIVSLVKSDQICIGYHANNSTEQVDTIMEKNVTVTHAQDILEKTHNGKLCDLNGVKPLILKDCSVAGWLLGN      80
PMCDEFIRVPEWSYIVERANPANDLCYPGSLNDYEELKHMLSRINHFEKIQIIPKSSWPNHETSLGVSAACPYQGAPSFF     160
RNVVWLIKKNDAYPTIKISYNNTNREDLLILWGIHHSNNAEEQTNLYKNPITYISVGTSTLNQRLAPKIATRSQVNGQRG     240
RMDFFWTILKPDDAIHFESNGNFIAPEYAYKIVKKGDSTIMKSGVEYGHCNTKCQTPVGAINSSMPFHNIHPLTIGECPK     320
YVKSNKLVLATGLRNSPLREKRRKRGLFGAIAGFIEGGWQGMVDGWYGYHHSNEQGSGYAADKESTQKAIDGVTNKVNSI     400
IDKMNTQFEAVGREFNNLERRIENLNKKMEDGFLDVWTYNAELLVLMENERTLDFHDSNVKNLYDKVRLQLRDNAKELGN     480
GCFEFYHKCDNECMESVRNGTYDYPQYSEEARLKREEISGVKLESVGTYQILSIYSTAASSLALAIMMAGLSLWMCSNGS     560
LQCRICI                                                                              640
..........................N...........N.........................................      80
................................................................................     160
....................N...........................................................     240
.............................................................N..................     320
................................................................................     400
................................................................................     480
..................N..........................................................N..     560
.......                                                                              640

(Threshold=0.5)
----------------------------------------------------------------------
SeqName      Position  Potential   Jury    N-Glyc
                                 agreement result
----------------------------------------------------------------------
WYX96735.1     26 NNST   0.3686     (9/9)   --
WYX96735.1     27 NSTE   0.7796     (9/9)   +++
WYX96735.1     39 NVTV   0.7182     (9/9)   ++
WYX96735.1    181 NNTN   0.6100     (7/9)   +
WYX96735.1    302 NSSM   0.5447     (6/9)   +
WYX96735.1    499 NGTY   0.5826     (6/9)   +
WYX96735.1    558 NGSL   0.6827     (9/9)   ++
----------------------------------------------------------------------
```

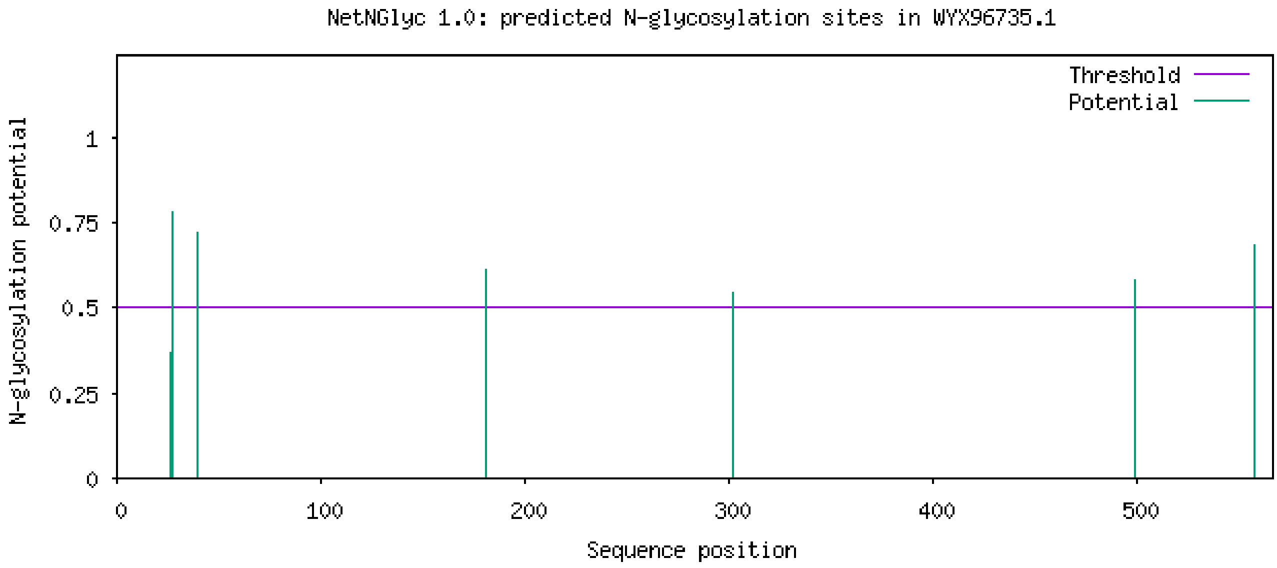

>WYX96737.1 neuraminidase [Influenza A virus/cattle/Texas/56283/2024]

```
Name:  WYX96737.1       Length:  469
MNPNQKITTIGSICMVIGIVSLMLQIGNIISIWVSHSIQTGNQYQPEPCNQSIITYENNTWVNQTYINISSTNFLAEQAV      80
TSVTLAGNSSLCPISGWAIYSKDNGIRIGSKGDVFVIREPFISCSHLECRTFFLTQGALLNDKHSNGTVKDRSPYRTLMS     160
CPVGEAPSPYNSRFESVAWSASACHDGISWLTIGISGPDNGAVAVLKYNGIITDTIKSWRNNILRTQESECACVNGSCFT     240
VMTDGPSNGQASYKIFKIEKGKVVKSVEMNAPNYHYEECSCYPDAGDIMCVCRDNWHGSNRPWVSFNQNLEYQIGYICSG     320
IFGDNPRPNDGTGSCSPMPSNGAYGVKGFSFKYGNGVWIGRTKSTSSRSGFEMIWDPNGWTETDSSFSVKQDIVEITDWS     400
GYSGSFVQHPELTGLDCMRPCFWVELIRGRPKENTIWTSGSSISFCGVNSDTVGWSWPDGAELPFTIDK     480
.................................................N.......N....N....N............      80
.......N.........................................................N..............     160
..........................................................................N.....     240
................................................................................     320
................................................................................     400
.....................................................................     480

(Threshold=0.5)
----------------------------------------------------------------------
SeqName      Position  Potential   Jury     N-Glyc
                                  agreement result
----------------------------------------------------------------------
WYX96737.1     50 NQSI   0.5883     (8/9)    +
WYX96737.1     58 NNTW   0.5251     (5/9)    +
WYX96737.1     63 NQTY   0.6874     (9/9)    ++
WYX96737.1     68 NISS   0.7140     (9/9)    ++
WYX96737.1     88 NSSL   0.7724     (9/9)    +++
WYX96737.1    146 NGTV   0.6873     (9/9)    ++
WYX96737.1    235 NGSC   0.7321     (9/9)    ++
----------------------------------------------------------------------
```

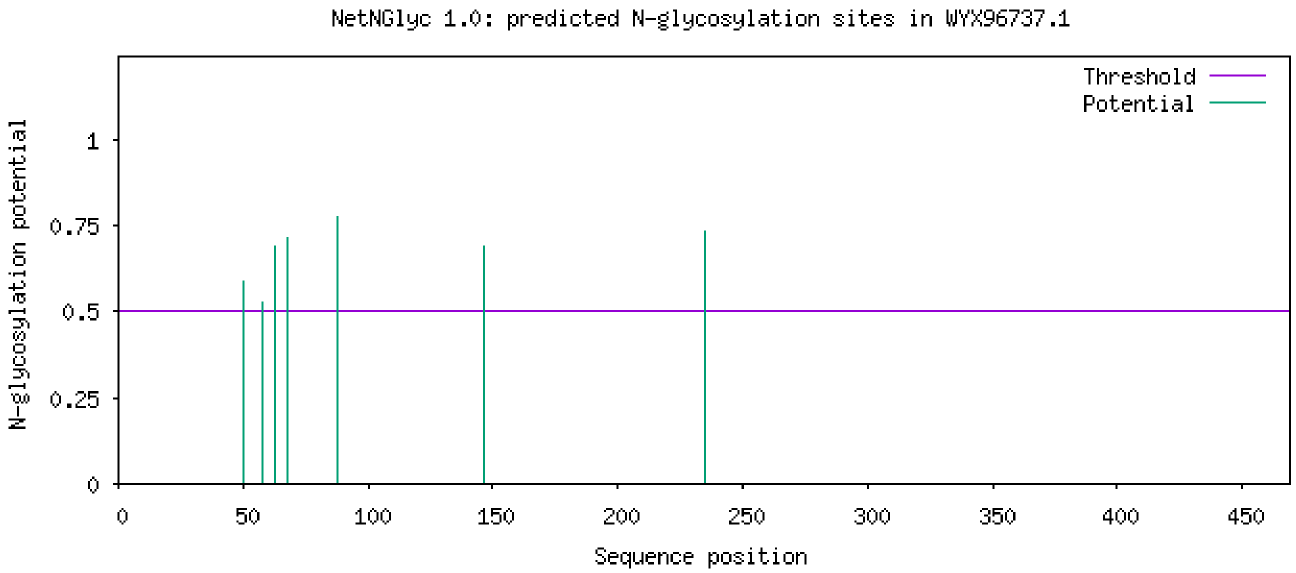

36. >XIJ00989.1 hemagglutinin [Influenza A virus/California/153/2024]

```
Name:  XIJ00989.1       Length:  567
MENIVLLLAIVSLVKSDQICIGYHANNSTEQVDTIMEKNVTVTHAQDILEKTHNGKLCDLNGVKPLILKDCSVAGWLLGN      80
PMCDEFIRVPEWSYIVERANPANGLCYPGSLNDYEELKHMLSRINHFEKIQIIPKSSWPNHETSLGVSAACPYQGAPSFF     160
RNVVWLIKKNDAYPTIKISYNNTNREDLLILWGIHHSNNAEEQTNLYKNPITYISVGTSTLNQRLAPKIATRSQVNGQRG     240
RMDFFWTILKPDDAIHFESNGNFIAPEYAYKIVKKGDSTIMKSGVEYGHCNTKCQTPVGAINSSMPFHNIHPLTIGECPK     320
YVKSNKLVLATGLRNNPLREKRRKRGLFGAIAGFIEGGWQGMVDGWYGYHHSNEQGSGYAADKESTQKAIDGVTNKVNSI     400
IDKMNTQFEAVGREFNNLERRIENLNKKMEDGFLDVWTYNAELLVLMENERTLDFHDSNVKNLYDKVRLQLRDNAKELGN     480
GCFEFYHKCDNECMESVRNGTYDYPQYSEEARLKREEISGVKLESVGTYQILSIYSTAASSLALAIMMAGLSLWMCSNGS     560
LQCRICI                                                                              640
..........................N...........N.........................................      80
................................................................................     160
....................N...........................................................     240
.............................................................N..................     320
................................................................................     400
................................................................................     480
..................N..........................................................N..     560
.......                                                                              640
```

```
(Threshold=0.5)
----------------------------------------------------------------------
SeqName      Position  Potential   Jury    N-Glyc
                                agreement  result
----------------------------------------------------------------------
XIJ00989.1     26 NNST   0.3687     (9/9)    --
XIJ00989.1     27 NSTE   0.7796     (9/9)    +++
XIJ00989.1     39 NVTV   0.7183     (9/9)    ++
XIJ00989.1    181 NNTN   0.6100     (7/9)    +
XIJ00989.1    302 NSSM   0.5447     (6/9)    +
XIJ00989.1    499 NGTY   0.5826     (6/9)    +
XIJ00989.1    558 NGSL   0.6829     (9/9)    ++
----------------------------------------------------------------------
```

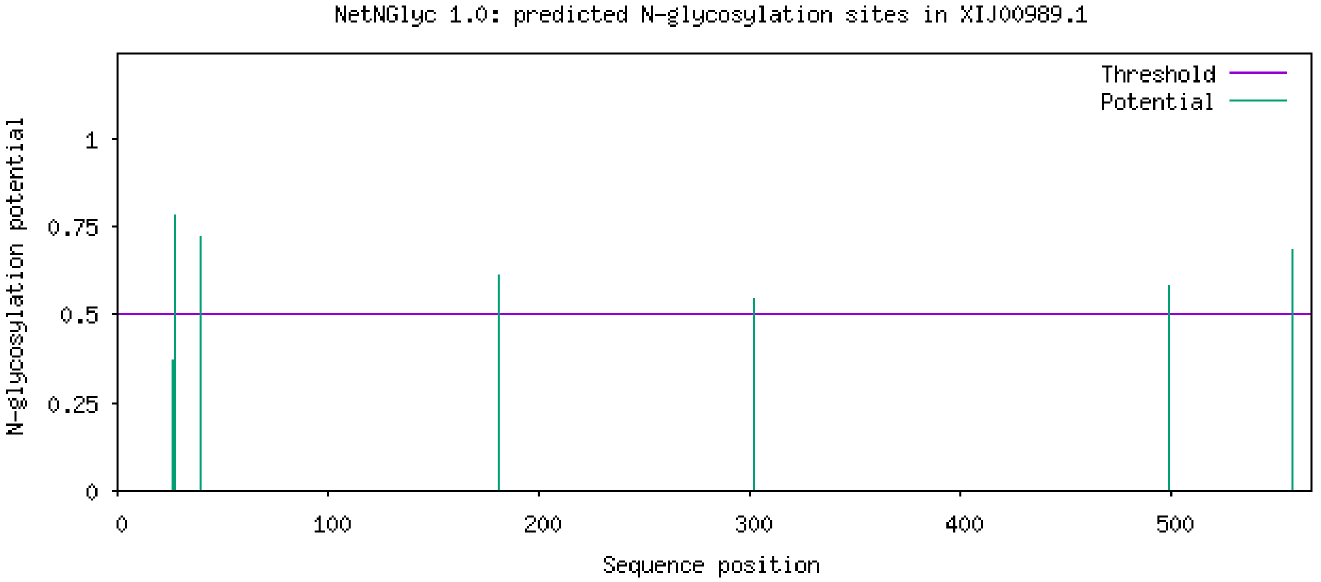

>XIJ00990.1 neuraminidase [Influenza A virusA/California/153/2024]

```
Name:  XIJ00990.1       Length:  469
MNPNQKITTIGSICMVIGIVSLMLQIGNIISIWVSHSIQTGNQYQPEPCNQSIITYENNTWVNQTYINISSTNFLAEQAV      80
TSVTLAGNSSLCPISGWAIYSKDNGIRIGSKGDVFVIREPFISCSHLECRTFFLTQGALLNDKHSNGTVKDRSPYRTLMS     160
CPVGEAPSPYNSRFESVAWSASACHDGISWLTIGISGPDNGAVAVLKYNGIITDTIKSWRNNILRTQESECACVNGSCFT     240
VMTDGPSNGQASYKIFKIEKGKVVKSVEMNAPNYHYEECSCYPDAGDIMCVCRDNWHGSNRPWVSFNQNLEYQIGYICSG     320
IFGDNPRPNDGTGSCSPMPSNGAYGVKGFSFKYGNGVWIGRTKSTSSRSGFEMIWDPNGWTETDSSFSVKQDIVEITDWS     400
GYSGSFVQHPELTGLDCMRPCFWVELIRGRPKENTIWTSGSSISFCGVNSDTVGWSWPDGAELPFTIDK                480
.................................................N.......N....N....N............      80
.......N.........................................................N..............     160
..........................................................................N.....     240
................................................................................     320
................................................................................     400
.....................................................................                480

(Threshold=0.5)
----------------------------------------------------------------------
SeqName      Position  Potential   Jury    N-Glyc
                                 agreement result
----------------------------------------------------------------------
XIJ00990.1     50 NQSI   0.5883     (8/9)   +
XIJ00990.1     58 NNTW   0.5251     (5/9)   +
XIJ00990.1     63 NQTY   0.6874     (9/9)   ++
XIJ00990.1     68 NISS   0.7140     (9/9)   ++
XIJ00990.1     88 NSSL   0.7724     (9/9)   +++
XIJ00990.1    146 NGTV   0.6873     (9/9)   ++
XIJ00990.1    235 NGSC   0.7321     (9/9)   ++
----------------------------------------------------------------------
```

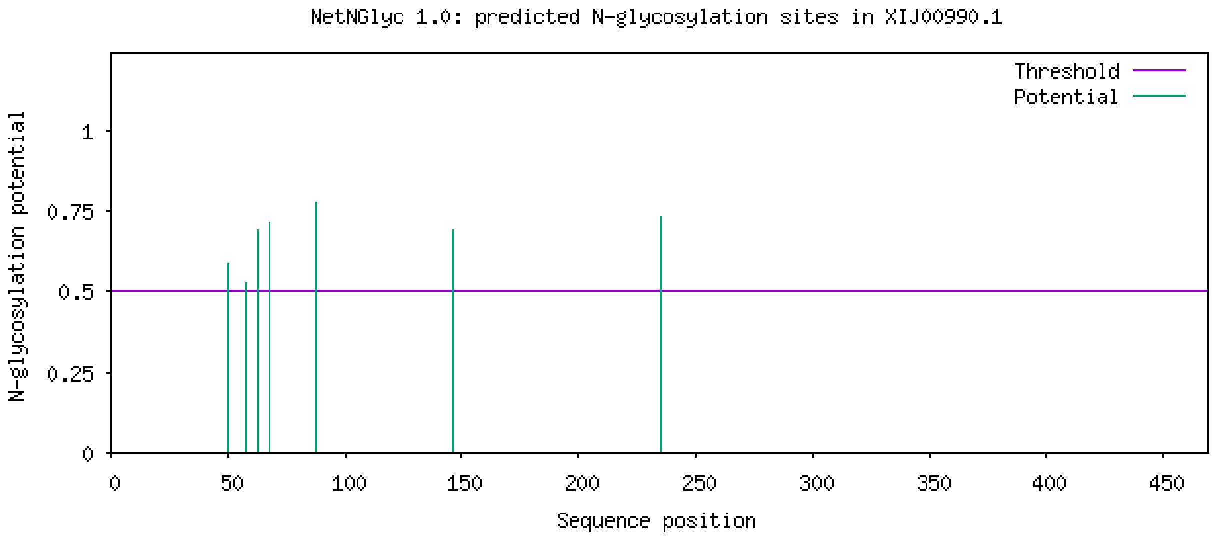

37. >XHG66702.2 hemagglutinin [Influenza A virus/cattle/Idaho/Broad_MF_006/2024]

```
Name:  XHG66702.2      Length:  567
MENIVLLLAIVSLVKSDQICIGYHANNSTEQVDTIMEKNVTVTHAQDILEKTHNGKLCDLNGVKPLILKDCSVAGWLLGN      80
PMCDEFIRVPEWSYIVERANPANDLCYPGSLNDYEELKHMLSRINHFEKIQIIPKSSWPNHETSLGVSAACPYQGAPSFF     160
RNVVWLIKKNDAYPTIKISYNNTNREDLLILWGIHHSNNAEEQTNLYKNPITYISVGTSTLNQRLAPKIATRSQVNGQRG     240
RMDFFWTILKPDDAIHFESNGNFIAPEYAYKIVKKGDSTIMKSGVEYGHCNTKCQTPVGAINSSMPFHNIHPLTIGECPK     320
YVKSNKLVLATGLRNSPLREKRRKRGLFGAIAGFIEGGWQGMVDGWYGYHHSNEQGSGYAADKESTQKAIDGVTNKVNSI     400
IDKMNTQFEAVGREFNNLERRIENLNKKMEDGFLDVWTYNAELLVLMENERTLDFHDSNVKNLYDKVRLQLRDNAKELGN     480
GCFEFYHKCDNECMESVRNGTYDYPQYSEEARLKREEISGVKLESVGTYQILSIYSTAASSLALAIMMAGLSLWMCSNGS     560
LQCRICI                                                                              640
.........................N............N.........................................      80
................................................................................     160
....................N...........................................................     240
.............................................................N..................     320
................................................................................     400
................................................................................     480
..................N..........................................................N..     560
.......                                                                              640

(Threshold=0.5)
----------------------------------------------------------------------
SeqName      Position  Potential   Jury    N-Glyc
                                 agreement result
----------------------------------------------------------------------
XHG66702.2    26 NNST   0.3686     (9/9)    --
XHG66702.2    27 NSTE   0.7796     (9/9)    +++
XHG66702.2    39 NVTV   0.7182     (9/9)    ++
XHG66702.2   181 NNTN   0.6100     (7/9)    +
XHG66702.2   302 NSSM   0.5447     (6/9)    +
XHG66702.2   499 NGTY   0.5826     (6/9)    +
XHG66702.2   558 NGSL   0.6827     (9/9)    ++
----------------------------------------------------------------------
```

NetNGlyc 1.0: predicted N-glycosylation sites in XHG66702.2

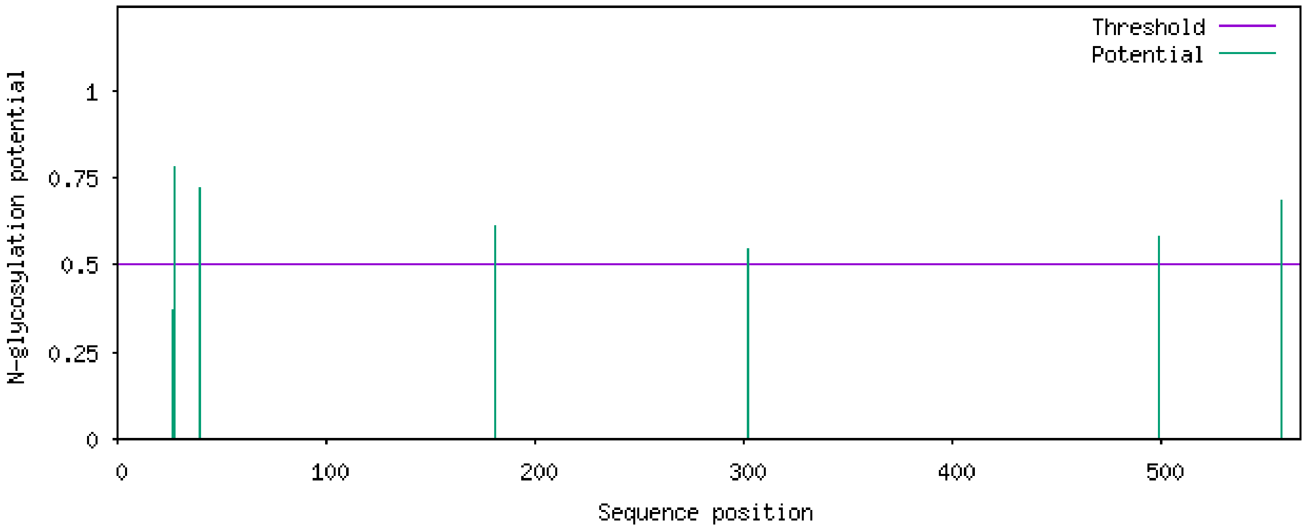

>XHG66704.2 neuraminidase, partial [Influenza A virus/cattle/Idaho/Broad_MF_006/2024

```
Name:  XHG66704.2      Length:  466
NQKITTIGSICMVIGIVSLMLQIGNIISIWVSHSIQTGNQYQPEPCNQSIITYENNTWVNQTYINISSTNFLAEQAVTSV      80
TLAGNSSLCPISGWAIYSKDNGIRIGSKGDVFVIREPFISCSHLECRTFFLTQGALLNDKHSNGTVKDRSPYRTLMSCPV     160
GEAPSPYNSRFESVAWSASACHDGISWLTIGISGPDNGAVAVLKYNGIITDTIKSWRNNILRTQESECACVNGSCFTVMT     240
DGPSNGQASYKIFKIEKGKVVKSVEMNAPNYHYEECSCYPDAGDIMCVCRDNWHGSNRPWVSFNQNLEYQIGYICSGIFG     320
DNPRPNDGTGSCSPMPSNGAYGVKGFSFKYGNGVWIGRTKSTSSRSGFEMIWDPNGWTETDSSFSVKQDIVEITDWSGYS     400
GSFVQHPELTGLDCMRPCFWVELIRGRPKENTIWTSGSSISFCGVNSDTVGWSWPDGAELPFTIDK                   480
..............................................N.......N....N....N...............      80
....N.........................................................N.................     160
.......................................................................N........     240
................................................................................     320
................................................................................     400
..................................................................                   480
```

(Threshold=0.5)

SeqName	Position	Potential	Jury agreement	N-Glyc result
XHG66704.2	47 NQSI	0.5892	(8/9)	+
XHG66704.2	55 NNTW	0.5264	(5/9)	+
XHG66704.2	60 NQTY	0.6879	(9/9)	++
XHG66704.2	65 NISS	0.7147	(9/9)	++
XHG66704.2	85 NSSL	0.7728	(9/9)	+++
XHG66704.2	143 NGTV	0.6880	(9/9)	++
XHG66704.2	232 NGSC	0.7323	(9/9)	++

NetNGlyc 1.0: predicted N-glycosylation sites in XHG66704.2

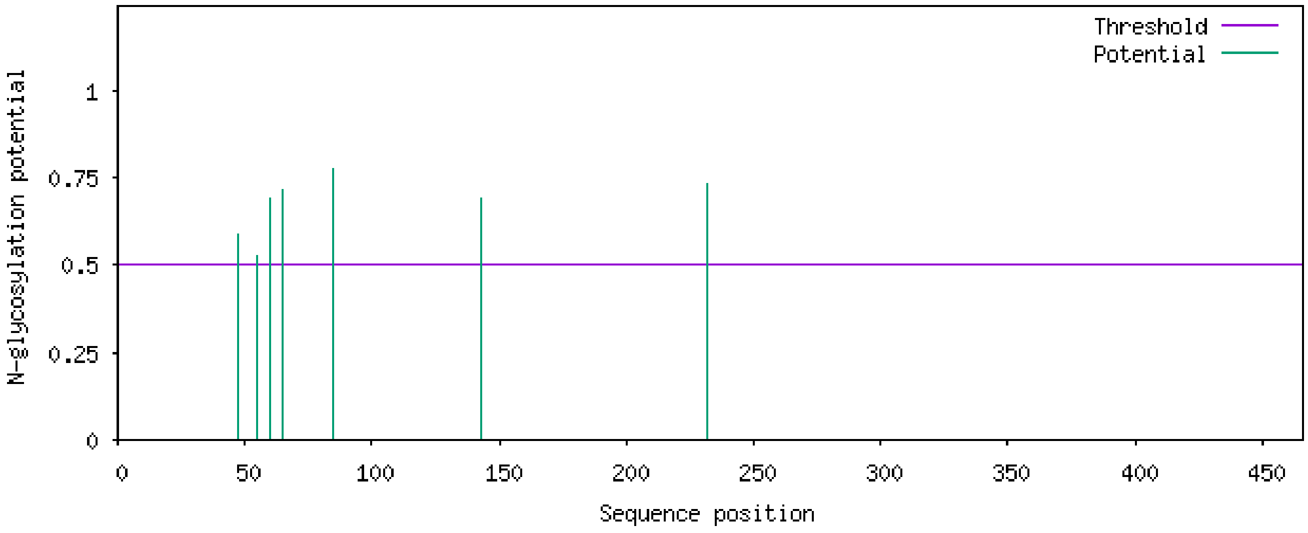

38. >XBS45858.1 hemagglutinin [Influenza A virus/Grackle/Texas/USL_047/2024]

```
Name:  XBS45858.1       Length:  567
MENIVLLLAIVSLVKSDQICIGYHANNSTEQVDTIMEKNVTVTHAQDILEKTHNGKLCDLNGVKPLILKDCSVAGWLLGN      80
PMCDEFIRVPEWSYIVERANPANDLCYPGSLNDYEELKHMLSRINHFEKIQIIPKSSWPNHETSLGVSAACPYQGAPSFF     160
RNVVWLIKKNDAYPTIKISYNNTNREDLLILWGIHHSNNAEEQTNLYKNPITYISVGTSTLNQRLAPKIATRSQVNGQRG     240
RMDFFWTILKPDDAIHFESNGNFIAPEYAYKIVKKGDSTIMKSGVEYGHCNTKCQTPVGAINSSMPFHNIHPLTIGECPK     320
YVKSNKLVLATGLRNSPLREKRRKRGLFGAIAGFIEGGWQGMVDGWYGYHHSNEQGSGYAADKESTQKAIDGVTNKVNSI     400
IDKMNTQFEAVGREFNNLERRIENLNKKMEDGFLDVWTYNAELLVLMENERTLDFHDSNVKNLYDKVRLQLRDNAKELGN     480
GCFEFYHKCDNECMESVRNGTYDYPQYSEEARLKREEISGVKLESVGTYQILSIYSTAASSLALAIMMAGLSLWMCSNGS     560
LQCRICI                                                                              640
.........................N...........N..........................................      80
................................................................................     160
....................N...........................................................     240
..............................................................N.................     320
................................................................................     400
................................................................................     480
..................N............................................................N..     560
.......                                                                              640

(Threshold=0.5)
----------------------------------------------------------------------
SeqName      Position  Potential   Jury     N-Glyc
                                  agreement result
----------------------------------------------------------------------
XBS45858.1    26 NNST   0.3686     (9/9)     --
XBS45858.1    27 NSTE   0.7796     (9/9)     +++
XBS45858.1    39 NVTV   0.7182     (9/9)     ++
XBS45858.1   181 NNTN   0.6100     (7/9)     +
XBS45858.1   302 NSSM   0.5447     (6/9)     +
XBS45858.1   499 NGTY   0.5826     (6/9)     +
XBS45858.1   558 NGSL   0.6827     (9/9)     ++
----------------------------------------------------------------------
```

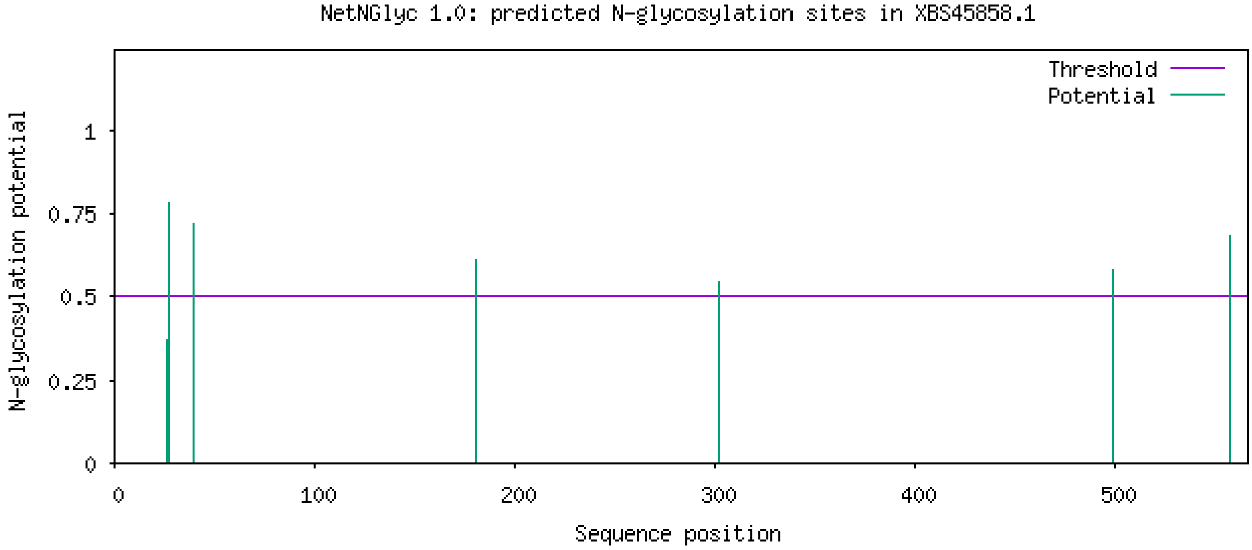

>XBS45860.1 neuraminidase [Influenza A virus/Grackle/Texas/USL_047/2024)]

```
Name:  XBS45860.1       Length:  469
MNPNQKITTIGSICMVIGIVSLMLQIGNIISIWVSHSIQTGNQYQPEPCNQSIITYENNTWVNQTYINISSTNFLAEQAV      80
TSVTLAGNSSLCPISGWAIYSKDNGIRIGSKGDVFVIREPFISCSHLECRTFFLTQGALLNDKHSNGTVKDRSPYRTLMS     160
CPVGEAPSPYNSRFESVAWSASACHDGISWLTIGISGPDNGAVAVLKYNGIITDTIKSWRNNILRTQESECACVNGSCFT     240
VMTDGPSNGQASYKIFKIEKGKVVKSVEMNAPNYHYEECSCYPDAGDIMCVCRDNWHGSNRPWVSFNQNLEYQIGYICSG     320
IFGDNPRPNDGTGSCSPMPSNGAYGVKGFSFKYGNGVWIGRTKSTSSRSGFEMIWDPNGWTETDSSFSVKQDIVEITDWS     400
GYSGSFVQHPELTGLDCMRPCFWVELIRGRPKENTIWTSGSSISFCGVNSDTVGWSWPDSAELPFTIDK                480
.................................................N.......N....N....N............      80
.......N.........................................................N..............     160
..........................................................................N.....     240
................................................................................     320
................................................................................     400
.....................................................................                480

(Threshold=0.5)
----------------------------------------------------------------------
SeqName      Position  Potential   Jury    N-Glyc
                                 agreement result
----------------------------------------------------------------------
XBS45860.1    50 NQSI   0.5882     (8/9)   +
XBS45860.1    58 NNTW   0.5251     (5/9)   +
XBS45860.1    63 NQTY   0.6874     (9/9)   ++
XBS45860.1    68 NISS   0.7140     (9/9)   ++
XBS45860.1    88 NSSL   0.7724     (9/9)   +++
XBS45860.1   146 NGTV   0.6873     (9/9)   ++
XBS45860.1   235 NGSC   0.7321     (9/9)   ++
----------------------------------------------------------------------
```

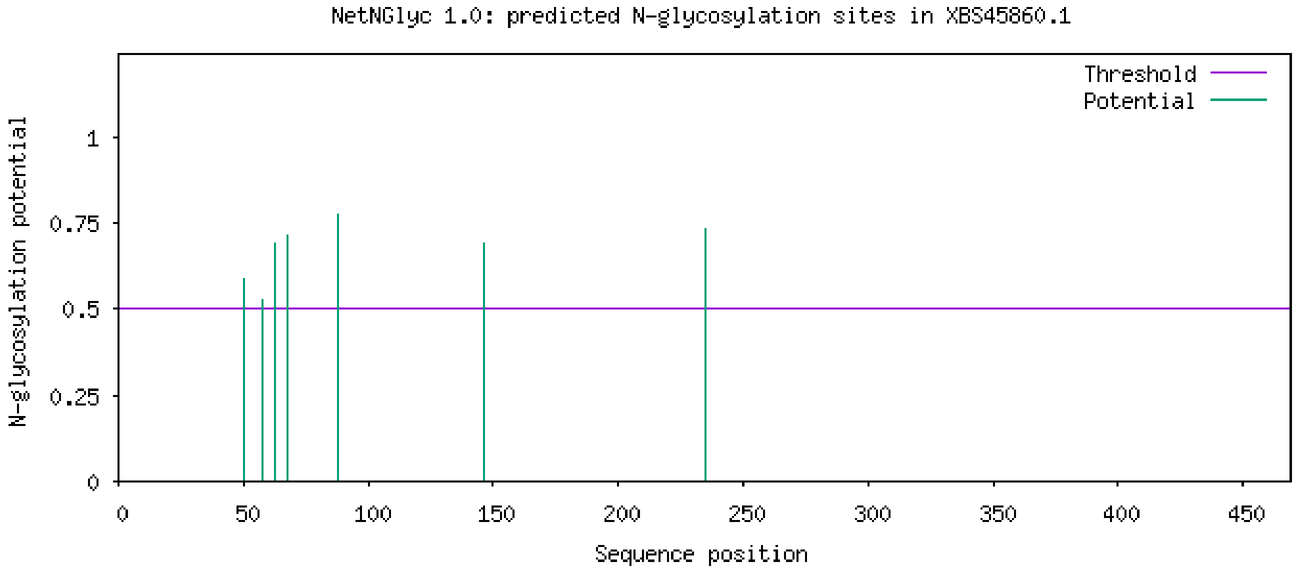

39. >XCO68626.1 hemagglutinin [Influenza A virus/Chicken/Idaho/24-013706-001-original/2024]

```
Name:   XCO68626.1        Length:   567
MENIVLLLAIVSLVKSDQICIGYHANNSTEQVDTIMEKNVTVTHAQDILEKTHNGKLCDLNGVKPLILKDCSVAGWLLGN      80
PMCDEFIRVPEWSYIVERANPANDLCYPGSLNDYEELKHMLSRINHFEKIQIIPKSSWPNHETSLGVSAACPYQGAPSFF     160
RNVVWLIKKNDAYPTIKISYNNTNREDLLILWGIHHSNNAEEQTNLYKNPITYISVGTSTLNQRLAPKIATRSQVNGQRG     240
RMDFFWTILKPDDAIHFESNGNFIAPEYAYKIVKKGDSTIMKSGVEYGHCNTKCQTPVGAINSSMPFHNIHPLTIGECPK     320
YVKSNKLVLATGLRNSPLREKRRKRGLFGAIAGFIEGGWQGMVDGWYGYHHSNEQGSGYAADKESTQKAIDGVTNKVNSI     400
IDKMNTQFEAVGREFNNLERRIENLNKKMEDGFLDVWTYNAELLVLMENERTLDFHDSNVKNLYDKVRLQLRDNAKELGN     480
GCFEFYHKCDNECMESVRNGTYDYPQYSEEARLKREEISGVKLESVGTYQILSIYSTAASSLALAIMMAGLSLWMCSNGS     560
LQCRICI                                                                              640
..........................N...........N.........................................      80
................................................................................     160
....................N...........................................................     240
.............................................................N..................     320
................................................................................     400
................................................................................     480
..................N..........................................................N..     560
.......                                                                              640

(Threshold=0.5)
----------------------------------------------------------------------
SeqName      Position  Potential   Jury     N-Glyc
                                 agreement  result
----------------------------------------------------------------------
XCO68626.1    26 NNST   0.3686     (9/9)     --
XCO68626.1    27 NSTE   0.7796     (9/9)     +++
XCO68626.1    39 NVTV   0.7182     (9/9)     ++
XCO68626.1   181 NNTN   0.6100     (7/9)     +
XCO68626.1   302 NSSM   0.5447     (6/9)     +
XCO68626.1   499 NGTY   0.5826     (6/9)     +
XCO68626.1   558 NGSL   0.6827     (9/9)     ++
----------------------------------------------------------------------
```

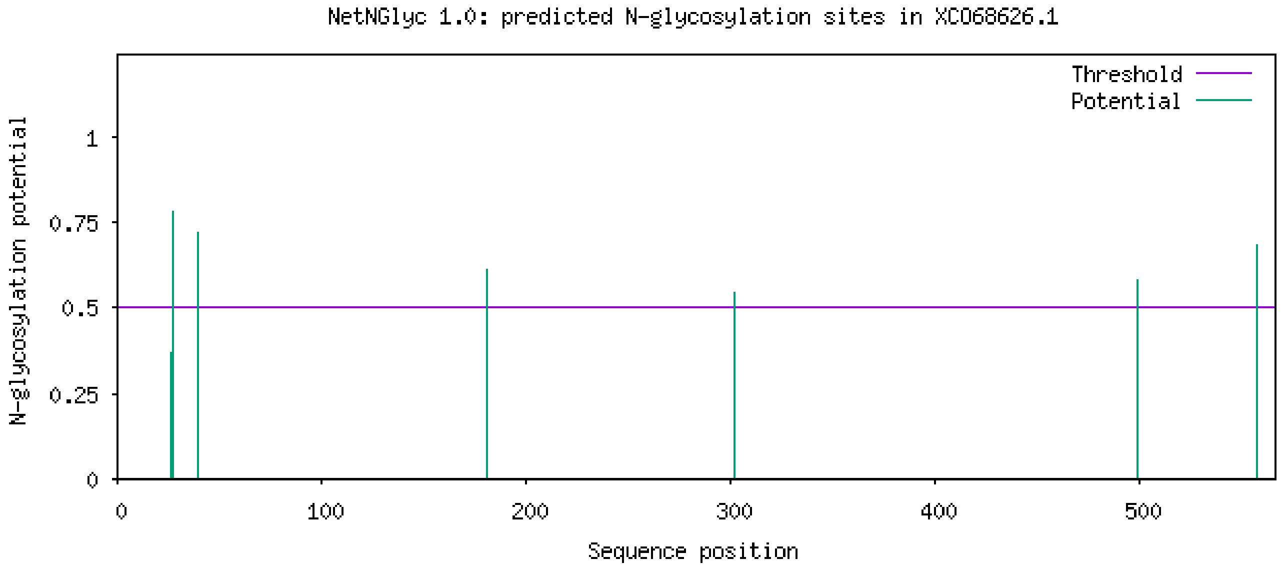

>XCO68628.1 neuraminidase [Influenza A virus/Chicken/Idaho/24-013706-001-original/2024]

```
Name:  XCO68628.1      Length:  469
MNPNQKITTIGSICMVIGIVSLMLQIGNIISIWVSHSIQTGNQYQPEPCNQSIITYENNTWVNQTYINISSTNFLAEQAV      80
TSVTLAGNSSLCPISGWAIYSKDNGIRIGSKGDVFVIREPFISCSHLECRTFFLTQGALLNDKHSNGTVKDRSPYRTLMS     160
CPVGEAPSPYNSRFESVAWSASACHDGISWLTIGISGPDNGAVAVLKYNGIITDTIKSWRNNILRTQESECACVNGSCFT     240
VMTDGPSNGQASYKIFKIEKGKVVKSVEMNAPNYHYEECSCYPDAGDIMCVCRDNWHGSNRPWVSFNQNLEYQIGYICSG     320
IFGDNPRPNDGTGSCSPMPSNGAYGVKGFSFKYGNGVWIGRTKSTSSRSGFEMIWDPNGWTETDSSFSVKQDIVEITDWS     400
GYSGSFVQHPELTGLDCMRPCFWVELIRGRPKENTIWTSGSSISFCGVNSDTVGWSWPDGAELPFTIDK                480
.................................................N.......N....N....N............      80
.......N.........................................................N..............     160
..........................................................................N.....     240
................................................................................     320
................................................................................     400
.....................................................................                480

(Threshold=0.5)
----------------------------------------------------------------------
SeqName      Position  Potential   Jury     N-Glyc
                                 agreement  result
----------------------------------------------------------------------
XCO68628.1     50 NQSI   0.5883     (8/9)   +
XCO68628.1     58 NNTW   0.5251     (5/9)   +
XCO68628.1     63 NQTY   0.6874     (9/9)   ++
XCO68628.1     68 NISS   0.7140     (9/9)   ++
XCO68628.1     88 NSSL   0.7724     (9/9)   +++
XCO68628.1    146 NGTV   0.6873     (9/9)   ++
XCO68628.1    235 NGSC   0.7321     (9/9)   ++
----------------------------------------------------------------------
```

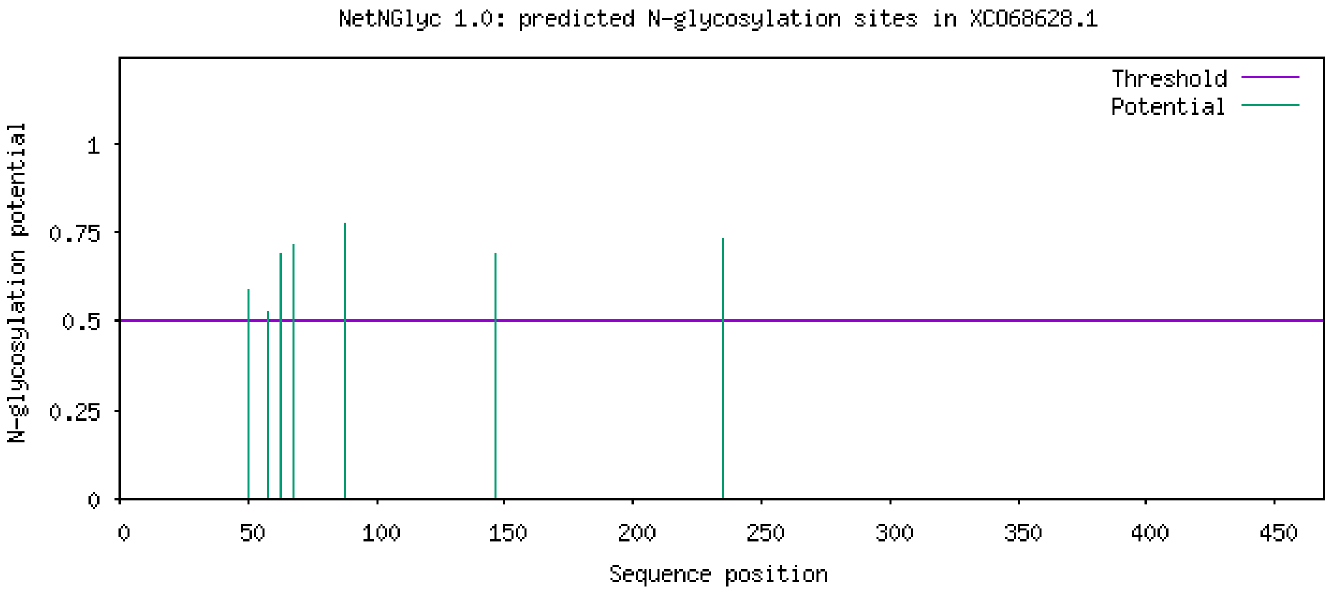

40. >XCZ64220.1 hemagglutinin [Influenza A virus/House Mouse/NM/24-014780-002-original/2024"]

```
Name:  XCZ64220.1      Length:  567
MENIVLLLAIVSLVKSDQICIGYHANNSTEQVDTIMEKNVTVTHAQDILEKTHNGKLCDLNGVKPLILKDCSVAGWLLGN     80
PMCDEFIRVPEWSYIVERANPANDLCYPGSLNDYEELKHMLSRINHFEKIQIIPKSSWPNHETSLGVSAACPYQGAPSFF    160
RNVVWLIKKNDAYPTIKISYNNTNREDLLILWGIHHSNNAEEQTNLYKNPITYISVGTSTLNQRLAPKIATRSQVNGQRG    240
RMDFFWTILKPDDAIHFESNGNFIAPEYAYKIVKKGDSTIMKSGVEYGHCNTKCQTPVGAINSSMPFHNIHPLTIGECPK    320
YVKSNKLVLATGLRNSPLREKRRKRGLFGAIAGFIEGGWQGMVDGWYGYHHSNEQGSGYAADKESTQKAIDGVTNKVNSI    400
IDKMNTQFEAVGREFNNLERRIENLNKKMEDGFLDVWTYNAELLVLMENERTLDFHDSNVKNLYDKVRLQLRDNAKELGN    480
GCFEFYHKCDNECMESVRNGTYDYPQYSEEARLKREEISGVKLESVGTYQILSIYSTAASSLALAIMMAGLSLWMCSNGS    560
LQCRICI                                                                             640
..........................N...........N.........................................     80
................................................................................    160
....................N...........................................................    240
.............................................................N..................    320
................................................................................    400
................................................................................    480
..................N...........................................................N..    560
.......                                                                             640

(Threshold=0.5)
----------------------------------------------------------------------
SeqName      Position  Potential   Jury    N-Glyc
                                 agreement result
----------------------------------------------------------------------
XCZ64220.1     26 NNST   0.3686    (9/9)   --
XCZ64220.1     27 NSTE   0.7796    (9/9)   +++
XCZ64220.1     39 NVTV   0.7182    (9/9)   ++
XCZ64220.1    181 NNTN   0.6100    (7/9)   +
XCZ64220.1    302 NSSM   0.5447    (6/9)   +
XCZ64220.1    499 NGTY   0.5826    (6/9)   +
XCZ64220.1    558 NGSL   0.6827    (9/9)   ++
----------------------------------------------------------------------
```

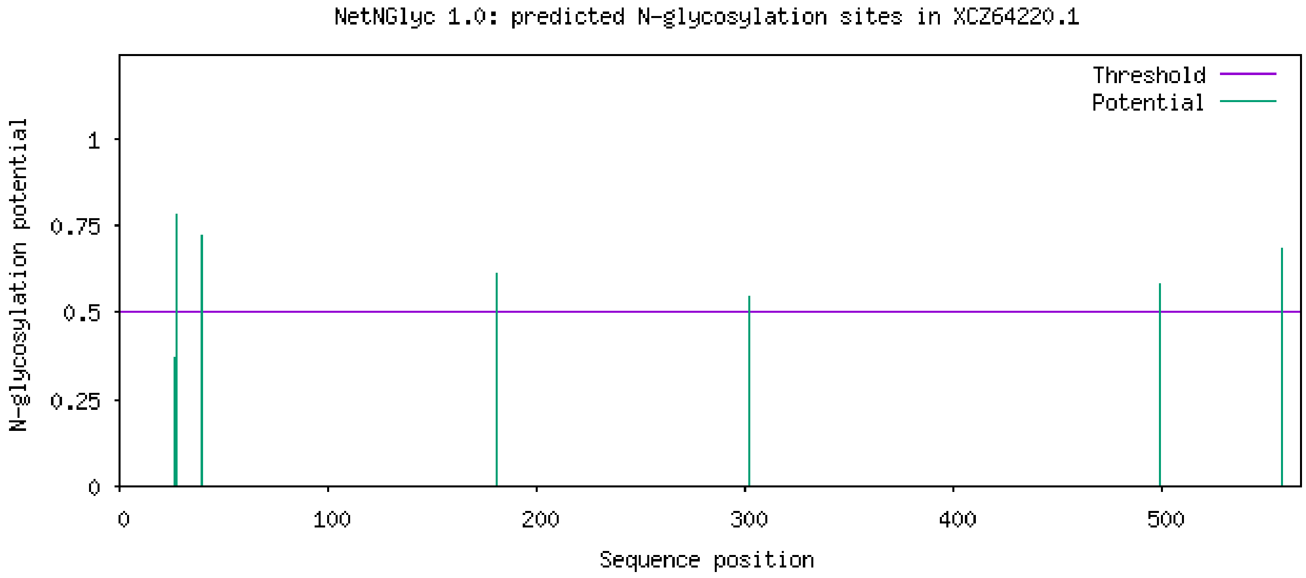

>XCZ64222.1 neuraminidase [Influenza A virus/House Mouse/NM/24-014780-002-original/2024)]

```
Name:  XCZ64222.1       Length:  469
MNPNQKITTIGSICMVIGIVSLMLQIGNIISIWVSHSIQTGNQYQPEPCNQSIITYENNTWVNQTYINISNTNFLAEQAV      80
TSVTLAGNSSLCPISGWAIYSKDNGIRIGSKGDVFVIREPFISCSHLECRTFFLTQGALLNDKHSNGTVKDRSPYRTLMS     160
CPVGEAPSPYNSRFESVAWSASACHDGISWLTIGISGPDNGAVAVLKYNGIITDTIKSWRNNILRTQESECACVNGSCFT     240
VMTDGPSNGQASYRIFKIEKGKVVKSVEMNAPNYHYEECSCYPDAGDIMCVCRDNWHGSNRPWVSFNQNLEYQIGYICSG     320
IFGDNPRPNDGTGSCSPMPSNGAYGVKGFSFKYGNGVWIGRTKSTSSRSGFEMIWDPNGWTETDSSFSVKQDIVEITDWS     400
GYSGSFVQHPELTGLDCMRPCFWVELIRGRPKENTIWTSGSSISFCGVNSDTVGWSWPDGAELPFTIDK                480
.................................................N.......N....N....N............      80
.......N.........................................................N..............     160
..........................................................................N.....     240
................................................................................     320
................................................................................     400
.....................................................................                480

(Threshold=0.5)
----------------------------------------------------------------------
SeqName       Position  Potential   Jury    N-Glyc
                                 agreement  result
----------------------------------------------------------------------
XCZ64222.1     50 NQSI   0.5883     (8/9)    +
XCZ64222.1     58 NNTW   0.5253     (5/9)    +
XCZ64222.1     63 NQTY   0.6762     (9/9)    ++
XCZ64222.1     68 NISN   0.6866     (7/9)    +
XCZ64222.1     88 NSSL   0.7724     (9/9)    +++
XCZ64222.1    146 NGTV   0.6874     (9/9)    ++
XCZ64222.1    235 NGSC   0.7320     (9/9)    ++
----------------------------------------------------------------------
```

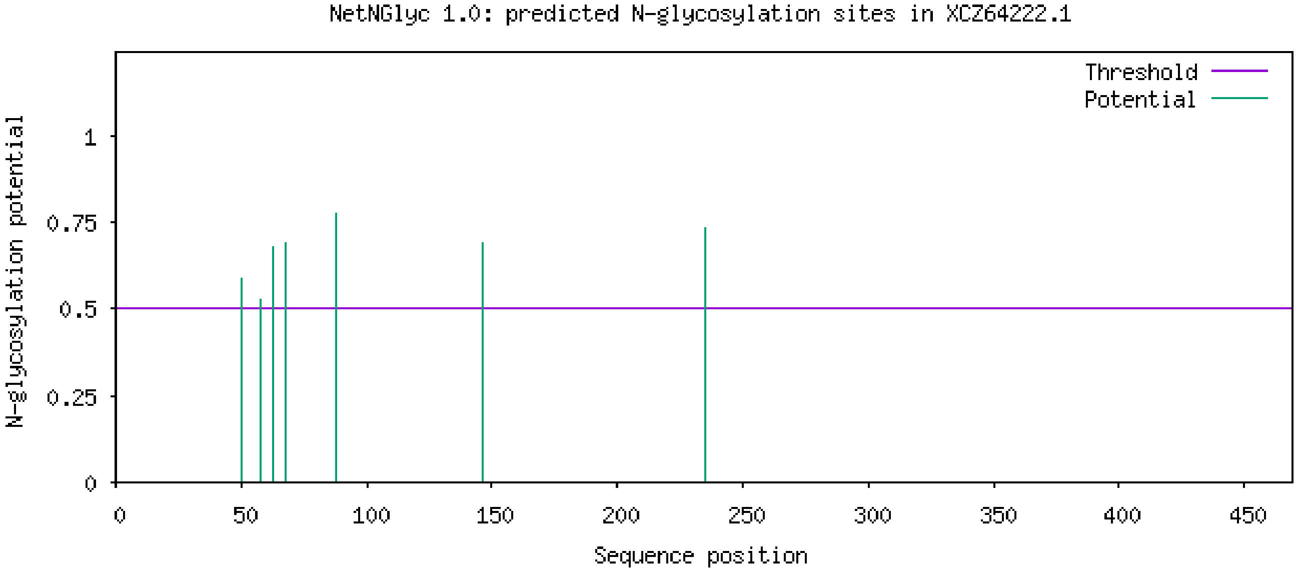

41. >BEP19168.1 haemagglutinin [Influenza A virus/white-tailed-eagle/Japan/OU-1/2022]

```
Name:  BEP19168.1       Length:  567
MENIVLLLAIISLVKSDQICIGYHANNSTEQVDTIMEKNVTVTHAQDILEKAHNGKLCDLNGVKPLILKDCSVAGWLLGN      80
PMCDEFIRVPEWSYIVERANPANDLCYPGSLNDYEELKHLLSRINHFEKILIIPKSSWPNHETSLGVSAACPYQGAPSFF     160
RNVVWLIKKNDAYPTIKISYNNTNREDLLILWGIHHSNNAEEQTNLYKNPTTYISVGTSTLNQRLVPKIATRSQVNGQRG     240
RMDFFWTILKPDDAIHFESNGNFIAPEYAYKIVKKGDSTIMKSGVEYGHCNTKCQTPVGAINSSMPFHNIHPLTIGECPK     320
YVKSNKLVLATGLRNSPLRERRRKRGLFGAIAGFIEGGWQGMVDGWYGYHHSNEQGSGYAADKESTQKAIDGVTNKVNSI     400
IDKMNTQFEAVGREFNNLERRIENLNKKMEDGFLDVWTYNAELLVLMENERTLDFHDSNVKNLYDKVRLQLRDNAKELGN     480
GCFEFYHKCDDECMESVRNGTYDYPQYSEEARLKREEISGVKLESIGTYQILSIYSTAASSLALAIMMAGLSLWMCSNGS     560
LQCRICI                                                                              640
..........................N...........N.........................................      80
................................................................................     160
....................N...........................N...............................     240
.............................................................N..................     320
................................................................................     400
................................................................................     480
..................N..........................................................N..     560
.......                                                                              640

(Threshold=0.5)
----------------------------------------------------------------------
SeqName      Position  Potential   Jury    N-Glyc
                                 agreement result
----------------------------------------------------------------------
BEP19168.1    26 NNST   0.3684     (9/9)   --
BEP19168.1    27 NSTE   0.7794     (9/9)   +++
BEP19168.1    39 NVTV   0.7182     (9/9)   ++
BEP19168.1   181 NNTN   0.6100     (7/9)   +
BEP19168.1   209 NPTT   0.6336     (8/9)   +    WARNING: PRO-X1.
BEP19168.1   302 NSSM   0.5447     (6/9)   +
BEP19168.1   499 NGTY   0.5806     (6/9)   +
BEP19168.1   558 NGSL   0.6826     (9/9)   ++
----------------------------------------------------------------------
```

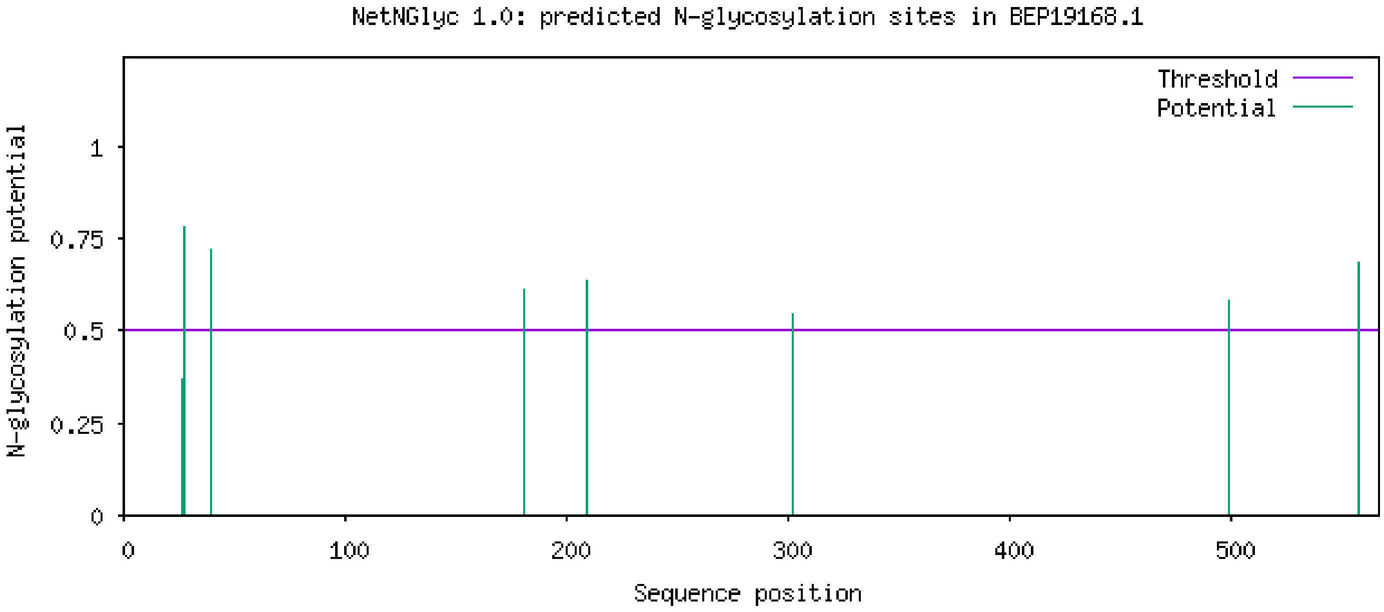

>BEP19170.1 neuraminidase [Influenza A virus/white-tailed eagle/Japan/OU-1/2022]

```
Name:  BEP19170.1       Length:  469
MNPNQRIITTGSICMVIGIVSLMLQIGNIISIWVSHSIQTGNQYQPEPCNQSIIAYENNTWINQTYVNINNTNFLAEQAV      80
TSVTLAGNSSLCPISGWAIYSKDNGIRIGSKGDVFVIREPFISCSHLECRTFFLTQGALLNDKHSNGTVKDRSPYRTLMS     160
CPVGEAPSPYNSRFESVAWSASACHDGISWLTIGISGPDNGAVAVLKYNGIITDTIKSWRNNILRTQESECACVNGSCFT     240
VMTDGPSNGQASYKIFKIEKGKVVKSVELNAPNYHYEECSCYPDAGEIMCVCRDNWHGSNRPWVSFNQNLEYQIGYICSG     320
VFGDNPRPNDGTGSCSPMSSNGAYGVKGFSFKYGNGVWIGRTKSTSSRSGFEMIWDPNGWTETDSSFSVKQDIVAITDLS     400
GYSGTFVQHPELTGLDCMRPCFWVELIRGRPKENTVWTSGSSISFCGVNSDTVGWSWPDGAELPFTIDK                480
.................................................N.......N....N......N..........      80
.......N.........................................................N..............     160
..........................................................................N.....     240
................................................................................     320
................................................................................     400
.....................................................................                480

(Threshold=0.5)
----------------------------------------------------------------------
SeqName      Position  Potential   Jury    N-Glyc
                                 agreement result
----------------------------------------------------------------------
BEP19170.1    50 NQSI   0.6061     (9/9)   ++
BEP19170.1    58 NNTW   0.5741     (8/9)   +
BEP19170.1    63 NQTY   0.5942     (9/9)   ++
BEP19170.1    70 NNTN   0.6889     (8/9)   +
BEP19170.1    88 NSSL   0.7724     (9/9)   +++
BEP19170.1   146 NGTV   0.6873     (9/9)   ++
BEP19170.1   235 NGSC   0.7320     (9/9)   ++
----------------------------------------------------------------------
```

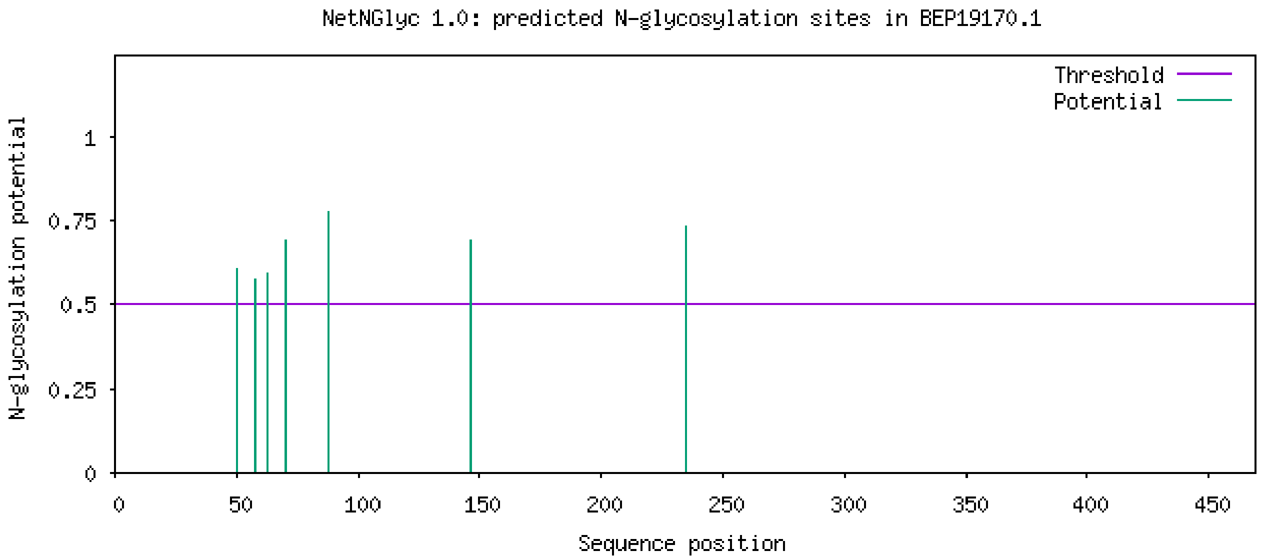

42. >BDH01750.1 haemagglutinin [Influenza A virus/chicken/Ehime/TU11-2-24,25/2022]

```
Name:  BDH01750.1       Length:  567
MENIVLLLAIVSLVKSDQICIGYHANNSTEQVDTIMEKNVTVTHAQDILEKTHNGKLCDLNGVKPLILKDCSVAGWLLGN      80
PMCDEFIRVPEWSYIVEKANPTNDLCYPGSLNDYEELKHLLSRINHFEKILIIPKNSWPNHETSLGVSAACPYQGAPSFF     160
RNVVWLIKKNDAYPTIKISYNNTNREDLLILWGIHHSNNAEEQTDLYKNPTTYISVGTSTLNQRLVPKIATRSQVNGQRG     240
RMDFFWTILKPDDAIHFESNGNFIAPEYAYKIVKKGDSTIMKSGVEYGHCNTKCQTPVGAINSSMPFHNIHPLTIGECPK     320
YVKSNKLVLATGLRNSPLRERRRKRGLFGAIAGFIEGGWQGMVDGWYGYHHSNEQGSGYAADKESTQKAIDGVTNKVNSI     400
IDKMNTQFEAVGREFNNLERRIENLNKKMEDGFLDVWTYNAELLVLMENERTLDFHDSNVKNLYDKVRLQLRDNAKELGN     480
GCFEFYHKCDNECMESVRNGTYDYPQYSEEARLKREEISGVKLESIGTYQILSIYSTAASSLALAIMMAGLSLWMCSNGS     560
LQCRICI                                                                              640
..........................N...........N.........................................      80
...................N............................................................     160
....................N...........................N...............................     240
.............................................................N..................     320
................................................................................     400
................................................................................     480
..................N..........................................................N..     560
.......                                                                              640

(Threshold=0.5)
----------------------------------------------------------------------
SeqName      Position  Potential   Jury    N-Glyc
                                 agreement result
----------------------------------------------------------------------
BDH01750.1    26 NNST   0.3686     (9/9)   --
BDH01750.1    27 NSTE   0.7796     (9/9)   +++
BDH01750.1    39 NVTV   0.7180     (9/9)   ++
BDH01750.1   100 NPTN   0.5232     (7/9)   +     WARNING: PRO-X1.
BDH01750.1   181 NNTN   0.6099     (7/9)   +
BDH01750.1   209 NPTT   0.5748     (8/9)   +     WARNING: PRO-X1.
BDH01750.1   302 NSSM   0.5446     (6/9)   +
BDH01750.1   499 NGTY   0.5824     (6/9)   +
BDH01750.1   558 NGSL   0.6828     (9/9)   ++
----------------------------------------------------------------------
```

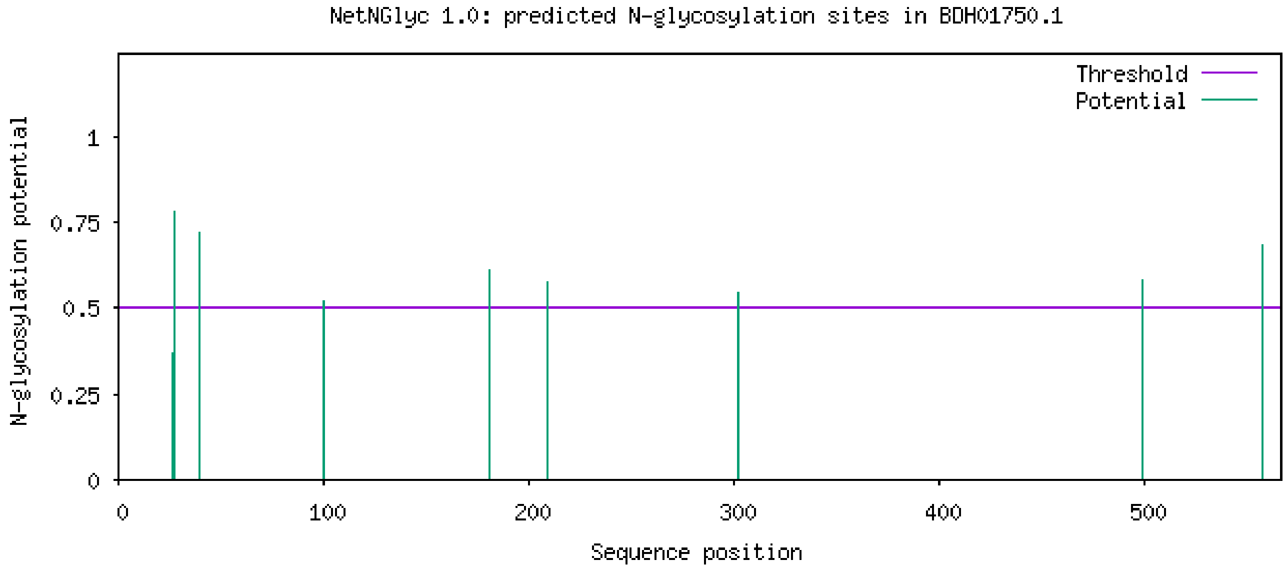

>BDH01752.1 neuraminidase [Influenza A virus/chicken/Ehime/TU11-2-24,25/2022]

```
Name:  BDH01752.1      Length:  469
MNPNQKITTIGSICMAIGIVSLMLQIGNIISIWVSHSIQTGNQYQPEPCNQSIITYENNTWVNQTYVNISNTNFLAEQAV      80
TSVTLAGNSSLCPISGWAIYSKDNGIRIGSKGDVFVIREPFISCSHLECRIFFLTQGALLNDKHSNGTVEDRSPYRTLMS     160
CPVGVAPSPYNSRFESVAWSASACHDGISWLTIGISGPDNEAVAVLKYNGIITDTIKSWRNNILRTQESECACVNGSCFT     240
VMTDGPSNGQASYKIFKIEKGKVVKSVELNAPNYHYEECSCYPNAGEIMCVCRDNWHGSNRPWVSFNQNLEYQIGYICSG     320
VFGDNPRPNDGTGSCNPMSSNGAYGVKGFSFKYGNGVWIGRTKSTSSRSGFEMIWDPNGWTETDSSFSVKQDIVAITDWS     400
GYSGSFVQHPELTGLDCMRPCFWVELIRGRPKENTIWTSGSSISFCGVNSDTVGWSWPDGAELPFTIDK                480
.................................................N.......N....N....N............      80
.......N.........................................................N..............     160
..........................................................................N.....     240
................................................................................     320
................................................................................     400
.....................................................................                480

(Threshold=0.5)
----------------------------------------------------------------------
SeqName      Position  Potential   Jury    N-Glyc
                                 agreement result
----------------------------------------------------------------------
BDH01752.1     50 NQSI   0.5882     (8/9)    +
BDH01752.1     58 NNTW   0.5496     (6/9)    +
BDH01752.1     63 NQTY   0.6634     (9/9)    ++
BDH01752.1     68 NISN   0.7377     (9/9)    ++
BDH01752.1     88 NSSL   0.7723     (9/9)    +++
BDH01752.1    146 NGTV   0.7377     (9/9)    ++
BDH01752.1    235 NGSC   0.7321     (9/9)    ++
----------------------------------------------------------------------
```

NetNGlyc 1.0: predicted N-glycosylation sites in BDH01752.1

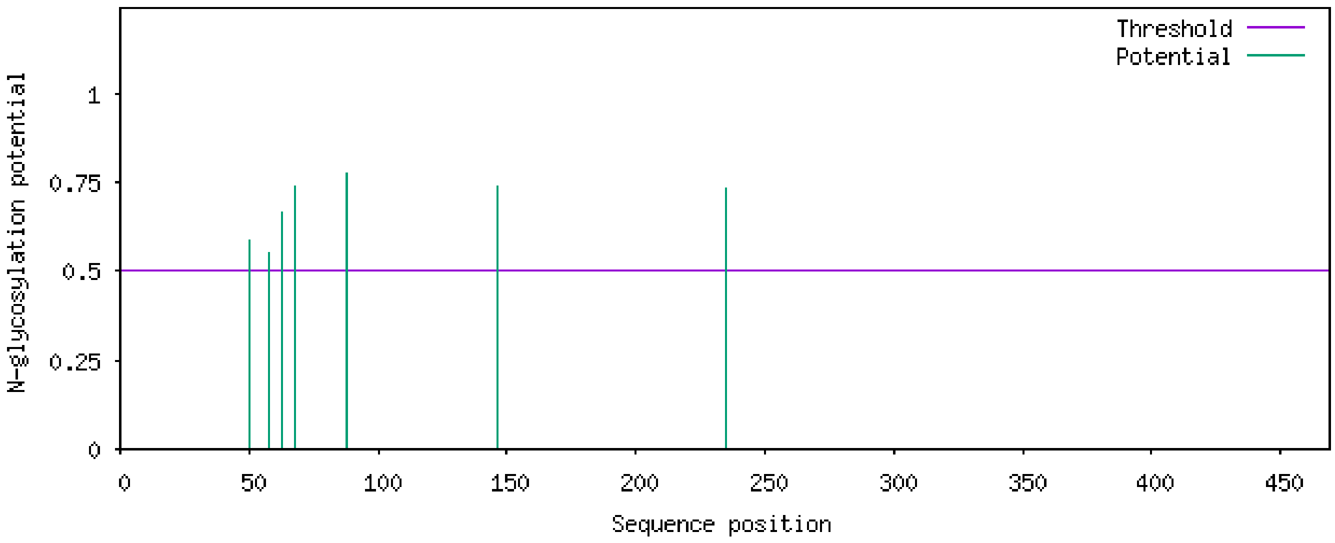

43. >XKQ11420.1 hemagglutinin [Influenza A virus/Washington/255/2024]

```
Name:  XKQ11420.1      Length:  567
MENIVLLLAIISLVKSDQICIGYHANNSTEQVDTIMEKNVTVTHAQDILEKAHNGKLCDLNGVKPLILKDCSVAGWLLGN      80
PMCDEFIRVPEWSYIVERANPANDLCYPGSLNDYEELKHLLSRINHFEKILIIPKSSWPNHETSLGVSAACPYQGAPSFF     160
RNVVWLIKKNDAYPTIKISYNNTNREDLLILWGIHHSNNAEEQTNLYKNPTTYISVGTSTLNQRLVPKIATRSQVNGQRG     240
RMDFFWTILKPDDAIHFESNGNFIAPEYAYKIVKKGDSTIMKSGVEYGHCNTKCQTPVGAINSSMPFHNIHPLTIGECPK     320
YVKSNKLVLATGLRNSPLRERRRKRGLFGAIAGFIEGGWQGMVDGWYGYHHSNEQGSGYAADKESTQKAIDGVTNKVNSI     400
IDKMNTQFEAVGREFNNLERRIENLNKKMEDGFLDVWTYNAELLVLMENERTLDFHDSNVKNLYDKVRLQLRDNAKELGN     480
GCFEFYHKCDDECMESVRNGTYDYPQYSEEARLKREEISGVKLESIGTYQILSIYSTAASSLALAIMMAGLSLWMCSNGS     560
LQCRICI                                                                              640
..........................N...........N.........................................      80
................................................................................     160
....................N...........................N...............................     240
.............................................................N..................     320
................................................................................     400
................................................................................     480
..................N..........................................................N..     560
.......                                                                              640
```

(Threshold=0.5)

SeqName	Position	Potential	Jury agreement	N-Glyc result	
XKQ11420.1	26 NNST	0.3684	(9/9)	--	
XKQ11420.1	27 NSTE	0.7794	(9/9)	+++	
XKQ11420.1	39 NVTV	0.7182	(9/9)	++	
XKQ11420.1	181 NNTN	0.6100	(7/9)	+	
XKQ11420.1	209 NPTT	0.6336	(8/9)	+	WARNING: PRO-X1.
XKQ11420.1	302 NSSM	0.5447	(6/9)	+	
XKQ11420.1	499 NGTY	0.5806	(6/9)	+	
XKQ11420.1	558 NGSL	0.6826	(9/9)	++	

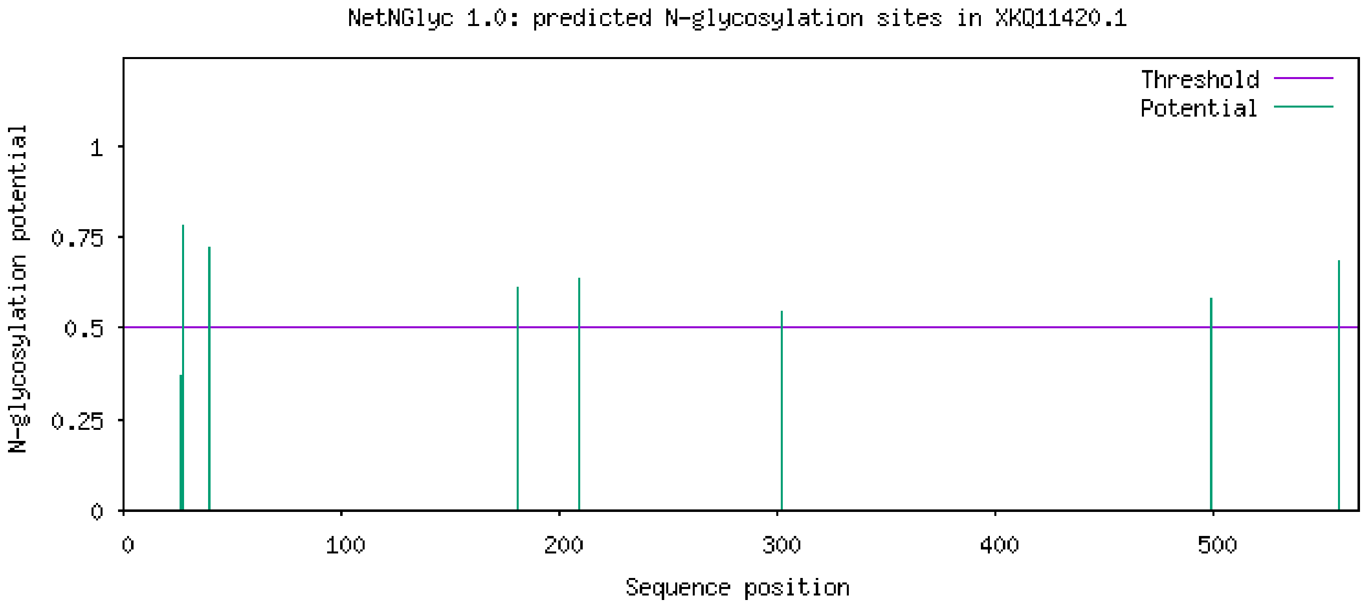

>XKQ11418.1 neuraminidase [Influenza A virus/Washington/255/2024)]

```
Name:  XKQ11418.1       Length:  469
MNPNQKIITIGSICMVIGIISLVLQIGNIISIWVSHSIQTGNQNHPETCNQSVITYENNTWVNQTYINISNTNLIAEQAV      80
DPVALAGNSSLCPISGWAIYSKDNGIRIGSKGDVFVIREPFISCSHLECRTFFLTQGALLNDKHSNGTVKDRSPYRTLMS     160
CPVGEAPSPYNSRFESVAWSASACHDGISWLTIGISGPDNGAVAVLKYNGIITDTIKSWRSNILRTQESECACINGSCFT     240
IMTDGPSNGQASYKIFRIEKGKVVKSVELNAPNYHYEECSCYPDASEVMCVCRDNWHGSNRPWVSFNQNLEYQIGYICSG     320
VFGDNPRPSDGTGSCGPVSSNGAYGVKGFSFKYGNGVWIGRTKSTSSRSGFEMIWDPNGWTETDSSFSVKQDIVAITDWS     400
GYSGSFVQHPELTGLDCMRPCFWVELIRGRPRENTIWTSGSSISFCGVNSDTVGWSWPDGAELPFTIDK     480
.................................................N.......N....N....N............      80
.......N.........................................................N..............     160
..........................................................................N.....     240
................................................................................     320
................................................................................     400
.....................................................................     480

(Threshold=0.5)
----------------------------------------------------------------------
SeqName      Position  Potential   Jury    N-Glyc
                                 agreement result
----------------------------------------------------------------------
XKQ11418.1     50 NQSV   0.6007     (8/9)    +
XKQ11418.1     58 NNTW   0.5474     (6/9)    +
XKQ11418.1     63 NQTY   0.6927     (9/9)    ++
XKQ11418.1     68 NISN   0.6721     (7/9)    +
XKQ11418.1     88 NSSL   0.7411     (9/9)    ++
XKQ11418.1    146 NGTV   0.6871     (9/9)    ++
XKQ11418.1    235 NGSC   0.6751     (9/9)    ++
----------------------------------------------------------------------
```

NetNGlyc 1.0: predicted N-glycosylation sites in XKQ11418.1

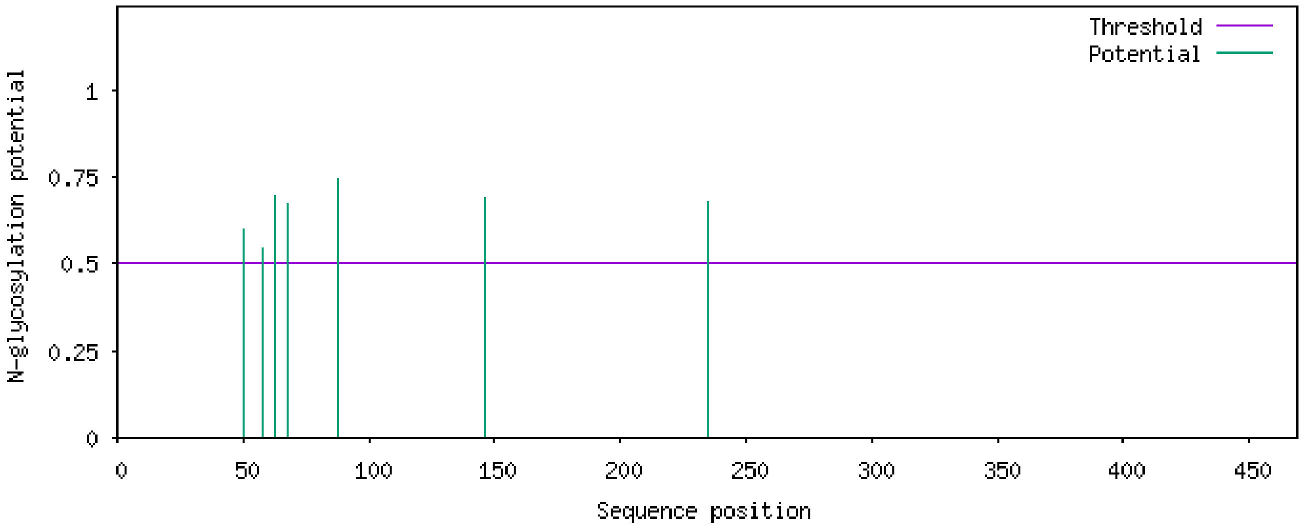

44. >XDJ26643.1 hemagglutinin [Influenza A virus/Colorado/139/2024]

```
Name:  XDJ26643.1       Length:  567
MENIVLLLAIVSLVKSDQICIGYHANNSTEQVDTIMEKNVTVTHAQDILEKTHNGKLCDLNGVKPLILKDCSVAGWLLGN      80
PMCDEFIRVPEWSYIVERANPANDLCYPGSLNDYEELKHMLSRINHFEKIQIIPKSSWPNHETSLGVSAACPYQGAPSFF     160
RNVVWLIKKNDAYPTIKISYNNTNREDLLILWGIHHSNNAEEQTNLYKNPITYISVGTSTLNQRLAPKIATRSQVNGQRG     240
RMDFFWTILKPDDAIHFESNGNFIAPEYAYKIVKKGDSTIMKSGVEYGHCNTKCQTPVGAINSSMPFHNIHPLTIGECPK     320
YVKSNKLVLATGLRNSPLREKRRKRGLFGAIAGFIEGGWQGMVDGWYGYHHSNEQGSGYAADKESTQKAIDGVTNKVNSI     400
IDKMNTQFEAVGREFNNLERRIENLNKKMEDGFLDVWTYNAELLVLMENERTLDFHDSNVKNLYDKVRLQLRDNAKELGN     480
GCFEFYHKCDNECMESVRNGTYDYPQYSEEARLKREEISGVKLESVGTYQILSIYSTAASSLALAIMMAGLSLWMCSNGS     560
LQCRICI                                                                              640
.........................N...........N..........................................      80
................................................................................     160
...................N............................................................     240
.............................................................N..................     320
................................................................................     400
................................................................................     480
..................N...........................................................N.     560
.......                                                                              640

(Threshold=0.5)
----------------------------------------------------------------------
SeqName      Position  Potential   Jury    N-Glyc
                                 agreement result
----------------------------------------------------------------------
XDJ26643.1    26 NNST   0.3686     (9/9)    --
XDJ26643.1    27 NSTE   0.7796     (9/9)    +++
XDJ26643.1    39 NVTV   0.7182     (9/9)    ++
XDJ26643.1   181 NNTN   0.6100     (7/9)    +
XDJ26643.1   302 NSSM   0.5447     (6/9)    +
XDJ26643.1   499 NGTY   0.5826     (6/9)    +
XDJ26643.1   558 NGSL   0.6827     (9/9)    ++
----------------------------------------------------------------------
```

NetNGlyc 1.0: predicted N-glycosylation sites in XDJ26643.1

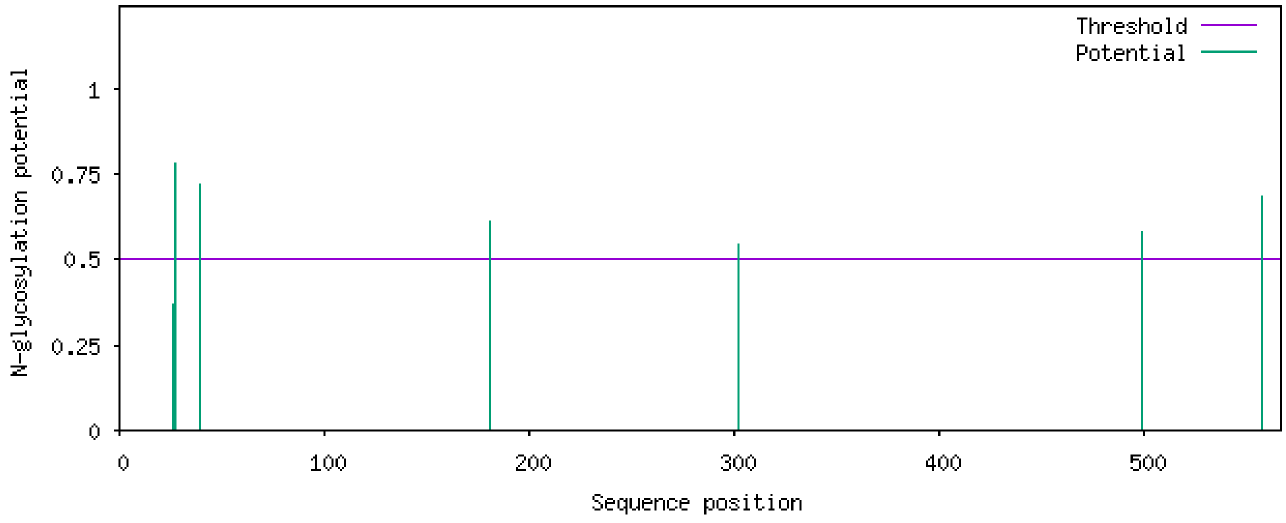

>XDJ26650.1 neuraminidase [Influenza A virus/Colorado/139/2024)

```
Name:  XDJ26650.1       Length:  469
MNPNQKITTIGSICMVIGIVSLMLQIGNIISIWVSHSIQTGNQYQPEPCNQSIITYENNTWVNQTYINISSTNFLAEQAV      80
TSVTLAGNSSLCPISGWAIYSKDNGIRIGSKGDVFVIREPFISCSHLECRTFFLTQGALLNDKHSNGTVKDRSPYRTLMS     160
CPVGEAPSPYNSRFESVAWSASACHDGISWLTIGISGPDNGAVAVLKYNGIITDTIKSWRNNILRTQESECACVNGSCFT     240
VMTDGPSNGQASYKIFKIEKGKVVKSVEMNAPNYHYEECSCYPDAGDIMCVCRDNWHGSNRPWVSFNQNLEYQIGYICSG     320
IFGDNPRPNDGTGSCSPMPSNGAYGVKGFSFKYGNGVWIGRTKSTSSRSGFEMIWDPNGWTETDSSFSVKQDIVEITDWS     400
GYSGSFVQHPELTGLDCMRPCFWVELIRGRPKENTIWTSGSSISFCGVNSDTVGWSWPDGAELPFTIDK                480
.................................................N.......N....N....N............      80
.......N.........................................................N..............     160
..........................................................................N.....     240
................................................................................     320
................................................................................     400
.....................................................................                480

(Threshold=0.5)
----------------------------------------------------------------------
SeqName      Position  Potential   Jury    N-Glyc
                                 agreement result
----------------------------------------------------------------------
XDJ26650.1     50 NQSI   0.5883     (8/9)    +
XDJ26650.1     58 NNTW   0.5251     (5/9)    +
XDJ26650.1     63 NQTY   0.6874     (9/9)    ++
XDJ26650.1     68 NISS   0.7140     (9/9)    ++
XDJ26650.1     88 NSSL   0.7724     (9/9)    +++
XDJ26650.1    146 NGTV   0.6873     (9/9)    ++
XDJ26650.1    235 NGSC   0.7321     (9/9)    ++
----------------------------------------------------------------------
```

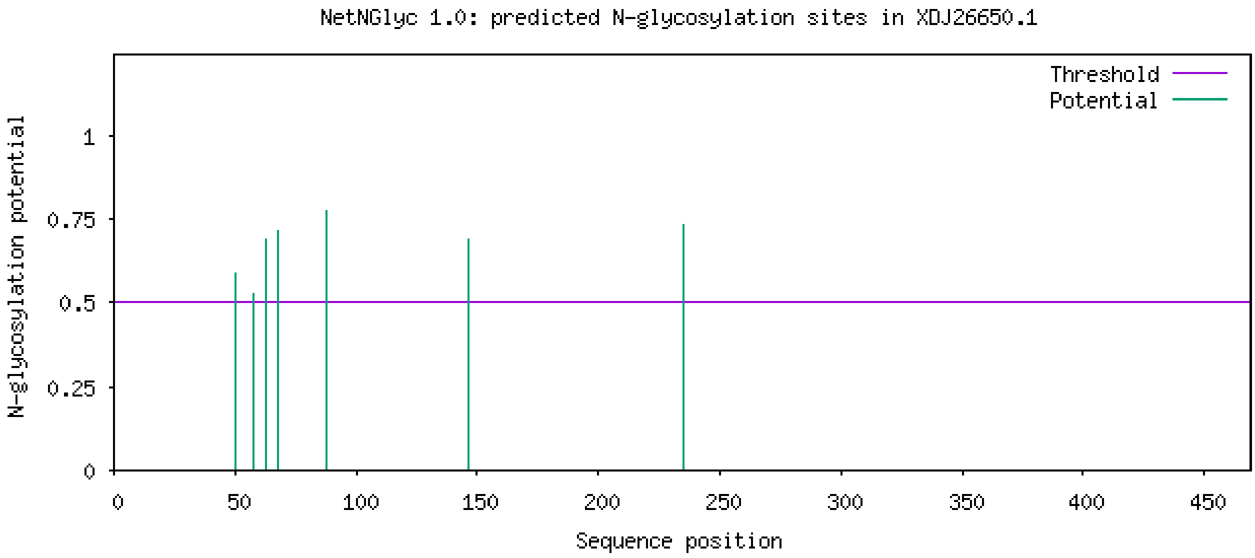

45. >WPM96680.1 hemagglutinin [Influenza A virus/Numida-meleagris/Maracaja/1843-N3/2023]

```
Name:  WPM96680.1      Length:  567
MENIVLLLAIVSLVKSDQICIGYHANNSTEQVDTIMEKNVTVTHAQDILEKTHNGKLCDLNGVKPLILQDCSVAGWLLGN      80
PMCDEFIRVPEWSYIVERANPANDLCYPGSLNDYEELKHMLSRINHFEKIQIIPKSSWPNHETSLGVSAACPYQGAPSFF     160
RNVVWLIKKNDAYPTIKISYNNTNREDLLILWGIHHSNNAEEQTNLYKNPTTYISVGTSTLNQRMAPKIATRSQVNEQRG     240
RMDFFWTLLKPDDAIHFECNGNFIAPEYAYKIVKKGYSTIMKSGEEYGHCNTKCQTPLGAINSSMPFHNIHPLTIEECPK     320
YVKTNKLVLATGLRNPLREKRRKRGLFGAIAGFIEGGWQGMVDGWYGYHHSNEQGSGYAADKESTQKAIDGVTNKVNSI     400
IDKMNTQFEAVGREFNNLERRIENLNKKMEDGFLDVWTYNAELLVLMENERTLDFHDSNVKNLYDKVRLQLRDNAKELGN     480
GCFEFYHKCDNDCMESVRNGTYDYPQYSEEARLKREEISGVKLESVGTYQILSIYSTAASSLALAIMMAGLSLWMCSNGS     560
LQCRICI                                                                             640
..........................N...........N.........................................      80
................................................................................     160
....................N...........................N...............................     240
.............................................................N..................     320
................................................................................     400
................................................................................     480
..................N..........................................................N..     560
.......                                                                             640

(Threshold=0.5)
----------------------------------------------------------------------
SeqName      Position  Potential   Jury     N-Glyc
                                 agreement  result
----------------------------------------------------------------------
WPM96680.1     26 NNST   0.3686     (9/9)    --
WPM96680.1     27 NSTE   0.7796     (9/9)    +++
WPM96680.1     39 NVTV   0.7183     (9/9)    ++
WPM96680.1    181 NNTN   0.6101     (7/9)    +
WPM96680.1    209 NPTT   0.6337     (8/9)    +     WARNING: PRO-X1.
WPM96680.1    302 NSSM   0.6028     (7/9)    +
WPM96680.1    499 NGTY   0.5844     (6/9)    +
WPM96680.1    558 NGSL   0.6829     (9/9)    ++
----------------------------------------------------------------------
```

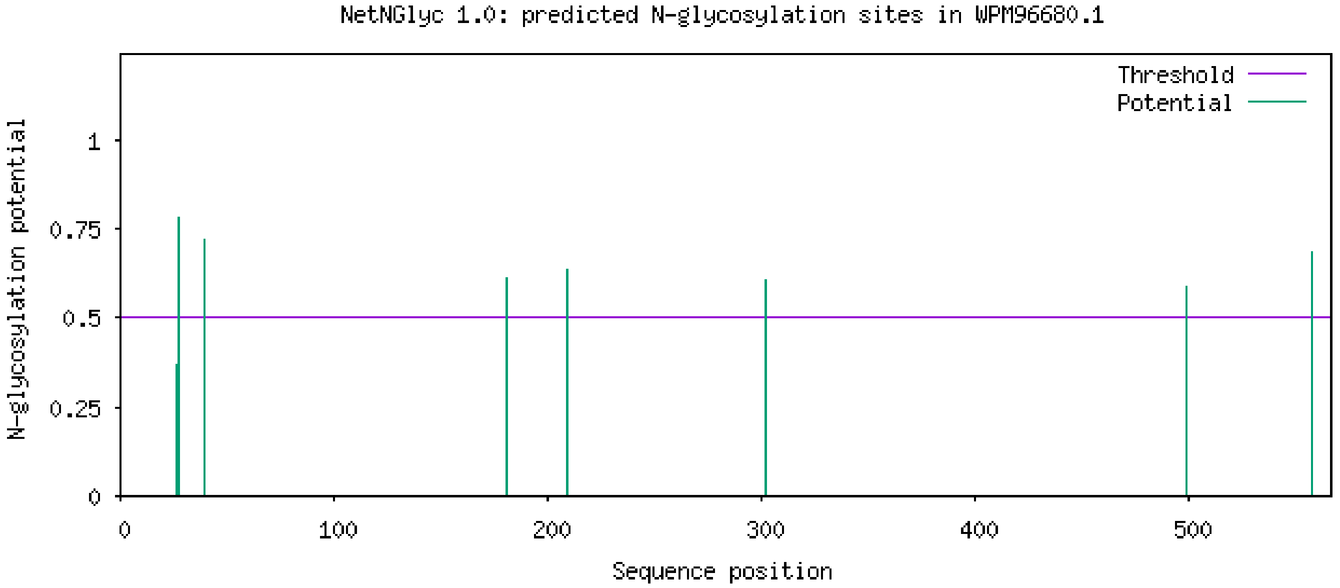

>WPM96682.1 neuraminidase [Influenza A virus/Numida meleagris/Maracaja/1843-N3/2023]

```
Name:  WPM96682.1       Length:  469
MNPNQKITTIGSICMVIGVVSLMLQIGNIISIWVSHSIQTGNQYQPEPCNQSIITYENNTWVNQTYVNISNTNFLAEQAV      80
TSVTLAGNSSLCPISGWAIYSKDNGIRIGSKGDVFVIREPFISCSHLECRTFFLTQGALLNDKHSNGTVKDRSPYRTLMS     160
CPVGEAPSPYNSRFESVAWSASACHDGISWLTIGISGPDNGAVAVLKYNGIITDTIRSWRNNILRTQESECACVNGSCFT     240
VMTDGPSNGQASYKIFKIEKGKVVKSVEMNAPNYHYEECSCYPDAGDIMCVCRDNWHGSNRPWVSFNQNLEYQIGYICSG     320
VFGDNPRPNDGTGSCSPMPSNGAYGVKGFSFKYGNGVWIGRTKSTSSRSGFEMIWDPNGWTETDSSFSVKQDIVEITDWS     400
GYSGSFVQHPELTGLDCIRPCFWVELIRGRPKENTIWTSGSSISFCGVNSDTVGWSWPDGAELPFTIDK                480
.................................................N.......N....N....N............      80
.......N.........................................................N..............     160
..........................................................................N.....     240
................................................................................     320
................................................................................     400
.....................................................................                480

(Threshold=0.5)
----------------------------------------------------------------------
SeqName      Position  Potential   Jury    N-Glyc
                                 agreement result
----------------------------------------------------------------------
WPM96682.1    50 NQSI   0.5884     (8/9)   +
WPM96682.1    58 NNTW   0.5496     (6/9)   +
WPM96682.1    63 NQTY   0.6636     (9/9)   ++
WPM96682.1    68 NISN   0.7377     (9/9)   ++
WPM96682.1    88 NSSL   0.7724     (9/9)   +++
WPM96682.1   146 NGTV   0.6876     (9/9)   ++
WPM96682.1   235 NGSC   0.7321     (9/9)   ++
----------------------------------------------------------------------
```

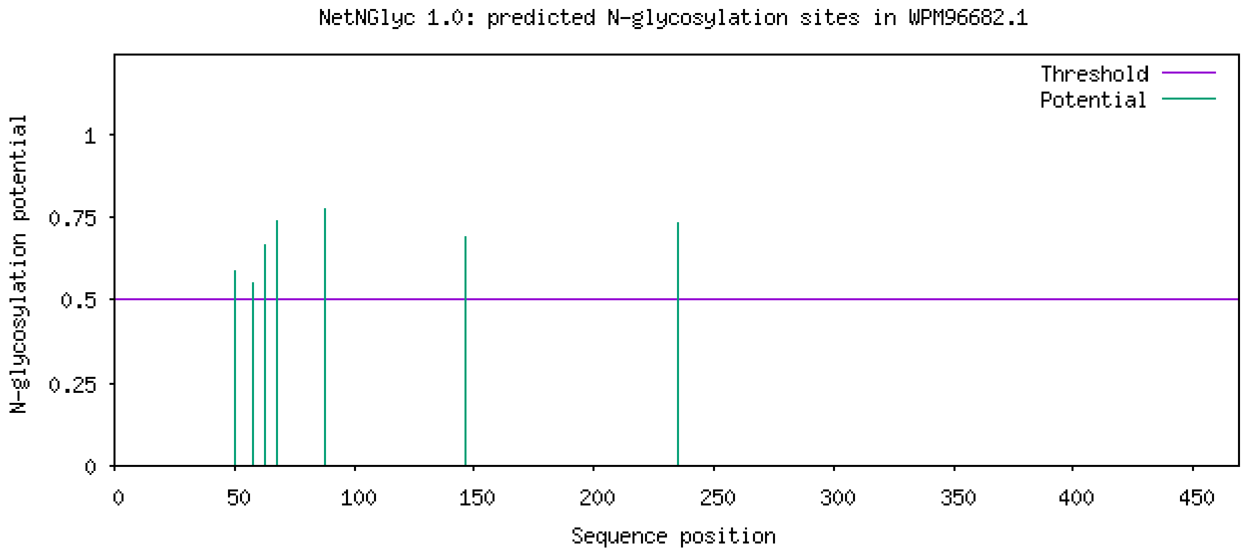

46. >XAN64344.1 hemagglutinin [Influenza A virus/Procellaria-aequinoctialis/UbatubaBR/2271-N/2023]

```
Name:  XAN64344.1       Length:  567
MENIVLLLAIVSLVKSDQICIGYHANNSTEQVDTIMEKNVTVTHAQDILEKTHNGKLCDLNGVKPLILKDCSVAGWLLGN      80
PMCDEFIRVPEWSYIVERANPANGLCYPGSLNDYEELKHMLSRINHFEKIQIIPKSSWPNHETSLGVSAACPYQGAPSFF     160
RNVVWLIKKNDAYPTIKISYNNTNREDLLILWGIHHSNNAEEQTNLYKNPTTYISVGTSTLNQRLAPKIATRSQVNGQRG     240
RIDFFWTILKPDDAIHFESNGNFIAPEYAYKIVKKGDSTIMKSGVEYGHCNTKCQTPVGAINSSMPFHNIHPLTIGECPK     320
YVKSNKLVLATGLRNSPLREKRKKRGLFGAIAGFIEGGWQGMVDGWYGYHHSNEQGSGYAADKESTQKAIDGVTNKVNSI     400
IDKMNTQFEAVGREFNNLERRIENLNKKMEDGFLDVWTYNAELLVLMENERTLDFHDSNVKNLYDKVRLQLRDNAKELGN     480
GCFEFYHKCDNECMESVRNGTYYYPQYSEEARLKREEISGVKLESVGTYQILSIYSTAASSLALAIMMAGLSLWMCSNGS     560
LQCRICI                                                                              640
..........................N...........N.........................................      80
................................................................................     160
....................N...........................N...............................     240
.............................................................N..................     320
................................................................................     400
................................................................................     480
..................N..........................................................N..     560
.......                                                                              640

(Threshold=0.5)
----------------------------------------------------------------------
SeqName      Position  Potential   Jury     N-Glyc
                                agreement  result
----------------------------------------------------------------------
XAN64344.1     26 NNST   0.3686     (9/9)    --
XAN64344.1     27 NSTE   0.7796     (9/9)    +++
XAN64344.1     39 NVTV   0.7182     (9/9)    ++
XAN64344.1    181 NNTN   0.6100     (7/9)    +
XAN64344.1    209 NPTT   0.6334     (8/9)    +     WARNING: PRO-X1.
XAN64344.1    302 NSSM   0.5448     (6/9)    +
XAN64344.1    499 NGTY   0.5821     (5/9)    +
XAN64344.1    558 NGSL   0.6826     (9/9)    ++
----------------------------------------------------------------------
```

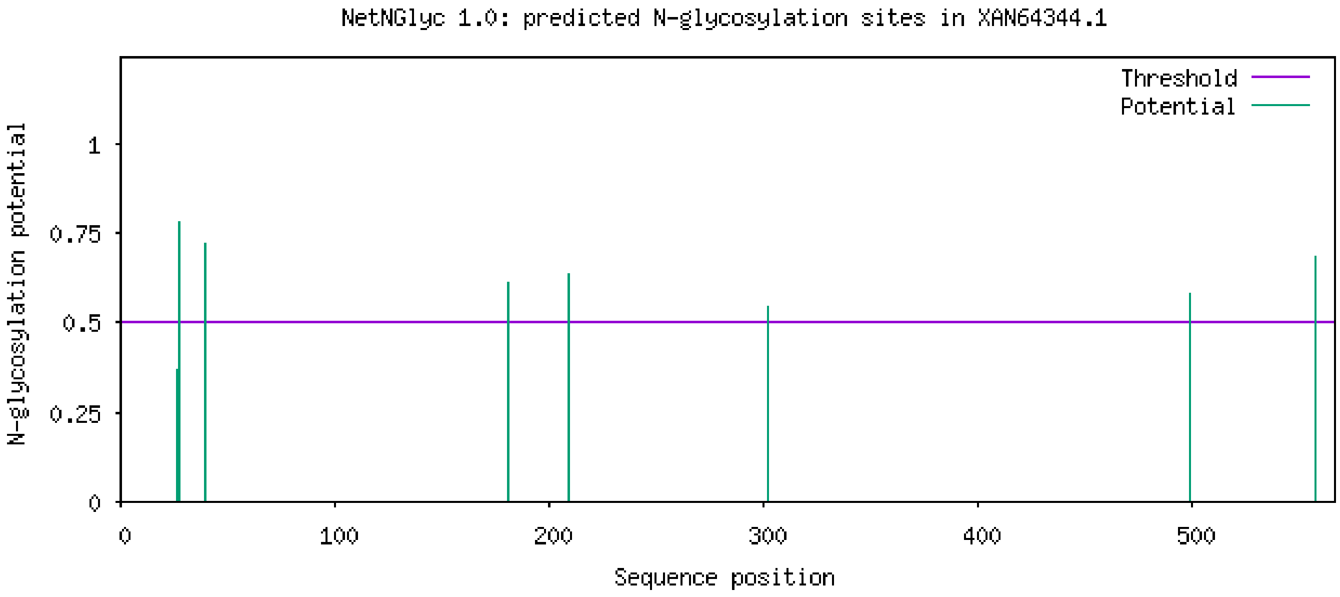

>XAN64345.1 neuraminidase [Influenza A virus/Procellaria aequinoctialis/UbatubaBR/2271-N/2023/]

```
Name:  XAN64345.1      Length:  469
MNPNQKITTIGSICMVIGIVSLMLQIGNIISIWVSHSIQTGNQYQPEPCNQSIITYENNTWVNQTYVNISNTNFLAEQAV      80
TSVTLAGNSSLCPISGWAIYSKDNGIRIGSKGDVFVIREPFISCSHLECRTFFLTQGALLNDKHSNGTVKDRSPYRTLMS     160
CPVGEAPSPYNSRFESVAWSASACHDGISWLTIGISGPDNGAVAVLKYNGIITDTIKSWRNNILRTQESECACVNGSCFT     240
VMTDGPSNGQASYKIFKIEKGKVVKSVEMNAPNYHYEECSCYPDAGDIMCVCRDNWHGSNRPWVSFNQNLEYQIGYICSG     320
VFGDNPRPNDGTGSCSPMPSNGAYGVKGFSFKYGNGVWIGRTKSTSSRSGFEMIWDPNGWTETDSSFSVKQDIVEITDWS     400
GYSGSFVQHPELTGLDCMRPCFWVELIRGRPKENTIWTSGSSISFCGVNSDTVGWSWPDGAELPFTIDK                480
.................................................N.......N....N....N............      80
.......N.........................................................N..............     160
..........................................................................N.....     240
................................................................................     320
................................................................................     400
.....................................................................                480

(Threshold=0.5)
----------------------------------------------------------------------
SeqName      Position  Potential   Jury    N-Glyc
                                 agreement result
----------------------------------------------------------------------
XAN64345.1     50 NQSI   0.5884     (8/9)   +
XAN64345.1     58 NNTW   0.5496     (6/9)   +
XAN64345.1     63 NQTY   0.6636     (9/9)   ++
XAN64345.1     68 NISN   0.7378     (9/9)   ++
XAN64345.1     88 NSSL   0.7724     (9/9)   +++
XAN64345.1    146 NGTV   0.6876     (9/9)   ++
XAN64345.1    235 NGSC   0.7321     (9/9)   ++
----------------------------------------------------------------------
```

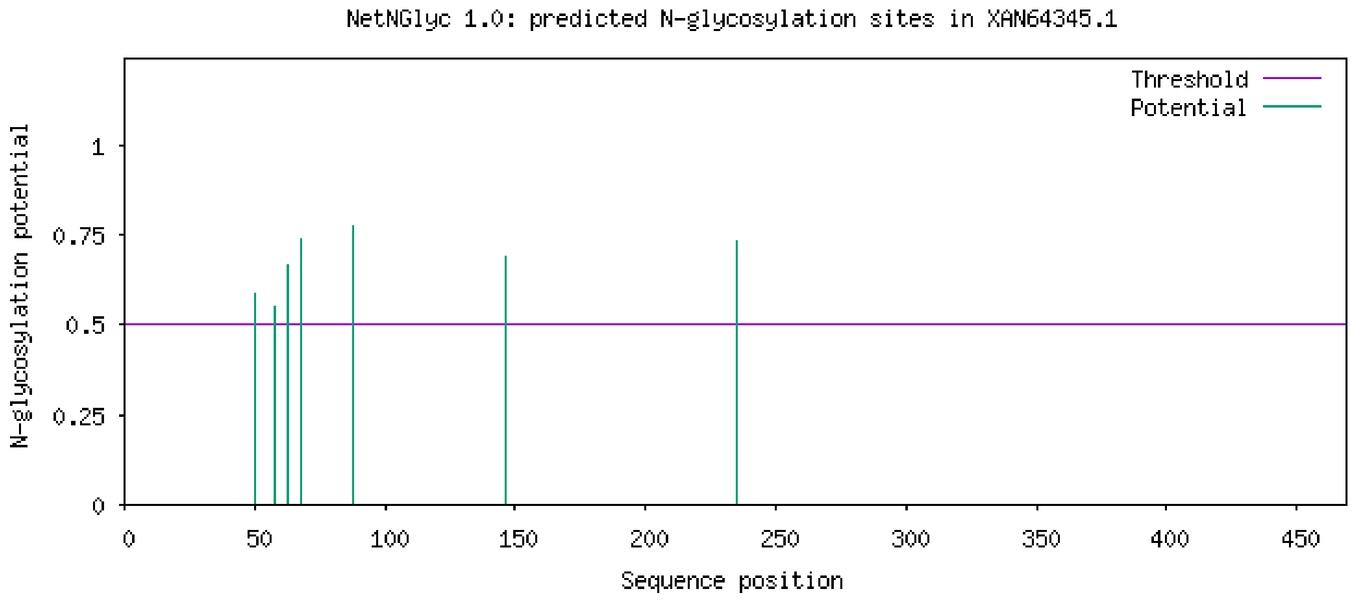

47. >XAO43963.1 hemagglutinin [Influenza A virus/Sterna-hirundo/MacaeBR/0177-N/2024]

```
Name:  XAO43963.1       Length:  567
MENIVLLLAIVSLVKSDQICIGYHANNSTEQVDTIMEKNVTVTHAQDILEKTHNGKLCDLNGVKPLILKDCSVAGWLLGN      80
PMCDEFIRVPEWSYIVERANPANGLCYPGSLNDYEELKHMLSRINHFEKIQIIPKSSWPNHETSLGVSAACPYQGAPSFF     160
RNVVWLIKKNDAYPTIKISYNNTNREDLLILWGIHHSNNAEEQTNLYKNPTTYISVGTSTLNQRLAPKIATRSQVNGQRG     240
RIDFFWTILKPDDAIHFESNGNFIAPEYAYKIVKKGDSTIMKSGVEYGHCNTKCQTPVGAINSNMPFHNIHPLTIGECPK     320
YVKSNKLVLATGLRNSPLREKRKKRGLFGAIAGFIEGGWQGMVDGWYGYHHSNEQGSGYAADKESTQKAIDGVTNKVNSI     400
IDKMNIQFEAVGREFNNLERRIENLNKKMEDGFLDVWTYNAELLVLMENERTLDFHDSNVKNLYDKVRLQLRDNAKELGN     480
GCFEFYHKCDNECMESVRNGTYYYPQYSEEARLKREEISGVKLESVGTYQILSIYSTAASSLALAIMMAGLSLWMCSNGS     560
LQCRICI                                                                              640
..........................N...........N.........................................      80
................................................................................     160
....................N...........................N...............................     240
................................................................................     320
................................................................................     400
................................................................................     480
..................N..........................................................N..     560
.......                                                                              640
```

(Threshold=0.5)

SeqName	Position	Potential	Jury agreement	N-Glyc result	
XAO43963.1	26 NNST	0.3687	(9/9)	--	
XAO43963.1	27 NSTE	0.7796	(9/9)	+++	
XAO43963.1	39 NVTV	0.7183	(9/9)	++	
XAO43963.1	181 NNTN	0.6101	(7/9)	+	
XAO43963.1	209 NPTT	0.6334	(8/9)	+	WARNING: PRO-X1.
XAO43963.1	499 NGTY	0.5821	(5/9)	+	
XAO43963.1	558 NGSL	0.6829	(9/9)	++	

NetNGlyc 1.0: predicted N-glycosylation sites in XAO43963.1

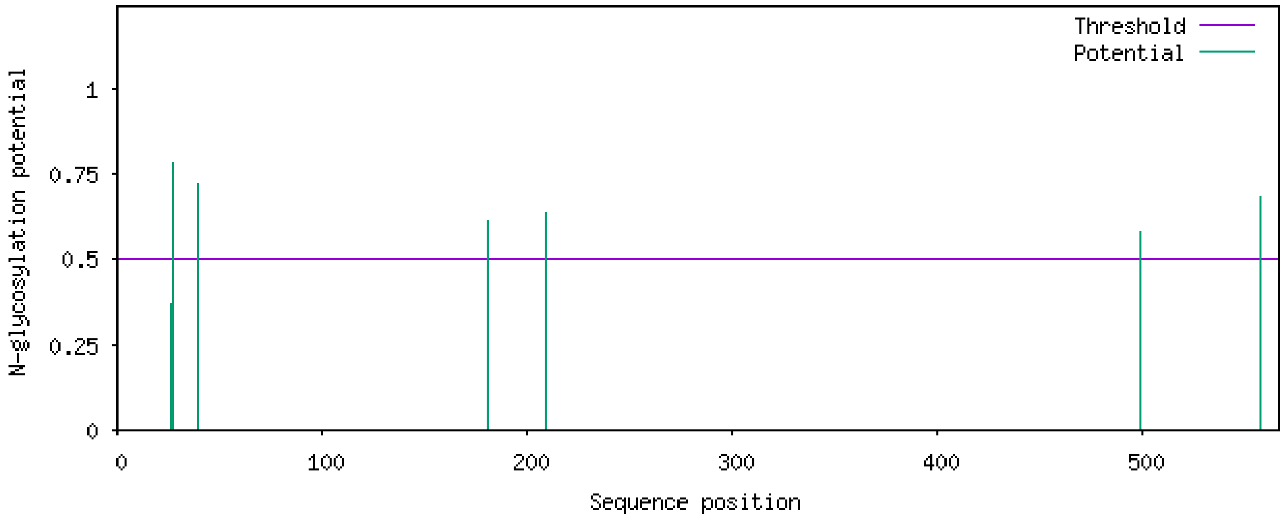

>XAO43965.1 neuraminidase [Influenza A virus/Sterna hirundo/MacaeBR/0177-N/2024]

```
Name:  XAO43965.1       Length:  469
MNPNQKITTIGSICMVIGIVSLMLQIGNIISIWVSHSIQTGNQYQPEPCNQSIITYENNTWVNQTYVNISNTNFLAEQAV      80
TSVTLAGNSSLCPISGWAIYSKDNGIRIGSKGDVFVIREPFISCSHLECRTFFLTQGALLNDKHSNGTVKDRSPYRTLMS     160
CPVGEAPSPYNSRFESVAWSASACHDGISWLTIGISGPDNGAVAVLKYNGIITDTIKSWRNNILRTQESECACVNGSCFT     240
VMTDGPSNGQASYKIFKIEKGKVVKSVEMNAPNYHYEECSCYPDAGDIMCVCRDNWHGSNRPWVSFNQNLEYQIGYICSG     320
VFGDNPRPNDGTGSCSPMPSNGAYGVKGFSFKYGNGVWIGRTKSISSRSGFEMIWDPNGWTETDSSFSVKQDIVEITDWS     400
GYSGSFVQHPELTGLDCMRPCFWVELIRGRPKENTIWTSGSSISFCGVNSDTVGWSWPDGAELPFTVDK                480
.................................................N.......N....N....N............      80
.......N.........................................................N..............     160
..........................................................................N.....     240
................................................................................     320
................................................................................     400
.....................................................................                480

(Threshold=0.5)
```

SeqName	Position		Potential	Jury agreement	N-Glyc result
XAO43965.1	50	NQSI	0.5883	(8/9)	+
XAO43965.1	58	NNTW	0.5496	(6/9)	+
XAO43965.1	63	NQTY	0.6636	(9/9)	++
XAO43965.1	68	NISN	0.7377	(9/9)	++
XAO43965.1	88	NSSL	0.7724	(9/9)	+++
XAO43965.1	146	NGTV	0.6876	(9/9)	++
XAO43965.1	235	NGSC	0.7321	(9/9)	++

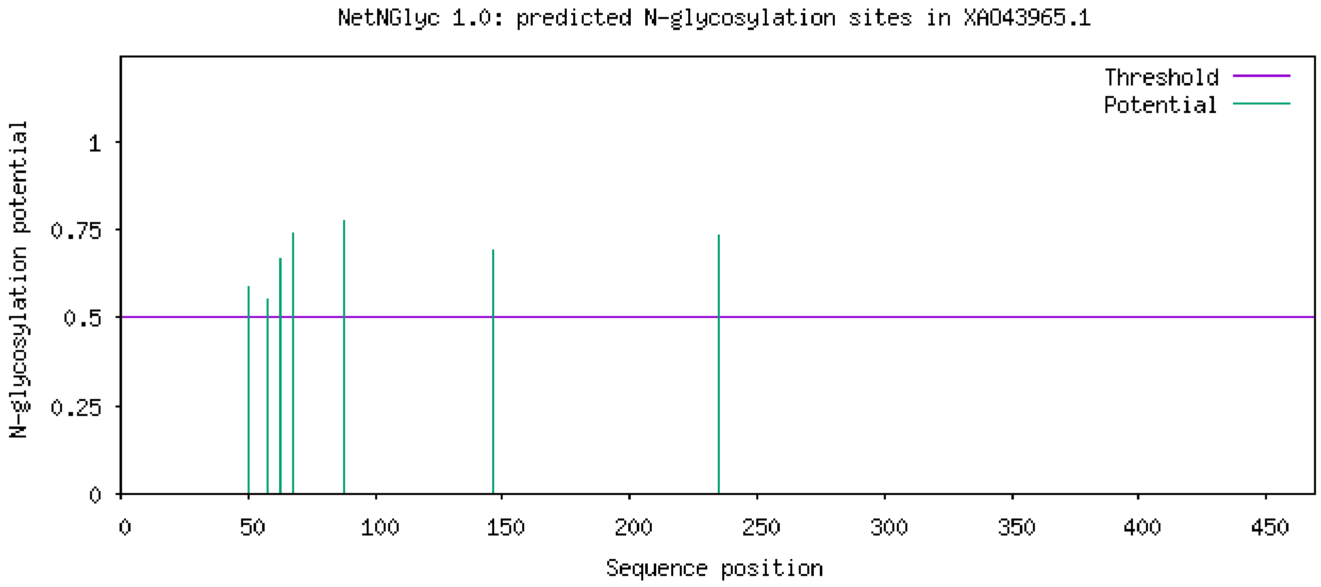

48. >XLV27167.1 hemagglutinin [Influenza A virus/Montana/51_LUL_HA/2024]

```
Name:  XLV27167.1       Length:  567
MENIVLLLAIVSLVKSDQICIGYHANNSTEQVDTIMEKNVTVTHAQDILEKTHNGKLCDLNGVKPLILKDCSVAGWLLGN      80
PMCDEFIRVPEWSYIVERANPANDLCYPGSLNDYEELKHMLSRINHFEKIQIIPKSSWPNHETSLGVSAACPYQGAPSFF     160
RNVVWLIKKNDAYPTIKISYNNTNREDLLILWGIHHSNNAEEQTNLYKNPITYISVGTSTLNQRLAPKIATRSQVNGQRG     240
RMDFFWTILKPDDAIHFESNGNFIAPEYAYKIVKKGDSTIMKSGVEYGHCNTKCQTPVGAINSSMPFHNIHPLTIGECPK     320
YVKSNKLVLATGLRNSPLREKRRKRGLFGAIAGFIEGGWQGMVDGWYGYHHSNEQGSGYAADKESTQKAIDGVTNKVNSI     400
IDKMNTQFEAVGREFNNLERRIENLNKKMEDGFLDVWTYNAELLVLMENERTLDFHDSNVKNLYDKVRLQLRDNAKELGN     480
GCFEFYHKCDNECMESVRNGTYDYPQYSEEARLKREEISGVKLESVGTYQILSIYSTAASSLALAIMMAGLSLWMCSNGS     560
LQCRICI                                                                              640
..........................N...........N.........................................      80
................................................................................     160
....................N...........................................................     240
.............................................................N..................     320
................................................................................     400
................................................................................     480
..................N..........................................................N..     560
.......                                                                              640
```

(Threshold=0.5)

SeqName	Position	Potential	Jury agreement	N-Glyc result
XLV27167.1	26 NNST	0.3686	(9/9)	--
XLV27167.1	27 NSTE	0.7796	(9/9)	+++
XLV27167.1	39 NVTV	0.7182	(9/9)	++
XLV27167.1	181 NNTN	0.6100	(7/9)	+
XLV27167.1	302 NSSM	0.5447	(6/9)	+
XLV27167.1	499 NGTY	0.5826	(6/9)	+
XLV27167.1	558 NGSL	0.6827	(9/9)	++

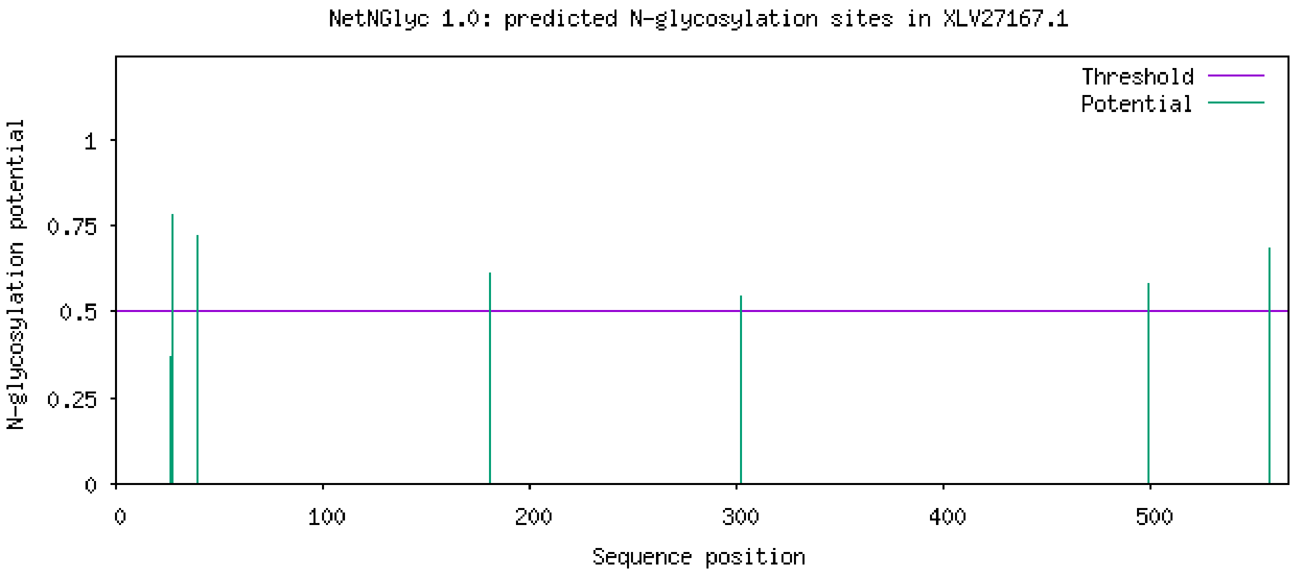

>XLV27170.1 neuraminidase [Influenza A virus/Montana/51_LUL_NA/2024]

```
Name:  XLV27170.1       Length:  469
MNPNQKITTIGSICMVIGIVSLMLQIGNIISIWVSHSIQTGNQYQPEPCNQSIITYENNTWVNQTYINISNTNFLAEQAV      80
TSVTLAGNSSLCPISGWAIYSKDNGIRIGSKGDVFVIREPFISCSHLECRTFFLTQGALLNDKHSNGTVKDRSPYRTLMS     160
CPVGEAPSPYNSRFESVAWSASACHDGISWLTIGISGPDNGAVAVLKYNGIITDTIKSWRNNILRTQESECACVNGSCFT     240
VMTDGPSNGQASYKIFKIEKGKVVKSVEMNAPNYHYEECSCYPDAGDIMCVCRDNWHGSNRPWVSFNQNLEYQIGYICSG     320
IFGDNPRPNDGTGSCSPMPSNGAYGVKGFSFKYGNGVWIGRTKSTSSRSGFEMIWDPNGWTETDSSFSVKQDIVEITDWS     400
GYSGSFVQHPELTGLDCMRPCFWVELIRGRPKENTIWTSGSSISFCGVNSDTVGWSWPDGAELPFTIDK                480
.................................................N.......N....N....N............      80
.......N.........................................................N..............     160
..........................................................................N.....     240
................................................................................     320
................................................................................     400
.....................................................................                480
```

```
(Threshold=0.5)
----------------------------------------------------------------------
SeqName      Position  Potential   Jury    N-Glyc
                                agreement  result
----------------------------------------------------------------------
```

SeqName	Position	Potential	Jury agreement	N-Glyc result
XLV27170.1	50 NQSI	0.5881	(8/9)	+
XLV27170.1	58 NNTW	0.5252	(5/9)	+
XLV27170.1	63 NQTY	0.6762	(9/9)	++
XLV27170.1	68 NISN	0.6866	(7/9)	+
XLV27170.1	88 NSSL	0.7724	(9/9)	+++
XLV27170.1	146 NGTV	0.6874	(9/9)	++
XLV27170.1	235 NGSC	0.7321	(9/9)	++

NetNGlyc 1.0: predicted N-glycosylation sites in XLV27170.1

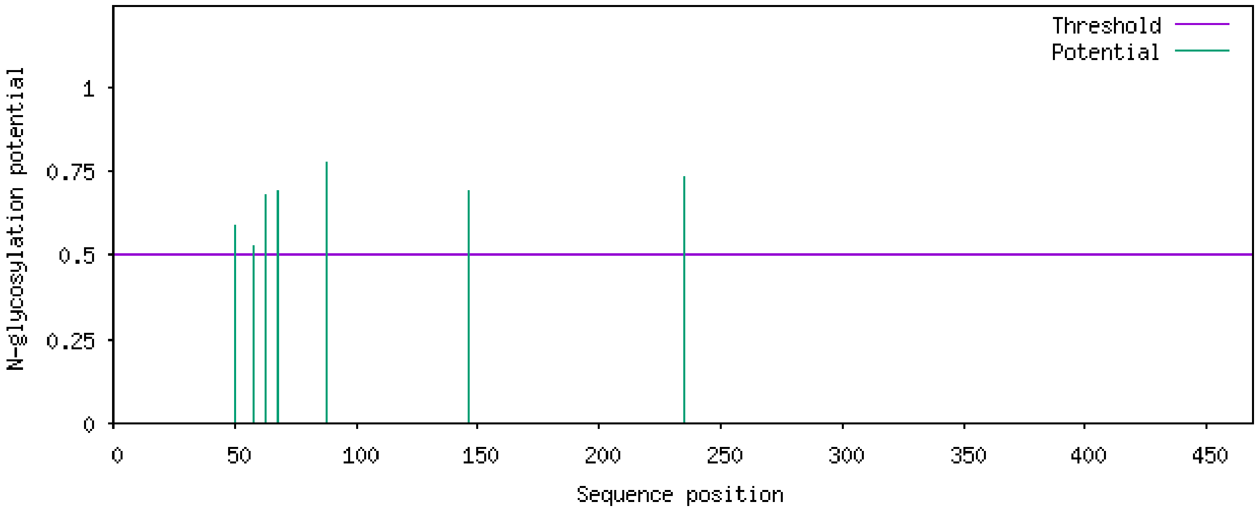

49. >XLL10621.1 hemagglutinin [Influenza A virus/turkey/Germany-NW/2024AI04873/2024]

```
Name:  XLL10621.1       Length:  567
MENIVLLLAIVSLVKSDQICIGYHANNSTEQVDTIMEKNVTVTHAQDILEKTHNGKLCDLNGVKPLILKDCSVAGWLLGN      80
PMCDEFIRVPEWSYIVERDSPANDLCYPGSLNDYEELKHLLSRINHFEKILIIPKSSWPNHETSLGVSAACSYQGAPSFF     160
RNVVWLIKKNDAYPTIKISYNNTNREDLLILWGIHHSNNAEEQTNLYKNPTTYISVGTSTLNQRLVPKIATRSQVNGQRG     240
RMDFFWTILKPDDAIHFESNGNFIAPEYAYKIVKKGDSTIMKSGVEYGHCNTKCQTPVGAINSSMPFHNIHPLTIGECPK     320
YVKSNKLVLATGLRNSPLREKRRKRGLFGAIAGFIEGGWQGMVDGWYGYHHSNEQGSGYAADKESTQKAIDGVTNKVNSI     400
IDKMNTQFEAVGREFNNLERRIENLNKKMEDGFLDVWTYNAELLVLMENERTLDFHDSNVKNLYDKVRLQLKDNAKELGN     480
GCFEFYHKCDNECMESVRNGTYDYPQYSEEARLKREEINGVKLESIGTYQILSIYSTAASSLALAIMMAGLSLWMCSNGS     560
LQCRICI                                                                             640
.........................N............N.........................................      80
................................................................................     160
....................N...........................N...............................     240
.............................................................N..................     320
................................................................................     400
................................................................................     480
..................N..........................................................N..     560
.......                                                                             640

(Threshold=0.5)
----------------------------------------------------------------------
SeqName      Position  Potential   Jury    N-Glyc
                                agreement  result
----------------------------------------------------------------------
XLL10621.1     26 NNST   0.3686     (9/9)   --
XLL10621.1     27 NSTE   0.7794     (9/9)   +++
XLL10621.1     39 NVTV   0.7181     (9/9)   ++
XLL10621.1    181 NNTN   0.6100     (7/9)   +
XLL10621.1    209 NPTT   0.6336     (8/9)   +     WARNING: PRO-X1.
XLL10621.1    302 NSSM   0.5447     (6/9)   +
XLL10621.1    499 NGTY   0.5824     (6/9)   +
XLL10621.1    558 NGSL   0.6828     (9/9)   ++
----------------------------------------------------------------------
```

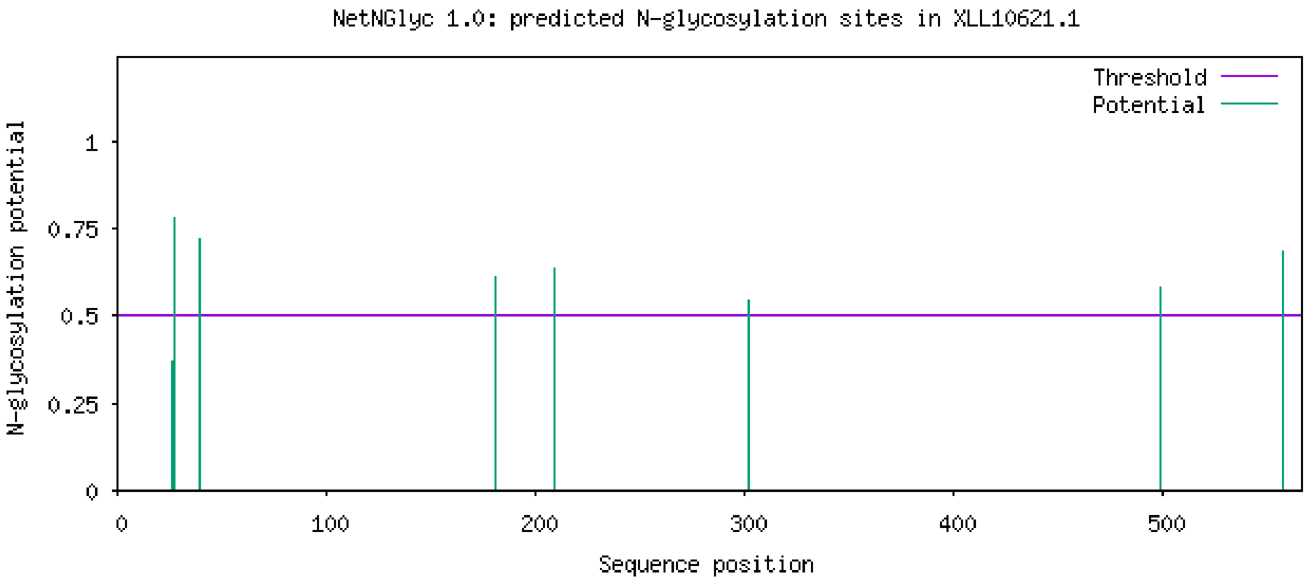

>XLL10623.1 neuraminidase [Influenza A virus/turkey/Germany-NW/2024AI04873/2024]

```
Name:  XLL10623.1      Length:  469
MNPNQRIITTGSICMTIGIISLMLQIGNIISIWVSHSIQTGNQYQPEPCNQSIITYENNTWVNQTYVNISNTNCLAEQAV      80
TSVTLAGNSPLCPISGWAIYSKDNGVRIGSKGDVFVIREPFISCSHLECRTFFLTQGALLNDKHSNGTVKDRSPYRTLMS     160
CPVGEAPSPYNSRFESVAWSASACHDGISWLTIGISGPDNGAVAVLKYNGIITDTIKSWRNNILRTQESECACVNGSCFT     240
VMTDGPSNGQASYKIFKIEKGKVVKSVELNAPNYHYEECSCYPDAGEIMCVCRDNWHGSNRPWVSFNKNLEYQIGYICSG     320
VFGDNPRPNDGTGSCSPMSSNGAYGVKGFSFKYGNGVWIGRTKSTSSRSGFEMIWDPNGWTETDSSFSVKQDIVAITDWS     400
GYSGTFVQHPELTGLDCMRPCFWVELIRGRPKENTIWTSGSSISFCGVNSDTVGWSWPDGAELPYTIDK                480
.................................................N.......N....N....N............      80
.................................................................N..............     160
..........................................................................N.....     240
................................................................................     320
................................................................................     400
.....................................................................                480

(Threshold=0.5)
----------------------------------------------------------------------
SeqName      Position  Potential   Jury    N-Glyc
                                agreement  result
----------------------------------------------------------------------
XLL10623.1     50 NQSI   0.5883     (8/9)   +
XLL10623.1     58 NNTW   0.5496     (6/9)   +
XLL10623.1     63 NQTY   0.6634     (9/9)   ++
XLL10623.1     68 NISN   0.6992     (8/9)   +
XLL10623.1    146 NGTV   0.6873     (9/9)   ++
XLL10623.1    235 NGSC   0.7320     (9/9)   ++
----------------------------------------------------------------------
```

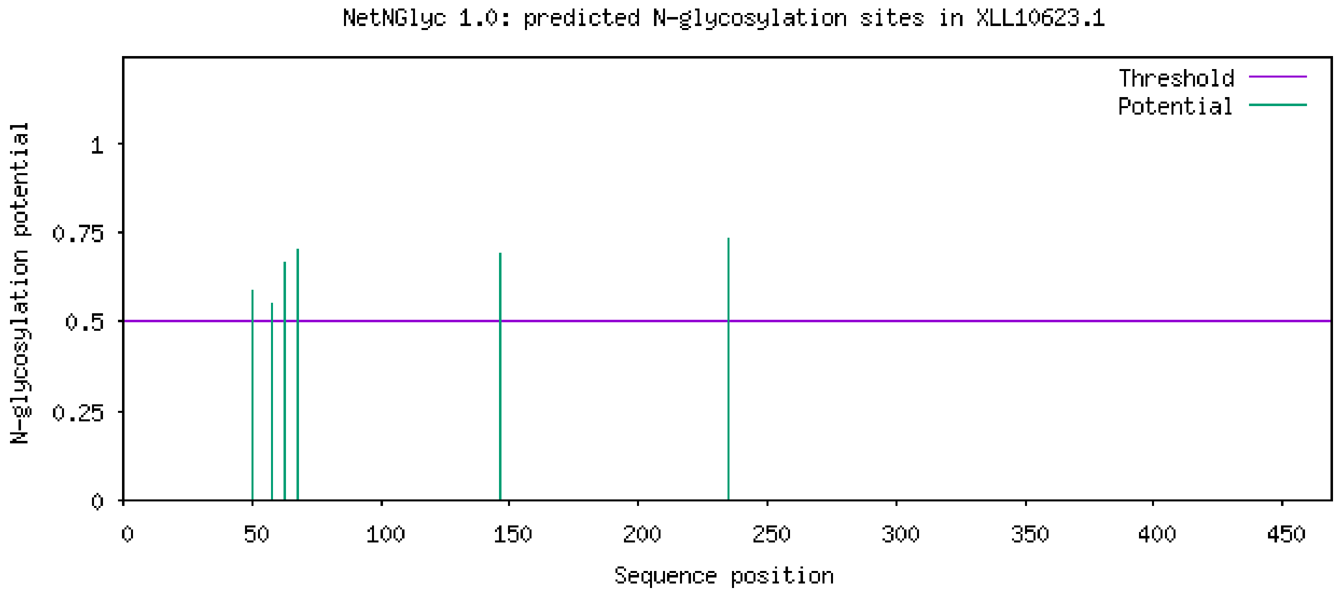

50. >WRI00961.1 hemagglutinin [Influenza A virus/duck/Bangladesh/58401/2023]

```
Name:  WRI00961.1       Length:  567
MENIVLLLATVSLVKSDQICIGYHANNSTEQVDTIMEKNVTVTHAQDILEKTHNGKLCDLNGVKPLILKDCSVAGWLLGN      80
PMCDEFIRVPEWSYIVERDNPANDLCYPGSLNDYEELKHLLSRINHFEKILIIPKSSWPNHETSLGVSAACPYQGAPSFF     160
RNVVWLIKKDDAYPTIKISYNNTNREDLLILWGIHHSNNAEEQTNLYKNPTTYISVGTSTLNQRLVPKIATRSQVNGQRG     240
RMDFFWTILKPDDAIHFESNGNFIAPEYAYKIVKKGDSTIMKSGVEYGHCNTKCQTPVGAINSSMPFHNIHPLTIGECPK     320
YVKSNKLVLATGLRNSPLREKRRKRGLFGAIAGFIEGGWQGMVDGWYGYHHSNEQGSGYAADKESTQKAIDGVTNKVNSI     400
IDKMNTQFEAVGREFNNLERRIENLNKKMEDGFLDVWTYNAELLVLMENERTLDFHDSNVKNLYDKVRLQLRDNAKELGN     480
GCFEFYHKCDNECMESVRNGTYYYPQYSEEARLKREEISGVKLESIGTYQILSIYSTAASSLALAIMMAGLSLWMCSNGS     560
LQCRICI                                                                              640
..........................N...........N.........................................      80
................................................................................     160
....................N...........................N...............................     240
.............................................................N..................     320
................................................................................     400
................................................................................     480
..................N.....................................................N..         560
.......                                                                              640

(Threshold=0.5)
----------------------------------------------------------------------
SeqName      Position  Potential   Jury    N-Glyc
                                 agreement result
----------------------------------------------------------------------
WRI00961.1     26 NNST   0.3687     (9/9)    --
WRI00961.1     27 NSTE   0.7794     (9/9)    +++
WRI00961.1     39 NVTV   0.7180     (9/9)    ++
WRI00961.1    181 NNTN   0.6099     (7/9)    +
WRI00961.1    209 NPTT   0.6336     (8/9)    +     WARNING: PRO-X1.
WRI00961.1    302 NSSM   0.5447     (6/9)    +
WRI00961.1    499 NGTY   0.5821     (5/9)    +
WRI00961.1    558 NGSL   0.6827     (9/9)    ++
----------------------------------------------------------------------
```

NetNGlyc 1.0: predicted N-glycosylation sites in WRI00961.1

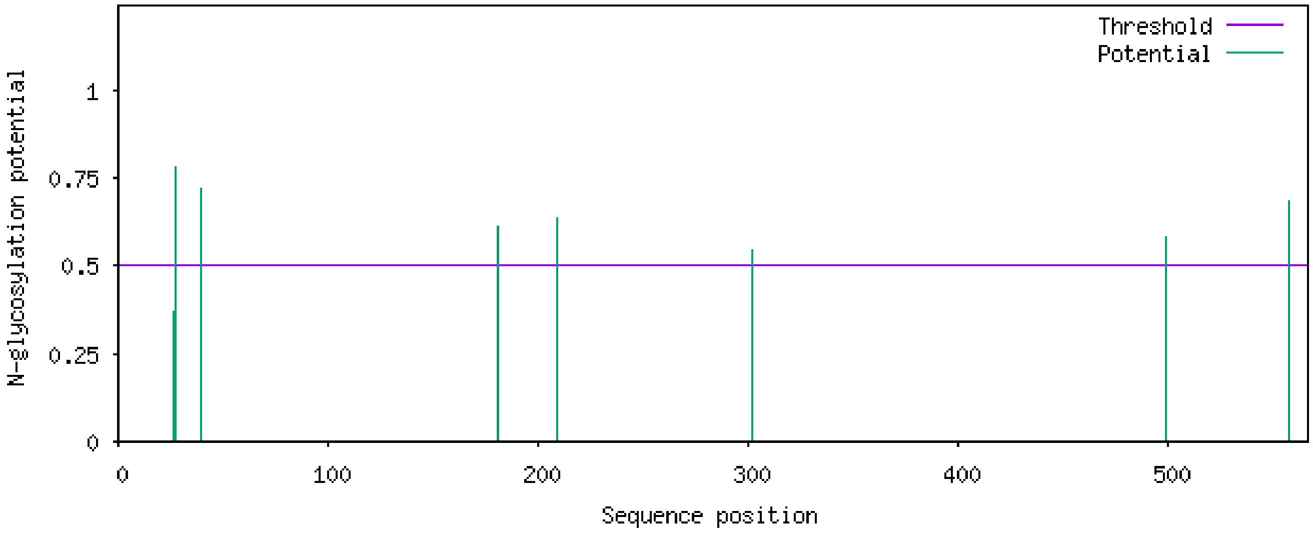

>WRI00963.1 neuraminidase [Influenza A virus duck/Bangladesh/58401/2023]

```
Name:  WRI00963.1       Length:  469
MNPNQRIITTGSICMVIGIVSLMLQIGNIISIWVSHSIQTGNQHHPGTCNQSIITYENNTWVNQTYVNINNTNFLAEQAV      80
TSVTLAGNSSLCPISGWAIYSKDNGIRIGSKGDVFVIREPFISCSHLECRTFFLTQGALLNDKHSNGTVKDRSPYRTLMS     160
CPVGEAPSPYNSRFESVAWSASACHDGISWLTIGISGPDNGAVAVLKYNGIITDTIKSWRNNILRTQESECACVNGSCFT     240
VMTDGPSNGQASYKIFKIEKGKVVKSVELNAPNYHYEECSCYPDAGEIMCVCRDNWHGSNRPWVSFNRNLEYQIGYICSG     320
VFGDNPRPNDGTGSCSPMSFNGAYGVKGFSFKYGNGVWIGRTKSTSSRSGFEMVWDPNGWTETDSSFSVKQDIIGITDWS     400
GYSGTFVQHPELTGLDCMRPCFWVELIRGRPKENTIWTSGSSISFCGVNSDTVGWSWPDGAELPFTIDK                480
.................................................N.......N....N......N..........      80
.......N.........................................................N..............     160
..........................................................................N.....     240
................................................................................     320
................................................................................     400
.....................................................................                480

(Threshold=0.5)
----------------------------------------------------------------------
SeqName      Position  Potential   Jury    N-Glyc
                                 agreement result
----------------------------------------------------------------------
WRI00963.1     50 NQSI   0.6834     (9/9)   ++
WRI00963.1     58 NNTW   0.5329     (6/9)   +
WRI00963.1     63 NQTY   0.6564     (9/9)   ++
WRI00963.1     70 NNTN   0.6889     (8/9)   +
WRI00963.1     88 NSSL   0.7723     (9/9)   +++
WRI00963.1    146 NGTV   0.6874     (9/9)   ++
WRI00963.1    235 NGSC   0.7321     (9/9)   ++
----------------------------------------------------------------------
```

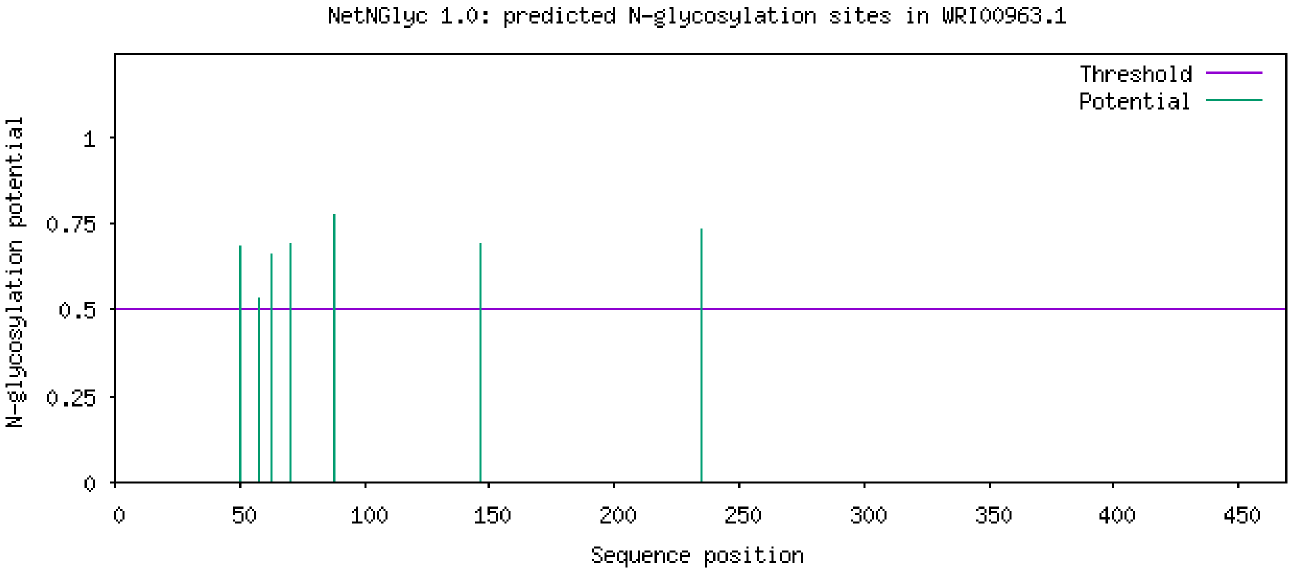

51. >WPZ50758.1 hemagglutinin [Influenza A virus/Chilean-dolphin/Maule/246026/2023]

```
Name:  WPZ50758.1       Length:  567
MENIVLLLAIVSLVKSDQICIGYHANNSTEQVDTIMEKNVTVTHAQDILEKTHNGKLCDLNGVKPLILKDCSVAGWLLGN      80
PMCDEFIRVPEWSYIVERANPANDLCYPGSLNDYEELKHMLSRINHFEKIQIIPKSSWPNHETSLGVSAACPYQGAPSFF     160
RNVVWLIKKNDAYPTIKISYNNTNREDLLILWGIHHSNNAEEQTNLYKNPTTYISVGTSTLNQRLAPKIATRSQVNGQRG     240
RMDFFWTILKPDDAIHFESNGNFIAPEYAYKIVKKGDSTIMKSGVEYGHCNTKCQTPVGAINSSMPFHNIHPLTIGECPK     320
YVKSNKLVLATGLRNSPLREKRRKRGLFGAIAGFIEGGWQGMVDGWYGYHHSNEQGSGYAADKESTQKAIDGVTNKVNSI     400
IDKMNTQFEAVGREFNNLERRIENLNKKMEDGFLDVWTYNAELLVLMENERTLDFHDSNVKNLYDKVRLQLRDNAKELGN     480
GCFEFYHKCDNECMESVRNGTYDYPQYSEEARLKREEISGVKLESVGTYQILSIYSTAASSLALAIMMAGLSLWMCSNGS     560
LQCRICI                                                                              640
..........................N...........N.........................................      80
................................................................................     160
....................N...........................N...............................     240
.............................................................N..................     320
................................................................................     400
................................................................................     480
..................N..........................................................N..     560
.......                                                                              640

(Threshold=0.5)
----------------------------------------------------------------------
SeqName      Position  Potential   Jury    N-Glyc
                                 agreement result
----------------------------------------------------------------------
WPZ50758.1    26 NNST   0.3686     (9/9)   --
WPZ50758.1    27 NSTE   0.7797     (9/9)   +++
WPZ50758.1    39 NVTV   0.7182     (9/9)   ++
WPZ50758.1   181 NNTN   0.6100     (7/9)   +
WPZ50758.1   209 NPTT   0.6336     (8/9)   +     WARNING: PRO-X1.
WPZ50758.1   302 NSSM   0.5447     (6/9)   +
WPZ50758.1   499 NGTY   0.5824     (6/9)   +
WPZ50758.1   558 NGSL   0.6827     (9/9)   ++
----------------------------------------------------------------------
```

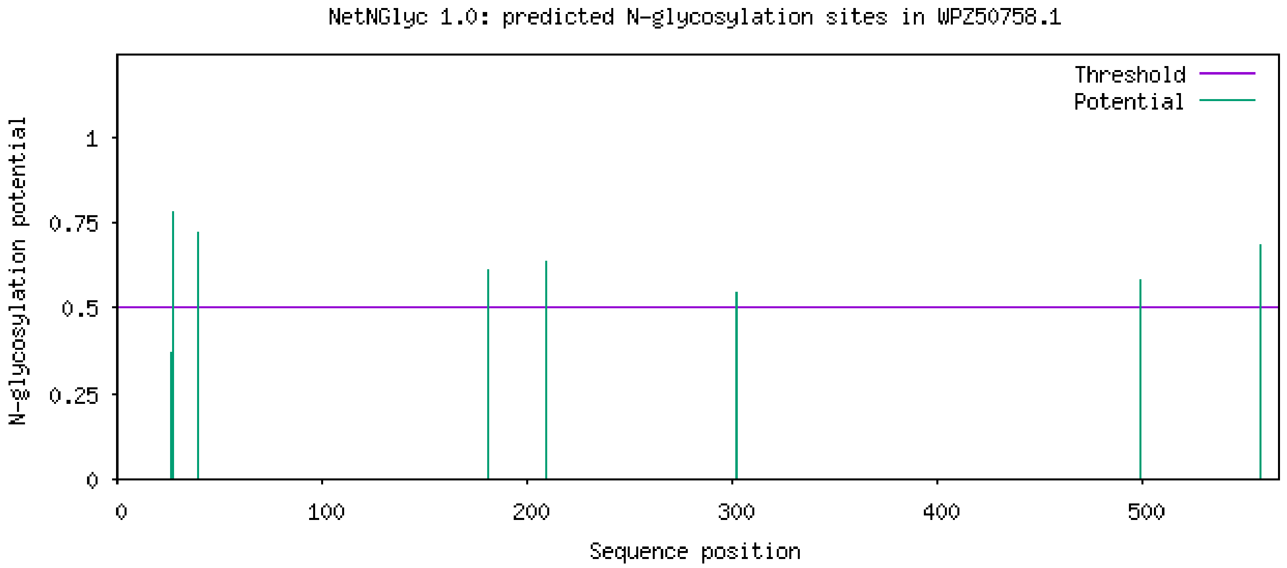

>WPZ50760.1 neuraminidase [Influenza A virus/Chilean dolphin/Maule/246026/2023]

```
Name:  WPZ50760.1      Length:  469
MNPNQKITTIGSICMVIGIVSLMLQIGNIISIWVSHSIQTGNQYQPEPCNQSIITYENNTWVNQTYVNISNTNFLAEQAV      80
TSVTLAGNSSLCPISGWAIYSKDNGIRIGSKGDVFVIREPFISCSHLECRTFFLTQGALLNDKHSNGTVKDRSPYRTLMS     160
CPVGEAPSPYNSRFESVAWSASACHDGISWLTIGISGPDNGAVAVLKYNGIITDTIKSWRNNILRTQESECACVNGSCFT     240
VMTDGPSNGQASYKIFKIEKGKVVKSVEMNAPNYHYEECSCYPDAGDIMCVCRDNWHGSNRPWVSFNQNLEYQIGYICSG     320
VFGDNPRPNDGTGSCSPMPSNGAYGVKGFSFKYGNGVWIGRTKSTSSRSGFEMIWDPNGWTETDSSFSVKQDIVEITDWS     400
GYSGSFVQHPELTGLDCMRPCFWVELIRGRPKENTIWTSGSSISFCGVNSDTVGWSWPDGAELPFTIDK                480
.................................................N.......N....N....N............      80
.......N.........................................................N..............     160
..........................................................................N.....     240
................................................................................     320
................................................................................     400
.....................................................................                480
```

(Threshold=0.5)

SeqName	Position		Potential	Jury agreement	N-Glyc result
WPZ50760.1	50	NQSI	0.5884	(8/9)	+
WPZ50760.1	58	NNTW	0.5496	(6/9)	+
WPZ50760.1	63	NQTY	0.6636	(9/9)	++
WPZ50760.1	68	NISN	0.7378	(9/9)	++
WPZ50760.1	88	NSSL	0.7724	(9/9)	+++
WPZ50760.1	146	NGTV	0.6876	(9/9)	++
WPZ50760.1	235	NGSC	0.7321	(9/9)	++

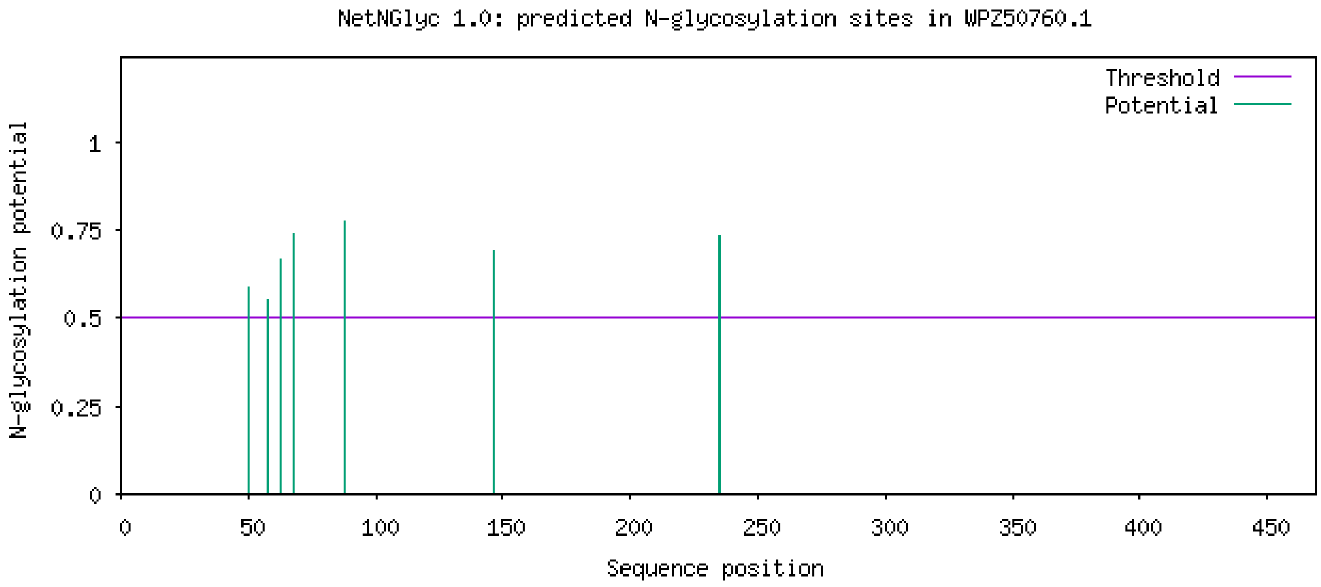

52. >XLY78253.1 hemagglutinin [Influenza A virus/Lousiana/Dec2024]

```
Name:  XLY78253.1       Length:  567
MENIVLLLAIISLVKSDQICIGYHANNSTEQVDTIMEKNVTVTHAQDILEKAHNGKLCDLNGVKPLILKDCSVAGWLLGN      80
PMCDEFIRVPEWSYIVERANPANDLCYPGSLNDYEELKHLLSRINHFEKILIIPKSSWPNHETSLGVSAACPYQGAPSFF     160
RNVVWLIKKNDAYPTIKISYNNTNREDLLILWGIHHSNNAEEQTNLYKNPTTYISVGTSTLNQRLVPKIATRSQVNGQRG     240
RMDFFWTILKPDDAIHFESNGNFIAPEYAYKIVKKGDSTIMKSGVEYGHCNTKCQTPVGAINSSMPFHNIHPLTIGECPK     320
YVKSNKLVLATGLRNSPLRERRRKRGLFGAIAGFIEGGWQGMVDGWYGYHHSNEQGSGYAADKESTQKAIDGVTNKVNSI     400
IDKMNTQFEAVGREFNNLERRIENLNKKMEDGFLDVWTYNAELLVLMENERTLDFHDSNVKNLYDKVRLQLRDNAKELGN     480
GCFEFYHKCDDECMESVRNGTYDYPQYSEEARLKREEISGVKLESIGTYQILSIYSTAASSLALAIMMAGLSLWMCSNGS     560
LQCRICI                                                                              640
.........................N............N.........................................      80
................................................................................     160
....................N...........................N...............................     240
.............................................................N..................     320
................................................................................     400
................................................................................     480
..................N..........................................................N..     560
.......                                                                              640

(Threshold=0.5)
----------------------------------------------------------------------
SeqName      Position  Potential   Jury    N-Glyc
                                agreement  result
----------------------------------------------------------------------
XLY78253.1     26 NNST   0.3684     (9/9)    --
XLY78253.1     27 NSTE   0.7794     (9/9)    +++
XLY78253.1     39 NVTV   0.7182     (9/9)    ++
XLY78253.1    181 NNTN   0.6100     (7/9)    +
XLY78253.1    209 NPTT   0.6336     (8/9)    +     WARNING: PRO-X1.
XLY78253.1    302 NSSM   0.5447     (6/9)    +
XLY78253.1    499 NGTY   0.5806     (6/9)    +
XLY78253.1    558 NGSL   0.6826     (9/9)    ++
----------------------------------------------------------------------
```

NetNGlyc 1.0: predicted N-glycosylation sites in XLY78253.1

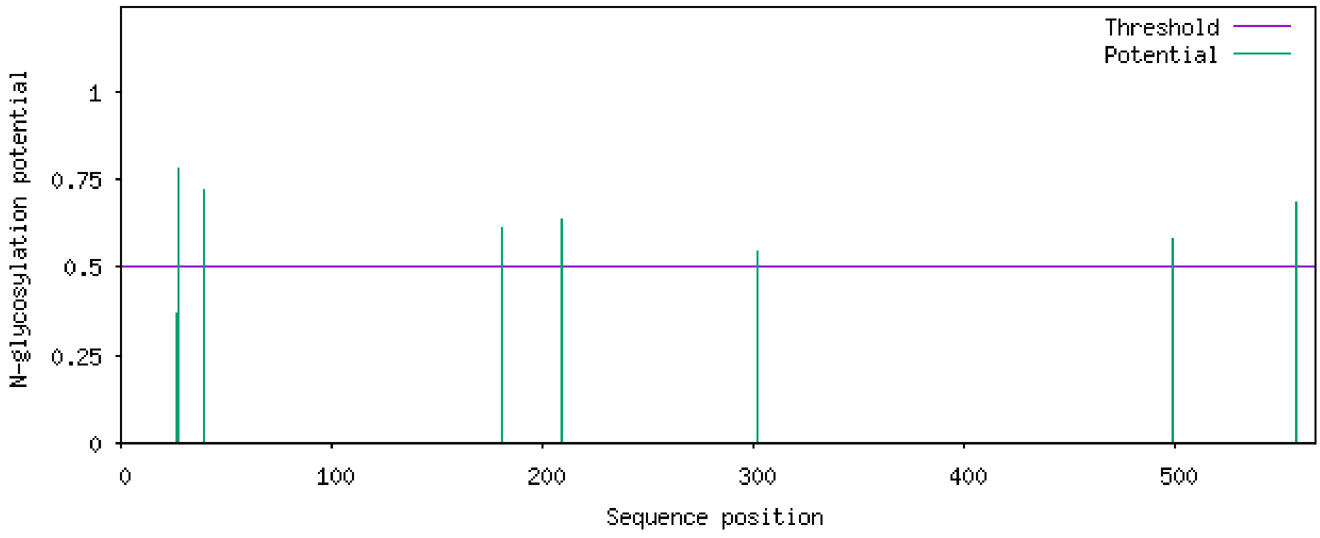

>XLY78254.1 neuraminidase [Influenza A virus/Lousiana/dec2024]

```
Name:  XLY78254.1       Length:  469
MNPNQKIITIGSICMVIGIISLVLQIGNIISIWVSHSIQTGNQNHPETCNQSVITYENNTWVNQTYINISNTNLIAEQAV      80
DPVALAGNSSLCPISGWAIYSKDNGIRIGSKGDVFVIREPFISCSHLECRTFFLTQGALLNDKHSNGTVKDRSPYRTLMS     160
CPVGEAPSPYNSRFESVAWSASACHDGISWLTIGISGPDNGAVAVLKYNGIITDTIKSWRSNILRTQESECACINGSCFT     240
IMTDGPSNGQASYKIFRIEKGKVVKSVELNAPNYHYEECSCYPDASEVMCVCRDNWHGSNRPWVSFNQNLEYQIGYICSG     320
VFGDNPRPSDGTGSCGPVSSNGAYGVKGFSFKYGNGVWIGRTKSTSSRSGFEMIWDPNGWTETDSSFSVKQDIVAITDWS     400
GYSGSFVQHPELTGLDCMRPCFWVELIRGRPKENTIWTSGSSISFCGVNSDTVGWSWPDGAELPFTIDK                480
.................................................N.......N....N....N............      80
.......N.........................................................N..............     160
..........................................................................N.....     240
................................................................................     320
................................................................................     400
.....................................................................                480

(Threshold=0.5)
----------------------------------------------------------------------
SeqName      Position  Potential   Jury     N-Glyc
                                 agreement  result
----------------------------------------------------------------------
XLY78254.1    50 NQSV   0.6007     (8/9)     +
XLY78254.1    58 NNTW   0.5473     (6/9)     +
XLY78254.1    63 NQTY   0.6926     (9/9)     ++
XLY78254.1    68 NISN   0.6721     (7/9)     +
XLY78254.1    88 NSSL   0.7411     (9/9)     ++
XLY78254.1   146 NGTV   0.6872     (9/9)     ++
XLY78254.1   235 NGSC   0.6750     (9/9)     ++
----------------------------------------------------------------------
```

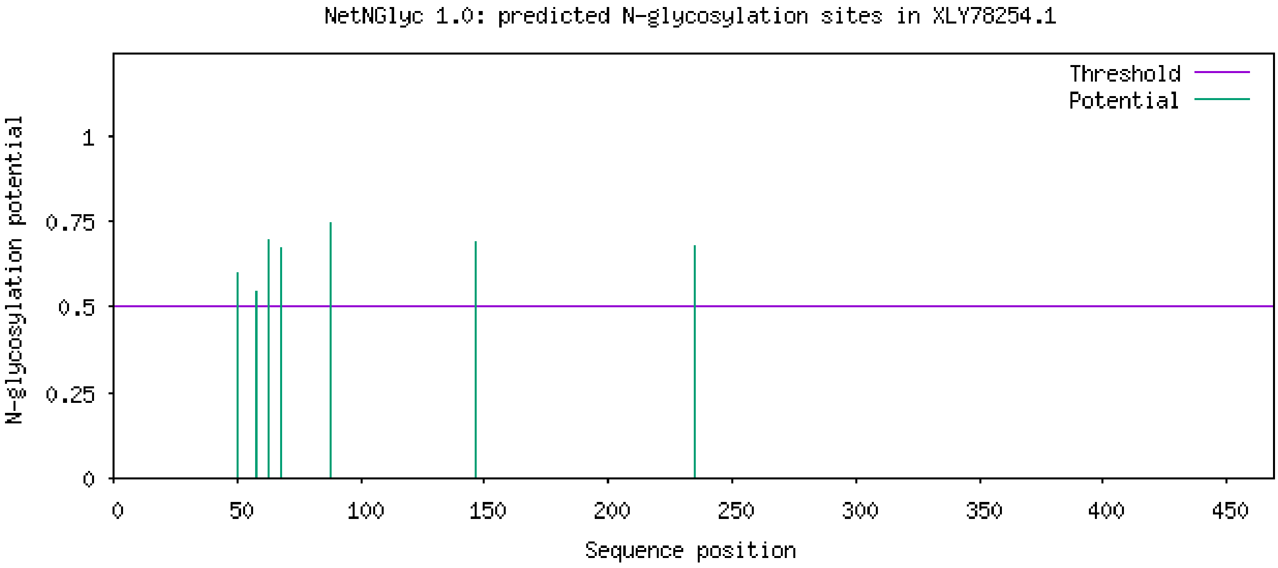

53. XAJ25928.1_HA_Influenza_A_virus_domestic-cat_Texas_24-009311-004_2024_ HA

```
Name:  XAJ25928.1_HA_Influenza_A_virus_domestic-cat_Texas_24-009311-004_2024_   Length:  56
MENIVLLLAIVSLVKSDQICIGYHANNSTEQVDTIMEKNVTVTHAQDILEKTHNGKLCDLNGVKPLILKDCSVAGWLLGN      80
PMCDEFIRVPEWSYIVERANPANDLCYPGSLNDYEELKHMLSRINHFEKIQIIPKSSWPNHETSLGVSAACPYQGAPSFF     160
RNVVWLIKKNDAYPTIKISYNNTNREDLLILWGIHHSNNAEEQTNLYKNPITYISVGTSTLNQRLAPKIATRSQVNGQRG     240
RMDFFWTILKPDDAIHFESNGNFIAPEYAYKIVKKGDSTIMKSGVEYGHCNTKCQTPVGAINSSMPFHNIHPLTIGECPK     320
YVKSNKLVLATGLRNSPLREKRRKRGLFGAIAGFIEGGWQGMVDGWYGYHHSNEQGSGYAADKESTQKAIDGVTNKVNSI     400
IDKMNTQFEAVGREFNNLERRIENLNKKMEDGFLDVWTYNAELLVLMENERTLDFHDSNVKNLYDKVRLQLRDNAKELGN     480
GCFEFYHKCDNECMESVRNGTYDYPQYSEEARLKREEISGVKLESVGTYQILSIYSTAASSLALAIMMAGLSLWMCSNGS     560
LQCRICI                                                                              640
..........................N...........N.........................................      80
................................................................................     160
....................N...........................................................     240
..............................................................N.................     320
................................................................................     400
................................................................................     480
..................N.......................................................N..      560
.......                                                                              640
```

(Threshold=0.5)

SeqName	Position	Potential	Jury agreement	N-Glyc result
XAJ25928.1_HA_Influenza_A_virus_domestic-cat_Texas_24-009311-004_2024_	26 NNST	0.3686	(9/9)	--
XAJ25928.1_HA_Influenza_A_virus_domestic-cat_Texas_24-009311-004_2024_	27 NSTE	0.7796	(9/9)	+++
XAJ25928.1_HA_Influenza_A_virus_domestic-cat_Texas_24-009311-004_2024_	39 NVTV	0.7182	(9/9)	++
XAJ25928.1_HA_Influenza_A_virus_domestic-cat_Texas_24-009311-004_2024_	181 NNTN	0.6100	(7/9)	+
XAJ25928.1_HA_Influenza_A_virus_domestic-cat_Texas_24-009311-004_2024_	302 NSSM	0.5447	(6/9)	+
XAJ25928.1_HA_Influenza_A_virus_domestic-cat_Texas_24-009311-004_2024_	499 NGTY	0.5826	(6/9)	+
XAJ25928.1_HA_Influenza_A_virus_domestic-cat_Texas_24-009311-004_2024_	558 NGSL	0.6827	(9/9)	++

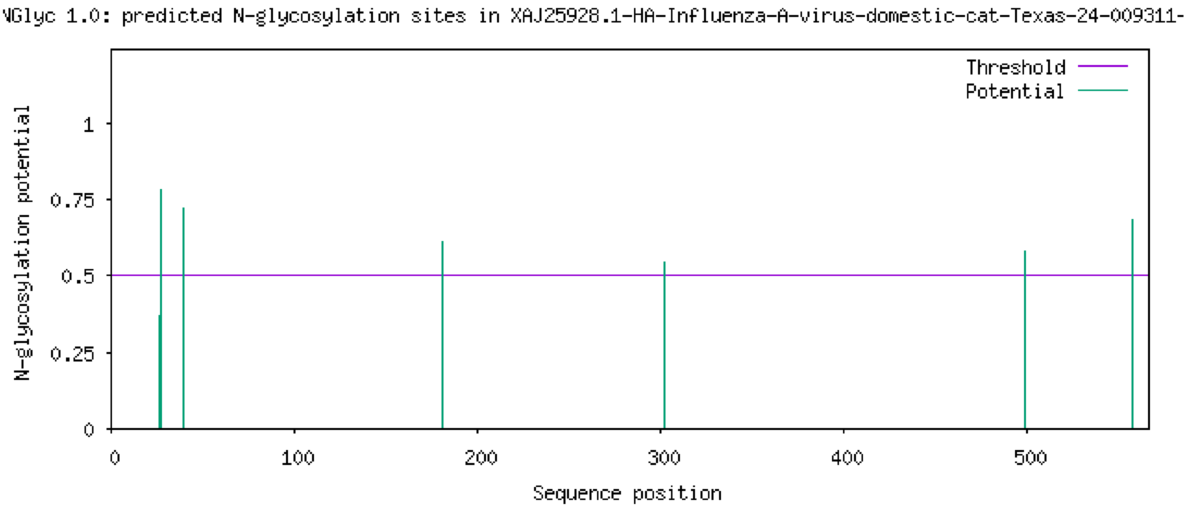

>XAJ25931.1_NA[Influenza_A_virus/domestic-cat/Texas/24-009311-004/2024]

```
Name:  XAJ25931.1_NA_Influenza_A_virus_domestic-cat_Texas_24-009311-004_2024_   Length:  469
MNPNQKITTIGSICMVIGIVSLMLQIGNIISIWVSHSIQTGNQYQPEPCNQSIITYENNTWVNQTYINISSTNFLAEQAV      80
TSVTLAGNSSLCPISGWAIYSKDNGIRIGSKGDVFVIREPFISCSHLECRTFFLTQGALLNDKHSNGTVKDRSPYRTLMS     160
CPVGEAPSPYNSRFESVAWSASACHDGISWLTIGISGPDNGAVAVLKYNGIITDTIKSWRNNILRTQESECACVNGSCFT     240
VMTDGPSNGQASYKIFKIEKGKVVKSVEMNAPNYHYEECSCYPDAGDIMCVCRDNWHGSNRPWVSFNQNLEYQIGYICSG     320
IFGDNPRPNDGTGSCSPMPSNGAYGVKGFSFKYGNGVWIGRTKSTSSRSGFEMIWDPNGWTETDSSFSVKQDIVEITDWS     400
GYSGSFVQHPELTGLDCMRPCFWVELIRGRPKENTIWTSGSSISFCGVNSDTVGWSWPDGAELPFTIDK     480
.................................................N.......N....N....N............      80
.......N.........................................................N..............     160
..........................................................................N.....     240
................................................................................     320
................................................................................     400
.....................................................................     480

(Threshold=0.5)
```

SeqName	Position	Potential	Jury agreement	N-Glyc result
XAJ25931.1_NA_Influenza_A_virus_domestic-cat_Texas_24-009311-004_2024_	50 NQSI	0.5883	(8/9)	+
XAJ25931.1_NA_Influenza_A_virus_domestic-cat_Texas_24-009311-004_2024_	58 NNTW	0.5251	(5/9)	+
XAJ25931.1_NA_Influenza_A_virus_domestic-cat_Texas_24-009311-004_2024_	63 NQTY	0.6874	(9/9)	++
XAJ25931.1_NA_Influenza_A_virus_domestic-cat_Texas_24-009311-004_2024_	68 NISS	0.7140	(9/9)	++
XAJ25931.1_NA_Influenza_A_virus_domestic-cat_Texas_24-009311-004_2024_	88 NSSL	0.7724	(9/9)	+++
XAJ25931.1_NA_Influenza_A_virus_domestic-cat_Texas_24-009311-004_2024_	146 NGTV	0.6873	(9/9)	++
XAJ25931.1_NA_Influenza_A_virus_domestic-cat_Texas_24-009311-004_2024_	235 NGSC	0.7321	(9/9)	++

NGlyc 1.0: predicted N-glycosylation sites in XAJ25931.1-NA-Influenza-A-virus-domestic-cat-Texas-24-009311-

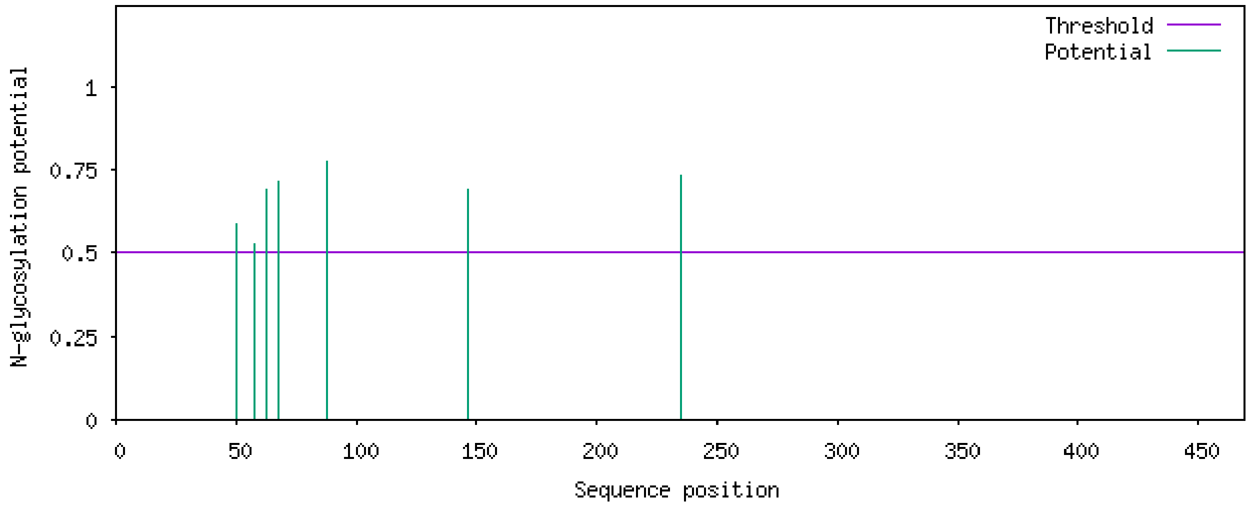

54. >XAJ25164.1_HA[Influenza_A_virus/A/goat/Minnesota/24-007234-006/2024]

```
Name:  XAJ25164.1_HA_Influenza_A_virus_A_goat_Minnesota_24-007234-006_2024_     Length:  567
MENIVLLLAIVSLVKSDQICIGYHANNSTEQVDTIMEKNVTVTHAQDILEKTHNGKLCDLNGVKPLILKDCSVAGWLLGN      80
PMCDEFIRVPEWSYIVERANPANDLCYPGSLNDYEELKHMLSRINHFEKIQIIPKSSWPNHETSLGVSAACPYQGAPSFF     160
RNVVWLIKKNDAYPTIKISYNNTNREDLLILWGIHHSNNAEEQTNLYKNPITYISVGTSTLNQRLAPKIATRSQVNGQRG     240
RMDFFWTILKPDDAIHFESNGNFIAPEYAYKIVKKGDSTIMKSGVEYGHCNTKCQTPVGAINSSMPFHNIHPLTIGECPK     320
YVKSNKLVLATGLRNSPLREKRRKRGLFGAIAGFIEGGWQGMVDGWYGYHHSNEQGSGYAADKESTQKAIDGVTNKVNSI     400
IDKMNTQFEAVGREFNNLERRIENLNKKMEDGFLDVWTYNAELLVLMENERTLDFHDSNVKNLYDKVRLQLRDNAKELGN     480
GCFEFYHKCDNECMESVRNGTYDYPQYSEEARLKREEIRGVKLESVGTYQILSIYSTAASSLALAIMMAGLSLWMCSNGS     560
LQCRICI                                                                              640
.........................N............N.........................................      80
................................................................................     160
....................N...........................................................     240
.............................................................N..................     320
................................................................................     400
................................................................................     480
..................N..........................................................N..     560
.......                                                                              640

(Threshold=0.5)
----------------------------------------------------------------------
SeqName      Position  Potential   Jury     N-Glyc
                                 agreement  result
----------------------------------------------------------------------
XAJ25164.1_HA_Influenza_A_virus_A_goat_Minnesota_24-007234-006_2024_   26 NNST   0.3686     (9/9)   --
XAJ25164.1_HA_Influenza_A_virus_A_goat_Minnesota_24-007234-006_2024_   27 NSTE   0.7796     (9/9)   +++
XAJ25164.1_HA_Influenza_A_virus_A_goat_Minnesota_24-007234-006_2024_   39 NVTV   0.7182     (9/9)   ++
XAJ25164.1_HA_Influenza_A_virus_A_goat_Minnesota_24-007234-006_2024_  181 NNTN   0.6100     (7/9)   +
XAJ25164.1_HA_Influenza_A_virus_A_goat_Minnesota_24-007234-006_2024_  302 NSSM   0.5447     (6/9)   +
XAJ25164.1_HA_Influenza_A_virus_A_goat_Minnesota_24-007234-006_2024_  499 NGTY   0.5824     (6/9)   +
XAJ25164.1_HA_Influenza_A_virus_A_goat_Minnesota_24-007234-006_2024_  558 NGSL   0.6828     (9/9)   ++
----------------------------------------------------------------------
```

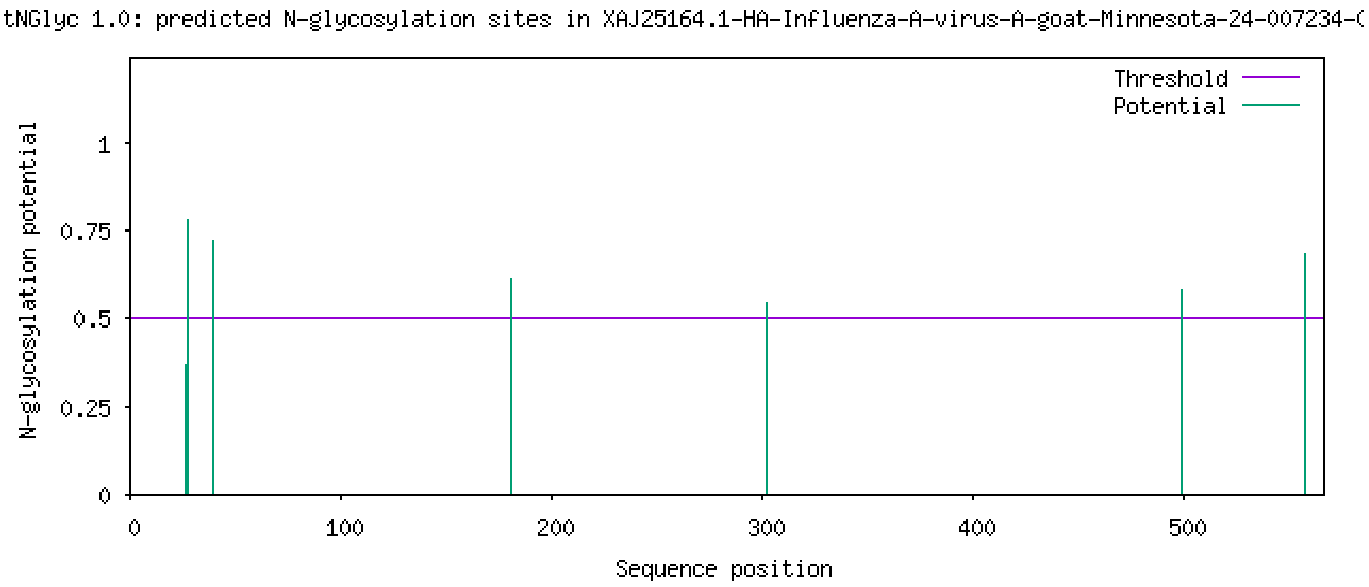

>XAJ25167.1_NA[Influenza_A_virus/goat/Minnesota/24-007234-006/2024]

```
Name:  XAJ25167.1_NA_Influenza_A_virus_goat_Minnesota_24-007234-006_2024_      Length:  469
MNPNQKITTIGSICMVIGIASLMLQIGNIISIWVSHSIQTGNQYQPEPCNQSIITYENNTWVNQTYVNISNTNFLAEQAV      80
TSVTLAGNSSLCPISGWAIYSKDNGIRIGSKGDVFVIREPFISCSHLECRTFFLTQGALLNDKHSNGTVKDRSPHRTLMS     160
CPVGEAPSPYNSRFESVAWSASACHDGISWLTIGISGPDNGAVAVLKYNGIITDTIKSWRNNILRTQESECACVNGSCFT     240
VMTDGPSNGQASYKIFKIEKGKVVKSVEMNAPNYHYEECSCYPDAGDIMCVCRDNWHGSNRPWVSFNQNLEYQIGYICSG     320
IFGDNPRPNDGTGSCSPMPSNGAYGVKGFSFKYGNGVWIGRTKSTSSRNGFEMIWDPNGWTETDSSFSVKQDIVEITDWS     400
GYSGSFVQHPELTGLDCMRPCFWVELIRGRPKENTIWTSGSSISFCGVNSDTVGWSWPDGAELPFTIDK     480
.................................................N.......N....N....N............      80
.......N.........................................................N..............     160
..........................................................................N.....     240
................................................................................     320
................................................................................     400
.....................................................................     480

(Threshold=0.5)
----------------------------------------------------------------------
SeqName      Position  Potential   Jury    N-Glyc
                                agreement result
----------------------------------------------------------------------
XAJ25167.1_NA_Influenza_A_virus_goat_Minnesota_24-007234-006_2024_   50 NQSI   0.5882     (8/9)   +
XAJ25167.1_NA_Influenza_A_virus_goat_Minnesota_24-007234-006_2024_   58 NNTW   0.5494     (6/9)   +
XAJ25167.1_NA_Influenza_A_virus_goat_Minnesota_24-007234-006_2024_   63 NQTY   0.6636     (9/9)   ++
XAJ25167.1_NA_Influenza_A_virus_goat_Minnesota_24-007234-006_2024_   68 NISN   0.7377     (9/9)   ++
XAJ25167.1_NA_Influenza_A_virus_goat_Minnesota_24-007234-006_2024_   88 NSSL   0.7724     (9/9)   +++
XAJ25167.1_NA_Influenza_A_virus_goat_Minnesota_24-007234-006_2024_  146 NGTV   0.6880     (9/9)   ++
XAJ25167.1_NA_Influenza_A_virus_goat_Minnesota_24-007234-006_2024_  235 NGSC   0.7320     (9/9)   ++
----------------------------------------------------------------------
```

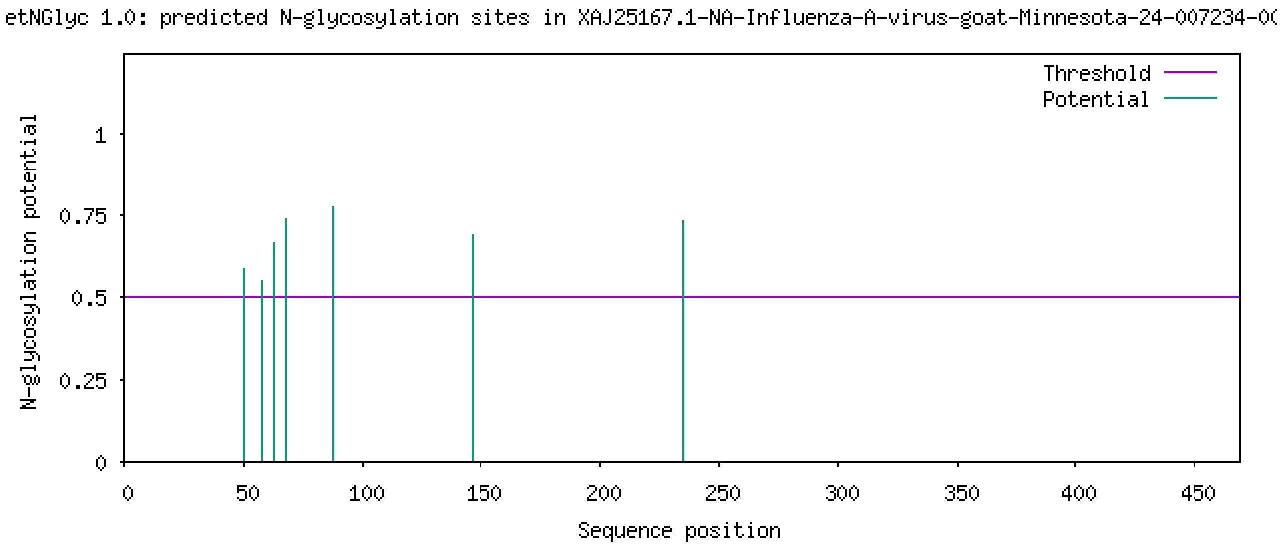

55. >XBA03461.1_HA[Influenza_A_virus/polar-bear/Alaska/23-038123/2023]

```
Name:  XBA03461.1_HA_Influenza_A_virus_polar-bear_Alaska_23-038123_2023_       Length:  567
MENIVLLLAIISLVKSDQICIGYHANNSTEQVDTIMEKNVTVTHAQDILEKAHNGKLCDLNGVKPLILKDCSVAGWLLGN      80
PMCDEFIRVPEWSYIVERANPANDLCYPGSLNDYEELKHLLSRINHFEKILIIPKSSWPNHETSLGVSAACPYQGAPSFF     160
RNVVWLIKKNDAYPTIKISYNNTNREDLLILWGIHHSNNAEEQTNLYKNPTTYISVGTSTLNQRLVPKIATRSQVNGQRG     240
RMDFFWTILKPDDAIHFESNGNFIAPEYAYKIVKKGDSTIMKSGVEYGHCNTKCQTPVGAINSSMPFHNIHPLTIGECPK     320
YVKSNKLVLATGLRNSPLRERRRKRGLFGAIAGFIEGGWQGMVDGWYGYHHSNEQGSGYAADKESTQKAIDGVTNKVNSI     400
IDKMNTQFEAVGREFNNLERRIENLNKKMEDGFLDVWTYNAELLVLMENERTLDFHDSNVKNLYDKVRLQLRDNAKELGN     480
GCFEFYHKCDDECMESVRNGTYDYPQYSGEARLKREEISGVKLESIGTYQILSIYSTAASSLALAIMMAGLSLWMCSNGS     560
LQCRICI                                                                              640
.........................N............N.........................................      80
................................................................................     160
....................N...........................N...............................     240
.............................................................N..................     320
................................................................................     400
................................................................................     480
..................N..........................................................N..     560
.......                                                                              640

(Threshold=0.5)
----------------------------------------------------------------------
SeqName      Position  Potential   Jury    N-Glyc
                                 agreement result
----------------------------------------------------------------------
XBA03461.1_HA_Influenza_A_virus_polar-bear_Alaska_23-038123_2023_   26 NNST   0.3684     (9/9)   --
XBA03461.1_HA_Influenza_A_virus_polar-bear_Alaska_23-038123_2023_   27 NSTE   0.7794     (9/9)   +++
XBA03461.1_HA_Influenza_A_virus_polar-bear_Alaska_23-038123_2023_   39 NVTV   0.7182     (9/9)   ++
XBA03461.1_HA_Influenza_A_virus_polar-bear_Alaska_23-038123_2023_  181 NNTN   0.6100     (7/9)   +
XBA03461.1_HA_Influenza_A_virus_polar-bear_Alaska_23-038123_2023_  209 NPTT   0.6336     (8/9)   +    WARNING: PRO-X1.
XBA03461.1_HA_Influenza_A_virus_polar-bear_Alaska_23-038123_2023_  302 NSSM   0.5447     (6/9)   +
XBA03461.1_HA_Influenza_A_virus_polar-bear_Alaska_23-038123_2023_  499 NGTY   0.5826     (6/9)   +
XBA03461.1_HA_Influenza_A_virus_polar-bear_Alaska_23-038123_2023_  558 NGSL   0.6827     (9/9)   ++
----------------------------------------------------------------------
```

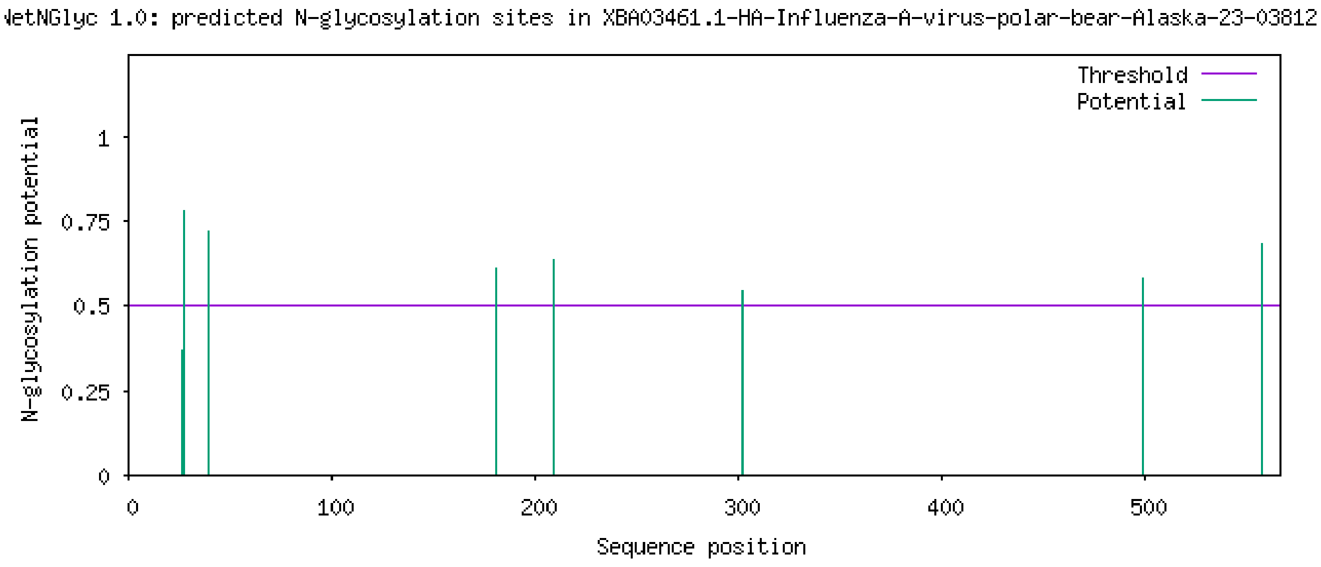

>XBA03463.1_NA[Influenza_A_virus/polar-bear/Alaska/23-038123/2023]

```
Name:  XBA03463.1_NA_Influenza_A_virus_polar-bear_Alaska_23-038123_2023_       Length:  469
MNPNQRIITTGSICMVIGIVSLMLQIGNIISIWVSHSIQTGNQYQPEPCNQSIIAYENNTWINQTYVNINNTNFLAEQAV      80
TSVTLAGNSSLCPISGWAIYSKDNGIRIGSKGDVFVIREPFISCSHLECRTFFLTQGALLNDKHSNGTVKDRSPYRTLMS     160
CPVGEAPSPYNSRFESVAWSASACHDGISWLTIGISGPDNGAVAVLKYNGIITDTIKSWRNNILRTQESECACVNGSCFT     240
VMTDGPSNGQASYKIFKIEKGKVVKSVELNAPNYHYEECSCYPDAGEIMCVCRDNWHGSNRPWVSFNQNLEYQIGYICSG     320
VFGDNPRPNDGTGSCSPMSSNGAYGVKGFSFKYGNGVWIGRTKSTSSRSGFEMIWDPNGWTETDSSFSVKQDIVAITDLS     400
GYSGTFVQHPELTGLDCMRPCFWVELIRGRPKENTVWTSGSSISFCGVNSDTVGWSWPDGAELPFTIDK                480
.................................................N.......N....N......N..........      80
.......N.........................................................N..............     160
..........................................................................N.....     240
................................................................................     320
................................................................................     400
.....................................................................                480

(Threshold=0.5)
----------------------------------------------------------------------
SeqName      Position  Potential   Jury    N-Glyc
                                agreement  result
----------------------------------------------------------------------
XBA03463.1_NA_Influenza_A_virus_polar-bear_Alaska_23-038123_2023_   50 NQSI   0.6061     (9/9)   ++
XBA03463.1_NA_Influenza_A_virus_polar-bear_Alaska_23-038123_2023_   58 NNTW   0.5741     (8/9)   +
XBA03463.1_NA_Influenza_A_virus_polar-bear_Alaska_23-038123_2023_   63 NQTY   0.5942     (9/9)   ++
XBA03463.1_NA_Influenza_A_virus_polar-bear_Alaska_23-038123_2023_   70 NNTN   0.6889     (8/9)   +
XBA03463.1_NA_Influenza_A_virus_polar-bear_Alaska_23-038123_2023_   88 NSSL   0.7724     (9/9)   +++
XBA03463.1_NA_Influenza_A_virus_polar-bear_Alaska_23-038123_2023_  146 NGTV   0.6873     (9/9)   ++
XBA03463.1_NA_Influenza_A_virus_polar-bear_Alaska_23-038123_2023_  235 NGSC   0.7320     (9/9)   ++
----------------------------------------------------------------------
```

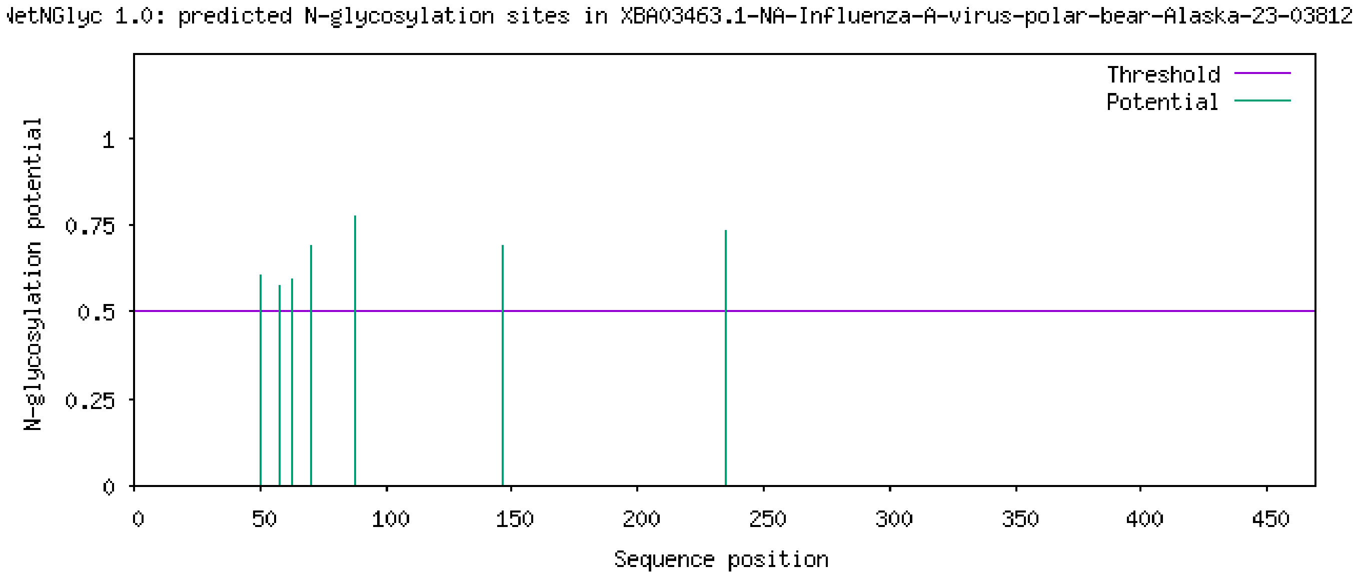

Supplement: Supplementary file 1 [file vetsci-12-00392-s001.zip › Suplementary 5 (S5).pdf]
